# Solid-Phase-Supported Chemoenzymatic Synthesis and Analysis of Chondroitin Sulfate Proteoglycan Glycopeptides

**DOI:** 10.1002/anie.202405671

**Published:** 2024-07-11

**Authors:** Po-han Lin, Yongmei Xu, Semiha Kevser Bali, Jandi Kim, Ana Gimeno, Elijah T. Roberts, Deepak James, Nuno M. S. Almeida, Narasimhan Loganathan, Fei Fan, Angela K. Wilson, I. Jonathan Amster, Kelley W. Moremen, Jian Liu, Jesús Jiménez-Barbero, Xuefei Huang

**Affiliations:** Department of Chemistry, Michigan State University, East Lansing, Michigan 48824, United States; Institute for Quantitative Health Science and Engineering, Michigan State University, East Lansing, Michigan 48824, United States; Division of Chemical Biology and Medicinal Chemistry, Eshelman, School of Pharmacy, University of North Carolina, Chapel Hill, North Carolina 27599, United States; Department of Chemistry, Michigan State University, East Lansing, Michigan 48824, United States; Department of Chemistry, University of Georgia, Athens, GA 30602, United States; Chemical Glycobiology Lab, Center for Cooperative Research in Biosciences (CICbioGUNE), Basque Research and Technology, Alliance (BRTA), 48160 Derio, Bizkaia, Spain; Ikerbasque, Basque Foundation for Science, Bilbao 48009, Spain; Department of Chemistry, University of Georgia, Athens, GA 30602, United States; Department of Chemistry, Michigan State University, East Lansing, Michigan 48824, United States; Department of Chemistry, Michigan State University, East Lansing, Michigan 48824, United States; Department of Chemistry, Michigan State University, East Lansing, Michigan 48824, United States; Institute for Quantitative Health Science and Engineering, Michigan State University, East Lansing, Michigan 48824, United States; Department of Chemistry, Michigan State University, East Lansing, Michigan 48824, United States; Department of Chemistry, Michigan State University, East Lansing, Michigan 48824, United States; Department of Chemistry, University of Georgia, Athens, GA 30602, United States; Department of Biochemistry & Molecular Biology, University of Georgia, Athens, GA 30602, United States; Complex Carbohydrate Research Center, University of Georgia, Athens, GA 30602, United States; Division of Chemical Biology and Medicinal Chemistry, Eshelman, School of Pharmacy, University of North Carolina, Chapel Hill, North Carolina 27599, United States; Chemical Glycobiology Lab, Center for Cooperative Research in Biosciences (CICbioGUNE), Basque Research and Technology, Alliance (BRTA), 48160 Derio, Bizkaia, Spain; Ikerbasque, Basque Foundation for Science, Bilbao 48009, Spain; Department of Inorganic & Organic Chemistry, Faculty of Science and Technology, University of the Basque Country, EHU-UPV, Leioa 48940, Spain; Centro de Investigación Biomédica en Red de Enfermedades, Respiratorias, Madrid 28029, Spain; Institute for Quantitative Health Science and Engineering, Michigan State University, East Lansing, Michigan 48824, United States; Department of Biomedical Engineering, Michigan State University, East Lansing, Michigan 48824, United States; Department of Chemistry, Michigan State University, East Lansing, Michigan 48824, United States

**Keywords:** chondroitin sulfate glycopeptide, enzymes, proteoglycan, solid phase, synthesis

## Abstract

Proteoglycans (PGs), consisting of glycosaminoglycans (GAGs) linked with the core protein through a tetrasaccharide linkage region, play roles in many important biological events. The chemical synthesis of PG glycopeptides is extremely challenging. In this work, the enzymes required for synthesis of chondroitin sulfate (CS) PG (CSPG) have been expressed and the suitable sequence of enzymatic reactions has been established. To expedite CSPG synthesis, the peptide acceptor was immobilized on solid phase and the glycan units were directly installed enzymatically onto the peptide. Subsequent enzymatic chain elongation and sulfation led to the successful synthesis of CSPG glycopeptides. The CS dodecasaccharide glycopeptide was the longest homogeneous CS glycopeptide synthesized to date. The enzymatic synthesis was much more efficient than the chemical synthesis of the corresponding CS glycopeptides, which could reduce the total number of synthetic steps by 80%. The structures of the CS glycopeptides were confirmed by mass spectrometry analysis and NMR studies. In addition, the interactions between the CS glycopeptides and cathepsin G were studied. The sulfation of glycan chain was found to be important for binding with cathepsin G. This efficient chemoenzymatic strategy opens new avenues to investigate the structures and functions of PGs.

## Introduction

Proteoglycans (PGs), a family of glycoproteins, are commonly found on cell surfaces and within the extracellular matrix. They play pivotal roles in many biological events including cell proliferation, inflammation, and viral infection.^[1–5]^ PGs contain one or more glycosaminoglycan (GAG) chains covalently conjugated to serine (Ser) residues on a core protein backbone through a typical tetrasaccharide linkage with the sequence of glucuronic acid (GlcA)-β1–3-galactose (Gal)-β1–3-galactose (Gal)-β1–4-xylose (Xyl)-β1-Ser (GlcAβ1–3Galβ1–3Galβ1–4Xylβ1-Ser). The GAG chain of proteoglycans can be chondroitin sulfate (CS) or heparan sulfate (HS) forming CS proteoglycan (CSPG) or HS proteoglycan with sulfations at various hydroxyl groups of the glycan chains.^[6]^ The sulfation patterns of naturally existing PGs are heterogeneous, resulting in large structural diversities.

Traditionally, the biological functions of PGs are thought to be generally directed by the glycan chains attached.^[7–8]^ However, an increasing body of research has suggested that the core protein can be important as well,^[9–11]^ with the core protein and the glycan chain potentially exhibiting synergistic effects for the biological functions of PGs.^[12–14]^ To gain deeper insights into the functions of PGs and decipher the respective roles of the glycan and the core protein, it is imperative to obtain structurally well-defined and homogeneous glycopeptides and PGs.

With their high heterogeneity, it is almost impossible to purify homogeneous PG structures from natural sources. Several strategies have been developed for the syntheses of HS and CS bearing glycopeptides containing the native tetrasaccharide linkage region.^[14–16]^ The chemical synthesis of GAG-bearing glycopeptides is a formidable challenge, stemming from the intricate series of protecting group manipulation required, glycosylation reactions, chemical sulfation, and the incompatibilities between typical peptide and GAG synthesis conditions.^[17–22]^ The longest HS and CS glycopeptides prepared to date bear octasaccharides on the peptide backbones.^[23–24]^ These syntheses were tedious with the total number of synthetic steps needed well over 100 for some of the targets.^[23]^ Recently, the Huisgen alkyneazide cycloaddition reactions were utilized to conjugate HS with protein backbones bearing alkynyl tyrosine.^[12]^ While ground-breaking, these PG mimetics contain heterogeneous glycans and the glycan chain was linked through an unnatural triazole moiety to tyrosine in the core protein.

To greatly expedite the synthesis of the native tetrasaccharide linkage region and CS-bearing glycopeptide, in this study, we introduce a chemoenzymatic method facilitated by solid phase support. The underlying principle involves the cloning and expression of the enzymes required for PG synthesis. This is followed by conjugation of the peptide backbone onto Sepharose beads (crosslinked agarose-based bead) with subsequent successive rounds of enzymatic extensions and modifications. The native tetrasaccharide linkage region and CS-bearing glycopeptides formed were then released under a mild reaction condition without affecting the sensitive glycopeptides. Leveraging this powerful strategy, we successfully generated multiple tetrasaccharide linkage-bearing glycopeptides bearing diverse amino acid sequences in the backbone, as well as CS-bearing glycopeptides of varying lengths in mg scales. The availability of these defined glycopeptides enabled structural analysis by NMR and MS. In addition, the affinities of the glycopeptides with a potential binder, cathepsin G (CatG)^[25]^ were investigated and rationalized with computational modeling, demonstrating the important role of glycan sulfation for CatG binding.

## Results and Discussion

There are multiple challenges in establishing a viable enzymatic route for PG synthesis, which include the identification of suitable enzymes to catalyze the synthesis, the production of the enzymes, and the time-consuming process of isolating the highly polar product from the aqueous reaction media. In order to expedite the synthesis and reduce the time needed to purify the highly polar glycopeptides, we investigated the possibility of performing enzymatic synthesis of PG glycopeptides on solid phase.^[26–28]^ Various solid supports have been reported for enzymatic synthesis of glycans or glycopeptides, which include polyethylene glycol polyacrylamide copolymer (PEGA),^[29]^ amine-functionalized silica,^[28]^ controlled pore glass (CPG),^[30]^ and thermo-responsive water-soluble polymers.^[31–32]^ While they have demonstrated compatibility with enzymes, each of the solid supports needs unique consideration. For instance, swelling resins like PEGA, due to their limited pore size, may prove insufficient for reactions requiring enzymes with molecular weight higher than 50 kDa.^[32]^ Conversely, non-swelling solid supports such as amine-functionalized silica and CPG may exhibit less compatibility with certain enzymes compared to swelling solid supports.^[32]^ Thermo-responsive water-soluble polymers can provide solution-like environment for enzymatic synthesis, while allowing precipitation of the product from the reaction media through heating after the reaction.^[33–34]^ However, when the glycan becomes charged after reactions, the polymer can no longer be precipitated from the solution upon heating.^[34]^ Thus, it may not be suitable for PG synthesis due to the highly negative charged GAG sequences on PG.

To enable the chemo-enzymatic synthesis of PG glycopeptides, we explored Sepharose^[35]^ as a potential solid phase support. Sepharose possesses commendable swelling properties in aqueous buffers with large pore sizes (~20,000 kDa), and is commonly employed for protein purification.^[36]^ PG glycopeptides exhibit limited stability under strongly acidic or basic conditions, primarily due to the susceptibility of glycopeptides to undergo glycan elimination under a basic condition,^[18,22]^ and the potential for sulfate loss under an acid condition. Consequently, a linker that can be cleaved under a mild condition, is imperative for solid phase synthesis. We opted to utilize diethyl squarate as a traceless linker^[37]^ as it can yield the native glycopeptide after cleavage from the resin.

### Enzymatic Syntheses of Tetrasaccharide Linkage Region Bearing Glycopeptides

**Scheme 1.**
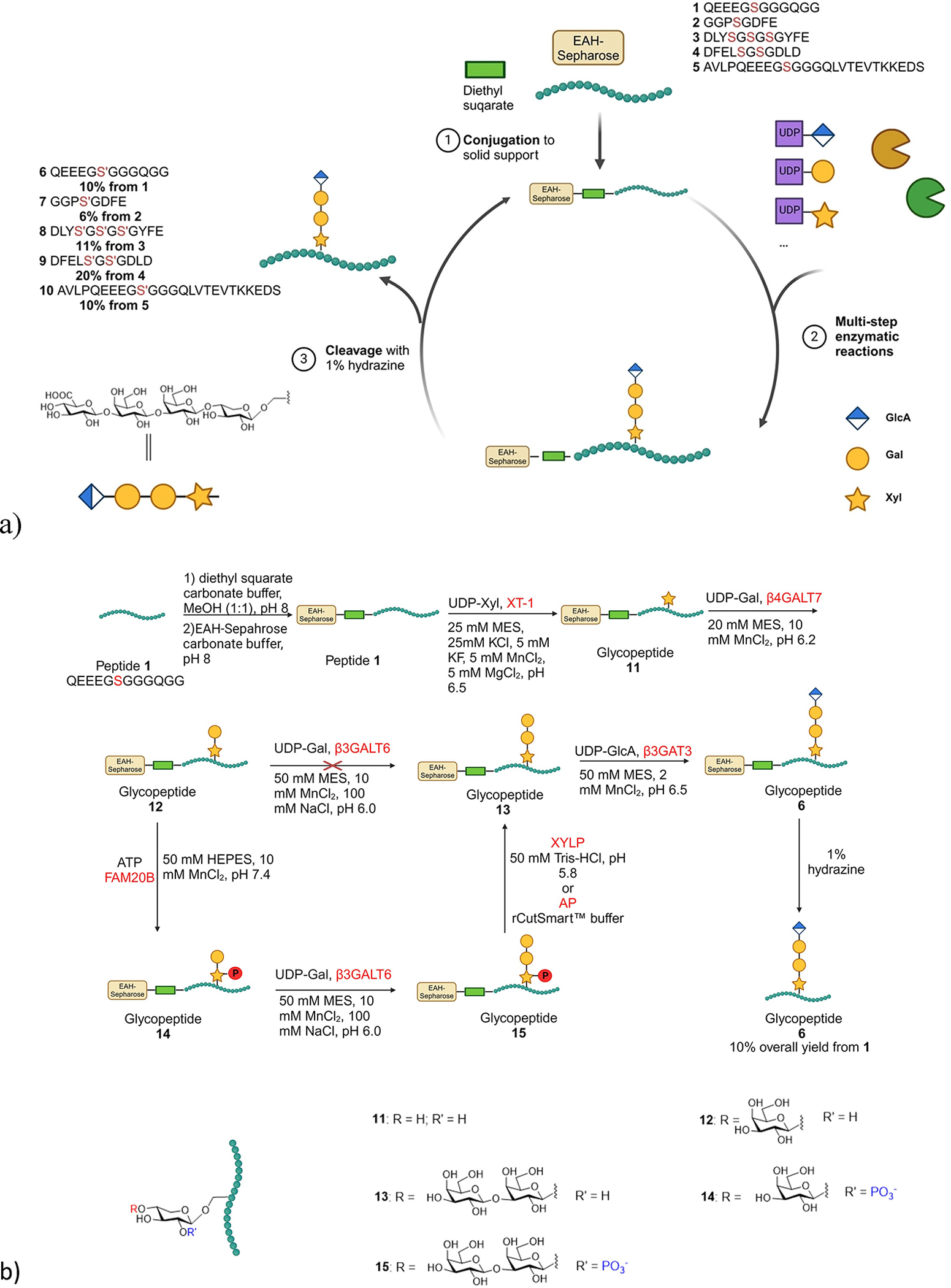
(a) Schematic demonstration of the solid phase supported enzymatic synthesis of tetrasaccharide linkage region bearing glycopeptides **6**–**10**. The serine glycosylation sites are highlighted in red for each peptide/glycopeptide sequence; (b) Sepharose supported enzymatic synthesis of glycopeptide **6** from peptide **1**. This Figure was created using BioRender with permission.

To establish the feasibility of solid phase supported enzymatic synthesis of PG glycopeptide, the peptide acceptor was functionalized through its *N*-terminal amine with diethyl squarate in a mixed solvent of carbonate buffer and methanol (Scheme 1a). After 6 hours, liquid chromatography mass spectrometry (LCMS) analysis confirmed the formation of the desired squarate-modified peptide with the complete consumption of the free peptide. Subsequently, the reaction mixture was incubated with EAH Sepharose, a commercially available Sepharose resin with an 11-atom hydrophilic spacer arm from the surface (2 equiv. based on free amine on the Sepharose to the peptide), in a carbonate buffer. The resulting slurry was kept in a frit-fitted syringe and agitated with end-to-end rotation for 24 hours, when LCMS analysis indicated no free squarate-modified peptide remained in solution.

In order to establish the cleavage conditions, we first treated the peptide **1** loaded Sepharose with boric acid in combination with concentrated ammonia.^[35]^ This yielded some desired peptide product along with squarate-modified peptide as a side produce based on LCMS analysis (data not shown). This method also generated a notable amount of ammonium borate salt, adversely affecting subsequent high performance liquid chromatography (HPLC) purification. The second approach investigated utilized 5% aqueous hydrazine.^[33]^ Interestingly, while it cleaved the peptide from Sepharose, it also led to an undesired side product with the *N*-terminal amino acid residue removed. Reducing the concentration of hydrazine to 1% completely mitigated this side reaction.

With the solid phase immobilization and cleavage conditions established, we moved on to express the requisite enzymes to form the glycosyl bonds in the linkage region,^[38–39]^ which included the xylosyl transferase-1 (XT-1),^[40–41]^ β1,4-galactosyl transferase 7 (β4GALT7),^[42–44]^ β1,4-galactosyl transferase 6 (β3GALT6),^[45]^ and β1,3-glucuronic acid transferase 3 (β3GAT3).^[46]^ We found that β4GALT7 and β3GAT3 could be expressed well in *E. coli* with the yields of 16.7 and 5 mg/L respectively, while XT-1 and β3GALT6 should be expressed in the HEK293F cells with the desired enzymes isolated from the supernatant in 10, 2.3 and 16.7 mg/L respectively.

Solid-phase enzymatic syntheses have been traditionally performed using a glycosylated peptide as the substrate to initiate enzymatic reactions.^[26,28–29,32]^ This strategy typically commences from chemical synthesis of the glycosylated amino acid cassette followed by its incorporation into the glycopeptide chain, which requires the usage of an excess of the valuable glycosyl amino acid building blocks. As an alternative, we opted to explore the direct glycosylation of the peptide on solid phase. The Sepharose resin loaded with peptide **1** was treated with uridine diphosphate (UDP)-Xyl and XT-1 (MW: 87 kDa) in a reaction buffer comprising 25 mM 2-(*N*-morpholino)ethanesulfonic acid (MES), 25 mM KCl, 5 mM KF, 5 mM MgCl_2_, 5 mM MnCl_2_, at pH 6.5 over a 12 hour period with end-over-end rotation at 4°C, which was repeated once to ensure complete conversion (Scheme 1b). The crude product was subjected to cleavage using 1% hydrazine solution in water followed by analysis via LCMS, which showed the desired target glycopeptide **11** as the sole product. With the confirmation of the successful xylosylation, the xylosylated glycopeptide on Sepharose was incubated with UDP-Gal and β4GALT7 in 20 mM MES and 10 mM MnCl_2_, at pH 6.2 at 4°C (Scheme 1b). Subsequent treatment of the Sepharose with 1% hydrazine gave glycopeptide **12** as the desired product suggesting the successful transfer of the Gal unit to xylosyl peptide **12**. Next, we tested the transfer of a second Gal to the linkage region. Unfortunately, upon incubation of the Gal-Xyl glycopeptide **12** bearing Sepharose with UDP-Gal and β3GALT6, no desired trisaccharide Gal-Gal-Xyl glycopeptide **13** was obtained. This failure was not due to the solid phase support as treatment of free glycopeptide **12** with UDP-Gal and β3GALT6 in solution also failed to yield the desired trisaccharide glycopeptide **13**.

The enzyme Family With Sequence Similarity 20 Member B (FAM20B) is a kinase capable of phosphorylating the 2-OH of xylose in the tetrasaccharide linkage region.^[47]^ It is known to act as a molecular switch regulating the functions of β3GALT6.^[38,48]^ To enhance the glycopeptide synthesis yield, disaccharide glycopeptide **12** on Sepharose was subjected to the phosphorylation condition with adenosine triphosphate (ATP) and FAM20B in 50 mM *N*-(2-hydroxyethyl)piperazine-*N’*-(2-ethanesulfonic acid) (HEPES) and 10 mM MnCl_2_ buffer at pH 7.4. This was followed by the treatment with UDP-Gal and β3GALT6 (Scheme 1b). Gratifyingly, cleavage of the glycopeptide from the Sepharose following this sequence of reactions showed the successful formation of the phosphorylated Gal-Gal-Xyl bearing glycopeptide **15**. This suggests FAM20B can phosphorylate a glycopeptide attached on solid phase and confirmed that the 2-*O* phosphorylation significantly enhanced the yield of Gal transfer by β3GALT6. The β3GALT6^[49]^ utilized is a truncated form of the protein comprising amino acids 35 to 329. This is the first demonstration that such a construct is enzymatically active with its activities regulated by FAM20B.

To regenerate the non-phosphorylated glycan, we expressed 2-phosphoxylose phosphatase (XYLP),^[50]^ which dephosphorylated glycopeptide **15** to glycopeptide **13**. As XYLP was expressed in HEK293F cells, we explored the potential replacement of XYLP with the commercially available alkaline phosphatase (AP) to reduce the costs associated with mammalian cell expression. Interestingly, AP could also efficiently dephosphorylate glycopeptide **15** on Sepharose. Subsequently, trisaccharide glycopeptide **13** was extended on Sepharose with β3GAT3 with UDP-GlcA as the donor in 50 mM MES, 2 mM MnCl_2_, at pH 6.5 producing glycopeptide **6** bearing the full tetrasaccharide linkage region. The overall yield from peptide **1** to glycopeptide **6** was 10%, which is an average of 77% yield per synthetic step.

With the successful enzymatic synthesis of glycopeptide **6** on solid phase, we tested the generality of the approach with several other representative peptide substrates **2**–**5** (Scheme 1a), which contain a variety of amino acid residues including acidic, basic, aromatic, and aliphatic amino acids flanking the glycosylation sites. These peptide sequences were derived from common proteoglycans in nature, which include syndecan 3 (peptides **2** and **3**), syndecan 4 (peptide **4**) and bikunin (peptides **1** and **5**). Peptides **2** and **3** have two glycosylation sites each, while peptide **4** has three glycosylation sites. Gratifyingly, following the same reaction protocol on Sepharose for the synthesis of glycopeptide **6**, peptides **2**–**5** were successfully converted to glycopeptides **7**–**10**, each bearing the full tetrasaccharide linkage regions in 6%, 11%, 20%, and 10% yields respectively demonstrating the robustness of the synthetic protocol. For each glycopeptide synthesis, it took nine steps from the peptide backbone. In comparison, chemical synthesis of a tetrasaccharide linkage region bearing peptide took 39 total synthetic steps and 1.1% yield for the longest linear steps (27 steps) from the peptide and commercially available carbohydrate building blocks.^[16]^

### Synthesis of Chondroitin Sulfate Glycopeptides

Bikunin, also known as inter-α-trypsin inhibitor or trypstatin, is a naturally existing CSPG.^[51]^ Initially discovered in urine and human plasma, it is implicated in various biological activities for anti-inflammation and cancer,^[52–55]^ and has been utilized to treat acute inflammatory disorders including sepsis.^[56–57]^ With the tetrasaccharide linkage region bearing glycopeptide **10** in hand, we proceed to synthesize homogeneous bikunin glycopeptide.

**Scheme 2.**
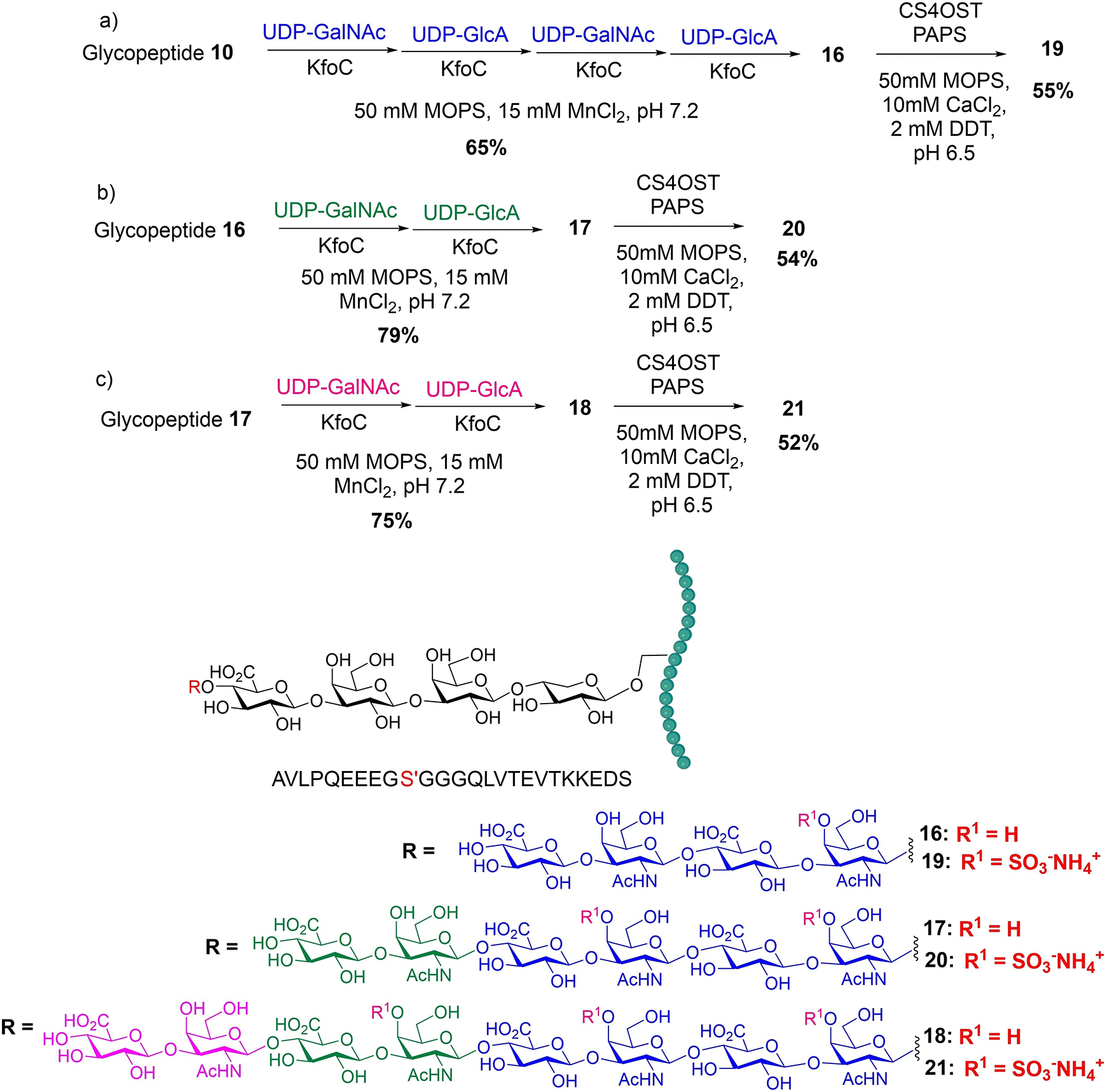
Enzymatic syntheiss of (a) CS octasaccharide glycopeptide **19**; (b) CS decasaccharide glycopeptide **20**; (c) CS dodecasaccharide glycopeptide **21**.

In nature, the synthesis of CSPG is directed by the immediate sugar residue added to the linkage region, with the transfer of an *N*-acetyl galactosamine (GalNAc) residue to the tetrasaccharide linkage region by the GalNAc transferase-1 (GalNAcT-I),^[58]^ initiating CS synthesis. The glycan chain is further extended by the GlcA transferase^[59]^ and the GalNAc transferase^[60]^ forming the CS backbone. Subsequetly, various sulfo-transferases will selectively install *O*-sulfates onto the CS backbone forming CSPG. KfoC is a bacterial enzyme involved in the synthesis of the capsular chondroitin backbone of *Escherichia coli* K4, which is bifunctional capable of transferring both GlcA and GalNAc to a chondroitin chain.^[61]^ Rather than expressing GalNAcT-I, we tested KfoC’s ability to direct the synthesis of CS glycopeptide. As illustrated in Scheme 2a, glycopeptide **10** was first treated with UDP-GalNAc and KfoC in 50 mM 3-(*N*-morpholino)propanesulfonic acid (MOPS) and 15 mM MnCl_2_ buffer at pH 7.2, followed by solid phase extraction through C18 silica gel. The resulting fractions containing the desired product were lyophilized and then subjected to treatment with UDP-GlcA and KfoC. This process was repeated two more times with the alternating usage of UDP-GalNAc and UDP-GlcA, producing octasaccharide chondroitin glycopeptide **16** in 65% yield from tetrasaccharide glycopeptide **10**. This suggests that KfoC can be useful to not only extend the chondroitin backbone, but also initiate the formation of chondrotin backone from the linkage region to enable CS glycoeptide synthesis.

With the octasaccharide glycopeptide **16** in hand, its glycan chain was further extended by alternating UDP-GalNAc and UDP-GlcA in the presence of KfoC, generating chondroitin glycopeptides **17** and **18** bearing decasaccharide and dodecasaccharide chain in 79% and 75% yields respectively (Schemes 2b and 2c). To synthesize CS glycopeptides, glycopeptides **16**, **17** and **18** were subjected to 4-*O* sulfation using 3’-phosphoadenosine-5’-phosphosulfate (PAPS) and CS 4-*O* sulfotransferase (CS4OST)^[62]^ (Scheme 2). The corresponding *O*-sulfated glycopeptides **19**, **20** and **21** were isolated using C18 reverse phase HPLC in 55%, 54%, and 52% yields respectively. The dodecasaccharide glycopeptide **21** bears three *O*-sulfates, which is the longest GAG-bearing glycopeptide synthesized to date.

**Scheme 3.**
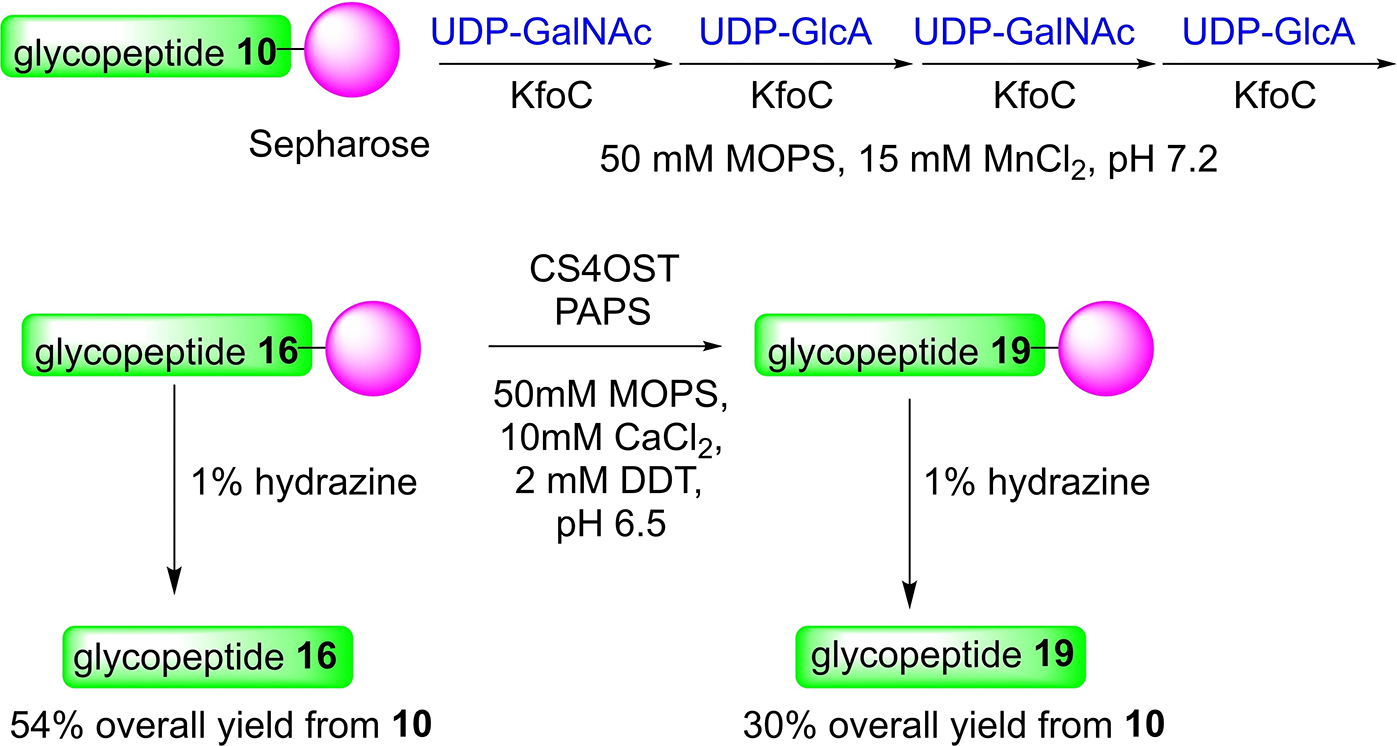
Solid phase supported synthesis of CS glycopeptide **19**.

To test the possibility of synthesizing the CS chain on solid phase support, we incubated the glycopeptide **10** attached Sepharose with KfoC and alternating UDP-GalNAc, UDP-GlcA, UDP-GalNAc, and UDP-GlcA followed by cleaveage from the solid phase with 1% hydrazine (Scheme 3). The glycopeptide **16** was obtained in 54% overall yield from **10** in 4 days. The sulfation reactions could be performed on solid phase as well. Treatment of Sepharose bearing **16** with PAPS and CS4OST followed by 1% hydrazine cleavage led to the CS octasaccharide glycopeptide **19** from **10** in 30% overall yield from glycopeptide **10**. While the overall yield of the solid phase supported synthesis of **19** was similar to that from solution based synthesis (Scheme 2), solid phase synthesis cut down the amount of time needed for synthesis by about 50% as it reduced the need for the time consuming intermediate purification and lyophilization for water removal and sample concentration.

Previously, a CS octasaccharide bearing glycopeptide was synthesized via chemical synthesis using a convergent strategy.^[24]^ From commerically available carbohydrate building blocks, it took more than 80 synthetic steps in total to complete with an overall yield of 0.73% for the longest linear synthetic sequence of 26 steps. In comparison, the enzymatic syntheis of CS glycopeptide **19** took a total of 14 synthetic steps from peptide **5** in 3.4% overall yield.

### Determination of the Location of Sulfations in CS Glycopeptides by Mass Spectrometry (MS)

**Figure 1.**
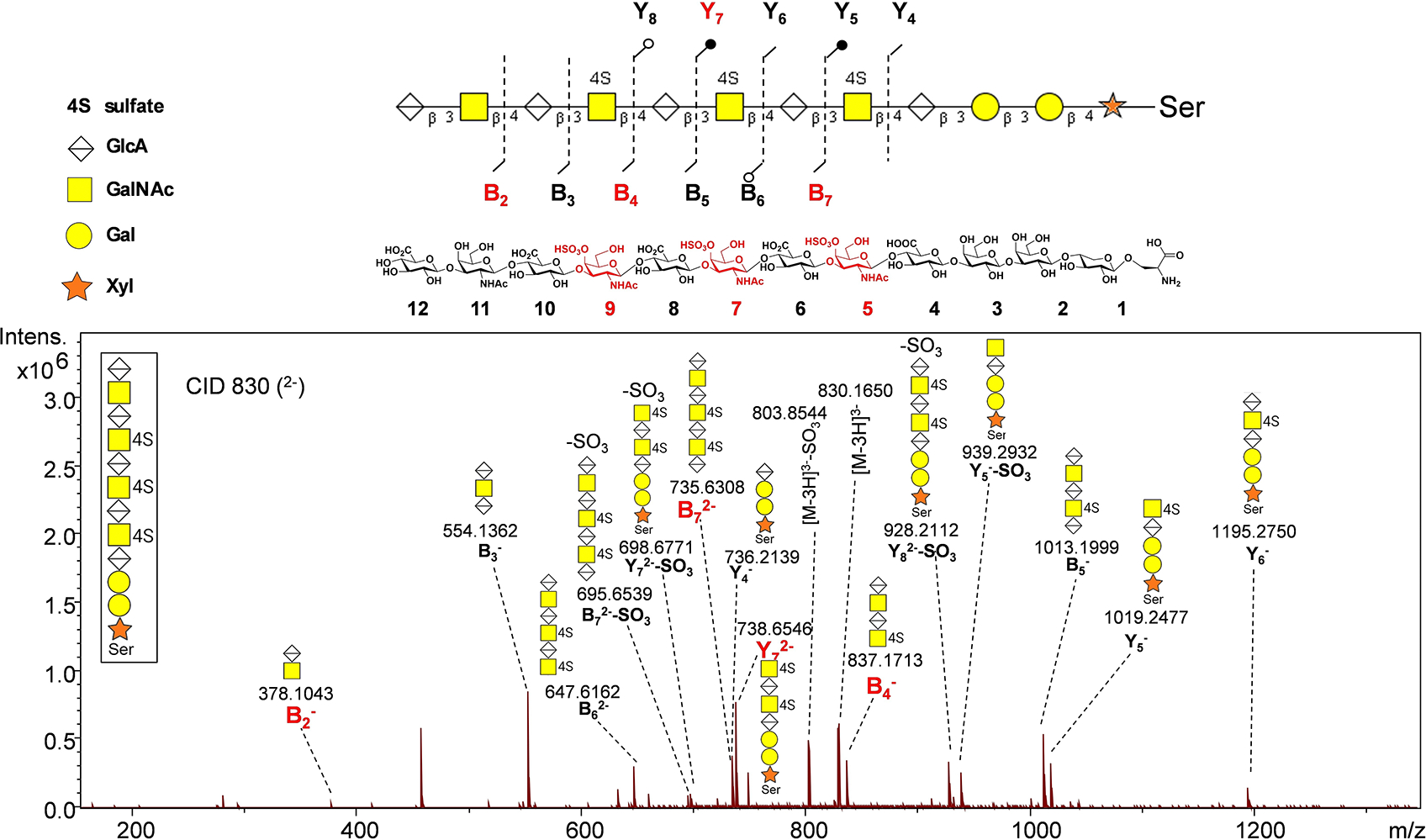
CZE-FT-ICR MS fragmentation pattern of glycopeptide **21**. The dashed lines on the structure indicate complete fragments were observed. Black filled circles on the sequence indicate both fragment ions with sulfate loss and complete fragments were observed. The empty circle indicates a fragment ion with sulfate loss was observed.

As there are multiple GalNAc residues thus potential sulfation sites within glycopeptide **19–21**, a MS based methodology was applied to determine the position(s) of sulfated GalNAc. The glycopeptides were digested with actinase E, and the resulting serine glycans were fragmented and sequenced using capillary zone electrophoresis Fourier transform ion cyclotron resonance (CZE-FT-ICR) MS. For example, for dodecasaccharide glycopeptide **21**, fragment Y_7_ reveals that the initial two sulfates are situated on GalNAc 5 and GalNAc 7 (Figure 1). The fragments B_2_ and B_7_ suggest that the GalNAc closest to the non-reducing end (GalNAc 11) was not sulfated. Thus, the third sulfated GalNAc was GalNAc-9, which was further supported by the observation of B_2_ and B_4_ fragments. These evidences collectively demonstrate a preference for sulfation on GalNAc residues toward the reducing end. The locations of sulfates on glycopeptides **19** and **20** were determined analogously (Figure S1). The lack of sulfation on the GalNAc closest to the non-reducing end is consistent with the literature,^[62]^ which is presumably because the CS4OST requires longer glycan present at the non-reducing end of the sulfation site.

### NMR-Based Conformational Analysis of Peptide and Glycopeptides 5, 10, 16, and 19

Despite the recognition of their functional importance, little is currently known about the structure features of PGs.^[63]^ As glycopeptides and glycoproteins, the conformation of the glycan chain of CSPG can be influenced by the protein core, and *vice versa.* Therefore, the integrative analysis of these components within the context of PG is of paramount importance. The access to well-defined CSPG structures provides an opportunity to determine the conformation and dynamics of these glycoconjugates.

**Figure 2.**
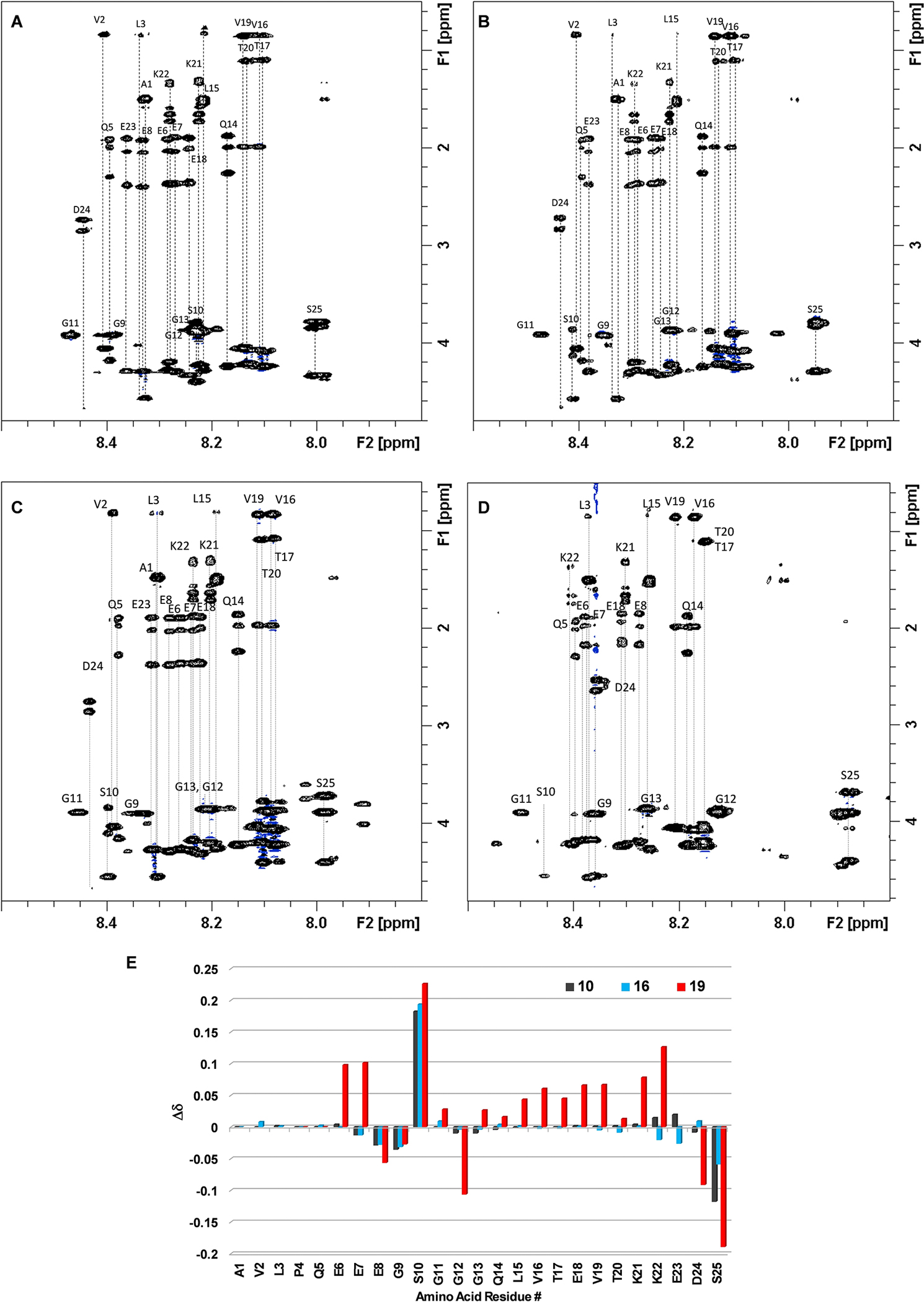
Analysis of ^1^H NMR resonances of NH groups of peptide backbone in compounds **5**, **10**, **16**, and **19**. 2D-TOCSYspectra with ^1^H resonance assignments of A) 1.54 mM **5** in H_2_O:D_2_O 90:10; B) 0.78 mM **10** in H_2_O:D_2_O 90:10; C) 0.69 mM **16** in H_2_O:D_2_O 90:10; and D) 0.2 mM **19** in H_2_O:D_2_O 90:10. E) Chemical shift perturbation observed for **10**, **16**, and **19** as compared to peptide **5** as the reference. Δδ was expressed as δ_NH(x)_-δ_NH(5)_.

A systematic analysis of the conformations of the peptide backbone in peptide **5** and glycopeptides **10**, **16**, and **19**, with increasing structural complexity, has been performed using NMR methodologies. Since the chemical shift of NH groups in the peptide backbone is especially sensitive to changes in the local environment and therefore, to changes in the secondary structure of the peptide, we compared ^1^H resonances of NH groups of peptide **5** with those of glycopeptides **10**, **16**, and **19**. 2D-TOCSY (Figure 2) and 2D-NOESY experiments were performed. All NMR measurements were conducted under identical experimental conditions including temperature and pH to rule out external factors not directly related with peptide’s structure, such as solvent effects. All compounds displayed well-dispersed NMR signals and most ^1^H resonances of the peptide backbones could be assigned. NOESY spectra showed the absence of medium- or long-range NOE effects, strongly suggesting the lack of any persistent secondary or tertiary structures of all compounds on the NMR time scale. These findings support the notion that these compounds may exist as dynamic conformational ensembles, with the average observables resembling those of flexible random coil conformations. This is consistent with similar circular dichroism (CD) spectra recorded for peptide, unsulfated glycopeptide, and sulfated glycopeptide, which were all resembling that of typical random coil (Figure S2).

As a general and distinctive feature of glycopeptides **10**, **16**, and **19**, notable distinctions were observed for the chemical shift of the backbone NH group of serine 10 (S10). In these glycopeptides, the signals from NH group of S10 were downfield shifted in comparison to those in peptide **5** (Figure 2). Minor variations were additionally detected for amino acids proximal to S10, including glutamic acid 7 (E7), glutamic acid 8 (E8) and glycine 9 (G9). Hence, backbone NHs of amino acids surrounding the glycosylation site were sensitive to the functionalization of Ser10 side chain. Slight variations were also noted for amino acids near the C-terminus peptide, particularly lysine 22 (K22), glutamic acid 23 (E23), and, to a greater extent, serine 25 (S25). Considering that S25 residue is situated farther from the glycosylation site, and no contacts with the glycan part were detected, we speculated that such variations may be indicative of subtle differences in the pKa of this residue.

Comparison of the chemical shift perturbations between the glycopeptides revealed different patterns of changes of the sulfated glycopeptide **19** *vs* the non-sulfated **10** and **16**. Besides the changes in chemical shifts around S10 common to all glycopeptides, significant variations were also detected in **19** for most of the amino acids throughout the peptide backbone of **19** (Figure 2E). These data suggest that glycan sulfation could significantly impact the overall 3D structure of glycopeptide **19**. Further investigations to decipher the detailed conformation of the sulfated glycopeptide are currently underway.

### The glycan Chain and Peptide Backbone of Bikunin CS Glycopeptide 16 Helped Enhance its Binding with Cathepsin G

With structurally defined glycopeptides in hand, we aim to better understand the structural features needed for bikunin glycopeptide binding. Cathepsin G (CatG), a neutrophil serine protease, plays a crucial role in regulating various physiological processes, including inflammation, digestion, smooth muscle contraction, and tissue remodeling.^[64]^ While CatG has been reported to interact with CSPG, the structural requirement of CSPG binding has not been established.^[25]^ To measure the binding affinity towards CatG, peptides **5** and glycopeptides **10**, **18**–**21** were biotinylated and immobilized on streptavidin coated sensors for biolayer interferometry (BLI) studies. Biotinylated 50 kDa CS and CS-A were also immobilized on the sensors for comparison. As shown in Table 1, biotinylated peptide **5**, the tetrasaccharide linkage region bearing glycopeptide **10**, and the non-sulfated chondroitin dodecasaccharide glycopeptide **18** exhibited similar dissociation constants (*K*_D_) of 400, 650, and 490 nM, respectively. Interestingly, sulfated 8-mer CS-bearing glycopeptide **19**, sulfated 10-mer CS-bearing glycopeptide **20**, and sulfated 12-mer CS-bearing glycopeptide **21** showed 10-fold lower *K*_D_ values at 49, 74, and 56 nM, indicating that sulfation on the glycopeptide could significantly enhance binding. The *K*_D_ values of commercially available CS and CS-A polymers with CatG were 180 and 220 nM, respectively. Collectively, these results demonstrate that both the sulfated glycan and the peptide backbone can participate in interaction with CatG.

### Modeling of Bikunin Glycopeptide Binding with Cathepsin G

**Figure 3.**
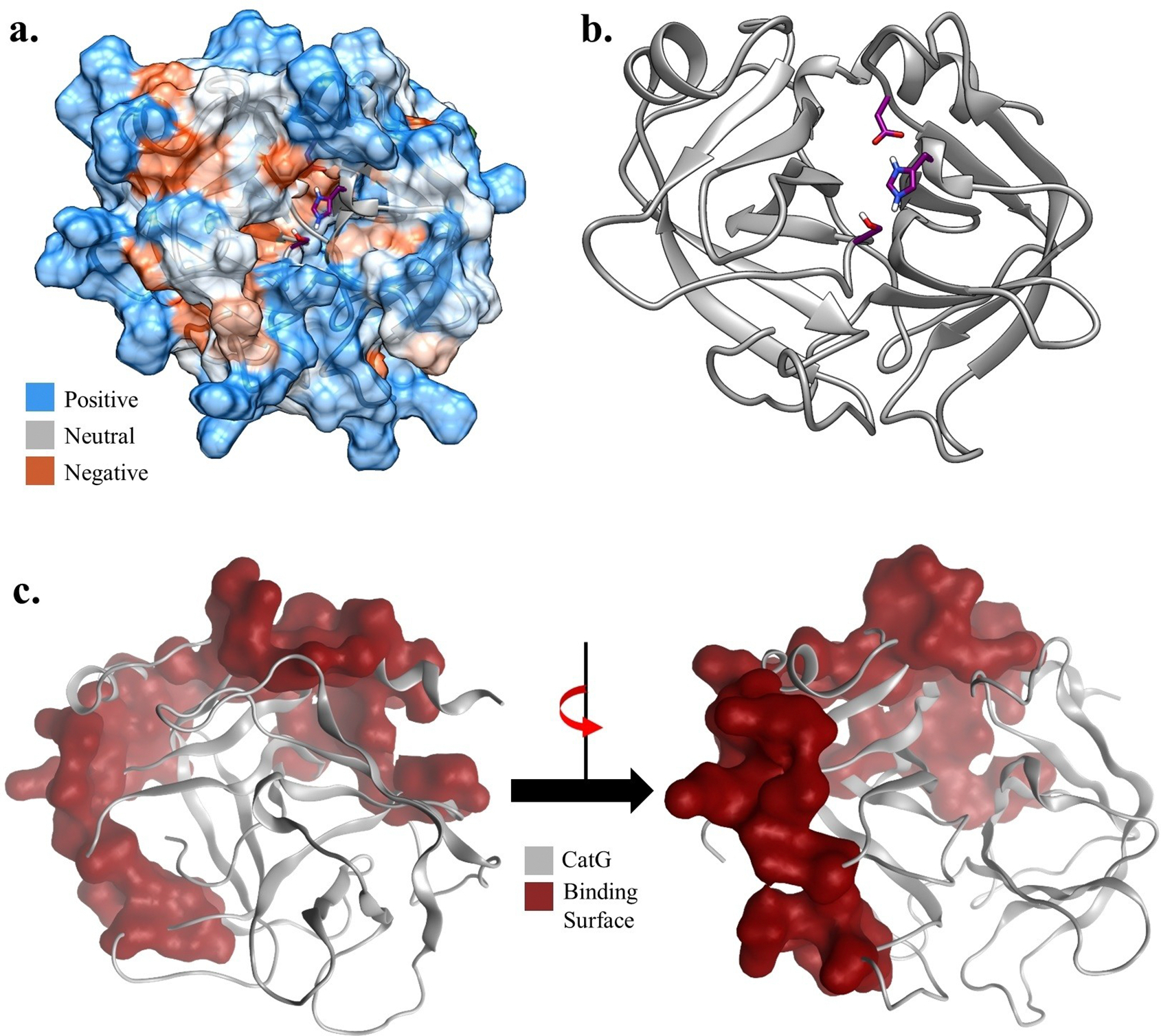
The surface and the binding surface of CatG protein. (a) Overall surface of CatG is shown in electrostatic representation with predominant positive charged residues. Blue color represents positive charge, red color indicates negative charge, and grey color represents neutral residues. (b) The catalytic residues, His57, Asp102, and Ser195 of CatG are shown in stick representation. (c) The selected region surface of CatG protein for glycopeptide binding. The numbering of the residues follows the 1T32 PDB numbering.

To better understand how sulfates can enhance the binding with CatG, a molecular docking approach was utilized. We prepared the structures of peptide **5**, non-sulfated dodecasaccharide glycopeptide **18** and three sulfated glycopeptides **19**–**21** and CatG (PDB: 1T32) using CHARMM GUI and Molecular Operating Environment (MOE) at pH=7 and 300 K.^[65–69]^ In addition, induced fit docking was considered for all obtained poses. CatG has a highly charged surface with multiple arginine residues, and its catalytic residues are buried within a cavity (Figures 3a,b). Based on docking poses, the region shown in Figure 3c has been identified as the potential binding site for the peptide and the glycopeptides.

**Figure 4.**
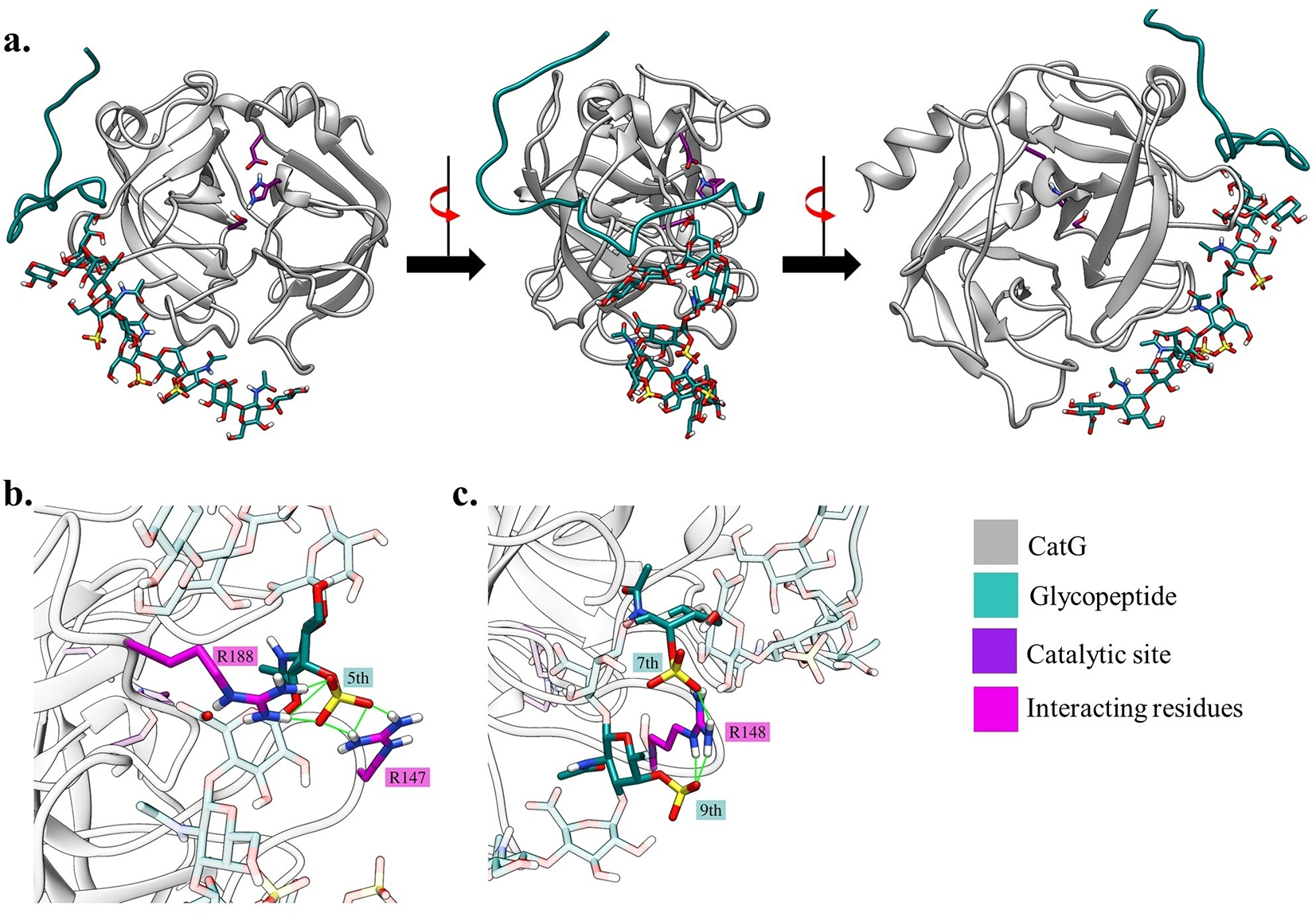
(a) The highest scoring pose of **21** with CatG. (b) The direct hydrogen bond interactions of the *O*-sulfate on GalNAc 5 are shown by the green lines. (c) The direct hydrogen bond interactions of the *O*-sulfates on GalNAc 7 and 9 are shown by the green lines. The numberings of the residues follow the 1T32 PDB numbering.

The docking results indicate that the bindings between CatG and the peptide occur mainly through the interactions formed with surface arginine and lysine residues of CatG and the negatively charged glutamic acid residues E8 and E18 of the peptide (Figure S4a). In addition, albeit weaker, there are interactions between the side chains or the backbone of CatG and the backbone of the peptide. The docking scores (Table S1) of the peptide and glycopeptides suggest that the presence of *O*-sulfate on glycopeptides led to lower docking scores and stronger binding, consistent with the dissociation constants determined experimentally (Table 1). The key interactions that lead to the stronger affinity are mainly between surface arginine residues of CatG protein and sulfate groups from the glycopeptides. For instance, all three sulfates of glycopeptide **21** were observed to directly interact with residues from CatG through hydrogen bonding, as shown in Figure 4. The *O*-sulfate on GalNAc 5 residue interacts with side chains of R147 and R188 residues, and similarly, the *O*-sulfates on GalNAc 7 and 9 residues form hydrogen bonds with the R148 residue (Figure 4). In the case of octasaccharide CSPG **19**, its *O*-sulfate forms hydrogen bonds with R185 and R186 (Figure S4b). For the decasaccharide CSPG **20**, the *O*-sulfate on GalNAc 5 is in proximity to R125 although there is no direct interaction observed (Figure S5b) and its *O*-sulfate on GalNAc 7 forms direct hydrogen bonds with R239 residue (Figure S5c). For the unsulfated glycopeptide **18**, the surface arginine residues of CatG did not form hydrogen bonds with the glycopeptide. These observations highlight the importance of sulfate groups on glycopeptides for stronger affinity towards the CatG protein by enabling the direct hydrogen bond interactions with surface arginine residues. It should be pointed out that the influence of the sulfate on the binding of glycoprotein beyond the glycopeptide to CatG remains to be established, as glycoprotein may be more conformationally rigid than glycopeptide.

## Conclusion

Chemoenzymatic synthesis of CSPG glycopeptides has been successfully developed for the first time. This approach comprises several key elements. The Sepharose beads were utilized as the solid phase support for multi-step enzymatic reactions and proved robust under the enzymatic reaction toward CSPG and hydrazine mediated cleavage conditions. The diethyl squarate was selected as a traceless linker, which is compatible with the CS glycopeptide, yielding the native glycopeptide as the final product after cleavage from Sepharose. The GAG-initiating transferase XT-1 transferred the first saccharide, xylose, to the peptide substrate, obviating the need to synthesize the glycosylated amino acid module for glycopeptide formation. To the best of our knowledge, this represents the first example of direct glycosylation of peptides rather than using a glycopeptide as an enzymatic substrate to initiate the synthesis on solid phase. All requisite enzymes have been produced and the necessary reaction sequence has been identified enabling the synthesis of PGs. With this approach, we have successfully synthesized five distinct tetrasaccharide linkage region-bearing glycopeptides, varying in peptide length, number of glycosylation sites, and polarity of amino acid residues flanking the glycosylation sites. Furthermore, this method is efficacious in producing CS-bearing glycopeptides including the longest homogeneous GAG-bearing glycopeptide synthesized to date. Compared to chemical synthesis, this new chemoenzymatic strategy can reduce the total number of synthetic steps required by more than 80%.

The availability of the various well-defined synthetic bikunin glycopeptides enabled the conformation studies of the glycopeptides by NMR, which suggest that glycan sulfation could significantly impact the overall 3D structure of glycopeptide **19**. Furthermore, binding study with CatG was performed. The presence of sulfate on the glycopeptide significantly enhances its affinity towards CatG, implying that CatG may potentially interact with bikunin in vivo. Docking studies provide further insights into the interactions between sulfates and residues on CatG, shedding light on the mechanism by which bikunin may engage with its respective protein partner. Therefore, the efficient chemoenzymatic strategy developed opens new avenues to synthesize and investigate the biological functions of PGs.

## Supplementary Material

Supporting info

## Figures and Tables

**Table 1:** BLI experiments determined dissociation constants of biotinylated compounds **5**, **10**, **18–21**, 50 kDa CS and 50 kDa CS-A.

Compound	K_D_ (nM) with Cathepsin G

**5**	400 ± 40
**10**	650 ± 80
**18**	490 ± 18
**19**	49 ± 5
**20**	74 ± 7
**21**	56 ± 8
50 kDa CS	180 ± 10
50 kDa CS-A	220 ± 20

## Data Availability

The data that support the findings of this study are available in the supplementary material of this article.
