# Supplementary material for: Solid-Phase-Supported Chemoenzymatic Synthesis and Analysis of Chondroitin Sulfate Proteoglycan Glycopeptides
Source: Angew Chem Int Ed Engl. Author manuscript; Available in PMC 2025 Jan 28. (PMC11772155; doi:10.1002/anie.202405671)

Supporting Information

Solid-Phase-Supported Chemoenzymatic Synthesis and Analysis of Chondroitin Sulfate Proteoglycan Glycopeptides

Po-han Lin,[1,2] Yongmei Xu,[3] Semiha Kevser Bali,[1] Jandi Kim,[4,] Ana Gimeno,[5,6] Elijah T. Roberts,[4] Deepak James,[1] Nuno M. S. Almeida,[1] Narasimhan Loganathan,[1] Fei Fan,[1,2] Angela K. Wilson,[1] I. Jonathan Amster,[4] Kelley W. Moremen,[7,8] Jian Liu,[3] Jesús Jiménez-Barbero,[5,6,9,10] and Xuefei Huang[1,2,11*]

[1]Department of Chemistry, [2]Institute for Quantitative Health Science and Engineering, Michigan State University, East Lansing, Michigan 48824, United States

[3]Division of Chemical Biology and Medicinal Chemistry, Eshelman School of Pharmacy, University of North Carolina, Chapel Hill, North Carolina 27599, United States

[4]Department of Chemistry, University of Georgia, Athens, GA 30602, United States

[5]Chemical Glycobiology Lab, Center for Cooperative Research in Biosciences (CICbioGUNE), Basque Research and Technology Alliance (BRTA), 48160 Derio, Bizkaia, Spain

[6]Ikerbasque, Basque Foundation for Science, Bilbao 48009, Spain

[7]Department of Biochemistry & Molecular Biology, University of Georgia, Athens, GA 30602, United States

[8]Complex Carbohydrate Research Center, University of Georgia, Athens, GA 30602, United States

[9]Department of Inorganic & Organic Chemistry, Faculty of Science and Technology, University of the Basque Country, EHU-UPV, Leioa 48940, Spain

[10]Centro de Investigación Biomédica en Red de Enfermedades Respiratorias, Madrid 28029, Spain

[11]Department of Biomedical Engineering, Michigan State University, East Lansing, Michigan 48824, United States

Email: huangxu2@msu.edu

Table of Contents

Materials and methods	S3
General information	S3
General procedure of peptide conjugation to EAH Sepharose	S3
Enzyme expression, purification and characterization	S4
General procedure of enzymatic glycosylation on peptide-conjugated Sepharose	S4
CZE-FT-ICR MS analysis	S6
General procedure for NMR analysis	S7
General procedure of glycopeptide biotinylation	S7
General procedure for BLI binding assay	S7
Docking methods	S8
Synthetic procedures and product characterization data	S9
Supporting Figures	S28-S32
Supporting **Table S1**	S33
References	S33
NMR spectra, HPLC and MS	S34-S109

Materials and methods

Materials. Plasmids for XT-1 and B4GALT7 were previously documented,[1-2] while FAM20B and B3GALT6 plasmids were graciously provided by Dr. Jack Dixon and Dr. Kelley Moremen. The XYLP and B3GAT3 plasmids were constructed following established literature methods.[3-4] Enzymes and substrates, including KfoC, CS4OST, UDP-GalNAc, and PAPS, were generously gifted by Dr. Jian Liu. The Expi 293 Expression system, along with Coomassie Brilliant Blue G-250, DTT, and EAH Sepharose, were purchased from Thermo Fischer Scientific (Waltham, MA). Nickel columns and Nickel resins, SDS-PAGE gels, 10x Tris/Glycine/SDS electrophoresis buffer, pre-stained protein ladder, sample loading buffer, and Coomassie Blue R-250 were obtained from Bio-rad (Hercules, California). Shrimp alkaline phosphatase (rSAP) was acquired from NEB (Ipswich, MA). Diethyl squarate, UDP-galactose, UDP-glucuronic acid, and ATP were sourced from Sigma Aldrich (St. Louis, MO). UDP-xylose was purchased from the Complex Carbohydrate Research Center (Athens, Georgia). The peptides were synthesized by Synpeptide (China), and syringes with frit were procured from Torviq (Tucson, AZ). The 50 kDa CS and 50 kDa CS-A were purchased from HAworks (Bedminster, NJ). Human Neutrophil Cathepsin G was purchased from Athens Research & Technology, Inc. (Athens, Georgia). All other chemicals were purchased from commercial sources and used without additional purifications unless otherwise noted.

General Information. High-performance liquid chromatography was carried out with two systems: LC-8A Solvent Pumps, DGU-14A Degasser, SPD-10A UV-Vis Detector, SCL-10A System Controller (Shimadzu Corporation, JP); G7111B 1260 quat pump, G7129A 1260 vial sampler, G7114A 1260 VWD, G1364F 1260 FC-AS, G1328C 1260 manual injetor (Agilent Technologies, CA). The columns utilized included: Vydac 218TP 10 μm C18 Preparative HPLC column (HICHROM Limited, VWR, UK) and 20RBAX 300SB-C18 Analytical HPLC column (Agilent Technologies, CA) using HPLC-grade acetonitrile (EMD Millipore Corporation, MA) and Milli-Q water (EMD Millipore Corporation, MA). A variety of eluting gradients were set up on LabSolutions software (Shimadzu Corporation, JP) and Agilent Open lab control panel (Agilent Technologies, CA). The dual wavelength UV detector was set at 220 nm and 254 nm for monitoring the absorbance from the amide and aromatic region. NMR data were acquired with Bruker 600 and 800 MHz NMR (Bruker, MA) at ambient temperature.

General procedure of peptide conjugation to EAH Sepharose

A solution of 10 mg of peptide in carbonate buffer (25 μL, 0.1 M, pH 8) was added to a 1.5 mL Eppendorf. Diethyl squarate (3 equiv.) was diluted with MeOH (25 μL) and then added to the peptide mixture. The pH value of the mixture was adjusted to 8 and incubated for 6h at RT until no starting material was observed by LCMS. Upon completion, mixtures were lyophilized, and the resulting white solid was dissolved in carbonate buffer (1 mL, 0.1 M, pH 8). EAH Sepharose (9 μmol amine per mL of drained Sepharose, 2 equiv. of peptide) was washed with water (20 mL) twice, carbonate buffer (20 mL, 0.1 M, pH 8) once and transferred to a syringe (10 mL) with frit. The peptide solution in carbonate buffer was added to the syringe and agitated with end-to-end rotation for 1 day at room temperature (RT) until no squarate conjugated peptide was observed in the supernatant by LCMS.

Expression, purification and characterization of β3GAT3.

β3GAT3-expressing BL21 competent cells were cultured onto a kanamycin containing petri dish, which was incubated at 37 °C overnight. One colony of the BL21 cells was picked and inoculated into 10 mL Luria-Bertani (LB) starter culture containing kanamycin (50 μg/ml). The cell culture was incubated at 37 °C overnight. The starter culture was then transferred into autoclaved 1L LB medium (with 30 mg/L kanamycin) and incubated at 37 °C with shaking at 250 rpm. When the OD600 reached 1.0, IPTG (1 mM) was added to induce protein expression at 23 °C for 16 hours. Cells were centrifuged at 4 °C, 10,000 g for 10 min. Cell pellet was lysed using 1X Cellytic in buffer (10 mL), 50 U/mL benzonase, 0.2 mg/mL lysozyme and 1 tablet of cOmplete™ Protease Inhibitor Cocktail EDTA-free for 20 min at ambient temperature. Clarified lysate was purified by a nickel column (a. washing buffer: 20 mM phosphate, 0.5 M NaCl and 40 mM imidazole; b. eluting buffer: 20 mM phosphate, 0.5 M NaCl and 40-250 mM imidazole). Protein purity was confirmed with SDS-PAGE gel electrophoresis (**Figure S6**) and the expression yield of 5 mg/L was determined by the standard Bradford assay.

Expression, purification and characterization of FAM20B, B3GALT6 and XYLP.

Expi293F cells were grown in FreeStyleTM 293 Expression Medium on a platform shaker in humidified 37 °C CO_2 (8%) incubator with rotation at 150 rpm. When the cell density reached between 4 x $10^5$and 3 x 10^6 cells/ml, cells were split to a density of 1.5 x 10^6 cells/ml and cultured 1 day with fresh medium. The desired plasmid (1 μg plasmid per ml medium) was diluted with Opti-MEM I Reduced-Serum Medium then mixed with ExpiFectamine™ 293 following the manufacturing protocol. This mixture was incubated for 15 min at RT then added dropwise into the cells. At this point, cell density should be around 3 x 10^6 cells/ml. The flask was returned to the shaker platform in the incubator. After 1 day, transfection enhancer was added. Six days after the transfection, medium was harvested. Clarified medium was purified by nickel column (a. washing buffer: 20 mM Tris, 0.5 M NaCl and 40 mM imidazole; b. eluting buffer: 20 mM Tris, 0.5 M NaCl and 250 mM imidazole). Protein purity was confirmed with SDS-PAGE gel electrophoresis (**Figure S6**) and the expression yields of XT-1, FAM20B and β3GALT6 were determined to be 10, 2.3 and 16.7 mg/L respectively by standard Bradford assay.

General procedure of enzymatic glycosylation on peptide-conjugated Sepharose:

Step 1:

Peptide conjugated Sepharose (1 mL, 50% loading) was drained and then resuspended in 4 mL of XT-1 reaction buffer. This buffer contained the following components: 25 mM MES, 25 mM KCl, 5 mM KF, 5 mM $MgCl_2$, 5 mM $MnCl_2$, pH 6.5. To this resuspended mixture, XT-1 (100 μg) and UDP-xylose (2 equiv. relative to the number of reactive sites) were added. The resulting reaction mixture was agitated with end-to-end rotation at 4 ºC for a duration of 12 h. It is important to perform the reaction at 4 ºC as higher reaction temperature tends to lead to precipitation of the enzyme during the agitated reaction.

Subsequently, the mixture was filtered, followed by two washes with a total volume of 20 mL of water each time. This entire process was then repeated one time.

Step 2:

The drained xylosylated peptide conjugated Sepharose from step 1 was resuspended in 4 mL of a B4GALT7 reaction buffer. This buffer consisted of 20 mM MES and 10 mM $MnCl_2$, pH 6.2. To this resuspended mixture, B4GALT7 (250 μg) and UDP-Gal (2 equiv. per SG sites) were added. The resulting reaction mixture was agitated with end-to-end rotation at 4 °C for 12 h. Subsequently, the mixture was subjected to filtration, followed by two washes with a total volume of 20 mL of water each time. This entire process was then repeated.

Step 3:

Drained disaccharide glycopeptide conjugated Sepharose from step 2 (1 mL, 50% loading) was resuspended in 4 mL of a FAM20B reaction buffer. This buffer solution consisted of 50 mM HEPES and 10 mM $MnCl_2$, pH 7.4. To this resuspended mixture, FAM20B (200 μg) and ATP (3 equiv. per SG sites) were added. The resulting reaction mixture was subjected to end-to-end rotation at 4 °C for a duration of 12 h. Subsequently, the mixture was filtered, followed by two washes with a total volume of 20 mL of water each time. This entire process was then repeated.

Step 4:

Drained phosphorylated disaccharide glycopeptide conjugated Sepharose (1 mL, 50% loading) from step 3 was resuspended using 4 mL of a B3GALT6 reaction buffer. This buffer solution consisted of 50 mM MES, 10 mM $MnCl_2$, 100 mM NaCl, pH 6.0. To this resuspended mixture, B3GALT6 (200 μg) and UDP-Gal (0.6 equiv. per SG sites) were added. The resulting reaction mixture was mixed with end-to-end rotation at 4 °C for a duration of 12 h. Subsequently, the mixture was filtered, followed by two washes with a total volume of 20 mL of water each time. This entire process was then repeated.

Step 5:

Drained phosphorylated trisaccharide glycopeptide conjugated Sepharose (1 mL, 50% loading) from step 4, was resuspended in 4 mL of a XYLP reaction buffer. This buffer solution consisted of 50 mM Tris-HCl, pH 5.8. To this reaction mixture, XYLP (200 μg) was added. The resulting reaction mixture was subjected to end-to-end rotation at 4 °C for a duration of 12 h. Subsequently, the mixture was filtered, followed by two washes with a total volume of 20 mL of water each time. This entire process was then repeated.

Step 6:

Drained trisaccharide glycopeptide conjugated Sepharose (1 mL, 50% loading) from step 5, was resuspended in 4 mL of a B3GAT3 reaction buffer. This buffer solution consisted of 50 mM MES, 2 mM $MnCl_2$, pH 6.5. To this mixture, B3GAT3 (500 μg) and UDP-GlcA (2 equiv. per SG site) were added. The resulting reaction mixture was subjected to end-to-end rotation at 4 °C for 12 h. Subsequently, the mixture was filtered, followed by two washes with a total volume of 20 mL of water each time. This entire process was then repeated.

Step 7:

Drained tetrasaccharide glycopeptide conjugated Sepharose (1 mL, 50% loading) from step 6, was resuspended in 4 mL of a KfoC reaction buffer. This buffer solution consisted of 50 mM MOPS, 15 mM $MnCl_2$, pH 7.2. To this resuspended mixture, KfoC (100 μg) and UDP-GalNAc (12.5 mM) were added. The resulting reaction mixture was subjected to end-to-end rotation at 4 ºC for 12 h. Subsequently, the mixture was filtered, followed by two washes with a total volume of 20 mL of water each time. This entire process was then repeated.

Step 8:

Drained pentasaccharide glycopeptide conjugated Sepharose (1 mL, 50% loading) from step 7, was resuspended in 4 mL of a KfoC reaction buffer. This buffer solution consisted of 50 mM MOPS, 15 mM $MnCl_2$, pH 7.2. To this resuspended mixture, KfoC (100 μg) and UDP-GlcA (2 equiv. per SG sites) were added. The resulting reaction mixture was subjected to end-to-end rotation at 4 ºC for a duration of 12 h. Subsequently, the mixture was subjected to filtration, followed by two washes with a total volume of 20 mL of water each time. This entire process was then repeated.

Step 9:

Drained CS backbone-bearing glycopeptide conjugated Sepharose (1 mL, 50% loading) from step 8 was resuspended in 4 mL of a CS4OST reaction buffer. This buffer solution consisted of 50mM MOPS, 10mM $CaCl_2$, fresh 2 mM DDT, pH 6.5. To this resuspended mixture, CS4OST (400 μg) and PAPS (2 equiv. per SG sites) were added. The resulting reaction mixture was subjected to end-to-end rotation at 4 ºC for 12 h. Subsequently, the mixture was filtered, followed by two washes with a total volume of 20 mL of water each time. This entire process was then repeated.

CZE-FT-ICR MS Analysis

Functionalized capillary was prepared through dichlorodimethylsilane (DMS) functionalization and HF etching procedures as reported previously.[5] Briefly, a 130 cm long functionalized capillary was segmented into two equal length pieces to ensure the uniformity of the internal derivatization. The final 10 mm of the outlet end of each capillary was etched with hydrofluoric acid (HF) to a conical shape with an outer diameter at the terminus of < 100 μm to reduce the mixing volume for analytes entering the sheath flow interface.

Capillary zone electrophoresis (CZE) was performed using a CMP ECE-001 CZE system (CMP scientific, Brooklyn, NY). The CZE was interfaced to the mass spectrometer with an electrokinetically pumped sheath flow CE-MS interface (EMASS-II interface, CMP Scientific). Mass spectra were collected in negative mode on Bruker 9.4 T SolariX FT-ICR mass spectrometer (Bruker Daltonics, Bremen, Germany). Mass spectra were collected between m/z 150 – 3000 with 1M data points and a 0.5592 s transient. Ion accumulation time was set to 0.3 s and the time of flight (TOF) was set to 0.8 ms. The flow rate of the drying gas was set to 2 L/min at 180 ºC. The inlet capillary voltage of the FT-ICR was set to 0 V.

CZE separations were performed using fused silica capillaries (60 cm x 360 μm OD x 50

μm ID) functionalized with dichlorodimethylsilane (DMS) neutral coated capillary. DMS functionalization and HF etching procedures have been reported previously.[5] A 130 cm long functionalized capillary was segmented into two equal length pieces to ensure the uniformity of the internal derivatization. The final 10 mm of the outlet end of each capillary was etched with hydrofluoric acid to a conical shape with an outer diameter at the terminus of < 100 μm to reduce the mixing volume for analytes entering the sheath flow interface.

Ammonium formate solution (25 mM) in 70% (v/v) methanol/water was used as a sheath liquid (SL) and a background electrolyte (BGE). The etched ends of both functionalized capillaries were positioned 0.5 mm from the tip of a borosilicate glass emitter orifice (0.75 mm ID, 5.0 cm length and 20 μm opening diameter of tip). The distance between the emitter opening and the inlet of MS was ca. 2.5 mm. The potential difference between the spray tip and the entrance to the mass spectrometer ESI inlet was -2.0 kV voltage. Each sample was injected into a CZE capillary using a pressure of 400 mbar for 10 s, resulting in circa 115 nL volume and 10.6% of the total capillary volume. The capillary was completely rinsed with fresh BGE after each run to remove residual carryover for the next run. In terms of the auto MS/MS mode (CID), a preferential and exclusion list was implemented for auto CID. MS1 scan was performed first followed by three MS/MS scans under the external ion accumulation time of 0.5 s. The collision voltage was fixed between 13 and 15 V for each mass spectrum.

Mass spectra were analyzed using Compass Data Analysis v4.1 software (Bruker Scientific, Bremen, Germany), in-house software developed in MATLAB (The MathWorks, Natick, MA) as well as Glycoworkbench to annotate fragment ions.[6]

General procedure for NMR analysis.

NMR experiments described in **Figure 2** were performed at 25 ºC on a Bruker 800 MHz AVANCE III spectrometer equipped with a cryoprobe. The ^{1}H-NMR resonances of the compounds were assigned through standard TOCSY (30 and 80 ms mixing times), NOESY (100-300 ms mixing times), and HSQC experiments. 500 μL samples were prepared by dissolving the purified compound in D_2O or H_2O : D_2O 90:10.

General procedure of glycopeptide biotinylation

To a solution of peptide or glycopeptide in DMSO/H_2O (1/1, 0.1 ml) was added NHS-LC-Biotin (4 equiv.) and diisoproylethylamine (pH ~ 8.5). The reaction was incubated at 37 ºC for 2 h. Then the mixture was dried *in vacuo* and purified by HPLC.

General procedure for BLI binding assay

The binding assay was performed on the Octet K2 System (Pall ForteBio). The biotinylated compounds were incubated with streptavidin (SA) sensors for 2 min. The sensor was then balanced in the assay buffer (PBS containing 0.005% P20) and dipped into Cathepsin G solution in assay buffer at 2000 nM, 1000 nM, 500 nM, 250 nM concentrations. After 5 min of association, the sensor was brought back to the previous assay buffer for a 5 min dissociation step. At the end of the assay, the sensor was regenerated in 2 M NaCl to remove the bound protein. Each measurement was repeated 3 times on the same sensor. The control assay was performed with another sensor loaded with a 2 mM biotin solution.

Docking methods

The protein structure of Cathepsin G (PDB ID: 1T32, Res.: 1.85 Å) was prepared using Molecular Operating Environment (MOE 2022.02) with structure preparation module. The pronation states of the titratable residues were determined using PropKa at pH 7. The glycopeptides were prepared on CharmmGUI, with ff14SB and GLYCAM forcefields for peptide and glycan, respectively. The glycopeptide structures were minimized in MOE and used for docking.

MOE is used for the docking procedure, which involves two different approaches. First, all CatG surface was selected as a potential binding site for the glycopeptides, and rigid docking was performed with GBVA/WSA scoring function, reporting 10 poses. This process was repeated three times, resulting in 30 accumulated poses for glycopeptide. Region is determined by the Site Finder algorithm of MOE by only selecting predicted site with lowest hydrophobicity score. For the highest scoring poses, the pose is minimized together with CatG protein and rescored.

Synthetic procedures and product characterization data

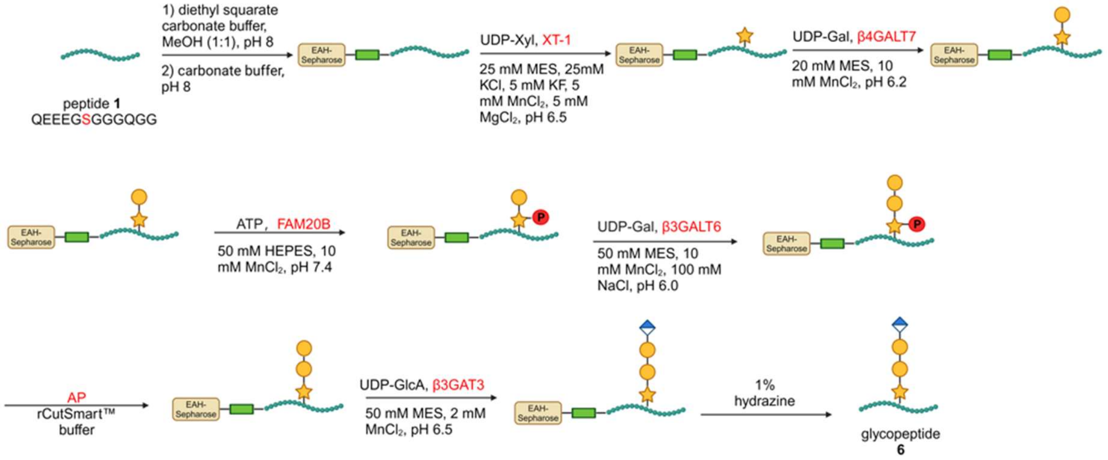

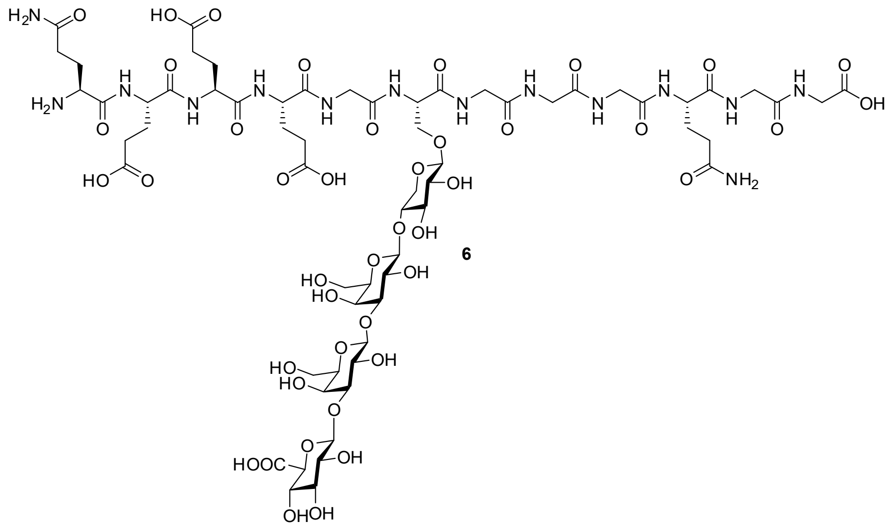

6

Peptide **1** (10 mg, 9.1 μmol) was conjugated to EAH Sepharose (2 mL, drained volume) following the general procedure of peptide conjugation to EAH Sepharose. The resulting Sepharose was resuspended in buffer following steps 1 to 6 of the general procedure of enzymatic glycosylation on peptide-conjugated Sepharose. Crude products were obtained after incubating glycosylated Sepharose with 1% hydrazine (10 mL) three times, 12 h each. The solution containing the glycopeptide was dried *in vacuo* and purified by prep C-18 HPLC (water/acetonitrile; 0.1% trifluoroacetic acid, 0% [0 min]-10% [15 min], 6min/15 min) to obtain a white amorphous solid compound **9** (1.5 mg) in 9.4% yield. ^{1}H NMR (800 MHz, D_2O) δ 4.66 – 4.60 (m, 3H), 4.46 (d, J = 7.9 Hz, 1H), 4.40 – 4.31 (m, 6H), 4.19 – 4.15 (m, 1H), 4.14 – 4.12 (m, 2H), 4.06 – 4.02 (m, 2H), 3.98 – 3.89 (m, 11H), 3.85 (d, J = 10.2 Hz, 2H), 3.80 – 3.60 (m, 11H), 3.55 (t, J = 9.0 Hz, 1H), 3.50 – 3.45 (m, 2H), 3.39 – 3.32 (m, 2H), 3.26 (t, J = 8.6 Hz, 1H), 2.52 – 2.34 (m, 9H), 2.33 (t, J = 7.9 Hz, 1H), 2.16 – 2.00 (m, 6H), 2.00 – 1.90 (m, 4H); ^{13}C NMR (201 MHz, D_2O) δ 182.23, 177.82, 177.22, 177.07 176.76,

175.27, 174.73, 173.75, 173.72, 173.18, 173.10, 173.00, 172.04, 172.01, 171.98, 171.59, 171.46, 171.33, 171.30, 169.25, 169.09, 103.88, 103.55, 102.79, 101.28, 82.29, 82.26, 81.91, 76.27, 75.85, 75.14, 74.88, 74.70, 73.66, 72.98, 72.64, 71.57, 70.04, 69.73, 68.63, 68.36, 67.97, 62.92, 60.99, 60.85, 56.67, 53.62, 53.14, 53.09, 53.03, 52.90, 52.16, 42.65, 42.42, 42.36, 42.33, 30.96, 30.13, 30.05, 29.85, 26.51, 26.36, 26.26, 26.18, 26.15, 26.08, 25.96. HRMS (ESI) m/z: $[M + 2H]^{2+}$ Calcd for Chemical Formula: $C_{63}H_{100}N_{14}O_{42}$ 862.3051; Found 862.3098.

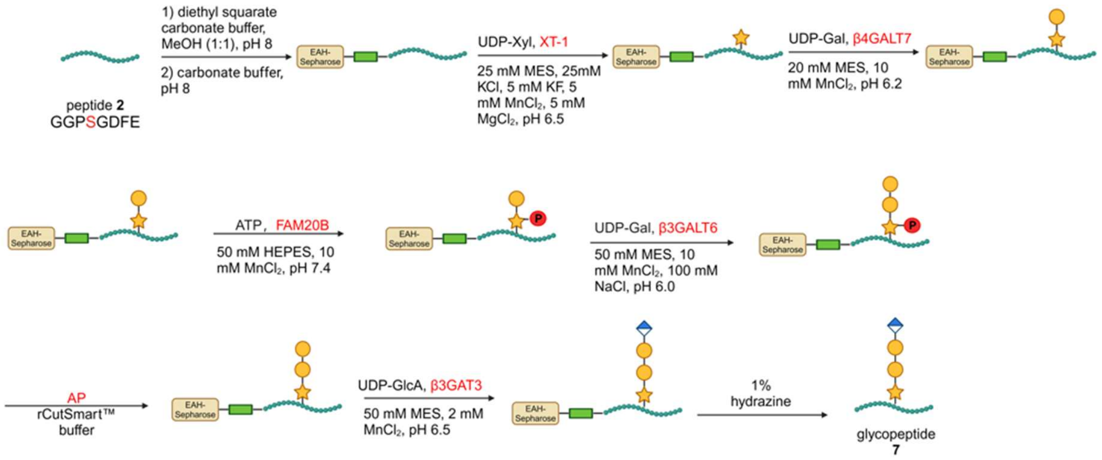

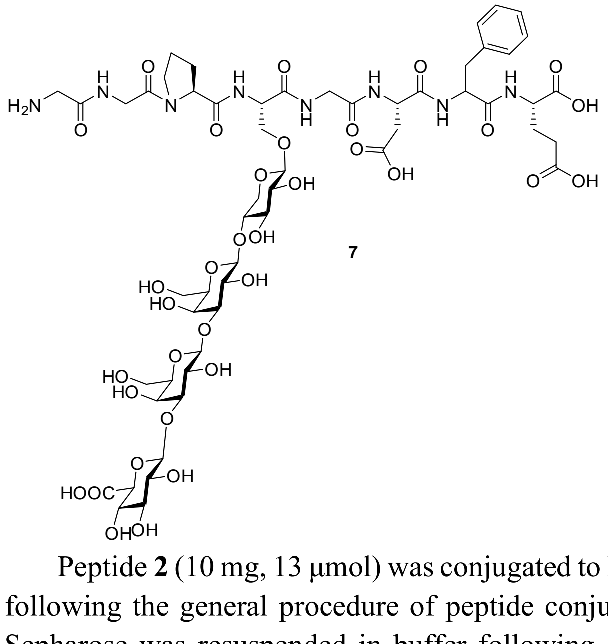

7

Peptide **2** (10 mg, 13 μmol) was conjugated to EAH Sepharose (2.9 mL, drained volume) following the general procedure of peptide conjugation to EAH Sepharose. The resulting Sepharose was resuspended in buffer following steps 1 to 6 of the general procedure of enzymatic glycosylation on peptide-conjugated Sepharose. Crude products were obtained after incubating glycosylated Sepharose with 1% hydrazine (10 mL) three times, 12 h each. The solution containing the glycopeptide was dried *in vacuo* and purified by prep C-18 HPLC (water/acetonitrile; 0.1% trifluoroacetic acid, 0% [0 min]-30% [30 min], 17min/30 min) to

obtain a white amorphous solid compound **6** (980 μg) in 5.4% overall yield from peptide **1**. ^{1}H NMR (800 MHz, D_2O) δ 7.28 – 7.15 (m, 5H), 4.63 – 4.52 (m, 2H), 4.51 – 4.45 (m, 1H), 4.43 – 4.32 (m, 3H), 4.15 – 4.07 (m, 4H), 4.05 – 4.02 (m, 1H), 4.01-3.97 (m, 1H), 3.93 – 3.83 (m, 2H), 3.87 – 3.76 (m, 3H), 3.75 – 3.49 (m, 15H), 3.44 – 3.40 (m, 2H), 3.35 – 3.26 (m, 2H), 3.26 – 3.20 (m, 1H), 3.12 (dd, J = 13.8, 5.3 Hz, 1H), 2.92 (dd, J = 13.8, 5.3 Hz, 1H), 2.52 (dd, J = 16.0, 8.7 Hz, 1H), 2.37 (dd, J = 16.0, 8.7 Hz, 1H), 2.24 – 2.19 (m, 1H), 2.13 (t, J = 7.2 Hz, 2H), 2.00 – 1.86 (m, 4H), 1.82-1.75 (m, 1H); ^{13}C NMR (201 MHz, D_2O) δ 180.56, 177.77, 177.12, 175.96, 174.56, 172.80, 172.00, 171.34, 170.62, 169.46, 167.49, 164.98, 136.48, 129.28, 128.66, 127.05, 103.91, 103.57, 102.95, 101.33, 82.40, 81.97, 76.34, 76.18, 75.26, 74.89, 74.75, 73.72, 73.10, 72.68, 71.73, 70.10, 69.78, 68.60, 68.41, 67.97, 62.94, 61.01, 60.92, 54.75, 53.49, 51.37, 47.06, 42.44, 41.74, 40.35, 38.02, 36.87, 32.70, 29.38, 27.99, 24.43. HRMS (ESI) m/z: $[M + H]^+$ Calcd for Chemical Formula: $C_{55}H_{81}N_8O_{34}$ 1397.4847; Found 1397.4796.

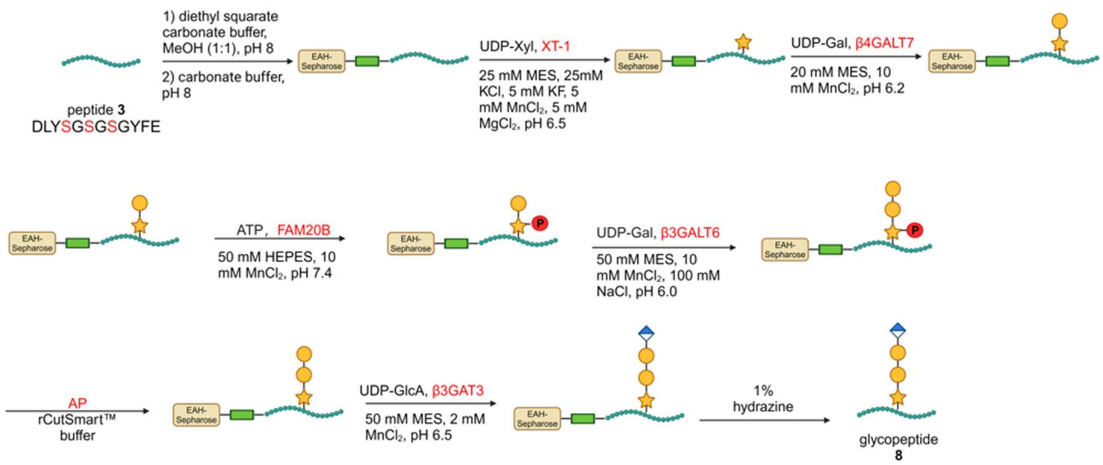

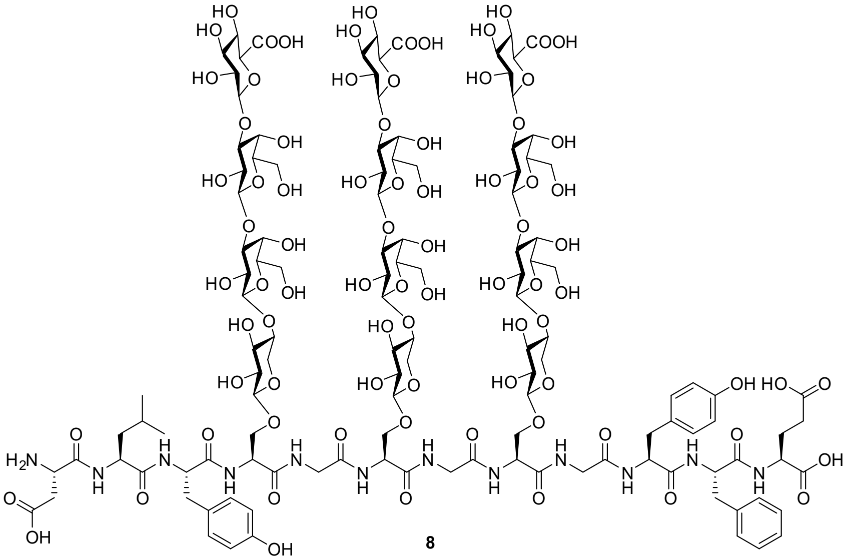

8

Peptide **3** (10 mg, 7.8 μmol) was conjugated to EAH Sepharose (1.73 mL, drained volume) following the general procedure of peptide conjugation to EAH Sepharose. The resulting Sepharose was resuspended in buffer following steps 1 to 6 of the general procedures of enzymatic glycosylation on peptide-conjugated Sepharose. Crude products were obtained after incubating glycosylated Sepharose with 1% hydrazine (10 mL) three times, 12 h each. The solution containing the glycopeptide was dried *in vacuo* and purified by prep C-18 HPLC (0-30% water/acetonitrile; 0.1% trifluoroacetic acid, 5% [0 min]-5% [1 min]-30% [10 min]-30% [31 min], 21min/30 min) to obtain a white amorphous solid compound **7** (2.63 mg) in 10.5% overall yield. ^{1}H NMR (800 MHz, D_2O) δ 7.30 – 7.27 (m, 2H), 7.25 – 7.22 (m, 1H), 7.18 – 7.16 (m, 2H), 7.09 – 7.07 (m, 2H), 6.95 – 6.92 (m, 2H), 6.78 – 6.72 (m, 4H), 4.65 – 4.58 (m, 7H), 4.58 – 4.53 (m, 3H), 4.51 (t, *J* = 8.1 Hz, 1H), 4.45 (d, *J* = 7.9 Hz, 1H), 4.41 (t, *J* = 7.7 Hz, 1H), 4.39 – 4.32 (m, 3H), 4.32 – 4.28 (m, 2H), 4.27 – 4.24 (m, 1H), 4.22 – 4.17 (m, 2H), 4.16 – 4.07 (m, 8H), 4.05 – 3.93 (m, 5H), 3.92 – 3.78 (m, 7H), 3.78 – 3.57 (m, 36H), 3.56 – 3.50 (m, 4H), 3.50 – 3.46 (m, 6H), 3.39 – 3.35 (m, 3H), 3.34 – 3.21 (m, 6H), 3.07 (dd, *J* = 13.9, 6.4 Hz, 1H), 3.01 (dd, *J* = 14.0, 7.5 Hz, 1H), 2.94 – 2.87 (m, 2H), 2.85 – 2.73 (m, 4H), 2.33 – 2.27 (m , 2H), 2.09 – 2.02 (m, 1H), 1.86 (sext, *J* = 6.7 Hz, 1H), 1.48 – 1.40 (m, 2H), 1.40 – 1.34 (m, 1H), 0.83 (d, *J* = 6.1 Hz, 3H), 0.79 (d, *J* = 6.1 Hz, 3H); ^{13}C NMR (201 MHz, D_2O) δ 177.34, 175.62, 175.19, 174.87, 174.76, 173.79, 173.15, 172.28, 171.97, 171.54, 171.11, 170.68, 170.57, 169.17, 154.51, 136.30, 130.59, 130.39, 129.28, 128.61, 127.92, 127.04, 115.39, 103.89, 103.55, 102.90, 102.80, 101.44, 101.41, 101.25, 82.30, 76.72, 76.27, 75.78, 75.27, 75.14, 74.91, 74.84, 74.75, 73.63, 73.14, 72.99, 72.66, 71.69, 70.08, 69.76, 68.63, 68.15, 67.96, 62.87, 62.90, 60.85, 55.16, 54.59, 53.61, 53.48, 53.03, 52.70, 50.21, 42.51, 39.62, 37.02, 36.22, 35.93, 30.12, 26.23, 24.13, 21.86, 20.92. HRMS (ESI) m/z: $[M - 2H]^{2-}$ Calcd for Chemical Formula: $C_{126}H_{182}N_{12}O_{82}$ 1587.5228; Found 1587.5127.

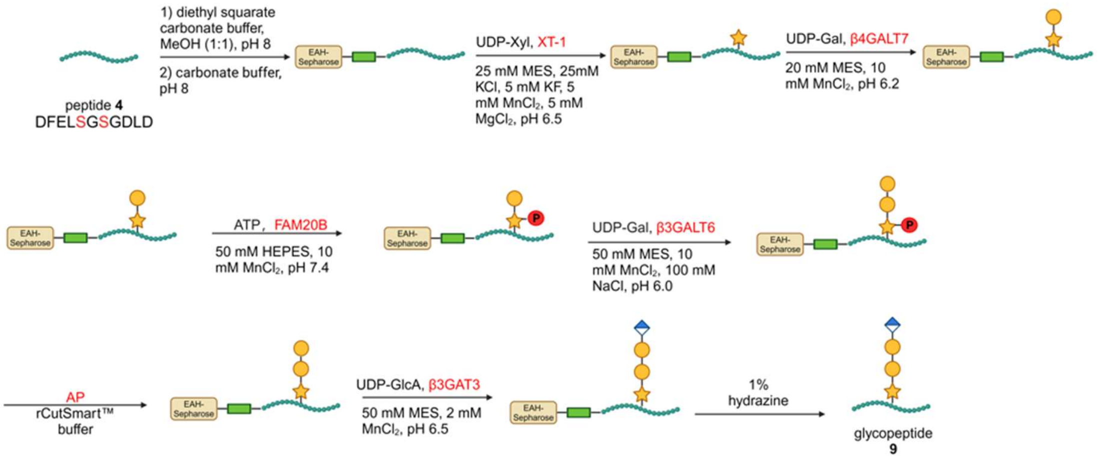

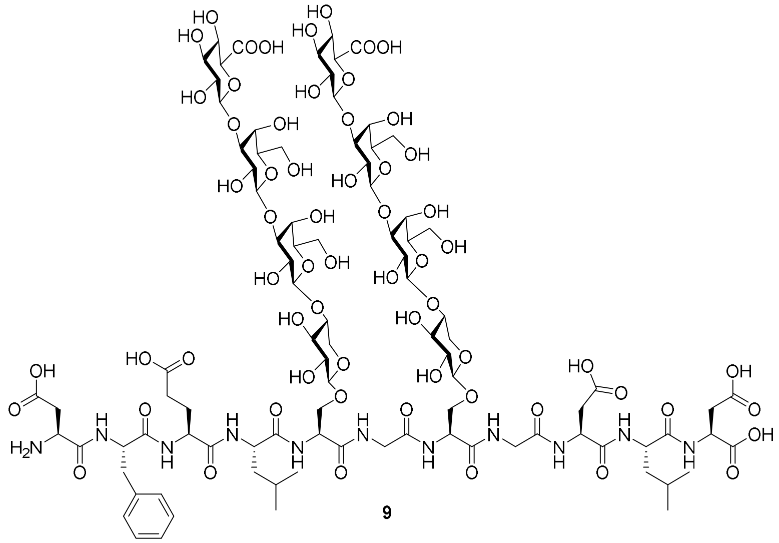

9

Peptide **4** (10 mg, 8.6 μmol) was conjugated to EAH Sepharose (1.92 mL, drained volume) following the general procedure of peptide conjugation to EAH Sepharose. The resulting Sepharose was resuspended in buffer following steps 1 to 6 of the general procedure of enzymatic glycosylation on peptide-conjugated Sepharose. Crude products were obtained after incubating the glycosylated Sepharose with 1% hydrazine (10 mL) three times, 12 h each. The solution containing the glycopeptide was dried *in vacuo* and purified by prep C-18 HPLC (0-50% water/acetonitrile; 0.1% trifluoroacetic acid, 5% [0 min]-5% [2 min]-50% [20 min]-100% [25 min], 19 min/30 min) to obtain a white amorphous solid compound **8** (4.1 mg) in 19.5% yield. ^{1}H NMR (800 MHz, D_2O) δ 7.34 – 7.30 (m, 2H), 7.29 – 7.26 (m, 1H), 7.23 – 7.20 (m, 2H), 4.68 – 4.56 (m, 9H), 4.37 (dd, *J* = 10.2, 8.0 Hz, 2H), 4.38 (t, *J* = 8.0 Hz, 2H), 4.34 – 4.31 (m, 1H), 4.30 – 4.27 (m, 2H), 4.20 – 4.16 (m, 1H), 4.16 – 4.08 (m, 6H), 4.08 – 4.00 (m, 3H), 4.00 – 3.94 (m, 2H), 3.93 – 3.87 (m, 4H), 3.81 – 3.59 (m, 23H), 3.55 (t, *J* = 8 Hz, 2H), 3.50 – 3.45 (m, 4H), 3.39 – 3.31 (m, 4H), 3.28 (q, *J* = 8.5 Hz, 2H), 3.07 (dd, *J* = 13.9, 8.0 Hz, 1H), 3.01 (dd, *J* = 13.9, 8.0 Hz, 1H), 2.91 – 2.72 (m, 6H), 2.36 – 2.31 (m, 2H), 2.02 – 1.96 (m, 1H), 1.87 – 1.82 (m, 1H), 1.65 – 1.52 (m, 6H), 0.83 (d, *J* = 5.5 Hz, 3H), 0.90 – 0.86 (m, 6H), 0.81 (d, *J* = 5.5 Hz, 3H); ^{13}C NMR (201 MHz, D_2O) δ 177.25, 175.03, 174.86, 174.52, 173.82, 172.59, 172.28, 172.21, 171.70, 171.45, 171.35, 170.95, 168.87, 135.95, 129.07, 128.73, 127.22, 103.88, 103.55, 102.92, 102.88, 101.28, 82.28, 81.92, 76.26, 75.68, 75.12, 74.86, 74.69, 73.65, 73.02, 72.97, 72.61, 71.55, 70.04, 69.72, 68.56, 68.37, 68.07, 67.98, 62.91, 60.97, 60.85, 55.23, 53.65, 53.48, 52.53, 52.43, 52.34, 50.16, 49.97, 42.58, 42.51, 39.53, 39.38, 36.64, 36.18, 36.06, 35.52, 29.95, 26.35, 24.23, 24.12, 22.20, 22.09, 20.88, 20.47. HRMS (ESI) m/z: $[M + 2H]^{2+}$ Calcd for Chemical Formula: $C_{94}H_{145}N_{11}O_{62}$ 1209.9257; Found 1209.9203.

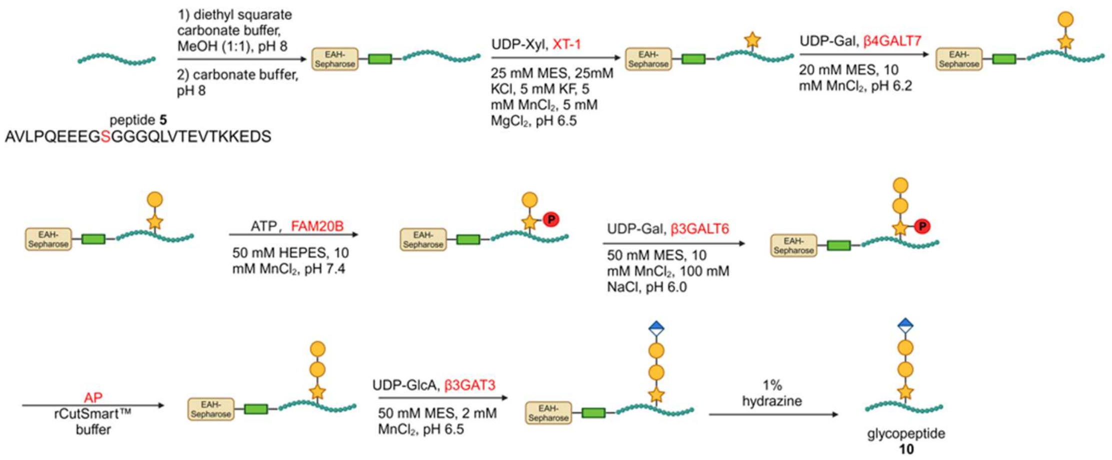

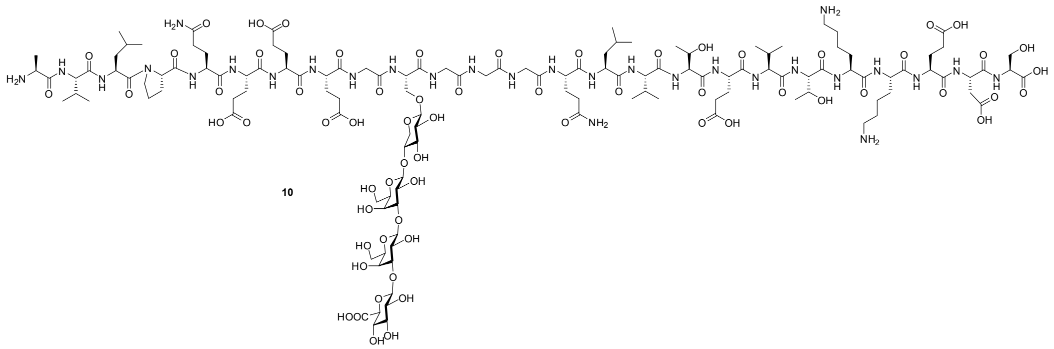

10

Peptide **5** (10 mg, 3.86 μmol) was conjugated to EAH Sepharose (0.86 mL, drained volume) following the general procedure of peptide conjugation to EAH Sepharose. The resulting Sepharose was resuspended in buffer following steps 1 to 6 of the general procedure of enzymatic glycosylation on peptide-conjugated Sepharose. Crude products were obtained after incubating glycosylated Sepharose with 1% hydrazine (10 mL) three times, 12 h each. The solution containing the glycopeptide was dried *in vacuo* and purified by prep C-18 HPLC (0-50% water/acetonitrile; 0.1% trifluoroacetic acid, 5% [0 min]-5% [2 min]-50% [20 min]-100% [25 min], 20.5 min/25 min) to obtain a white amorphous solid compound **10** (1.2 mg) in 9.5% yield. ^{1}H NMR (800 MHz, D_2O) δ 4.73 – 4.70 (m, 1H), 4.66 (d, *J* = 7.9 Hz, 1H), 4.63 – 4.58 (m, 3H), 4.46 (d, *J* = 7.9 Hz, 1H), 4.40 – 4.20 (m, 14H), 4.20 – 4.16 (m, 1H), 4.16 – 4.00 (m , 9H), 4.00 – 3.86 (m, 10H), 3.86 – 3.59 (m, 14H), 3.55 (t, *J* = 9.2 Hz, 1H), 3.51 – 3.45 (m, 2H), 3.39 – 3.31 (m, 4H), 3.25 (t, *J* = 8.5 Hz, 1H), 2.96 – 2.87 (m, 5H), 2.79 (dd, *J* = 16.9, 8.2 Hz, 1H), 2.49 – 2.37 (m, 10H), 2.37 – 2.24 (m, 5H), 2.12 – 1.90 (m, 15H), 1.89 – 1.82 (m, 1H), 1.80 – 1.74 (m, 2H), 1.74 – 1.67 (m, 2H), 1.67 – 1.60 (m, 5H), 1.60 – 1.51 (m, 5H), 1.46 (d, *J* = 7.1 Hz, 3H), 1.43 – 1.30 (m, 4H), 1.17 – 1.12 (m, 6H), 0.98 – 0.84 (m, 27H), 0.82 (d, *J* = 5.7 Hz, 3H); ^{13}C NMR (201 MHz, D_2O) δ 177.77, 177.70, 177.11, 177.02, 176.96, 176.93, 176.89, 174.57, 174.44, 174.18, 173.72, 173.58, 173.44, 173.35, 173.10, 173.07, 172.98, 172.91, 172.46, 172.02, 171.84, 171.52, 171.46, 171.30, 170.60, 103.89, 103.56, 102.78, 101.29, 82.24, 81.91, 76.28, 75.39, 75.09, 74.88, 74.69, 73.67, 72.93, 72.65, 71.46, 70.05, 69.73, 68.61, 68.37, 68.01, 66.88, 62.93, 61.34, 60.99, 60.84, 60.45, 59.74, 59.61, 59.33, 59.18, 58.97, 55.80, 53.67, 53.53, 53.41, 53.30, 53.02, 53.00, 52.92, 52.86, 52.47, 50.28, 50.04, 48.72, 47.82, 42.47, 42.34, 39.06, 38.84, 35.70, 30.96, 30.94, 30.30, 30.21,

29.98, 29.92, 29.25, 26.20, 26.14, 26.06, 26.00, 24.66, 24.28, 24.24, 22.30, 21.95, 21.91, 20.80, 20.44, 18.78, 18.76, 18.32, 18.30, 18.28, 17.82, 17.66, 16.57. HRMS (ESI) m/z: $[M+3H]^{3+}$ Calcd for Chemical Formula: $C_{131}H_{216}N_{29}O_{64}$ 1073.8223; Found 1073.8217.

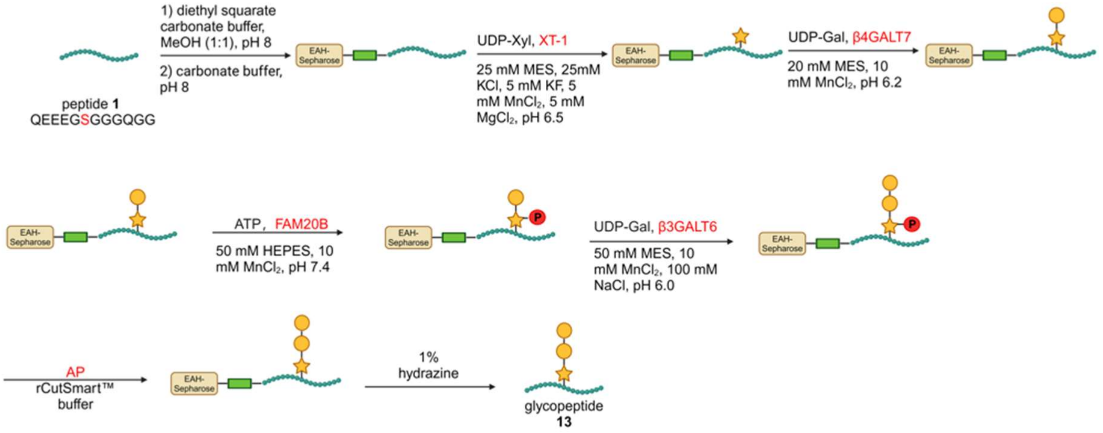

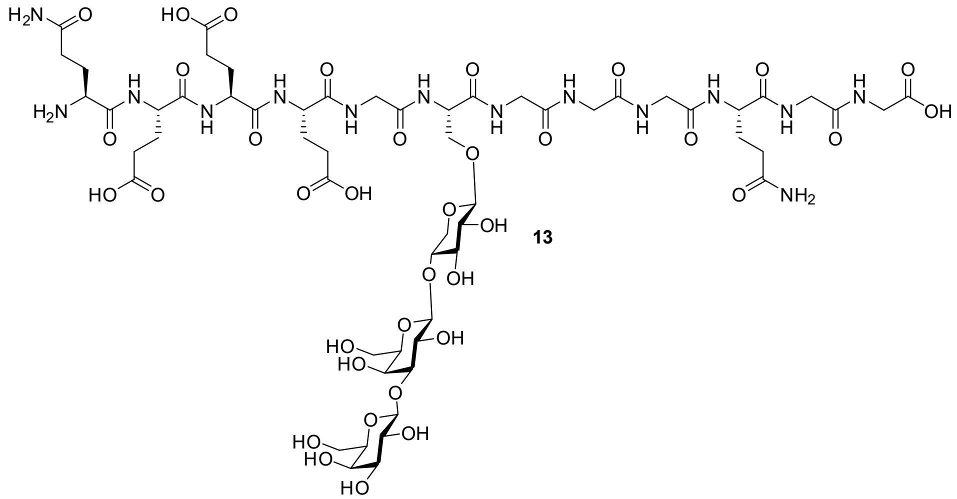

13

Peptide **1** (10 mg, 9.1 μmol) was conjugated to EAH Sepharose (2 mL, drained volume) following the general procedure of peptide conjugation to EAH Sepharose. The resulting Sepharose was resuspended in buffer following steps 1 to 5 of the general procedure of enzymatic glycosylation on peptide-conjugated Sepharose. To monitor the reaction, crude products were obtained after incubating glycosylated Sepharose with 1% hydrazine (10 mL) three times, 12 h each. The solution containing the glycopeptides was dried *in vacuo* and purified by prep C-18 HPLC (water/acetonitrile; 0.1% trifluoroacetic acid, 0% [0 min]-10% [15 min], 7.2 min/15 min) to obtain a white amorphous solid compound **13**. ^{1}H NMR (800 MHz, D_2O) δ 4.62 (t, J = 4.8 Hz, 1H), 4.56 (d, J = 7.8 Hz, 1H), 4.47 (d, J = 7.9 Hz, 1H), 4.40 – 4.32 (m, 4H), 4.18 (dd, J = 6.9, 5.2 Hz, 1H), 4.14 (d, J = 3.3 Hz, 1H), 4.08 – 4.01 (m, 2H), 4.01 – 3.88 (m, 14H), 3.87 (d, J = 3.4 Hz, 1H), 3.80 – 3.65 (m, 7H), 3.65 – 3.59 (m, 3H), 3.57 – 3.53 (m, 2H), 3.35 (t, J = 11.1 Hz, 1H), 3.26 (t, J =8.7 Hz, 1H), 2.54 – 2.36 (m, 9H), 2.34 – 2.31 (m, 1H) 2.18 – 2.00 (m, 6H), 2.00 – 1.89 (m, 4H); ^{13}C NMR (201 MHz, D_2O) δ 182.22, 177.81, 176.94, 176.89, 176.88, 176.75, 174.95, 173.76, 173.65, 173.52, 173.14,

173.05, 172.92, 172.01, 171.60, 171.55, 171.42, 169.09, 116.96, 115.51, 104.25, 102.79, 102.77, 101.28, 81.98, 76.27, 74.98, 74.87, 73.66, 72.64, 72.39, 70.91, 69.72, 68.64, 68.46, 62.92, 60.99, 60.86, 56.66, 53.61, 53.15, 53.04, 53.02, 52.97, 52.93, 52.81, 52.77, 52.14, 42.65, 42.42, 42.36, 42.33, 42.29, 41.20, 41.14, 30.95, 29.89, 29.85, 29.75, 29.71, 29.15, 26.50, 26.37, 26.07, 26.06, 26.01, 25.84, 25.82. HRMS (ESI) m/z: $[M + H]^+$ Calcd for Chemical Formula: $C_{57}H_{91}N_{14}O_{36}$ 1547.5712; Found 1547.5770.

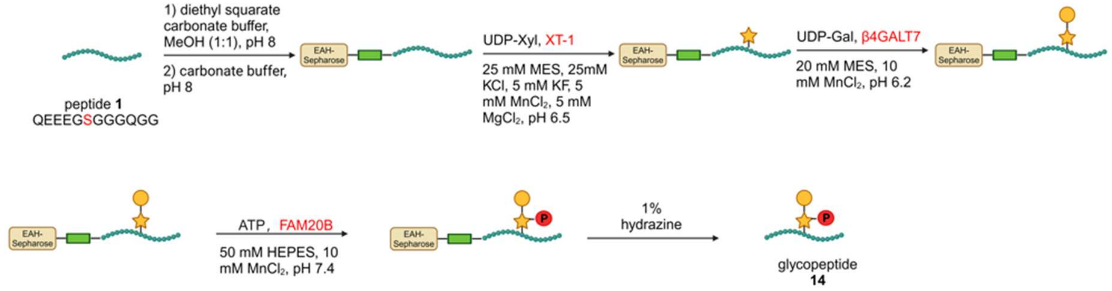

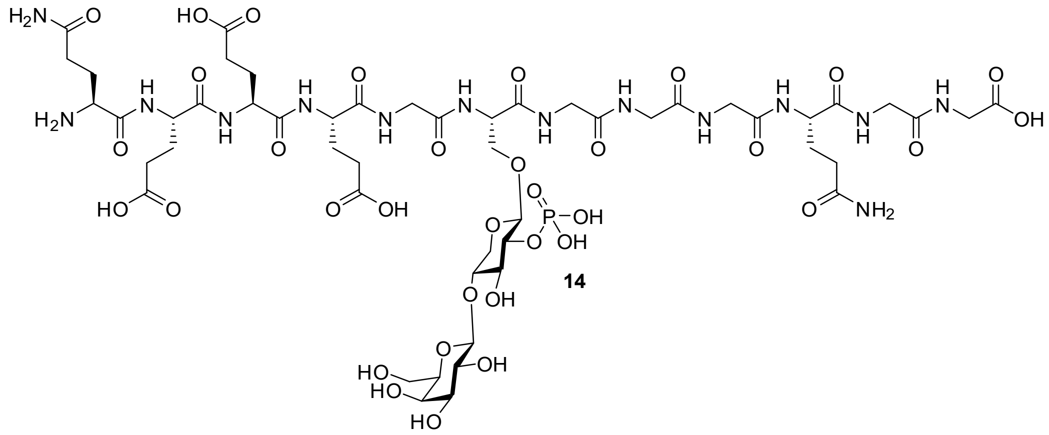

14

Peptide **1** (10 mg, 9.1 μmol) was conjugated to EAH Sepharose (2 mL, drained volume) following the general procedure of peptide conjugation to EAH Sepharose. The resulting Sepharose was resuspended in buffer following steps 1 to 3 of the general procedure of enzymatic glycosylation on peptide-conjugated Sepharose. To monitor the reaction, crude products were obtained after incubating glycosylated Sepharose with 1% hydrazine (10 mL) three times, 12 h each. The solution containing the glycopeptide was dried *in vacuo* and purified by prep C-18 HPLC (water/acetonitrile; 0.1% trifluoroacetic acid, 0% [0 min]-10% [15 min], 8 min/15 min) to obtain a white amorphous solid compound **14**. ^{1}H NMR (800 MHz, D_2O) δ 4.53 – 4.48 (m, 2H), 4.42 – 4.31 (m, 5H), 4.27 (d, J = 9.8 Hz, 1H), 4.07 – 3.90 (m, 13H), 3.87 – 3.83 (m, 2H), 3.80 – 3.74 (m, 3H), 3.70 – 3.66 (m, 3H), 3.66 – 3.63 (m, 1H), 3.61 – 3.58 (m, 1H), 3.46 (dd, J = 9.7, 7.9 Hz, 1H), 3.36 (t, J = 10.8 Hz, 1H), 2.52 – 2.30 (m, 10H), 2.17 – 2.03 (m, 6H), 1.99 – 1.92 (m, 4H); ^{13}C NMR (201 MHz, D_2O) δ 177.83, 176.90, 176.76, 173.78, 173.45, 172.87, 172.81, 172.67, 172.29, 172.12, 171.65, 171.58, 171.54, 169.08, 101.82, 101.58, 77.03, 75.71, 75.26, 73.13, 72.49, 70.50, 68.95, 68.55, 62.75, 62.36, 61.05, 59.19, 56.67, 54.09, 53.15, 53.06, 52.85, 52.80, 52.15, 42.93, 42.51, 42.38, 42.31,

42.26, 30.95, 29.84, 29.75, 26.49, 26.35, 26.24, 26.07, 26.00, 25.87. HRMS (ESI) m/z: [M + H]$^+$ Calcd for Chemical Formula: $C_{51}H_{82}N_{14}O_{34}P$ 1465.4847; Found 1465.4823.

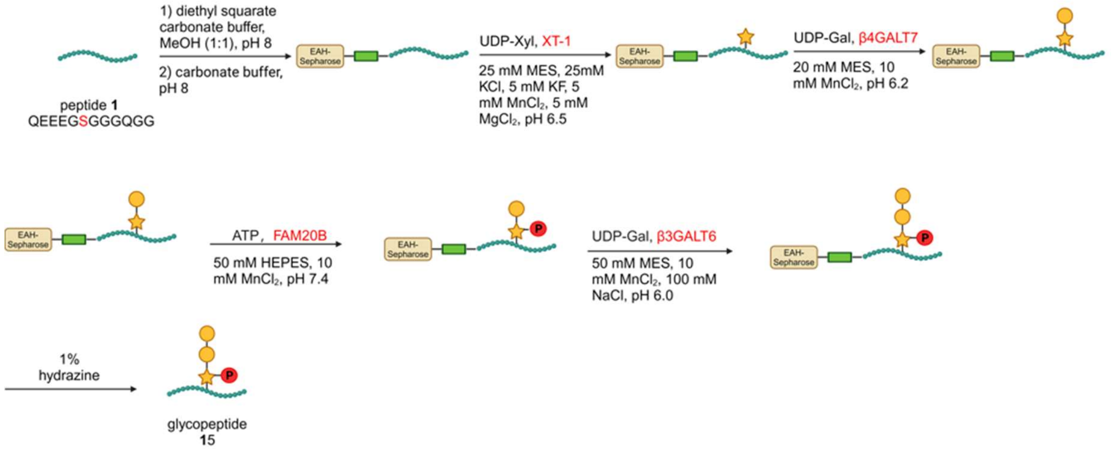

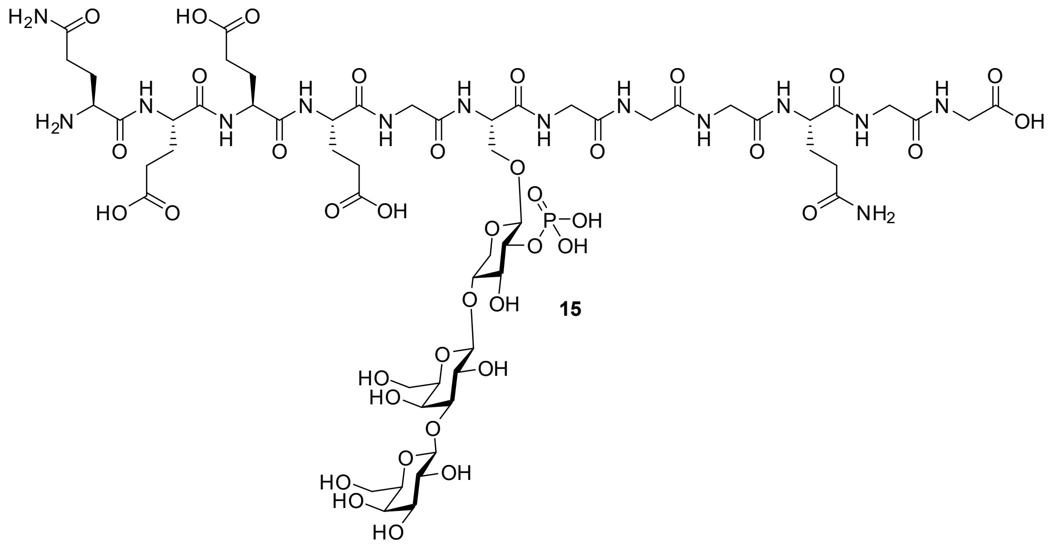

15

Peptide **1** (10 mg, 9.1 μmol) was conjugated to EAH Sepharose (2 mL, drained volume) following the general procedure of peptide conjugation to EAH Sepharose. The resulting Sepharose was resuspended in buffer following steps 1 to 4 of the general procedure of enzymatic glycosylation on peptide-conjugated Sepharose. To monitor the reaction, crude products were obtained after incubating glycosylated Sepharose with 1% hydrazine (10 mL) three times, 12 h each. Basic solutions containing glycopeptides were dried *in vacuo* and purified by prep C-18 HPLC (water/acetonitrile; 0.1% trifluoroacetic acid, 0% [0 min]-10% [15 min], 5.4 min/15 min) to obtain a white amorphous solid compound **15**. ^{1}H NMR (800 MHz, D_2O) δ 4.56 (d, J = 7.8 Hz, 1H), 4.52 – 4.48 (m, 2H), 4.47 (d, J = 7.9 Hz, 1H), 4.42 – 4.31 (m, 4H), 4.29 – 4.26 (m, 1H), 4.14 (d, J = 3.3 Hz, 1H), 4.06 – 4.00 (m, 4H), 3.99 – 3.90 (m, 9H), 3.88 – 3.82 (m, 2H), 3.81 – 3.74 (m, 4H), 3.74 – 3.65 (m, 6H), 3.65 – 3.60 (m, 3H), 3.56 –3.53 (m, 1H), 3.37 (t, J = 11.4 Hz, 1H), 2.49 – 2.41 (m, 6H), 2.41 – 2.35 (m, 2H), 2.35 – 2.31 (m, 2H), 2.16 – 2.04 (m, 6H), 2.00 – 1.92 (m, 4H); ^{13}C NMR (201 MHz, D_2O) δ 182.25, 177.80, 176.90, 176.87, 176.86, 176.82, 176.76, 176.75, 173.78, 173.44, 173.19, 173.06, 172.85, 172.79, 172.65, 172.12, 171.65, 169.09, 104.25, 101.83, 101.18, 81.95, 77.08, 77.05, 75.63, 74.97, 74.89, 73.08, 72.39, 70.91, 69.70, 68.97, 68.46, 68.35, 62.72,

60.99, 60.85, 59.19, 56.66, 54.08, 53.16, 53.03, 52.97, 52.83, 52.81, 52.78, 52.15, 42.93, 42.51, 42.38, 42.29, 42.26, 41.00, 40.98, 30.95, 29.84, 29.73, 29.68, 29.65, 26.48, 26.36, 26.21, 26.18, 26.02, 25.97, 25.85. HRMS (ESI) m/z: $[M + 2H]^{2+}$ Calcd for Chemical Formula: $C_{57}H_{93}N_{14}O_{39}P$ 814.2722; Found 814.2753.

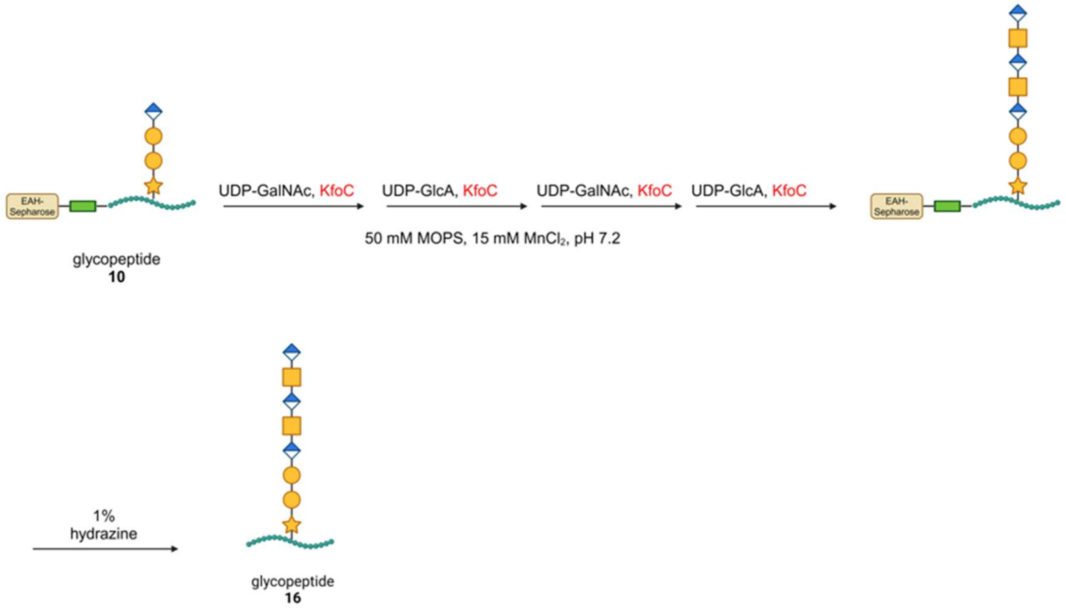

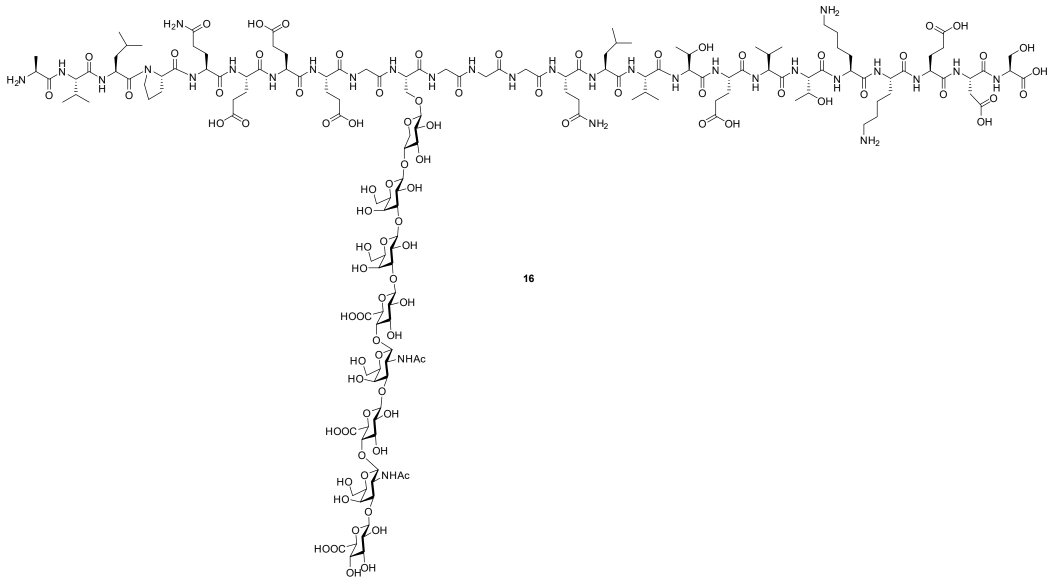

16

Glycopeptide **10** (2 mg, 0.62 μmol) was conjugated to EAH Sepharose (0.4 mL, drained volume) following the general procedure of peptide conjugation to EAH Sepharose. The resulting Sepharose was resuspended in buffer following steps 7 to 8 from the general procedure of enzymatic glycosylation on peptide-conjugated Sepharose. Steps 7 and 8 were repeated once to afford the octasaccharide bearing glycopeptide on Sepharose. Crude products were obtained after incubating glycosylated Sepharose with 1% hydrazine (10 mL)

three times, 12 h each. The solution containing the glycopeptide was dried *in vacuo* and purified by prep C-18 HPLC (0-50% water/acetonitrile; 0.1% trifluoroacetic acid, 5% [0 min]-5% [2 min]-50% [20 min]-100% [25 min], 19.8 min/25 min) to obtain a white amorphous solid compound **16** (1.3 mg) in 54% yield. ^{1}H NMR (600 MHz, D_2O) δ 4.77 – 4.74 (m, 1H), 4.70 – 4.65 (m, 3H), 4.59 – 4.49 (m, 6H), 4.46 – 4.37 (m, 8H), 4.37 – 4.26 (m, 6H), 4.24 – 4.07 (m, 12H), 4.04 – 3.92 (m, 15H), 3.91 – 3.66 (m, 24H), 3.66 – 3.45 (m, 5H), 3.42 – 3.29 (m, 4H), 3.03 – 2.93 (m, 5H), 2.91 – 2.84 (m, 1H), 2.56 – 2.43 (m, 10H), 2.43 – 2.30 (m, 5H), 2.19 – 1.96 (m, 25H), 1.96 – 1.88 (m, 1H), 1.88 – 1.80 (m, 2H), 1.79 – 1.63 (m, 2H), 1.73 – 1.65 (m, 5H), 1.66 – 1.56 (m, 5H), 1.52 (d, *J* = 7.1 Hz, 3H), 1.48 – 1.36 (m, 4H), 1.21 (t, *J* = 6.6 Hz, 6H), 1.00 – 0.91 (m, 27H), 0.88 (d, *J* = 5.8 Hz, 3H); ^{13}C NMR (151 MHz, D_2O) δ 177.83, 177.75, 176.91, 176.84, 176.80, 174.88, 174.62, 174.26, 174.01, 173.77, 173.73, 173.64, 173.53, 173.42, 173.36, 173.16, 173.10, 172.98, 172.93, 172.53, 172.14, 172.06, 171.94, 171.62, 171.51, 171.37, 170.66, 117.29, 115.35, 104.31, 104.18, 103.93, 103.80, 102.86, 101.38, 101.23, 82.32, 81.98, 80.11, 80.05, 79.89, 79.81, 76.39, 75.05, 74.93, 74.72, 74.68, 74.08, 73.76, 73.67, 72.72, 72.56, 72.45, 72.06, 71.33, 70.08, 69.80, 68.43, 68.19, 67.60, 66.93, 63.01, 61.13, 61.05, 60.92, 60.88, 60.56, 59.85, 59.70, 59.40, 59.27, 59.04, 55.21, 53.72, 53.62, 53.53, 53.41, 53.05, 52.93, 52.57, 51.05, 50.14, 50.11, 48.81, 47.88, 42.78, 42.55, 42.43, 39.52, 39.14, 38.96, 35.46, 31.03, 30.33, 30.27, 29.98, 29.93, 29.90, 29.86, 29.81, 29.30, 26.71, 26.54, 26.25, 26.20, 26.05, 24.72, 24.35, 24.31, 22.36, 22.01, 20.89, 20.53, 18.85, 18.83, 18.39, 18.37, 18.34, 17.89, 17.88, 17.72, 16.64. HRMS (ESI) m/z: $[M + 3H]^{3+}$ Calcd for Chemical Formula: $C_{159}H_{258}N_{31}O_{86}$ 1326.5633; Found 1326.5579.

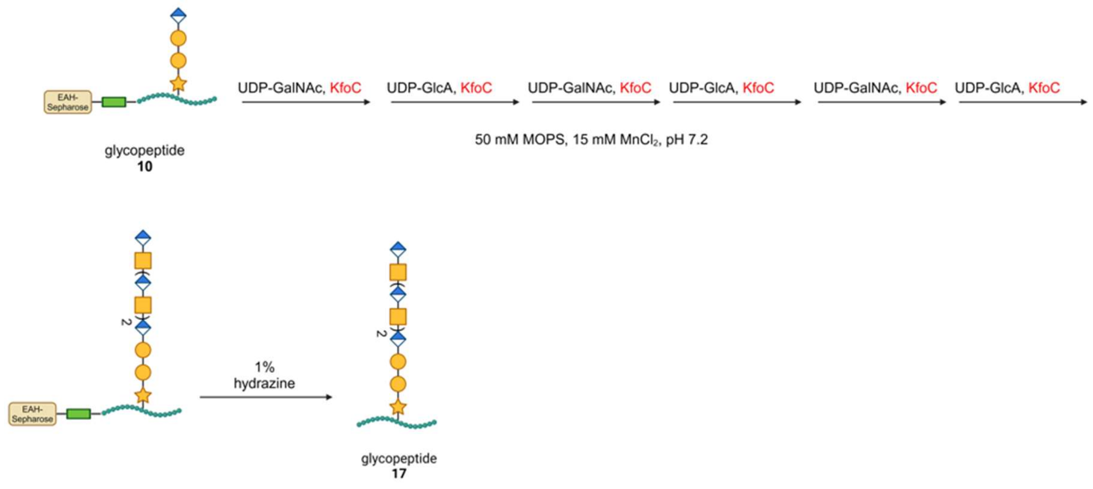

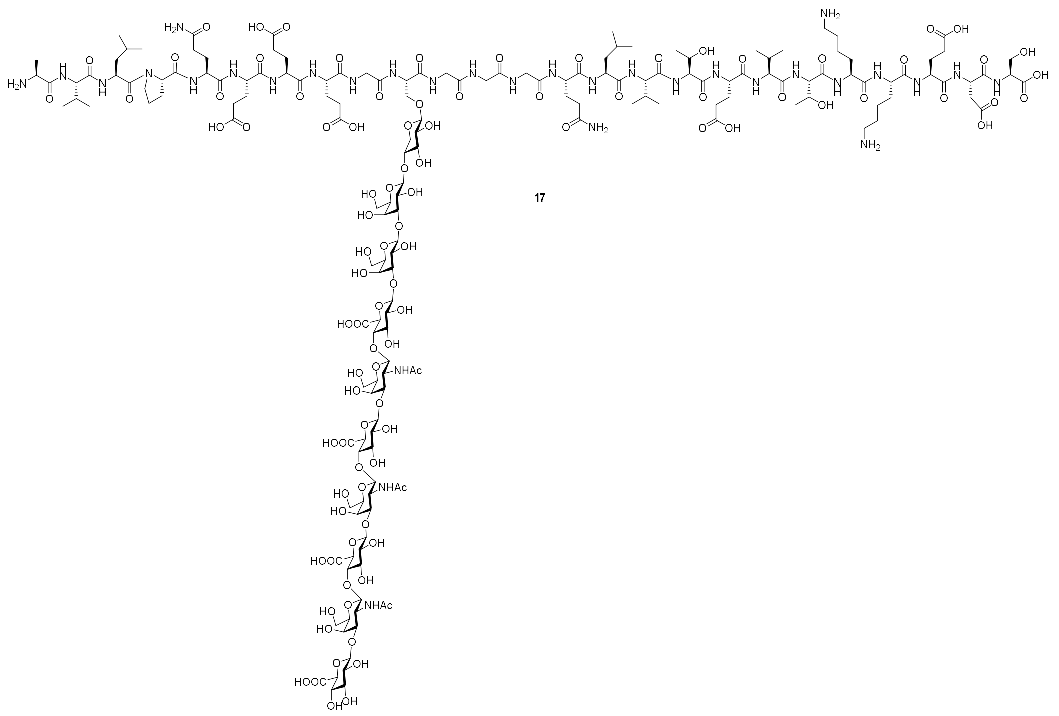

17

Glycopeptide **10** (5 mg, 1.6 µmol) was dissolved in 1 mL of KfoC reaction buffer (25 mM MOPS, 15 mM $MnCl_2$, pH 7.2) containing 100 µg KfoC, UDP-GalNAc (2.9 mg, 4.7 µmol). The reaction mixture was incubated for 3 h at 37 ºC. Upon completion, 1 mL of MeOH was added and the mixture was centrifuged under 10,000 g for 10 min and dried *in vacuo*. The mixture was loaded to a Biotage® Sfar C18 column to perform a solid phase extraction. Fractions containing the desired product were lyophilized and redissolved in another 1 mL of KfoC reaction buffer containing 100 µg KfoC, UDP-GlcA (3 mg, 5.16 µmol). The reaction was again incubated for 3 h at 37 ºC and was purified as aforementioned. These two reactions were repeated three times to obtain the desired decasaccharide glycopeptide. The final product was purified by prep C18 HPLC (0-50% water/acetonitrile; 0.1% trifluoroacetic acid, 5% [0 min]-5% [2 min]-50% [20 min]-100% [25 min], 18 min/25 min) to obtain a white amorphous solid compound **17** (3.6 mg) in 51% yield. ^{1}H NMR (800 MHz, D_2O) δ 4.73 – 4.66 (m, 4H), 4.57 – 4.48 (m, 7H), 4.48 – 4.31 (m, 16H), 4.30 – 4.24 (m, 2H), 4.25 – 4.09 (m, 11H), 4.09 – 3.95 (m, 10H), 3.93 – 3.68 (m, 35H), 3.68 – 3.58 (m, 5H), 3.53 – 3.46 (m, 3H), 3.45 – 3.40 (m, 1H), 3.38 (t, *J* = 8.9 Hz, 1H), 3.35 – 3.31 (m, 1H), 3.03 – 2.98 (m, 4H), 2.79 (dd, *J* = 16.4, 5.0 Hz, 1H), 2.68 (dd, *J* = 16.4, 4.9 Hz, 1H), 2.44 – 2.27 (m, 15H), 2.18 – 1.94 (m, 29H), 1.90 – 1. 82 (m, 2H), 1.82 – 1.75 (m, 2H), 1.75 – 1.68 (m, 5H), 1.69 – 1.59 (m, 5H), 1.55 (d, *J* = 7.1 Hz, 3H), 1.51 – 1.38 (m, 3H), 1.31 (br, 1H), 1.23 (dd, *J* = 12.3, 6.4 Hz, 6H), 1.01 – 0.92 (m, 27H), 0.90 (d, *J* = 5.9 Hz, 3H). ^{13}C NMR (201 MHz, D_2O) δ 175.15, 170.78, 104.47, 103.96, 101.08, 80.48, 79.88, 76.51, 76.32, 75.44, 75.12, 73.85, 72.93, 72.83, 72.75, 72.69, 71.90, 70.08, 69.96, 68.54, 67.94, 67.83, 67.20, 67.17, 62.20, 61.23, 61.07, 60.75, 59.62, 59.29, 58.97, 53.98, 53.82, 53.65, 52.53, 51.72, 51.08, 50.11, 48.98, 42.70, 42.65, 42.54, 39.15, 33.35, 31.09, 30.61, 30.45, 30.13, 27.55, 27.39, 26.26, 24.81, 24.32, 22.55, 22.07, 21.91, 20.94, 20.62, 18.85, 18.52, 18.04, 16.75.

HRMS (ESI) m/z: $[M - 3H]^{3-}$ Calcd for Chemical Formula: $C_{173}H_{275}N_{32}O_{97}$ 1450.9198; Found 1450.9186.

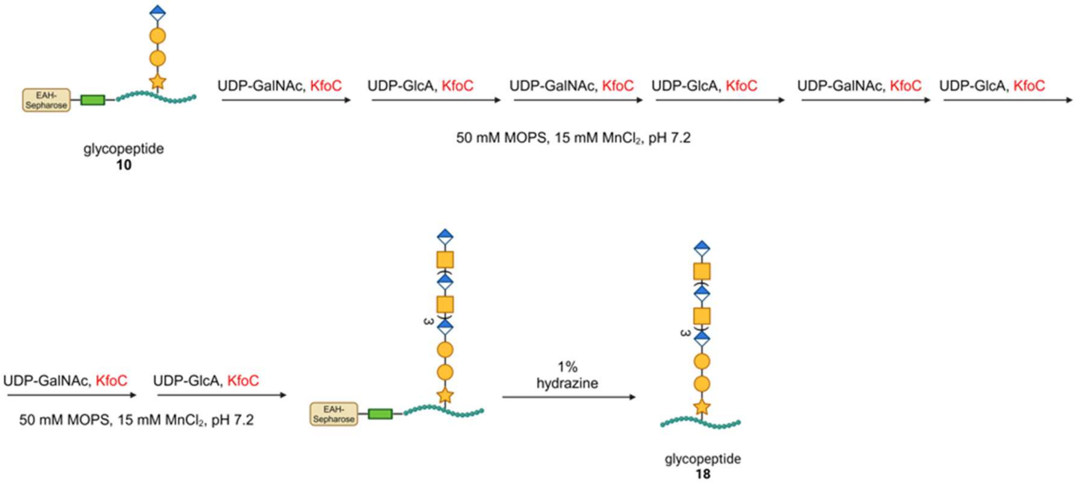

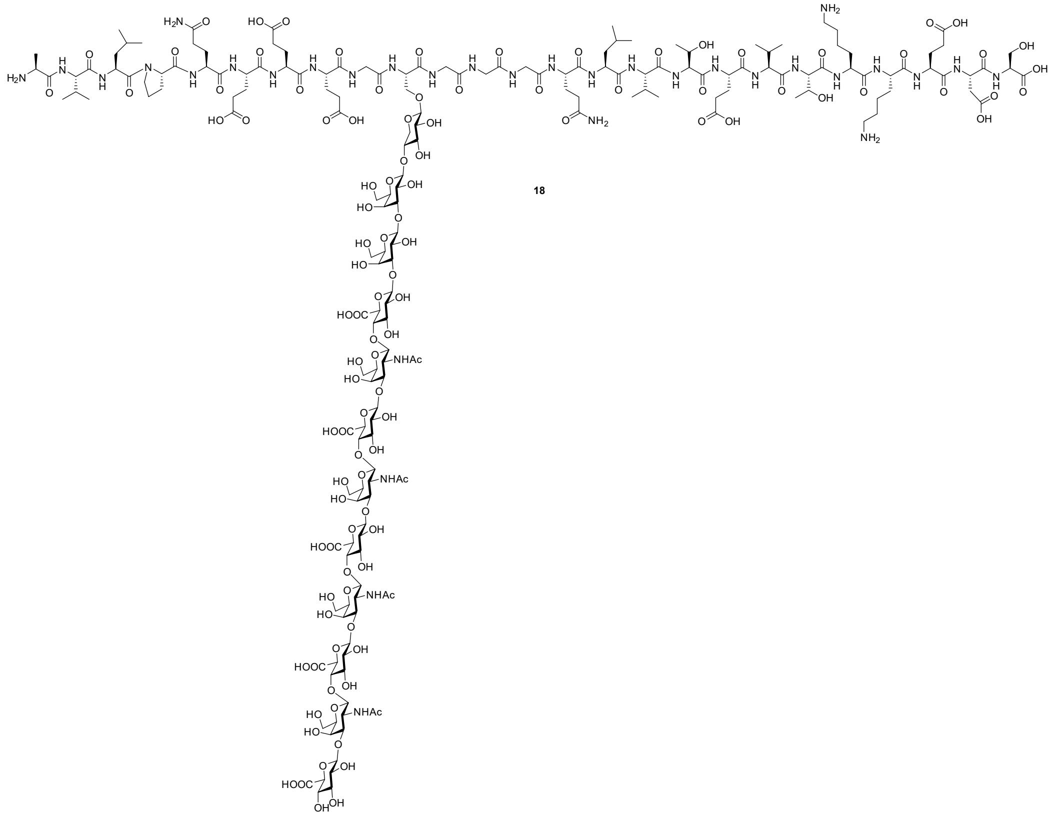

18

Glycopeptide **10** (5 mg, 1.6 μmol) was dissolved in 1 mL of KfoC reaction buffer (25 mM MOPS, 15 mM $MnCl_2$, pH 7.2) containing 100 μg KfoC, UDP-GalNAc (2.9 mg, 4.7 μmol). The reaction mixture was incubated for 3 h at 37 ºC. Upon completion, 1 mL of MeOH was added and the mixture was centrifuged under 10,000 g for 10 min and dried *in vacuo*. The mixture was loaded to a Biotage® Sfar C18 column to perform a solid phase extraction.

Fractions containing the desired product were lyophilized and redissolved in another 1 mL of KfoC reaction buffer containing 100 µg KfoC, UDP-GlcA (3 mg, 5.2 µmol). The reaction was again incubated for 3 h at 37 °C and was purified as aforementioned. These two reactions were repeated three times to obtain the desired dodecasaccharide glycopeptide. The final product was purified by prep C18 HPLC (0-50% water/acetonitrile; 0.1% trifluoroacetic acid, 5% [0 min]-5% [2 min]-50% [20 min]-100% [25 min], 17 min/25 min) to obtain a white amorphous solid compound **18** (3.6 mg) in 48% yield. ^{1}H NMR (800 MHz, D_2O) δ 4.73 – 4.65 (m, 6H), 4.58 – 4.47 (m, 9H), 4.45 – 4.43 (m, 1H), 4.43 – 4.31 (m, 14H), 4.31 – 4.27 (m, 2H), 4.26 – 4.22 (m, 1H), 4.21 – 4.08 (m, 14H), 4.05 – 3.93 (m, 14H), 3.89 – 3.65 (m, 41H), 3.65 – 3.56 (m, 3H), 3.51 – 3.44 (m, 3H), 3.43 – 3.38 (m, 1H), 3.36 (t, *J* = 8.6 Hz, 3H), 3.33 – 3.29 (m, 2H), 3.03 – 2.98 (m, 4H), 2.81 (dd, *J* = 16.3, 4.8 Hz, 1H), 2.70 (dd, *J* = 16.3, 4.8 Hz, 1H), 2.44 – 2.29 (m, 15H), 2.18 – 1.88 (m, 31H), 1.87 – 1.80 (m, 3H), 1.80 – 1.73 (m, 2H), 1.73 – 1.67 (m, 5H), 1.66 – 1.55 (m, 5H), 1.52 (d, *J* = 7.1 Hz, 3H), 1.48 – 1.37 (m, 4H), 1.21 (dd, *J* = 11.0, 6.4 Hz, 6H), 1.00 – 0.91 (m, 27H), 0.88 (d, *J* = 5.9 Hz, 3H). ^{13}C NMR (201 MHz, D_2O) δ 179.39, 178.70, 177.77, 177.66, 176.80, 175.73, 174.97, 174.33, 173.58, 173.47, 173.25, 173.04, 172.45, 172.07, 170.75, 104.33, 104.15, 103.00, 100.84, 80.38, 79.67, 76.51, 76.19, 75.39, 75.28, 74.95, 73.94, 73.69, 72.81, 72.71, 72.45, 71.79, 70.07, 69.91, 68.46, 67.72, 66.99, 62.02, 61.06, 60.63, 59.76, 59.48, 59.27, 59.20, 59.11, 58.97, 57.18, 53.86, 53.47, 52.50, 53.34, 51.33, 50.97, 50.08, 48.83, 47.99, 47.88, 42.81, 42.54, 39.56, 39.14, 32.01, 31.06, 31.03, 30.37, 29.98, 29.32, 26.96, 26.76, 26.26, 26.19, 24.76, 24.37, 24.31, 22.47, 22.41, 22.03, 21.92, 21.89, 20.91, 20.53, 18.88, 18.81, 18.42, 18.40, 18.37, 17.86, 17.81, 17.74, 16.65. HRMS (ESI) m/z: $[M - 3H]^{3-}$ Calcd for Chemical Formula: $C_{187}H_{296}N_{33}O_{108}$ 1577.2903; Found 1577.2984.

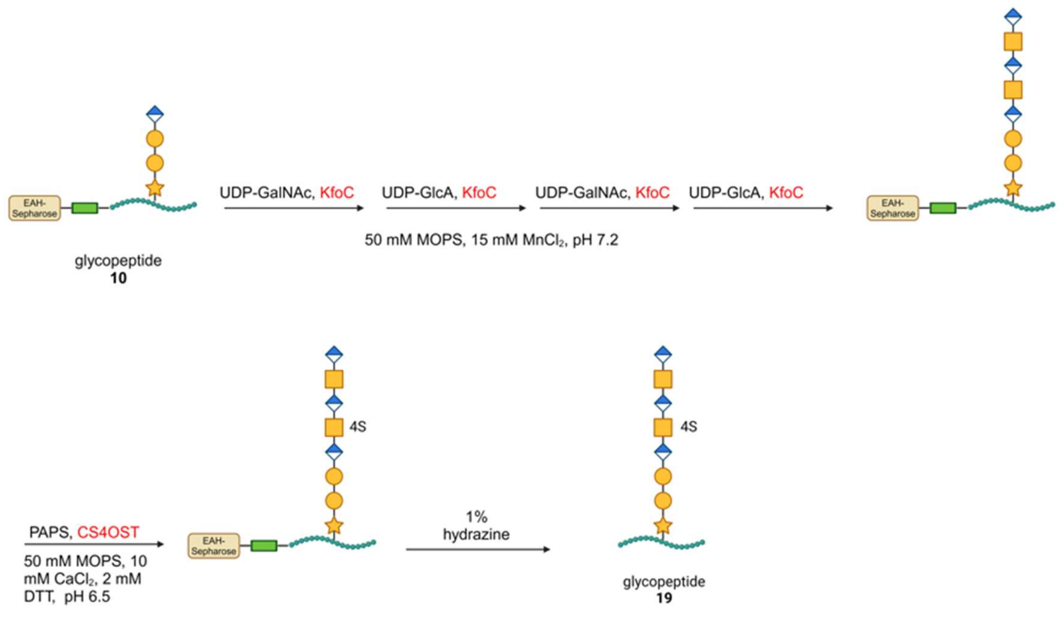

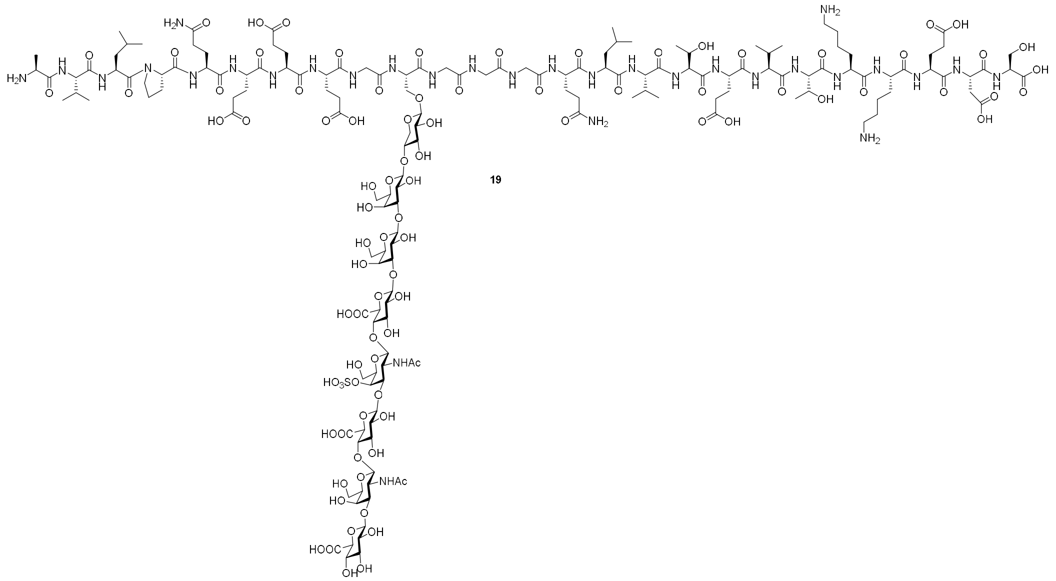

19

Glycopeptide **10** (2 mg, 0.67 μmol) was conjugated to EAH Sepharose (0.4 mL, drained volume) following the general procedure of peptide conjugation to EAH Sepharose. The resulting Sepharose was resuspended in buffer following steps 7 to 9 from the general procedure of enzymatic glycosylation on peptide-conjugated Sepharose. Steps 7 and 8 were repeated twice to afford octasaccharide-bearing glycopeptide. Finally, glycopeptide-conjugated Sepharose was sulfated following step 9. Crude products were obtained after incubating glycosylated Sepharose with 1% hydrazine (10 mL) three times, 12 h each. Basic solutions containing glycopeptides were dried *in vacuo* and purified by prep C-18 HPLC (-0-50% water, 50 mM ammonium formate /acetonitrile, 5% [0 min]-5% [2 min]-50% [20 min]-100% [25 min], 18.1 min/25 min) to obtain a white amorphous solid compound **19** (0.98 mg) in an overall yield of 36%. ^{1}H NMR (800 MHz, D_2O) δ 4.72 – 4.64 (m, 5H), 4.57 (d, *J* = 8.8 Hz, 1H), 4.54 – 4.51 (m, 2H), 4.50 (d, *J* = 7.6 Hz, 1H), 4.47 (d, *J* = 7.5 Hz, 1H), 4.45 (d, *J* = 8 Hz, 1H), 4.43 – 4.23 (m, 12H), 4.23 – 4.09 (m, 9H), 4.08 – 3.93 (m, 10H), 3.91 – 3.54 (m, 35H), 3.51 – 3.35 (m, 5H), 3.35 – 3.30 (m, 2H), 3.03 – 2.98 (m, 4H), 2.81 – 2.76 (m, 1H), 2.67 (dd, *J* = 16.3, 4.7 Hz, 1H), 2.44 – 2.25 (m, 15H), 2.16 – 1.92 (m, 26H), 1.90 – 1.81 (m, 2H), 1.81 – 1.73 (m, 2H), 1.73 – 1.67 (m, 5H), 1.67 – 1.57 (m, 5H), 1.53 (d, *J* = 7.1 Hz, 3H), 1.49 – 1.38 (m, 4H), 1.21 (dd, *J* = 6.4, 5.4 Hz, 6H), 1.01 – 0.90 (m, 27H), 0.89 (d, *J* = 5.9 Hz, 3H); ^{13}C NMR (201 MHz, D_2O) δ 180.48, 175.00, 174.24, 173.70, 173.58, 173.39, 172.33, 172.09, 171.88, 171.74, 171.71, 171.55, 170.93, 170.74, 104.16, 103.86, 103.83, 103.71, 102.95, 101.27, 101.00, 82.14, 80.43, 80.09, 76.61, 76.27, 75.31, 75.29, 74.91, 74.73, 74.63, 73.92, 73.77, 73.72, 73.53, 72.84, 72.73, 72.29, 71.81,70.02, 69.79, 69.57, 68.47, 68.05, 67.02, 62.47, 62.07, 61.07, 61.00, 60.65, 59.66, 59.51, 59.19, 58.94, 57.25, 53.76, 53.46, 53.33, 52.47, 51.50, 51.00, 50.07, 48.84, 47.88, 42.82, 42.41, 39.14, 32.74, 31.03, 30.39, 30.03, 29.98, 29.32, 27.39, 27.26, 27.07, 26.75, 26.58, 26.26, 26.18, 24.77, 24.37, 24.31, 22.42, 22.02, 21.90, 21.87, 20.92, 20.53, 18.89, 18.80, 18.42, 18.40, 17.86, 17.80, 17.76, 16.66. HRMS (ESI) m/z: $[M - 3H]^{3-}$ Calcd for Chemical Formula: $C_{159}H_{254}N_{31}O_{89}S$ 1351.2016; Found 1351.2030.

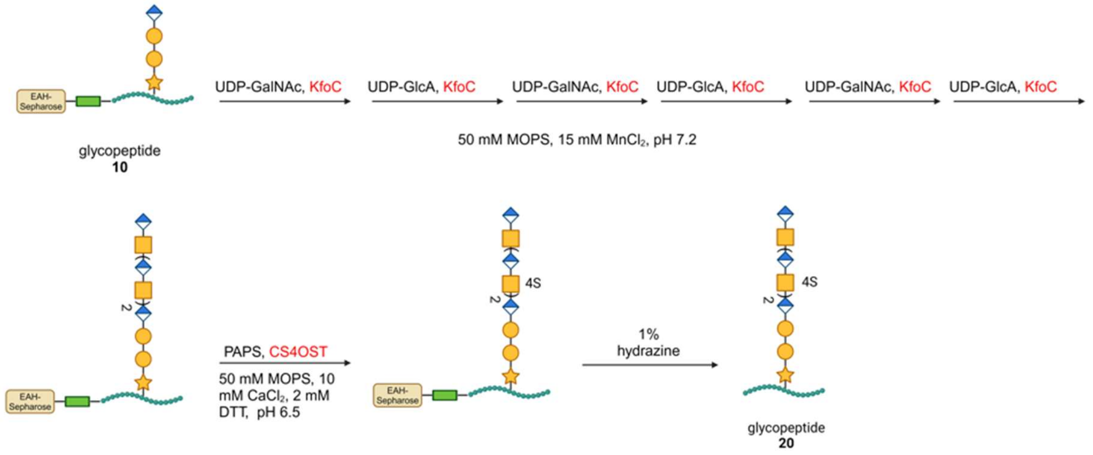

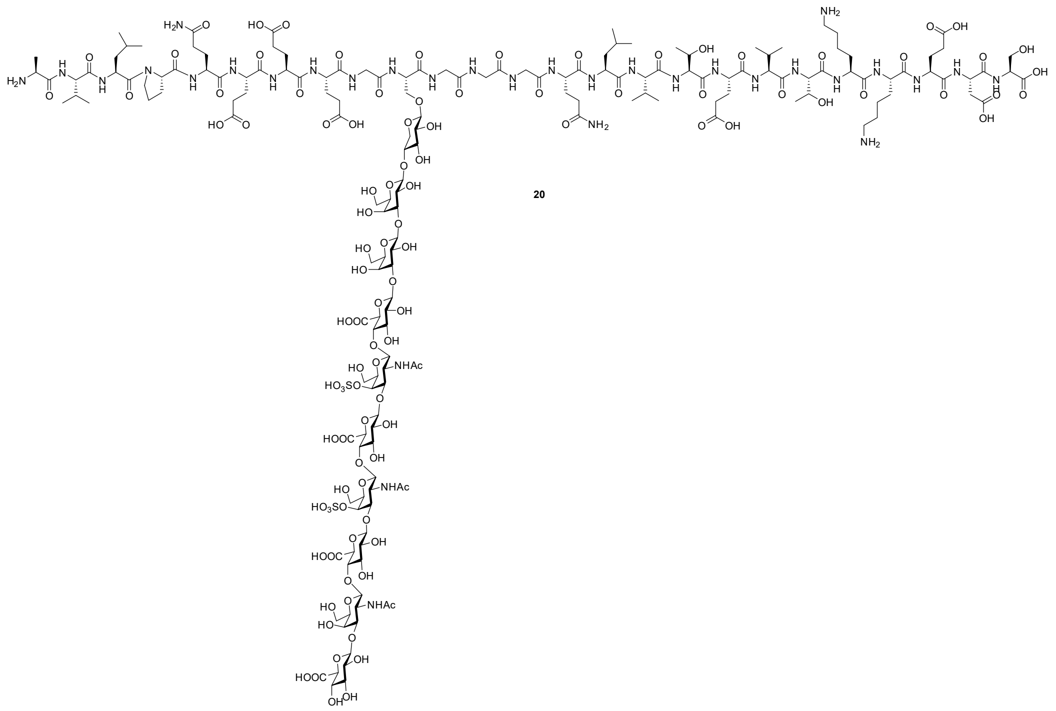

Glycopeptide **17** (1 mg, 0.22 μmol) was dissolved in 0.2 mL of CS4OST reaction buffer (50 mM MOPS, 10 mM $CaCl_2$, fresh 2 mM DDT, pH 6.5) containing CS4OST (50 μg), PAPS (0.25 mg, 0.5 μmol). The reaction mixture was incubated for 6 h at 37 ºC, then another 0.2 mL of reaction buffer with enzymes and PAPS was added to the mixture again and was incubated for another 6 h at 37 ºC. Upon completion, 0.4 mL of MeOH was added and the mixture was centrifuged under 10,000 g for 10 min and dried *in vacuo*. The mixture was purified by prep C18 HPLC (0-50% water, 50 mM ammonium formate/acetonitrile, 5% [0 min]-5% [2 min]-50% [20 min]-100% [25 min], 17 min/25 min)) to obtain a white amorphous compound **20** (0.56 mg) in 54 % yield. ^{1}H NMR (800 MHz, D_2O) δ 4.72 – 4.65 (m, 4H), 4.61 – 4.27 (m, 25H), 4.25 (d, J = 5.9 Hz, 2H), 4.23 – 4.08 (m, 11H), 4.08 – 3.95 (m, 10H), 3.93 – 3.55 (m, 36H), 3.52 – 3.44 (m, 3H), 3.44 – 3.36 (m, 3H), 3.35 – 3.30 (m, 2H), 3.02 (dt, J = 12.5, 7.5 Hz, 4H), 2.80 (dd, J = 16.2, 4.7 Hz, 1H), 2.69 (dd, J = 16.2, 4.6

Hz, 1H), 2.47 – 2.29 (m, 15H), 2.17 – 1.92 (m, 29H), 1.88 – 1.81 (m, 2H), 1.81 – 1.74 (m, 2H), 1.73 – 1.64 (m, 4H), 1.68 – 1.57 (m, 4H), 1.53 (d, J = 7.1 Hz, 2H), 1.50 – 1.38 (m , 2H), 1.38 – 1.25 (m, 5H), 1.21 (dd, J = 11.6, 6.4 Hz, 6H), 1.03 – 0.91 (m, 27H), 0.89 (d, J = 5.9 Hz, 3H); ^{13}C NMR (201 MHz, D_2O) δ 175.08, 173.36, 173.25, 172.40, 172.07, 171.97, 171.54, 171.43, 170.68, 104.23, 103.94, 103.87, 103.04, 101.20, 101.00, 82.81, 82.16, 80.55, 80.39, 76.52, 76.36, 75.56, 75.39, 74.91, 74.75, 73.78, 72.98, 72.82, 72.33, 71.85, 70.08, 69.92, 69.59, 68.47, 67.98, 67.18, 67.01, 62.18, 61.05, 60.73, 59.76, 59.28, 58.96, 57.35, 53.80, 53.48, 52.51, 51.54, 51.22, 50.09, 48.97, 47.84, 42.68, 42.52, 39.62, 39.15, 32.69, 31.08, 30.43, 30.11, 24.79, 27.37, 27.05, 26.24, 24. 47, 24.31, 22.54, 22.03, 20.98, 20.60, 20.12, 18.99, 18.35, 17.86, 17.06, 16.74. HRMS (ESI) m/z: $[M -3H]^{3-}$ Calcd for Chemical Formula: $C_{173}H_{275}N_{32}O_{103}S_2$ 1504.2234; Found 1504.2235.

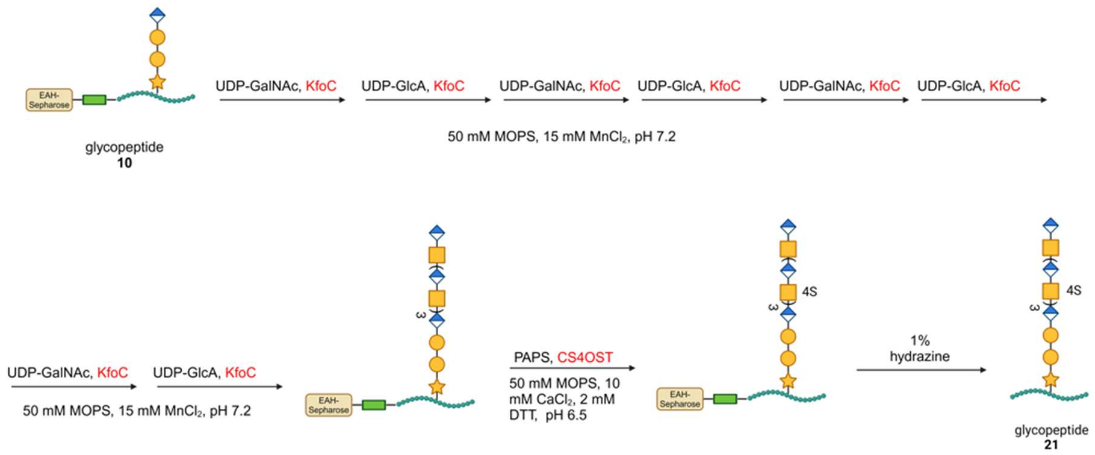

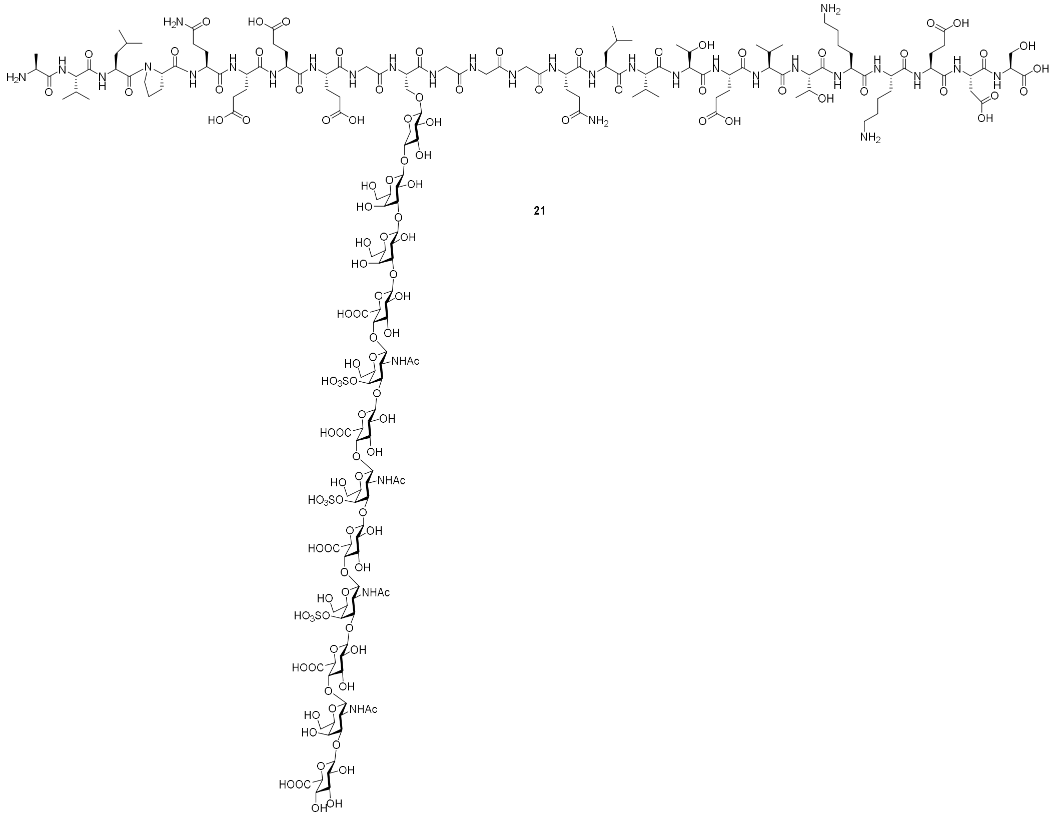

21

Glycopeptide **18** (1 mg, 0.21 μmol) was dissolved in 0.2 mL of CS4OST reaction buffer (50 mM MOPS, 10 mM $CaCl_2$, fresh 2 mM DDT, pH 6.5) containing CS4OST (50 μg) and donor PAPS (0.25 mg, 0.5 μmol). The reaction mixture was incubated for 6 h at 37 ºC, then another 0.2 mL of reaction buffer with enzymes and PAPS was added to the mixture again and was incubated for another 6 h at 37 ºC. Upon completion, 0.4 mL of MeOH was added and the mixture was centrifuged under 10,000 g for 10 min and dried *in vacuo*. The mixture was purified by prep C18 HPLC (0-50% water, 50 mM ammonium formate /acetonitrile, 5% [0 min]-5% [2 min]-50% [20 min]-100% [25 min], 15.5 min/25 min)) to obtain a white amorphous compound **21** (0.56 mg) in 52% yield. ^{1}H NMR (800 MHz, D_2O) δ 4.76 – 4.73 (m, 4H), 4.73 – 4.66 (m, 5H), 4.61 – 4.55 (br, 3H), 4.53 (d, *J* = 8.1 Hz, 2H), 4.52 – 4.26 (m, 21H), 4.26 – 4.22 (br, 1H),4.22 – 4.13 (m, 9H), 4.13 – 4.08 (br, 1H),4.08 – 3.96 (m, 15H), 3.91 – 3.54 (m, 44H), 3.50 – 3.43 (m, 3H), 3.43 – 3.35 (m, 4H), 3.35 – 3.30 (m, 2H), 3.03 – 2.98 (m, 4H), 2.81 (dd, *J* = 16.2, 4.7 Hz, 1H), 2.70 (dd, *J* = 16.2, 4.6 Hz, 1H), 2.48 – 2.29 (m, 15H), 2.17 – 1.91 (m, 31H), 1.88 – 1.80 (m, 3H), 1.80 – 1.74 (m, 2H), 1.73 – 1.67 (m, 5H), 1.67 – 1.54 (m, 5H), 1.52 (d, *J* = 7.1 Hz, 3H), 1.50 – 1.38 (m, 4H), 1.21 (dd, *J* = 11.0, 6.4 Hz, 6H), 1.00 – 0.91 (m, 27H), 0.89 (d, *J* = 5.9 Hz, 3H); ^{13}C NMR (201 MHz, D_2O) δ 178.70, 175.05, 173.65, 173.44, 173.33, 173.48, 172.37, 172.26, 172.07, 171.55, 171.36, 170.74, 104.17, 103.88, 103.82, 103.77, 103.71, 102.92, 101.32, 101.00, 100.84, 82.65, 82.16, 80.42, 76.46, 76.32, 75.57, 75.28, 74.93, 74.57, 73.77, 73.51, 72.83, 72.72, 72.27, 71.79, 70.03, 69.80, 68.69, 68.44, 68.07, 67.66, 66.99, 62.47, 62.01, 61.07, 60.99, 60.62, 59.71, 59.47, 59.20, 58.97, 57.14, 53.77, 53.57, 53.44, 53.34, 52.99, 52.49, 51.53, 51.32, 51.01, 50.08, 48.84, 47.89, 42.71, 42.55, 39.57, 39.15, 38.99, 37.85, 32.00, 31.03, 30.36, 29.99, 29.32, 26.96, 26.75, 26.57, 26.26, 26.19, 24.76, 24.37, 24.32, 22.50, 22.41, 22.03,

21.93, 21.90, 20.91, 20.54, 18.89, 18.82, 18.42, 18.40, 17.86, 17.82, 17.76, 16.67. HRMS (ESI) m/z: $[M - 3H]^{3-}$ Calcd for Chemical Formula: $C_{187}H_{296}N_{33}O_{117}S_{3}$ 1657.2471; Found 1657.2493.

CZE-FT-ICR MS fragmentation pattern of glycopeptides **19** and **20**

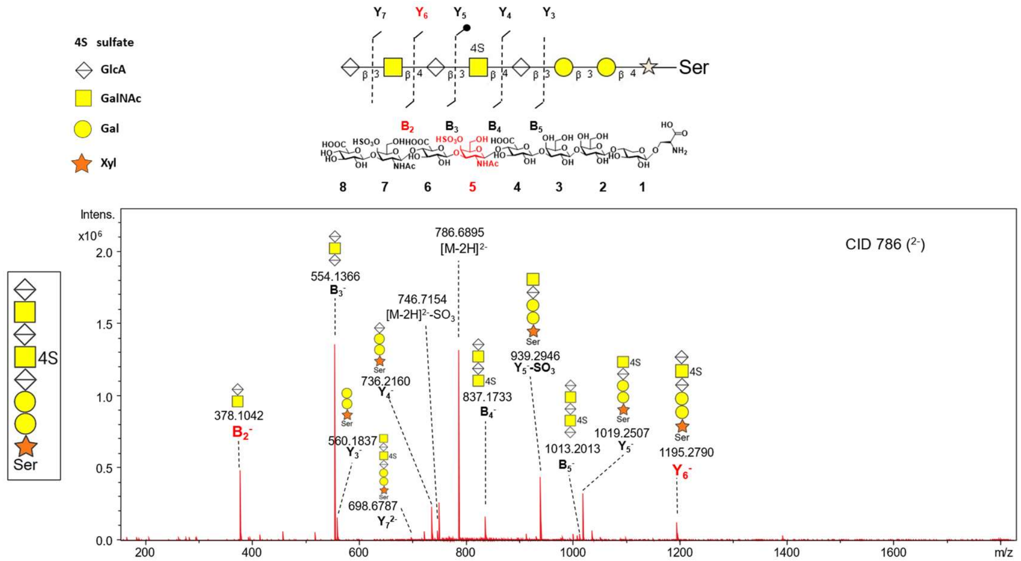

a.

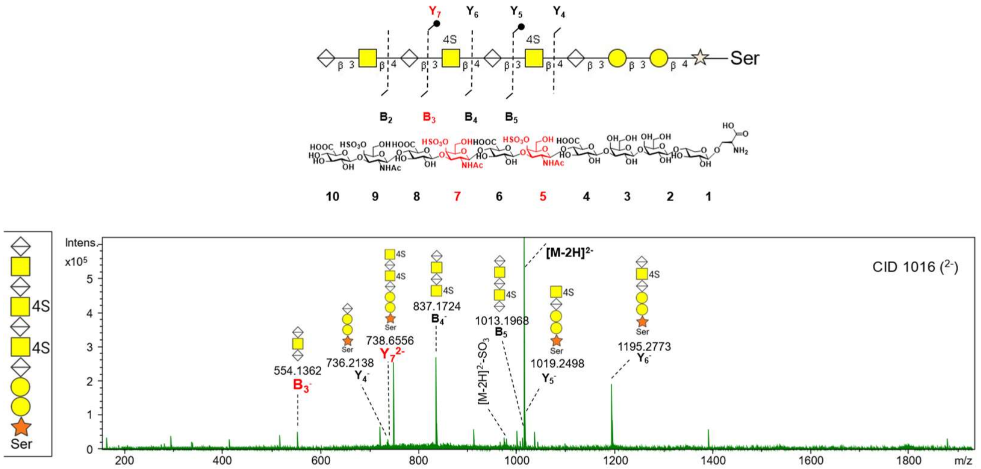

b.

Figure S1. The dashed lines on the structure indicate fragments with no sulfate loss observed. Black filled circles on the sequence indicate both fragment ions with sulfate loss and without sulfate loss were observed. The empty circle indicates a fragment ion with sulfate loss was observed. **a**. Sulfation pattern analysis of compound **19**, fragment ions B_2 and Y_6 indicate that sulfate is on GalNAc 5. **b**. Sulfation pattern analysis of compound **20**, fragment ions B_3 and Y_7 indicate that sulfate is on GalNAc 5 and GalNAc 7.

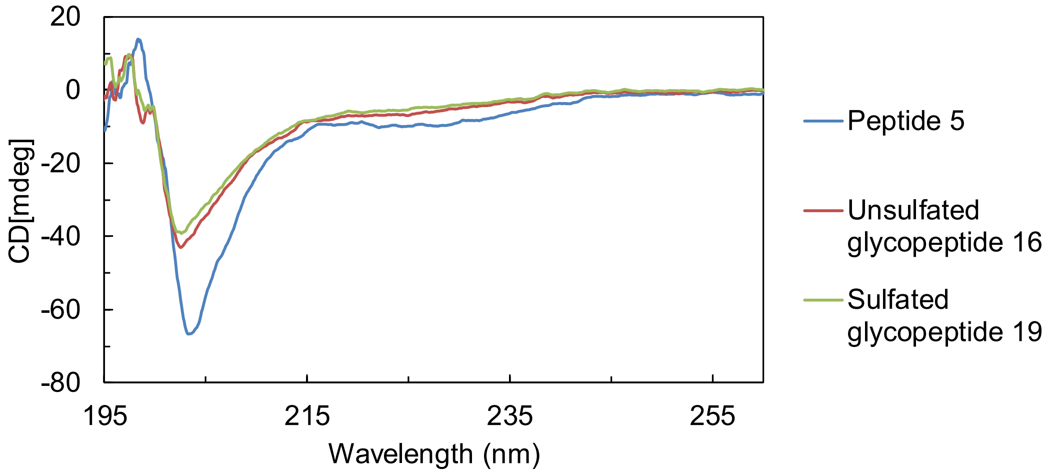

Figure S2. CD spectra of peptide **5**, unsulfated glycopeptide **16**, and sulfated glycopeptide **19** measured in PBS buffer. For all three compounds, the absorbance values were 1 at 210 nm. The CD spectra were most consistent with all compounds having random coil conformations.

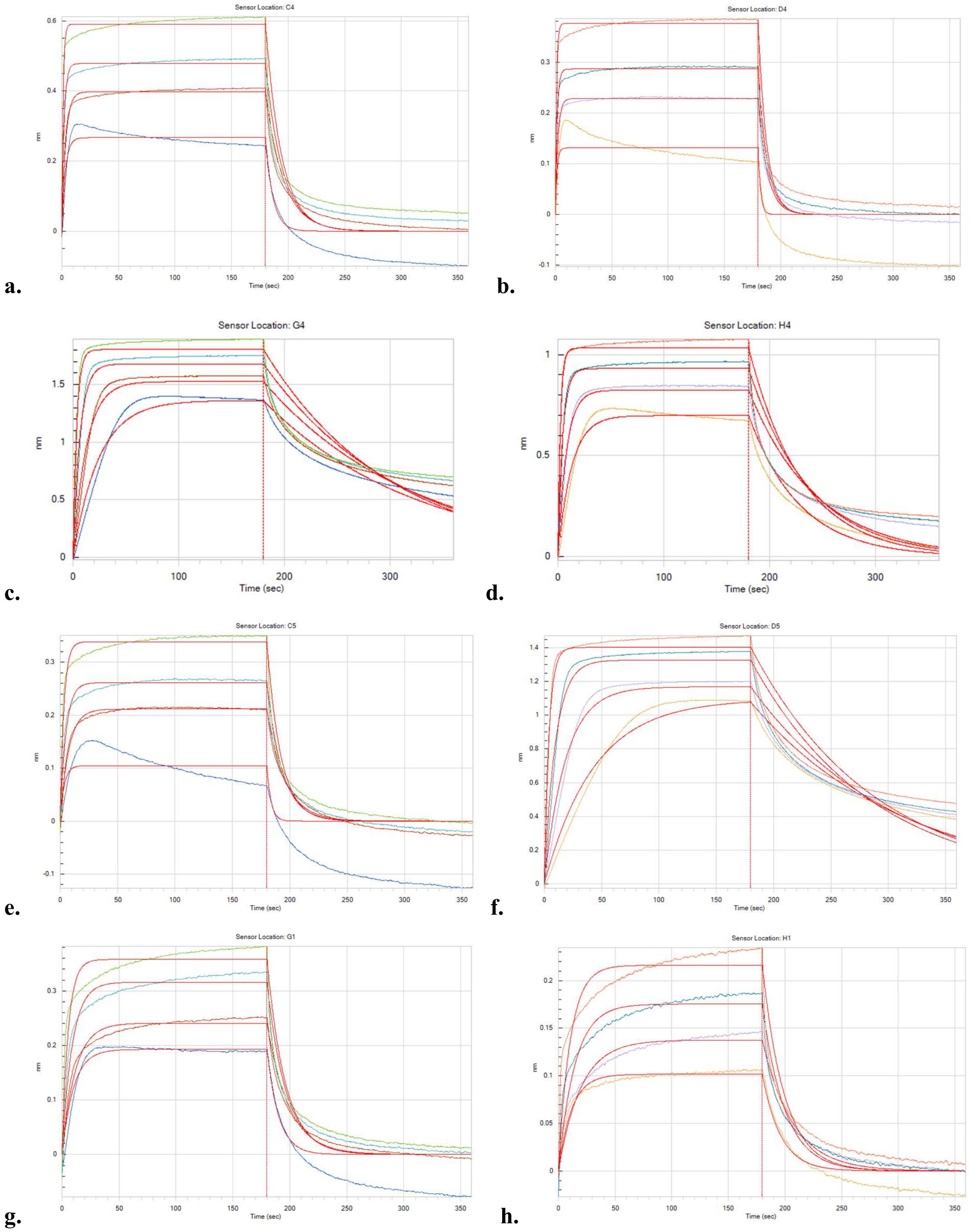

Figure S3. Binding of peptide and glycopeptides a) **5**, b) **10**, c) **18**, d) **19**, e) **20**, f) **21**, g) commercially available biotinylated 50 kDa CS, and h) 50 kDa CS-A to neutrophil Cathepsin G as measured by BLI (**a-h** respectively). The biotinylated compounds (50 nM) were immobilized on streptavidin coated biosensors, and human neutrophil Cathepsin G was captured on biosensors with four concentrations at 2000 nM, 1000 nM, 500 nM, 250 nM. Fitting curves were shown in red lines.

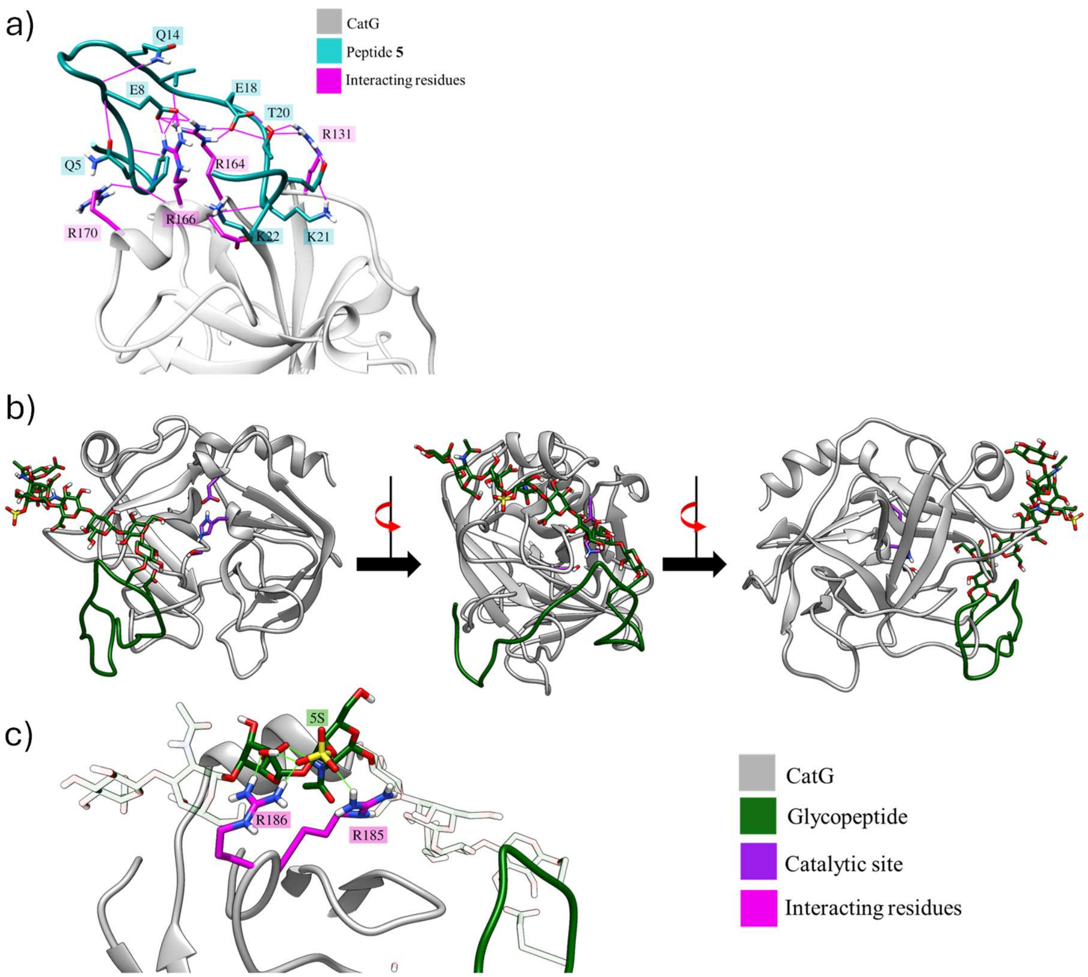

Figure S4. (a) The main interactions identified through docking between peptide **5** and CatG. The main binding was through surface arginine and lysine residues of CatG with the negatively charged glutamic acid residues E8 and E18 of the peptide. In addition, albeit weaker, there are interactions between the side chains or the backbone of CatG and the backbone of the peptide; (b) The highest scoring poses for glycopeptide **19** with CatG. (c) The direct hydrogen bond interactions of sulfate on GalNAc 5. The numbering of the residues follows the 1T32 PDB numbering.

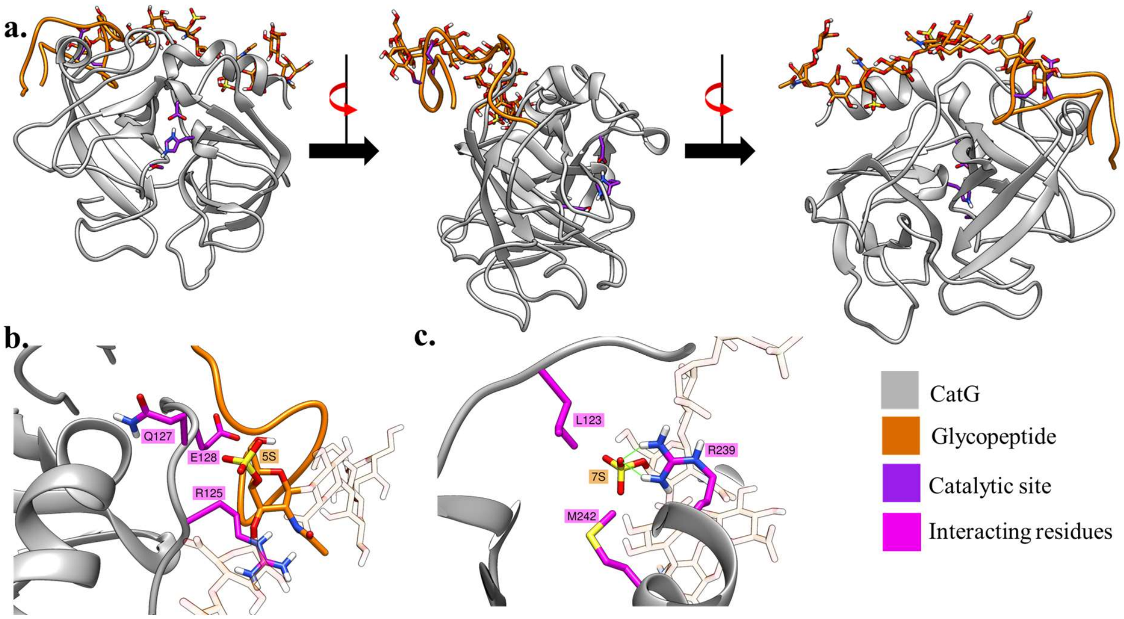

Figure S5. (a) The highest scoring poses for **20**. (b) The direct hydrogen bond interactions of sulfate on GalNAc 5. (c) The direct hydrogen bond interactions of sulfate on GalNAc 7. The numbering of the residues follows the 1T32 PDB numbering.

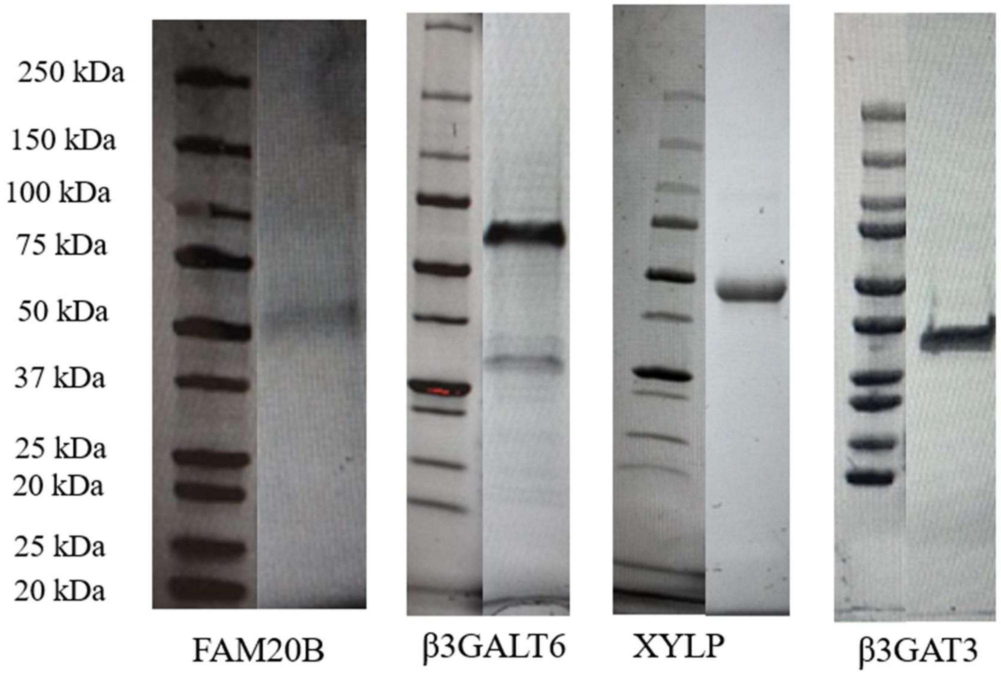

Figure S6. SDS PAGE gels of purified FAM20B, β3GALT6, XYLP, and β3GAT3.

Compound #	Binding Scores (*kcal mol^{-1}*)
**5**	-17.63
**18**	-21.34
**19**	-26.98
**20**	-26.88
**21**	-31.81

Table S1. The docking scores of the investigated glycopeptides, as described in the Methods section. Annotation of glycopeptide structures are shown.

References:

(1) Gao, J.; Lin, P.-H.; Nick, S. T.; Huang, J.; Tykesson, E.; Ellervik, U.; Li, L.; Huang, X. *Org. Lett.* **2021**, *23*, 1738-1741.

(2) Gao, J.; Lin, P.-h.; Nick, S. T.; Liu, K.; Yu, K.; Hohenester, E.; Huang, X. *Org. Biomol. Chem.* **2021**, *19*, 3374-3378.

(3) Koike, T.; Izumikawa, T.; Sato, B.; Kitagawa, H. *J. Biol. Chem.* **2014**, *289*, 6695-6708.

(4) Tone, Y.; Kitagawa, H.; Imiyab, K.; Okab, S.; Kawasaki, T.; Sugahara, K. *FEBS Lett.* **1999**, *459*, 415-420.

(5) Sanderson, P.; Stickney, M.; Leach, F. E., 3rd; Xia, Q.; Yu, Y.; Zhang, F.; Linhardt, R. J.; Amster, I. J. *J. Chromatogr. A* **2018**, *1545*, 75-83.

(6) Ceroni, A.; Maass, K.; Geyer, H.; Geyer, R.; Dell, A.; Haslam, S. M. *J. Proteome Res.* **2008**, *7*, 1650-1659.

NMR spectra, HPLC and LCMS

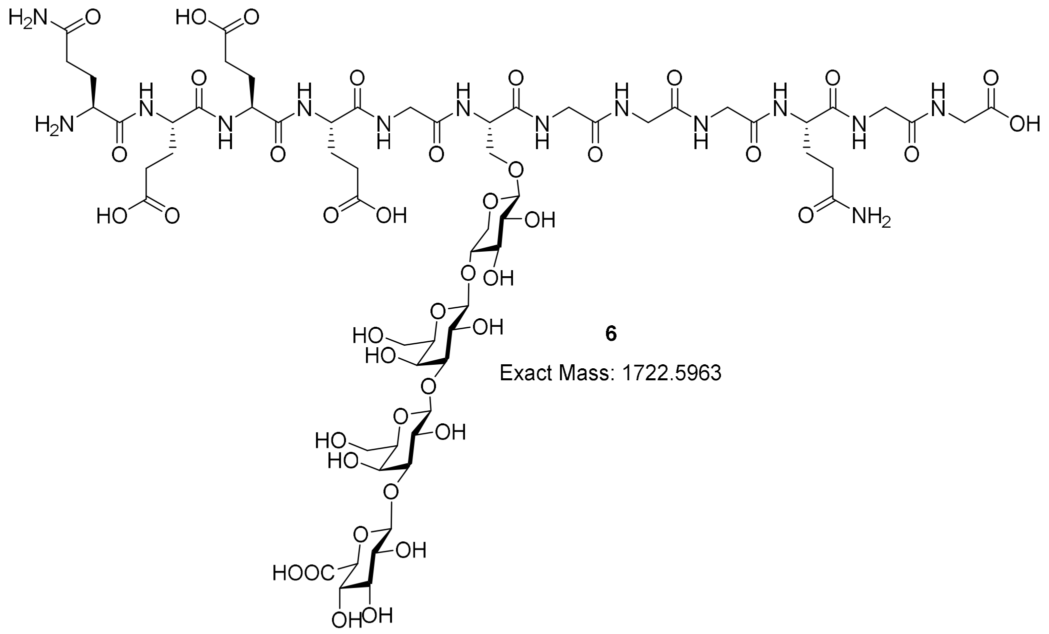

6

Exact Mass: 1722.5963

^{1}H NMR (800 MHz, D_2O)

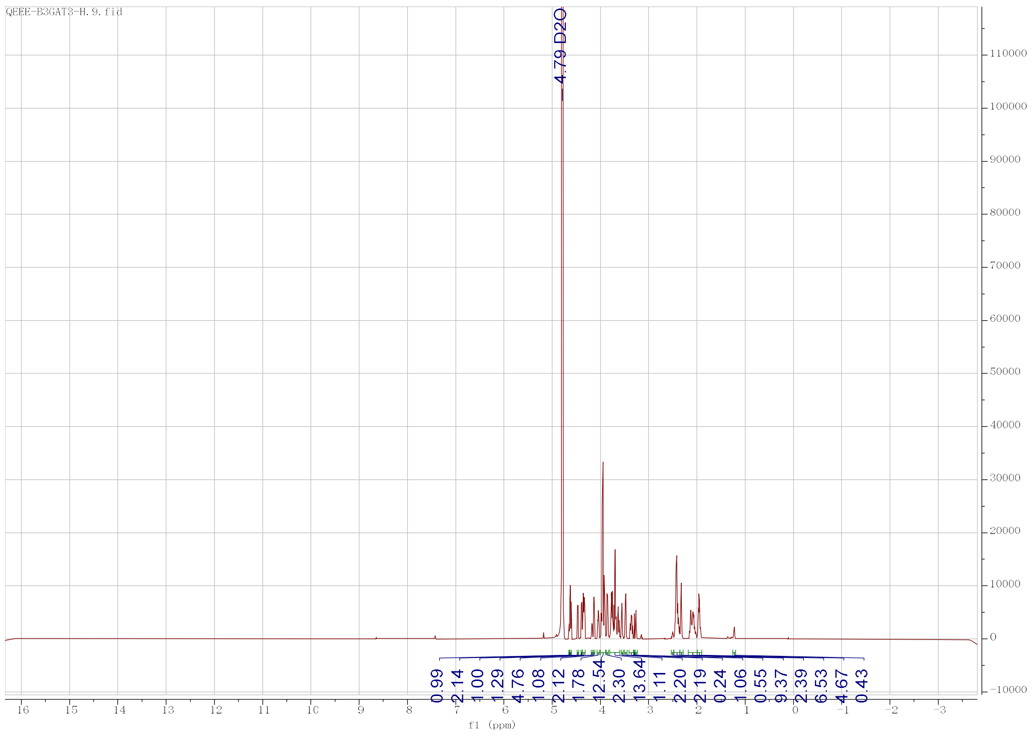

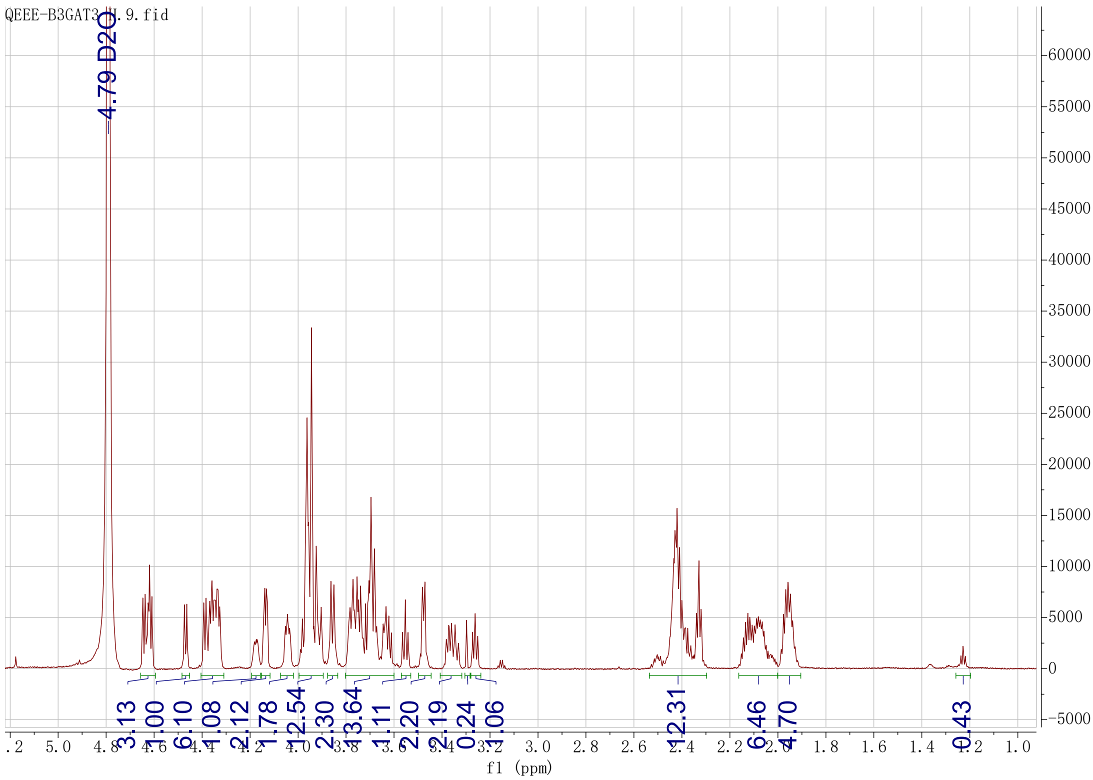

^{13}C NMR (201 MHz, D_2O)

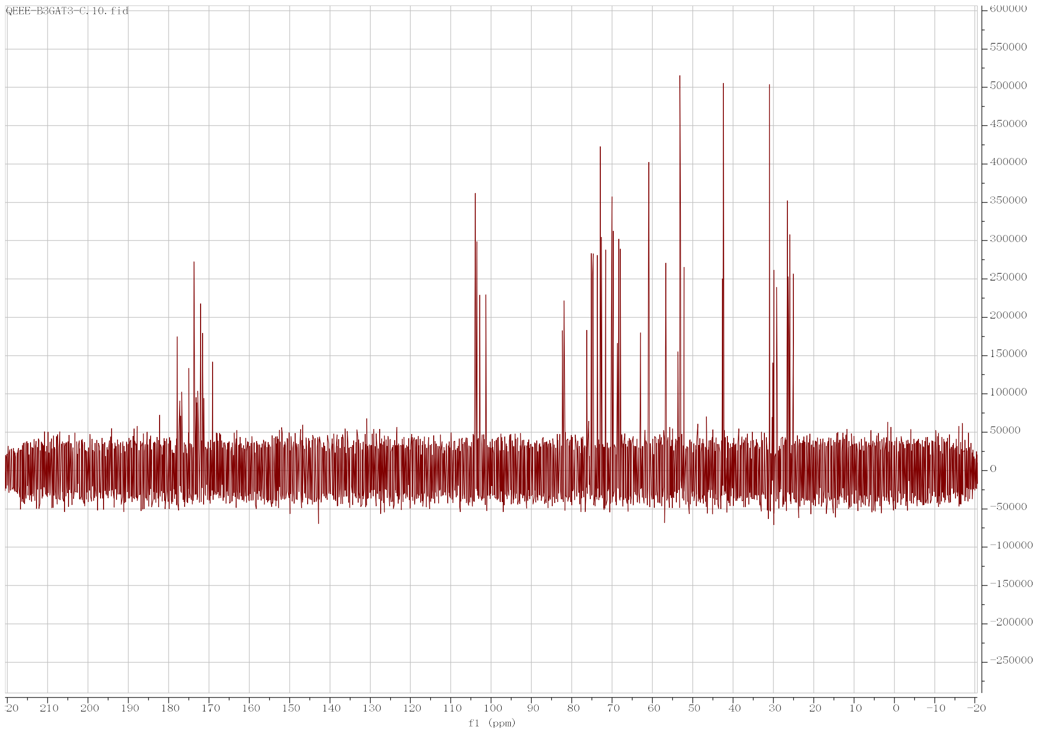

COSY (800 MHz, D_2O)

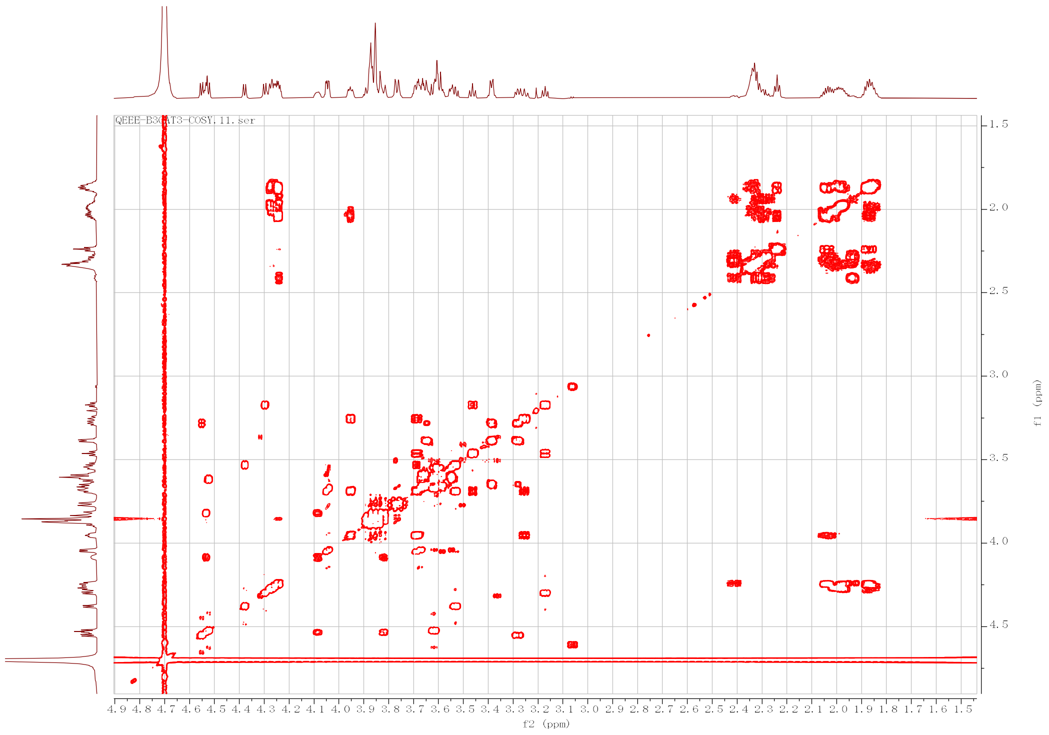

HSQC (800 MHz, D_2O)

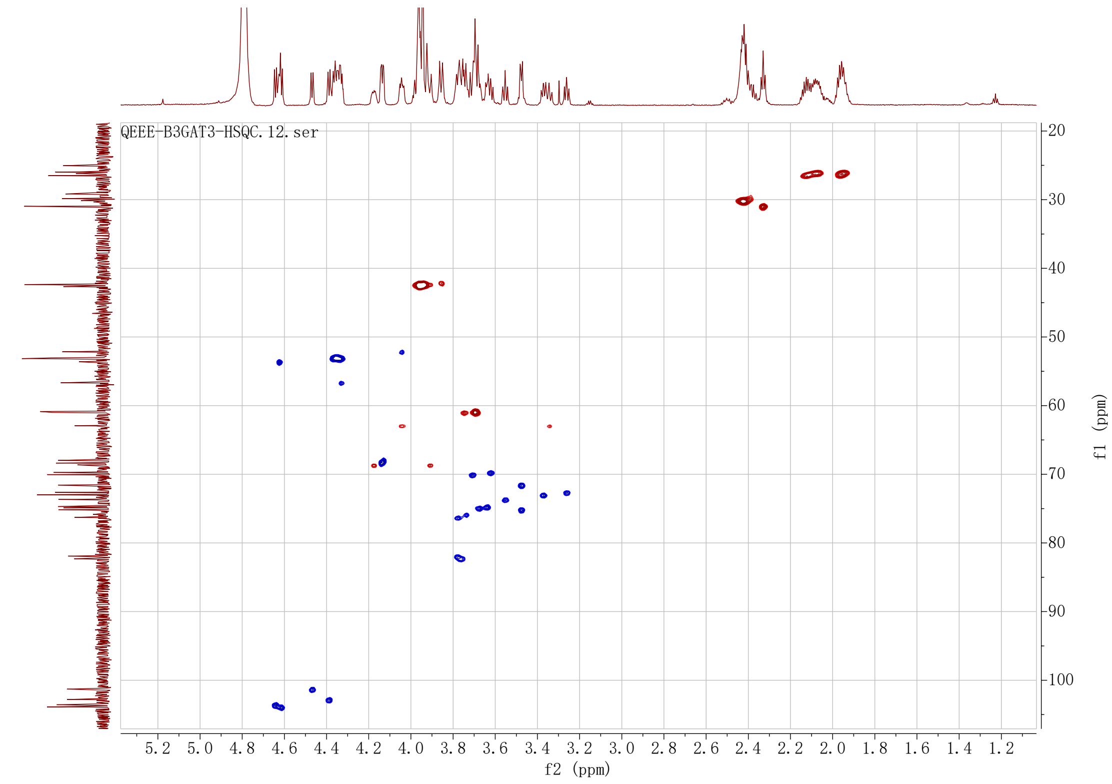

Coupled HSQC (800 MHz, D_2O)

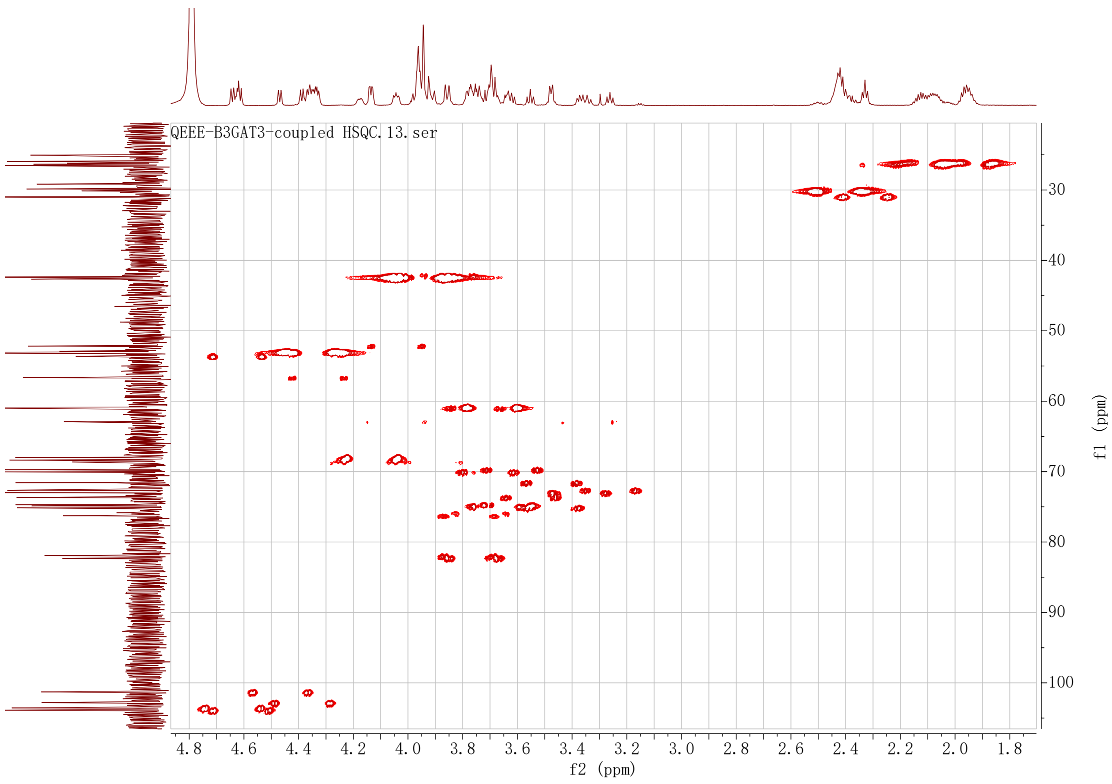

HMBC (800 MHz, D_2O)

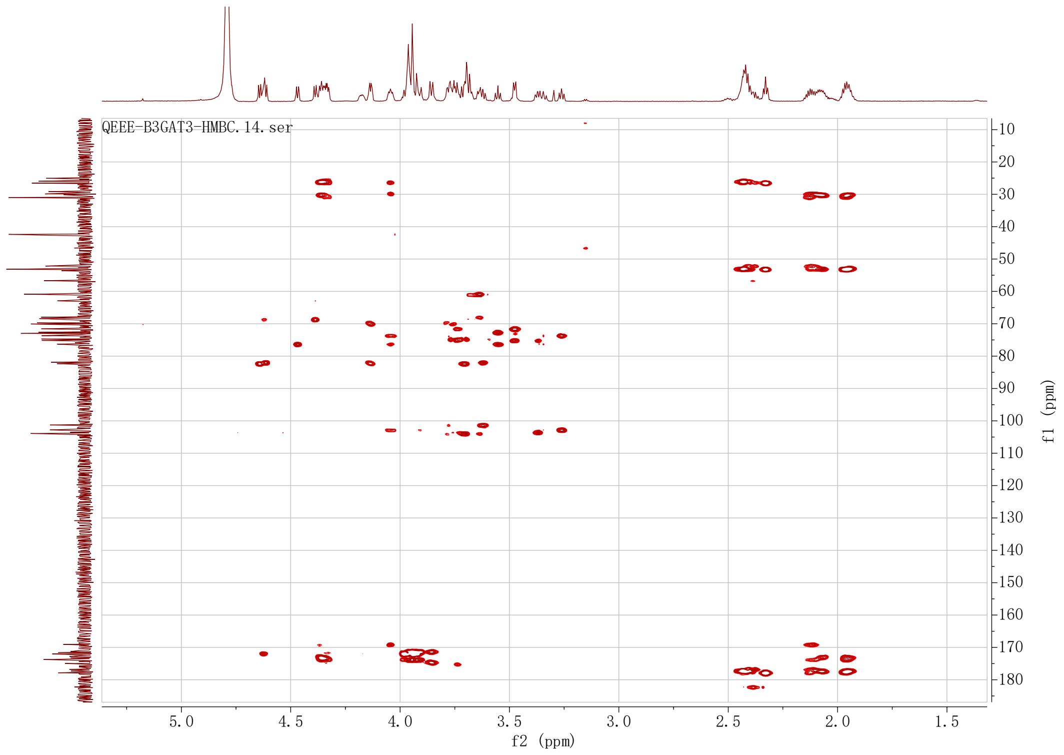

MS

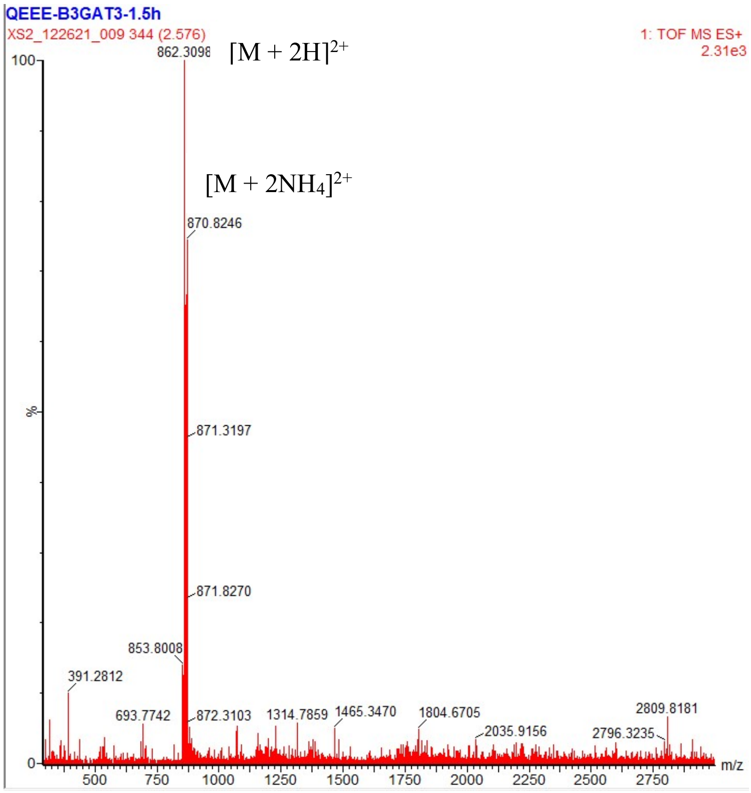
QEEE-B3GAT3-1.5h
XS2_122621_009 344 (2.576)
1: TOF MS ES+
2.31e3
862.3098
[M + 2H]2+
[M + 2NH4]2+
870.8246
871.3197
871.8270
853.8008
391.2812
693.7742
872.3103
1314.7859
1465.3470
1804.6705
2035.9156
2796.3235
2809.8181
100
%
0
500
750
1000
1250
1500
1750
2000
2250
2500
2750
m/z

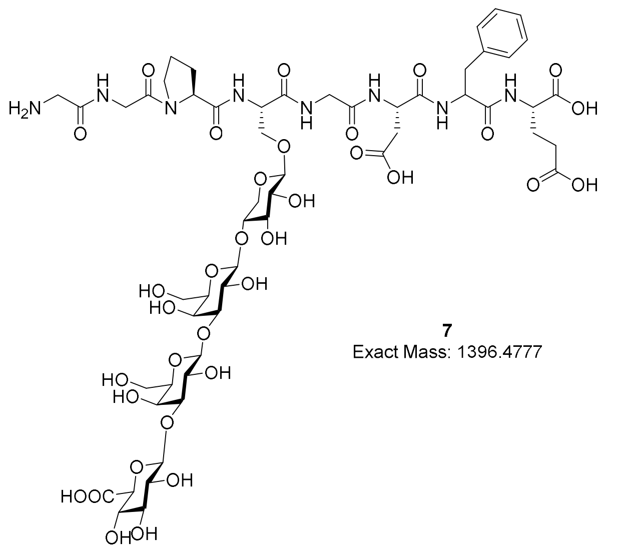

7
Exact Mass: 1396.4777

^{1}H NMR (800 MHz, D_2O)

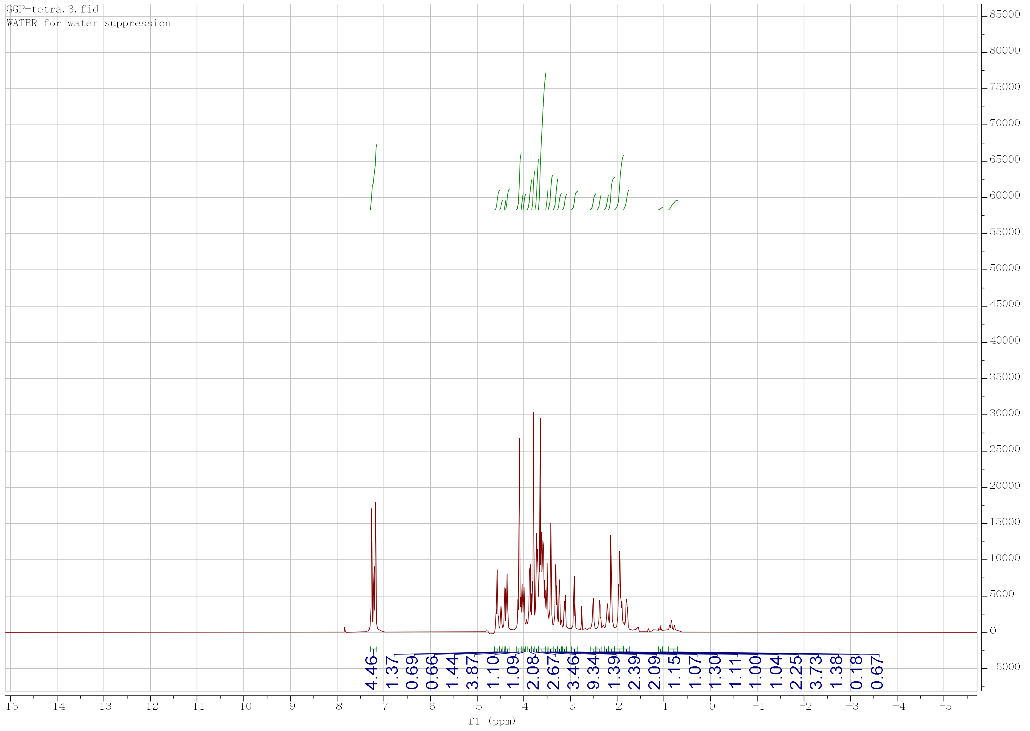

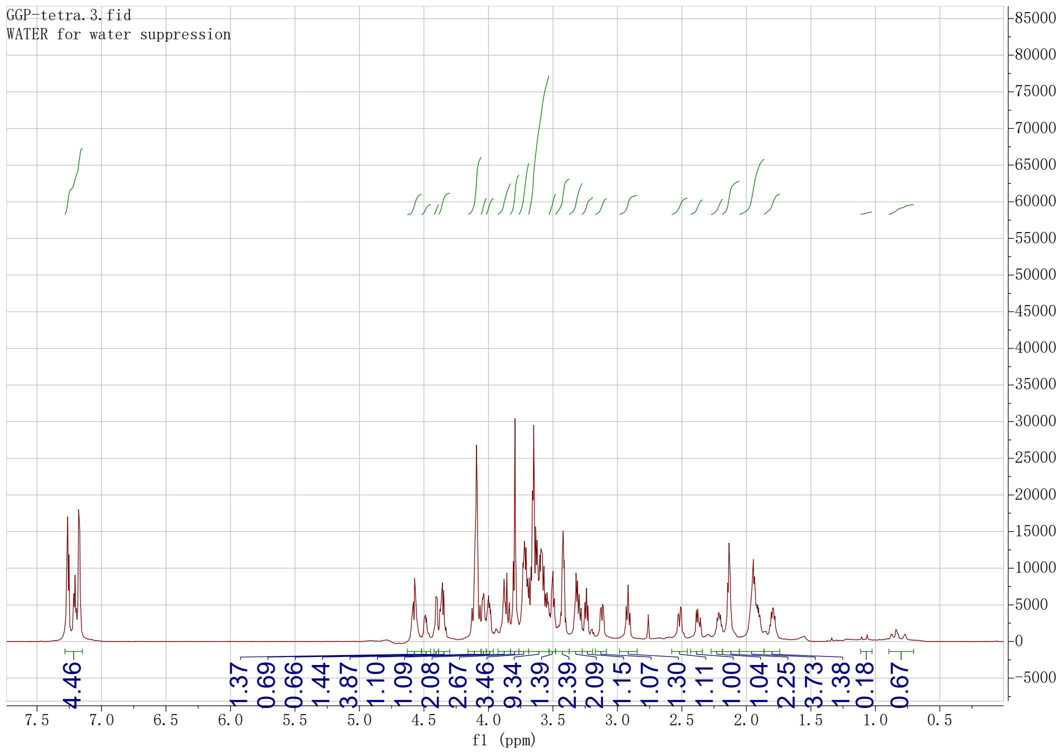
GGP-tetra.3.fid
WATER for water suppression
f1 (ppm)

^{13}C NMR (201 MHz, D_2O)

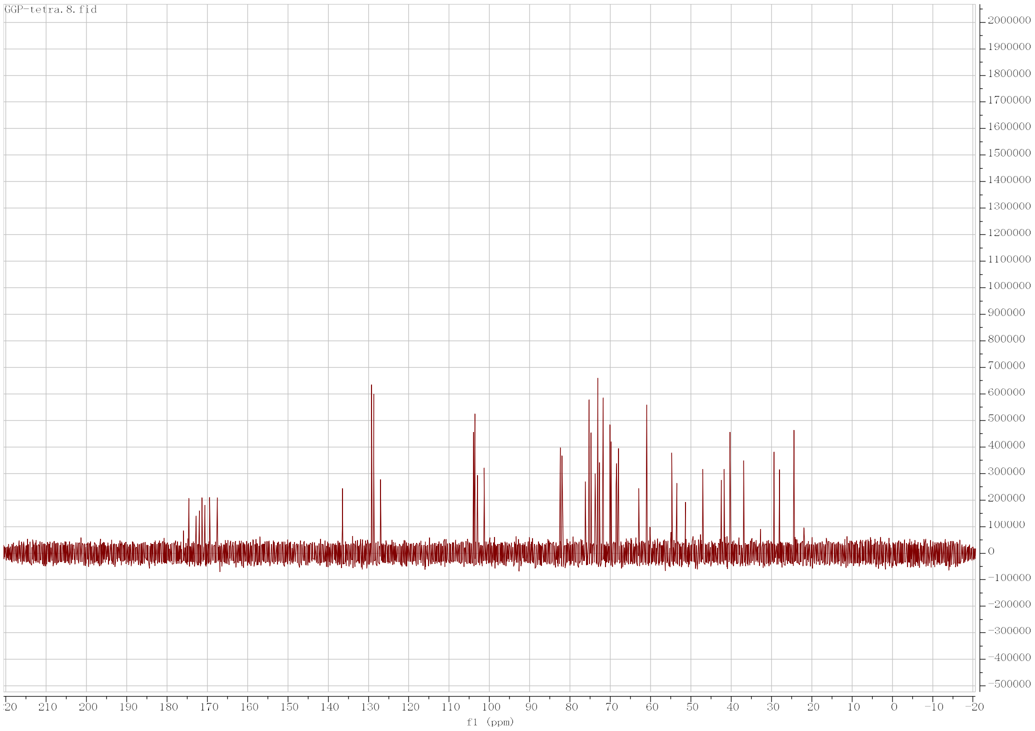

COSY (800 MHz, D_2O)

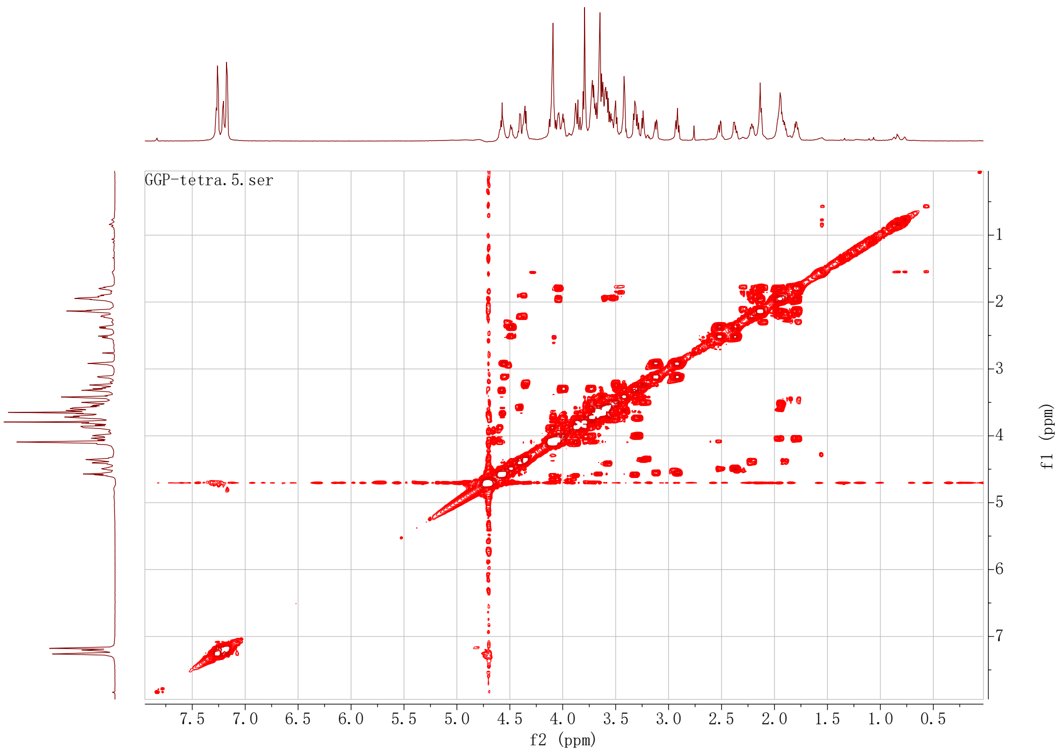

HSQC (800 MHz, D_2O)

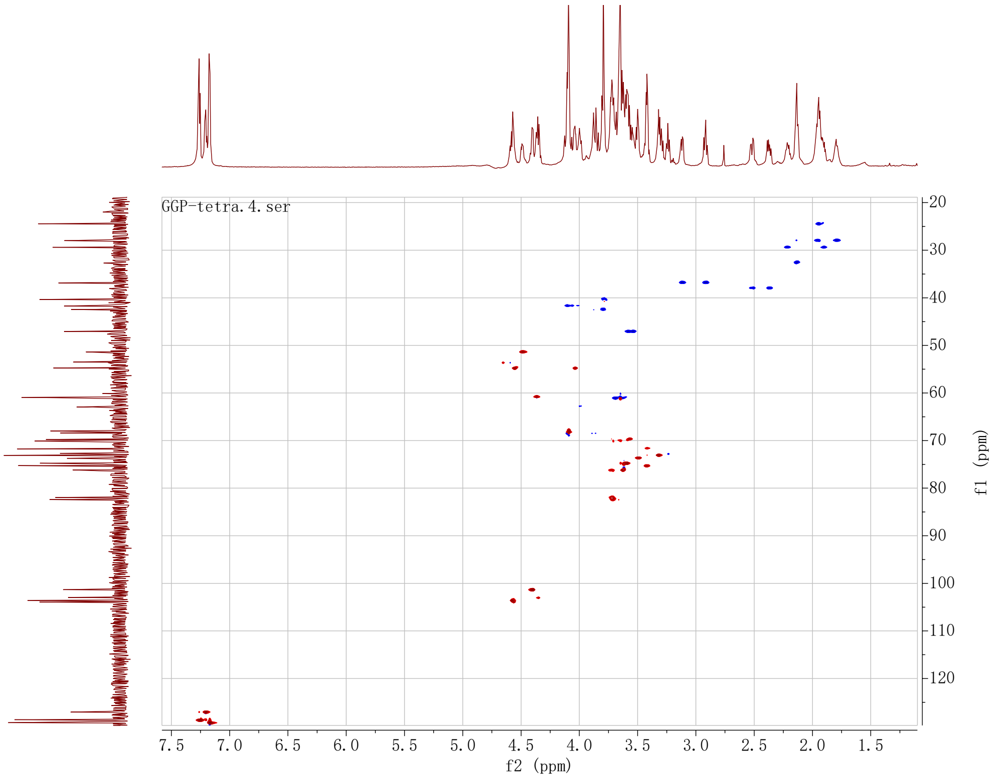

Coupled HSQC (800 MHz, D_2O)

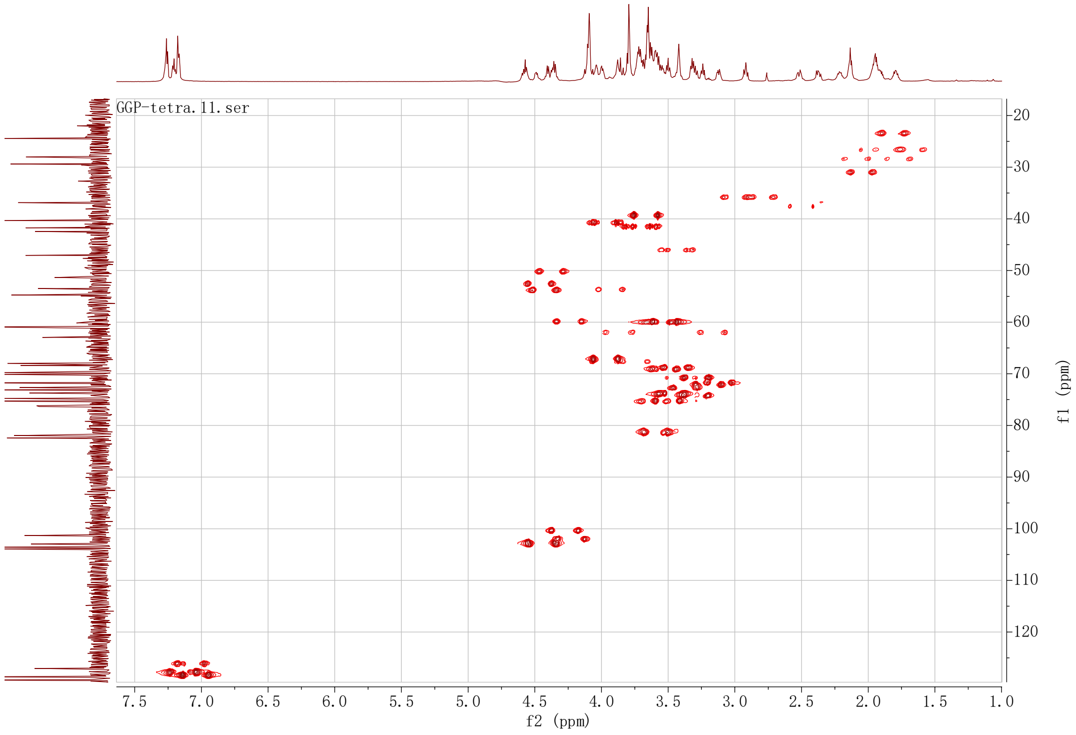

HMBC (800 MHz, D_2O)

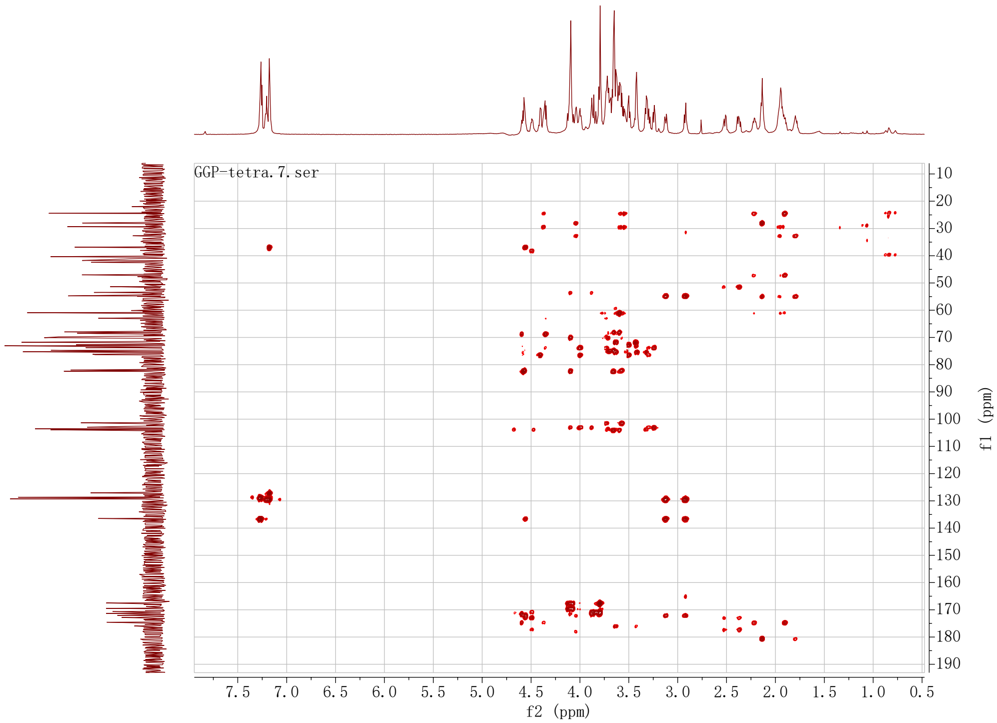

HPLC

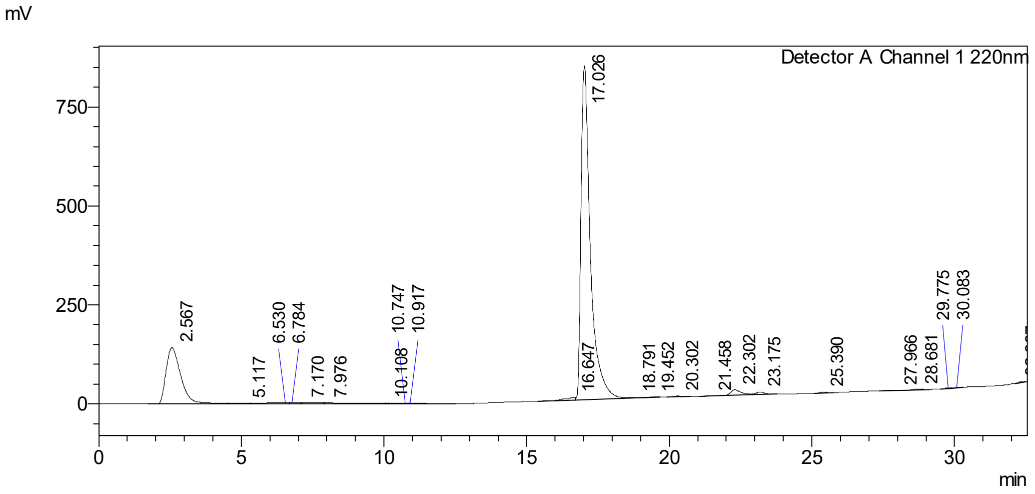

MS

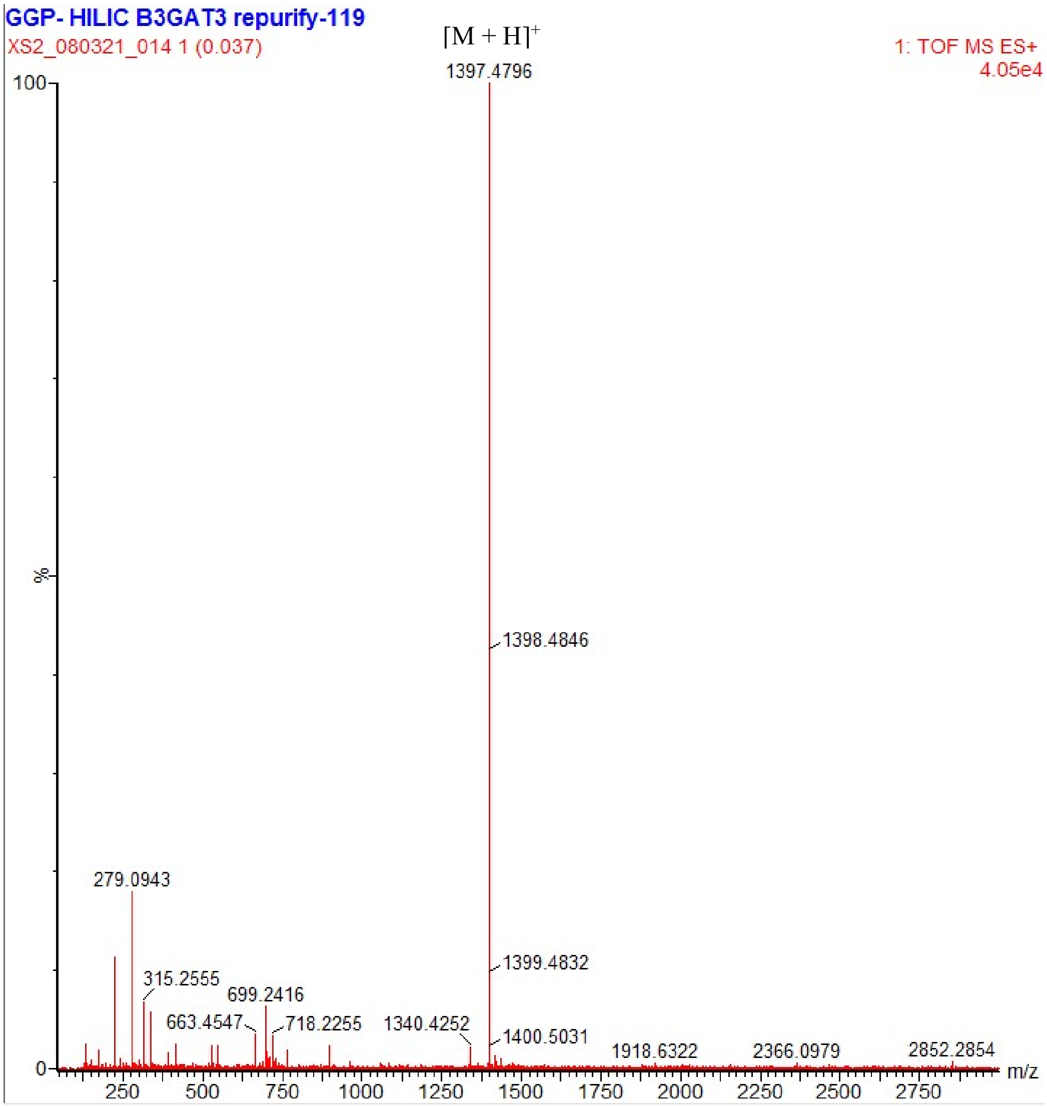
GGP- HILIC B3GAT3 repurify-119
XS2_080321_014 1 (0.037)
[M + H]+
1397.4796
1: TOF MS ES+
4.05e4
100
%
0
1398.4846
1399.4832
279.0943
315.2555
699.2416
663.4547
718.2255
1340.4252
1400.5031
1918.6322
2366.0979
2852.2854
250
500
750
1000
1250
1500
1750
2000
2250
2500
2750
m/z

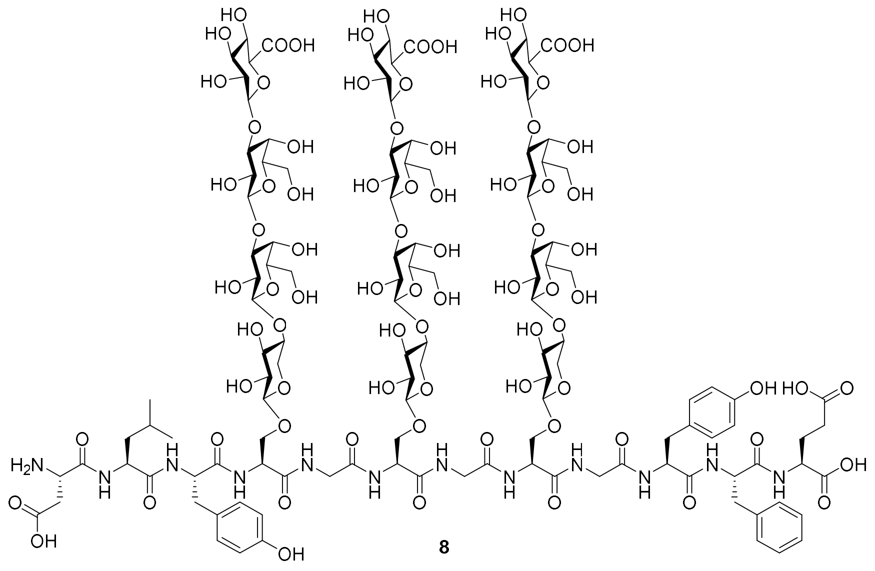

8

Exact Mass: 3177.0597

^{1}H NMR (800 MHz, D_2O)

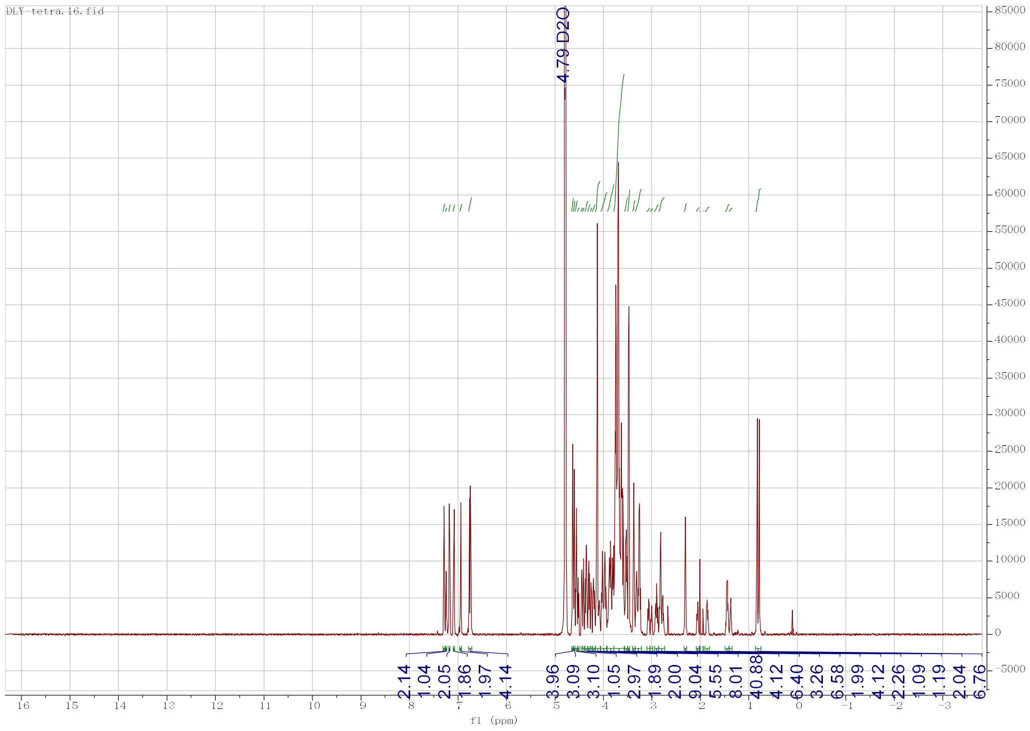

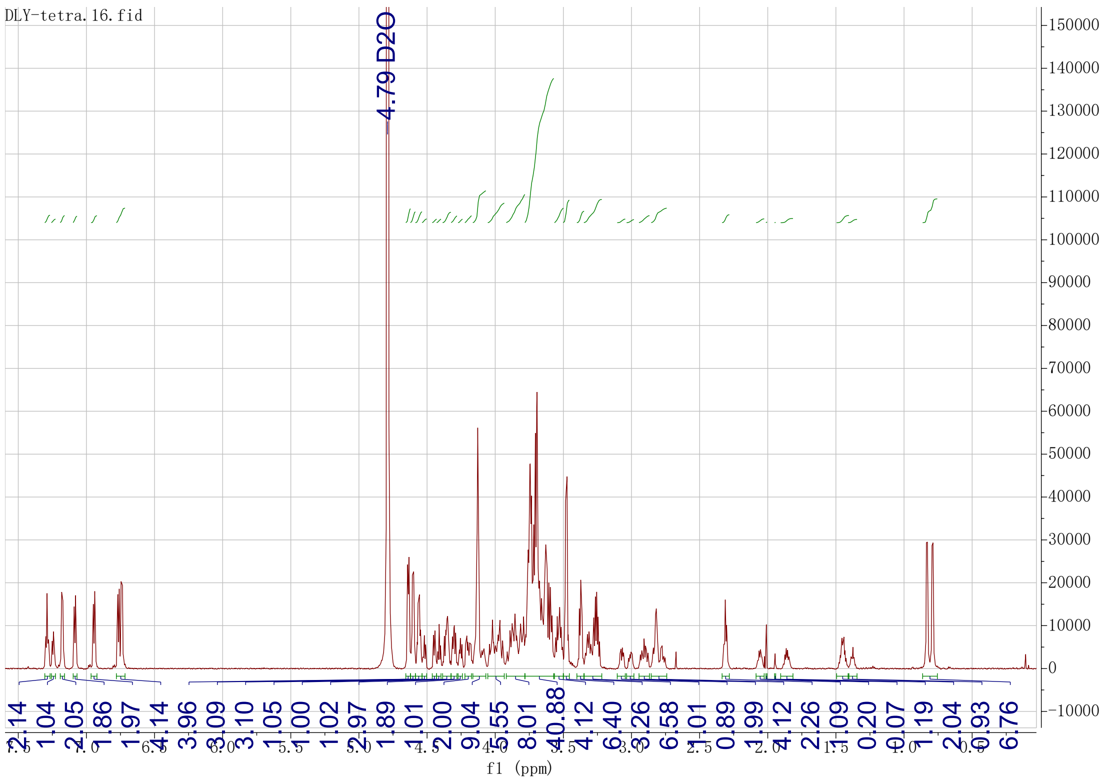

DLY-tetra.16.fid
4.79 D2O
f1 (ppm)

^{13}C NMR (201 MHz, D_2O)

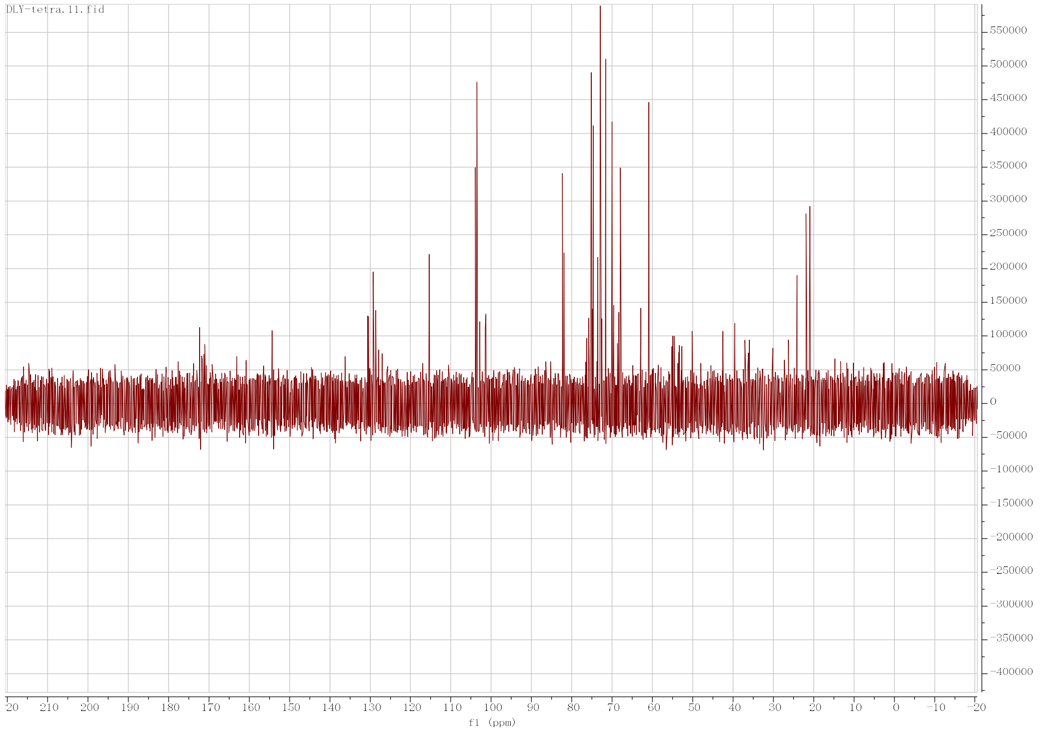

COSY (800 MHz, D_2O)

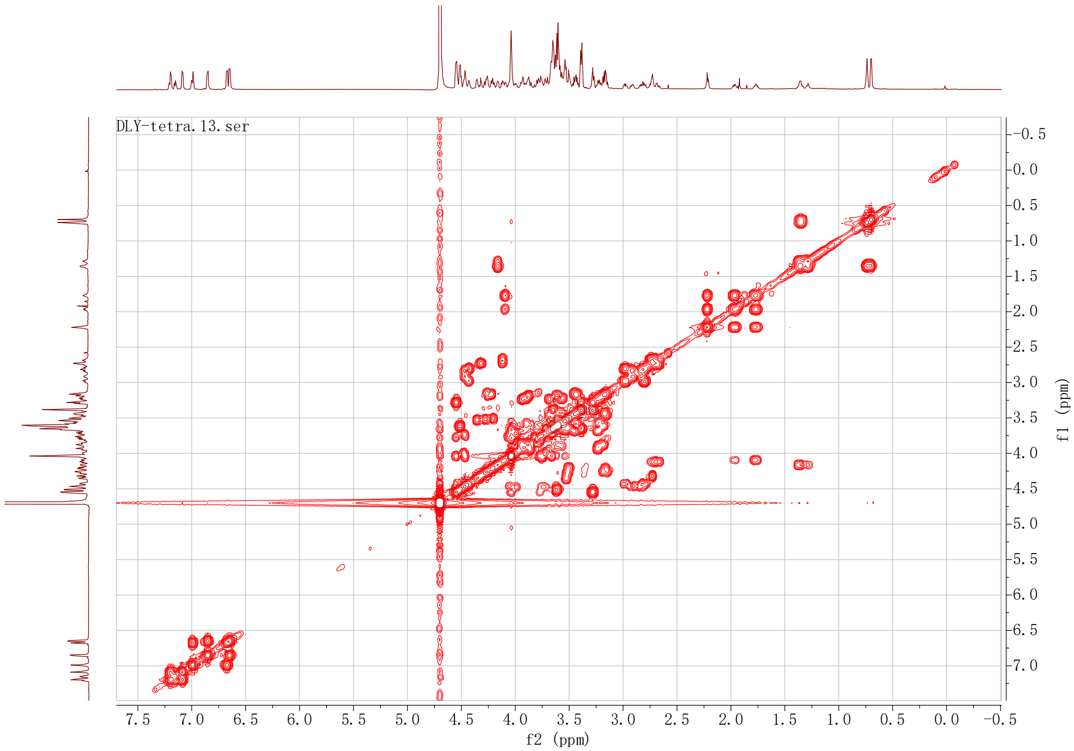

HSQC (800 MHz, D_2O)

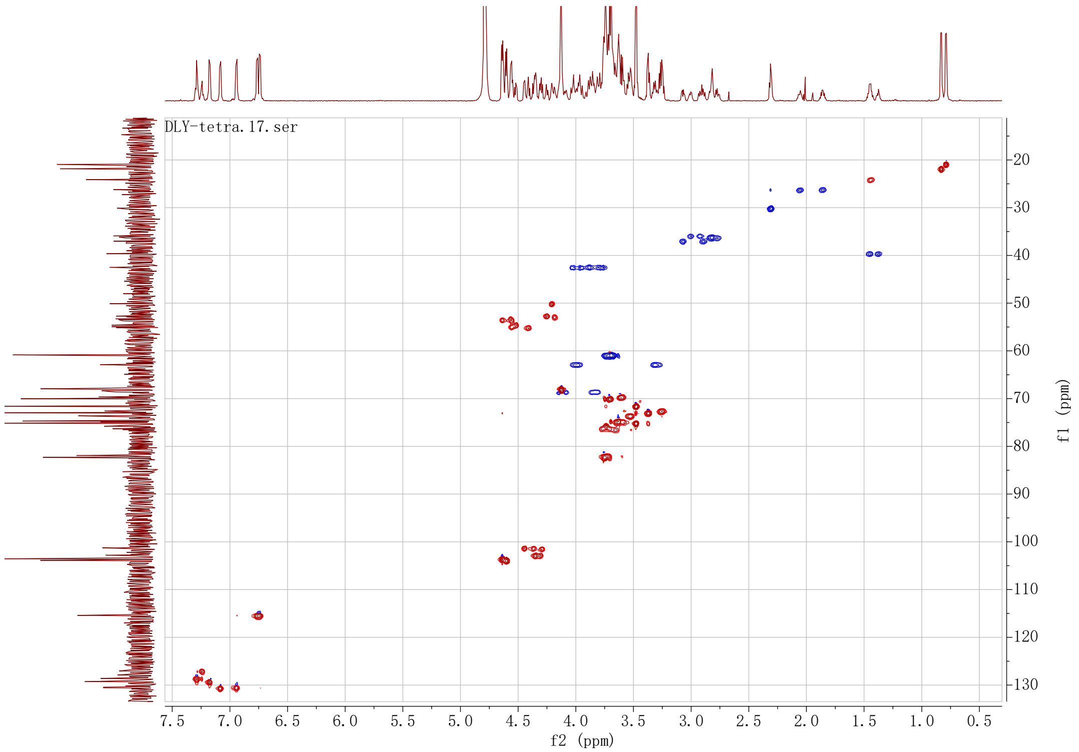

Coupled HSQC (800 MHz, D_2O)

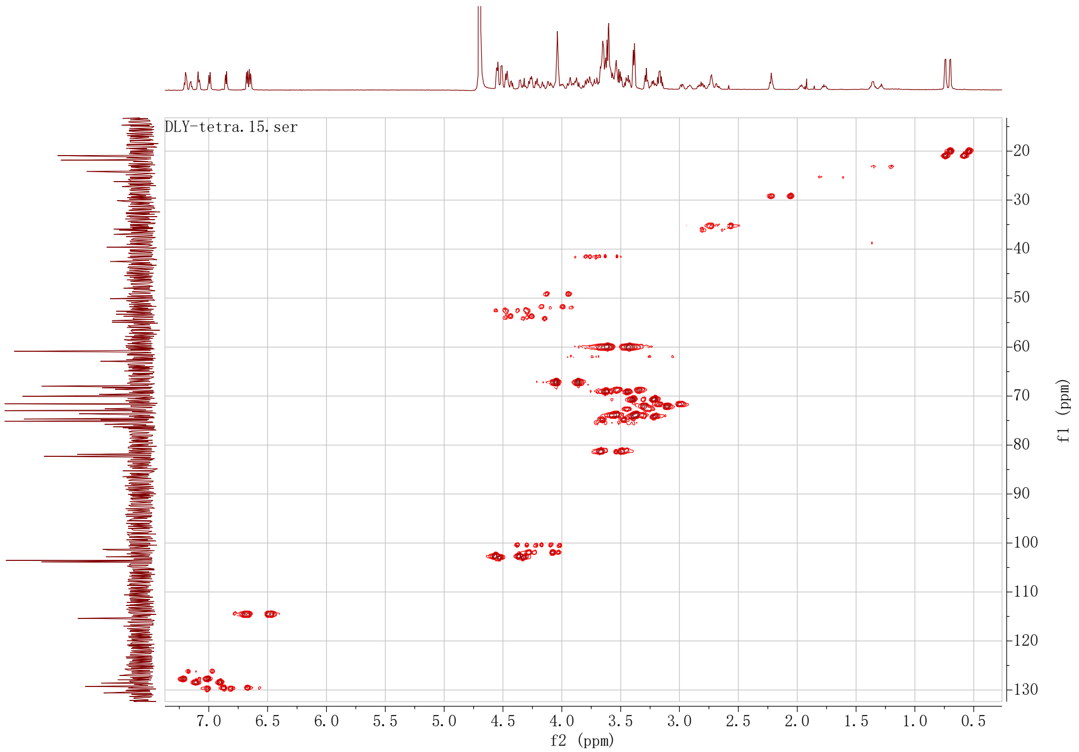

HMBC (800 MHz, D_2O)

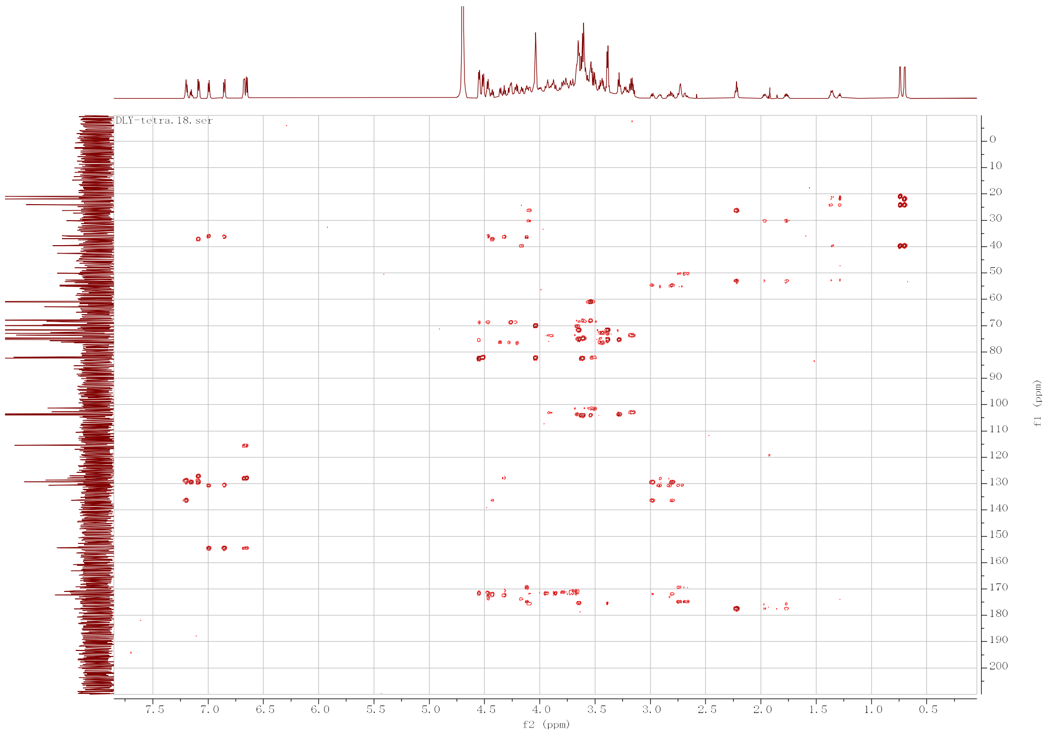

HPLC

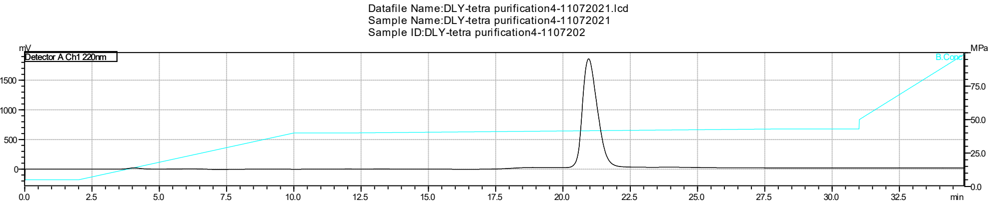

MS

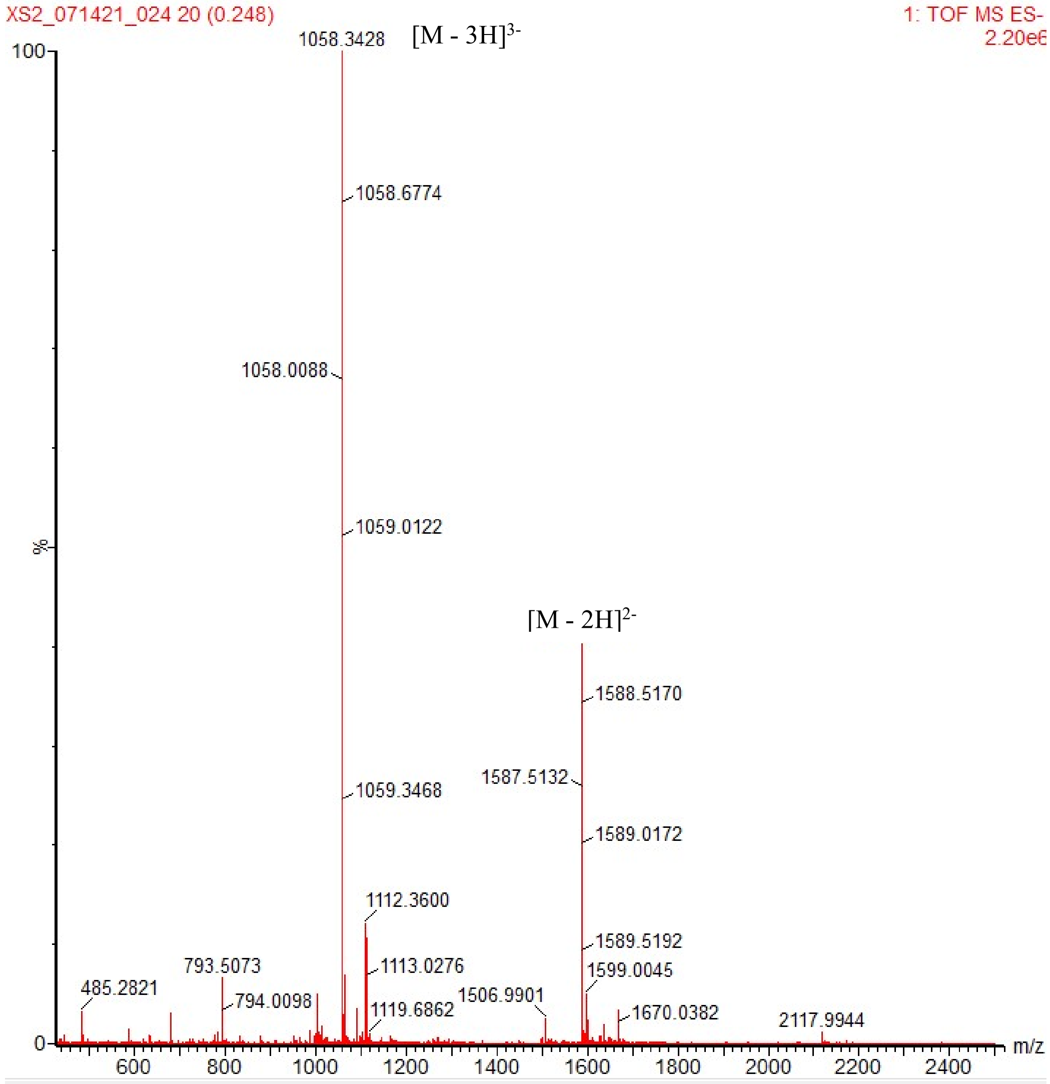
XS2_071421_024 20 (0.248)
1: TOF MS ES-
2.20e6
1058.3428
[M - 3H]$^{3-}$
1058.6774
1058.0088
1059.0122
1059.3468
[M - 2H]$^{2-}$
1588.5170
1587.5132
1589.0172
1589.5192
1599.0045
1112.3600
1113.0276
1119.6862
1506.9901
1670.0382
2117.9944
793.5073
794.0098
485.2821
100
%
0
600
800
1000
1200
1400
1600
1800
2000
2200
2400
m/z

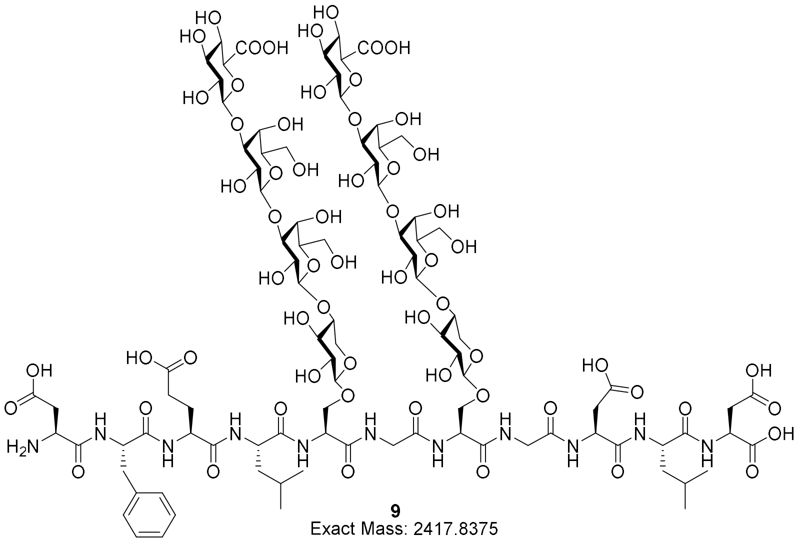

^{1}H NMR (800 MHz, D_2O)

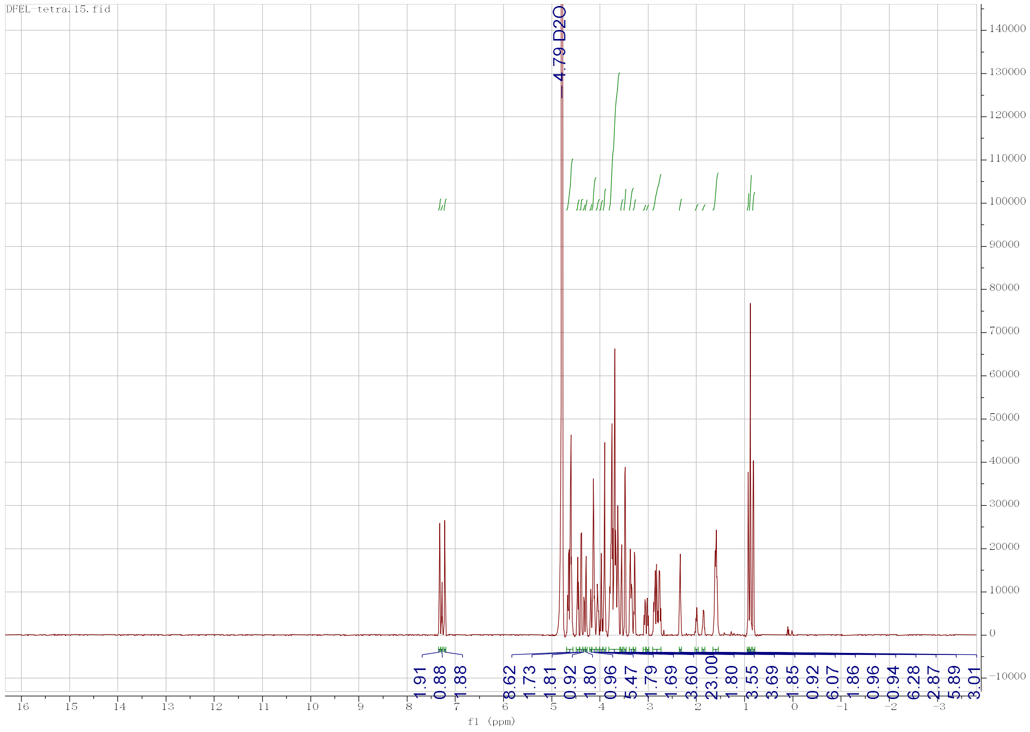

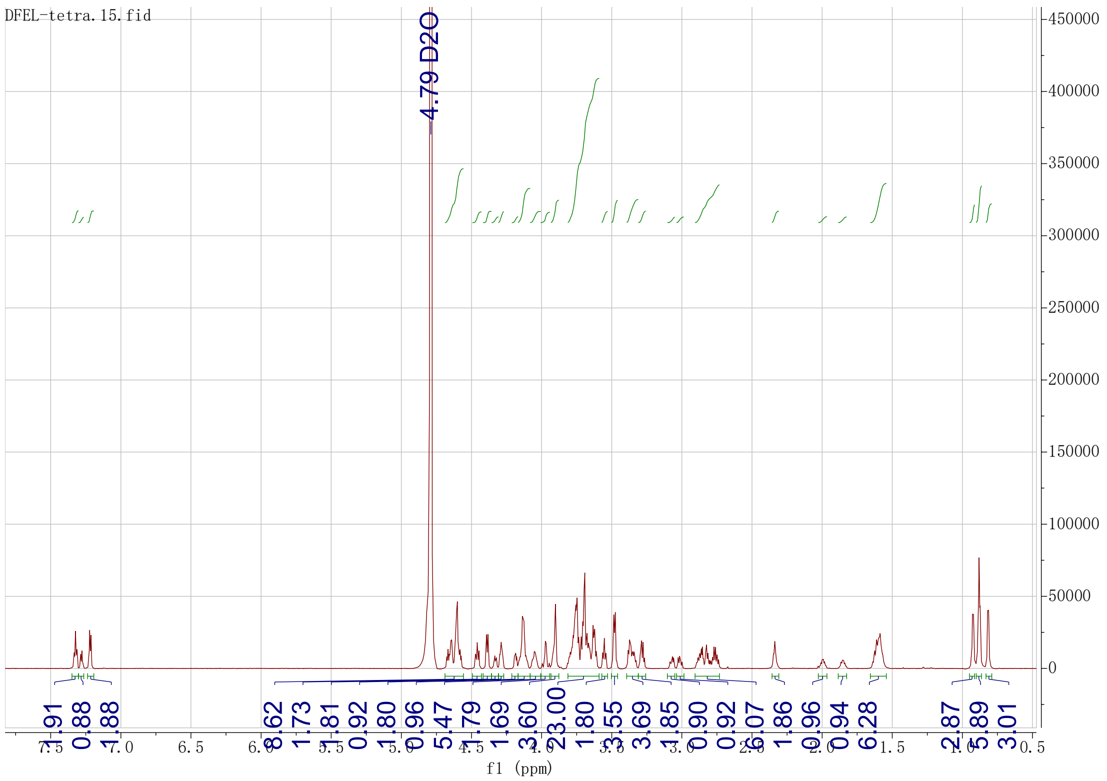
DFEL-tetra.15.fid
4.79 D2O
f1 (ppm)
1.91
0.88
1.88
8.62
1.73
1.81
0.92
1.80
0.96
5.47
1.79
1.69
3.60
23.00
1.80
3.55
3.69
1.85
0.90
0.92
6.07
1.86
0.96
0.94
6.28
2.87
5.89
3.01

^{13}C NMR (201 MHz, D_2O)

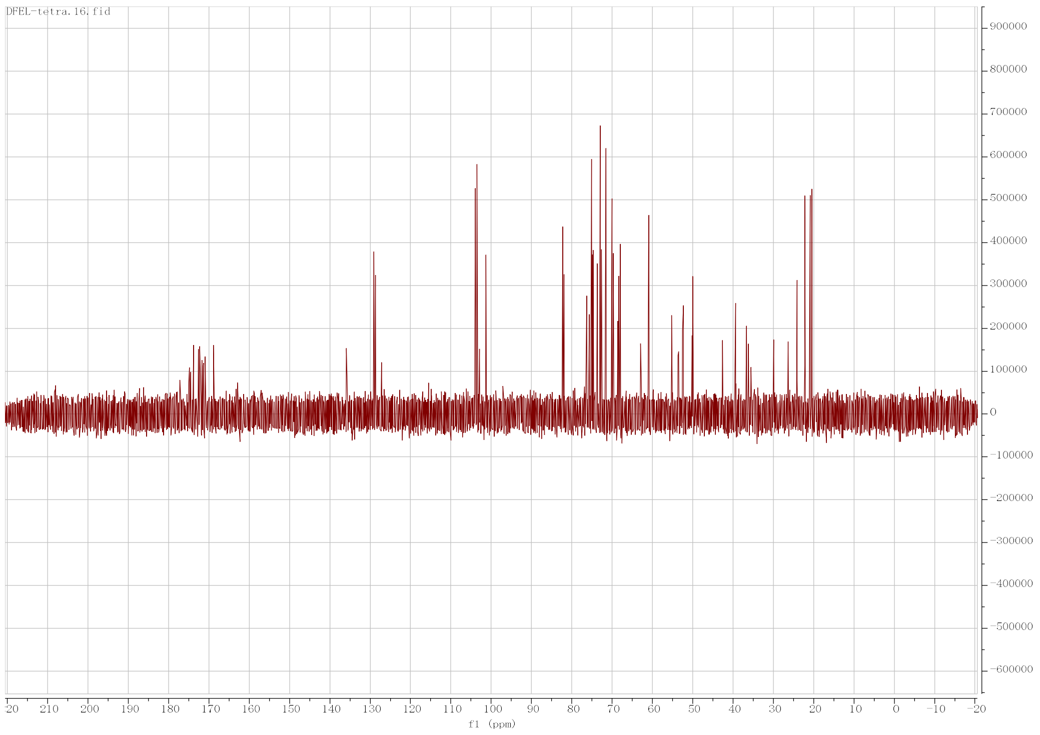

COSY (800 MHz, D_2O)

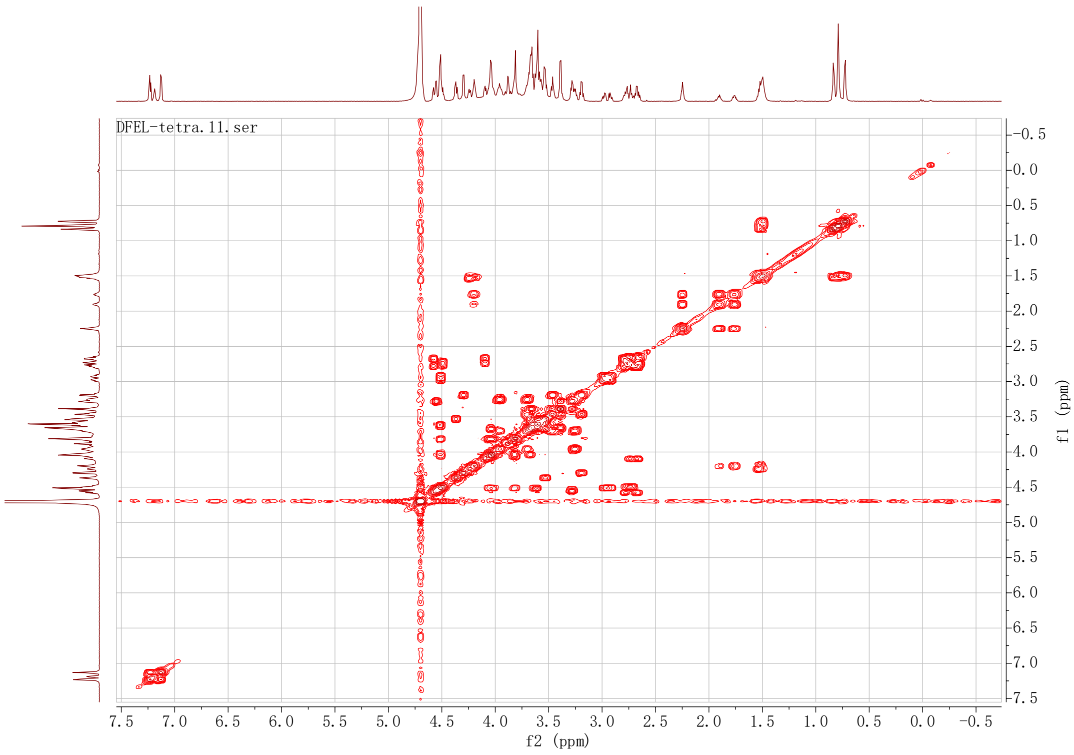

HSQC (800 MHz, D_2O)

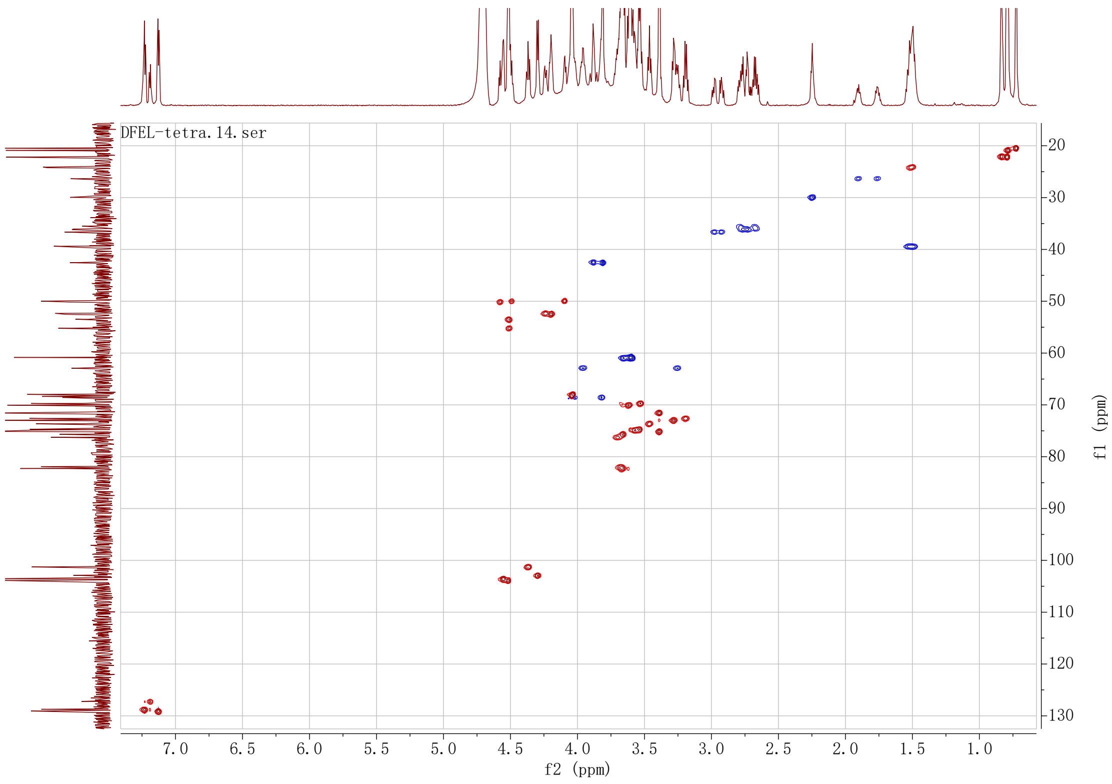

Coupled HSQC (800 MHz, D_2O)

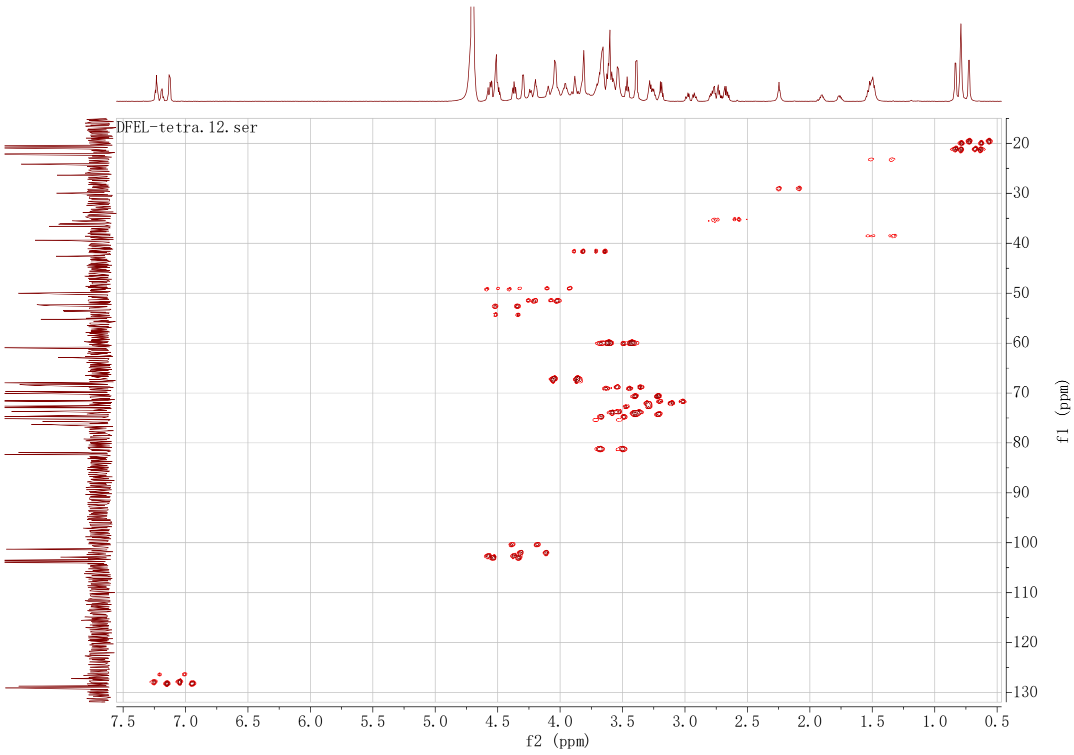

HMBC (800 MHz, D_2O)

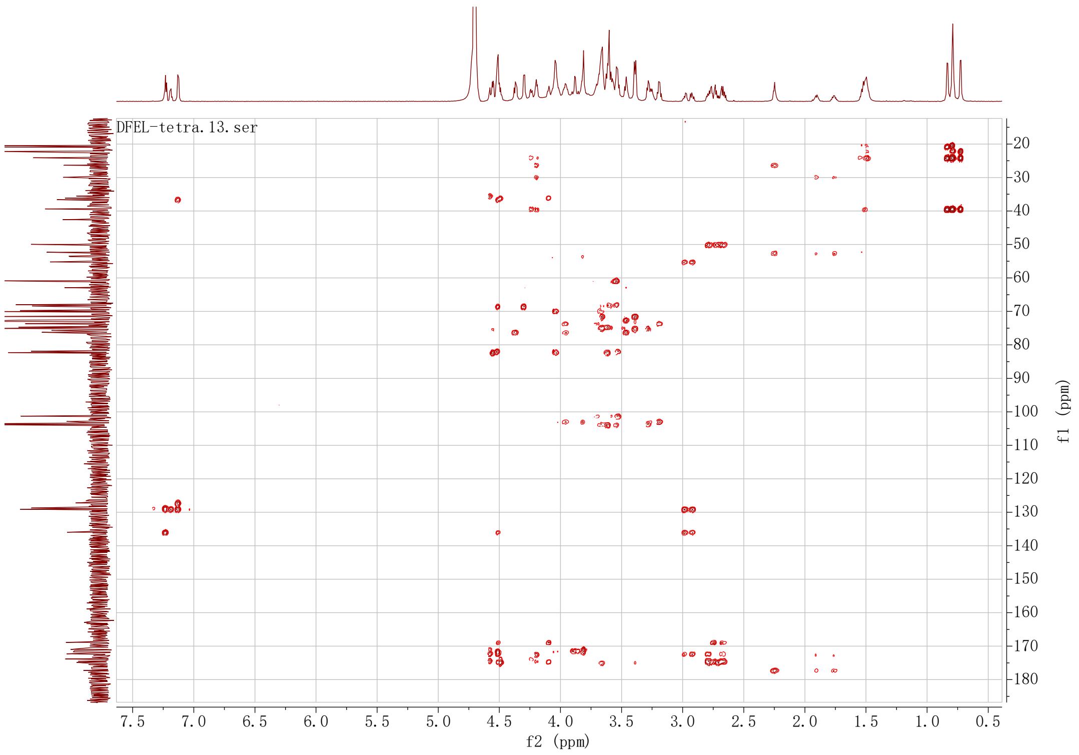

HPLC

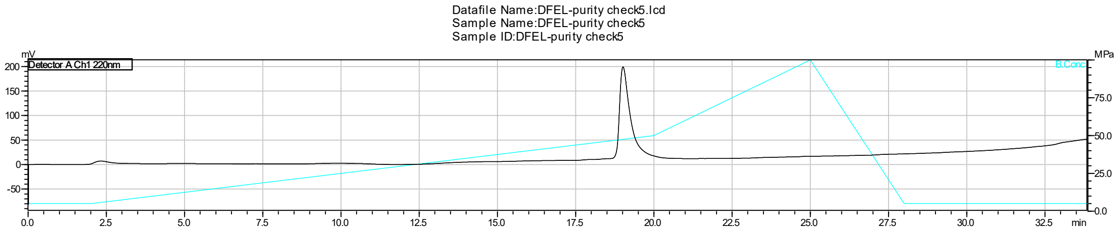

MS

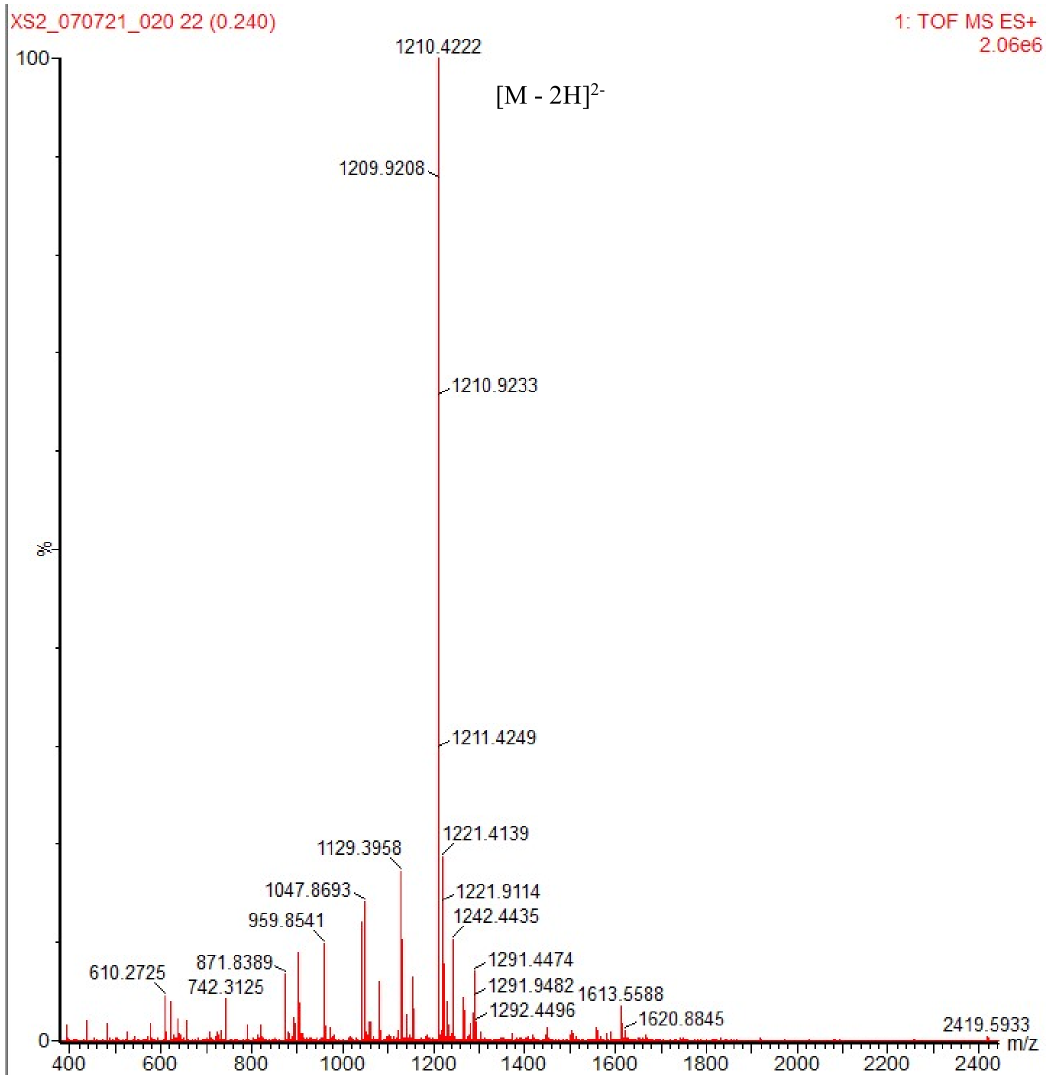

XS2_070721_020 22 (0.240)
1: TOF MS ES+
2.06e6
[M - 2H]^2-
1210.4222
1209.9208
1210.9233
1211.4249
1221.4139
1129.3958
1047.8693
1221.9114
959.8541
1242.4435
871.8389
1291.4474
610.2725
742.3125
1291.9482
1613.5588
1292.4496
1620.8845
2419.5933
100
%
0
400
600
800
1000
1200
1400
1600
1800
2000
2200
2400
m/z

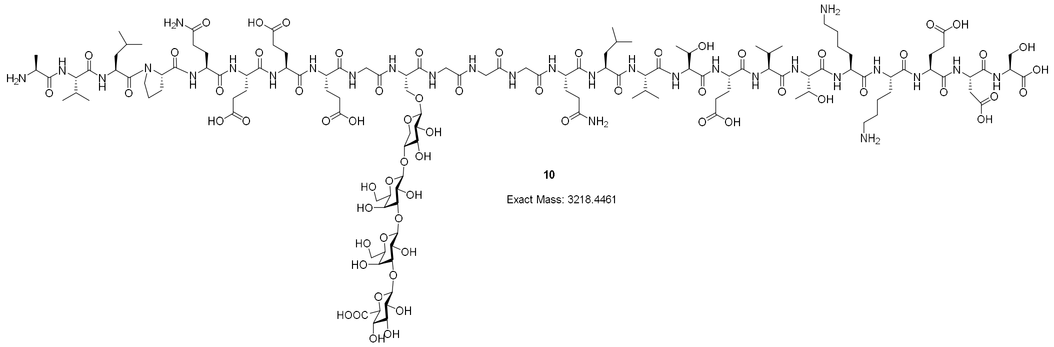

10

Exact Mass: 3218.4461

^{1}H NMR (800 MHz, D_2O)

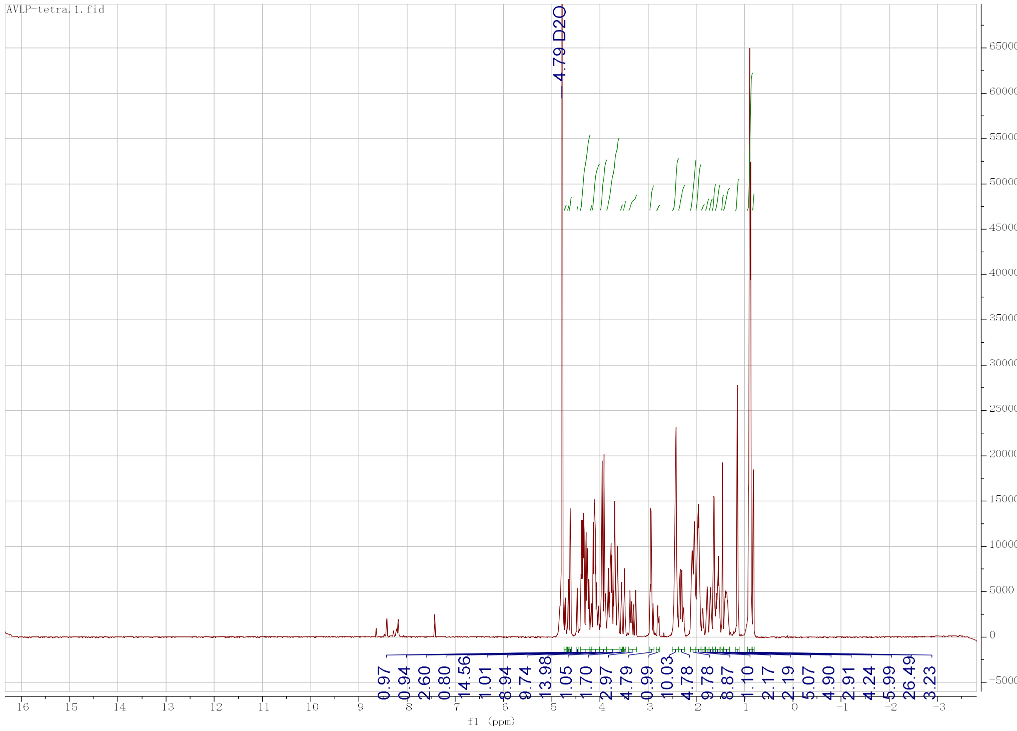

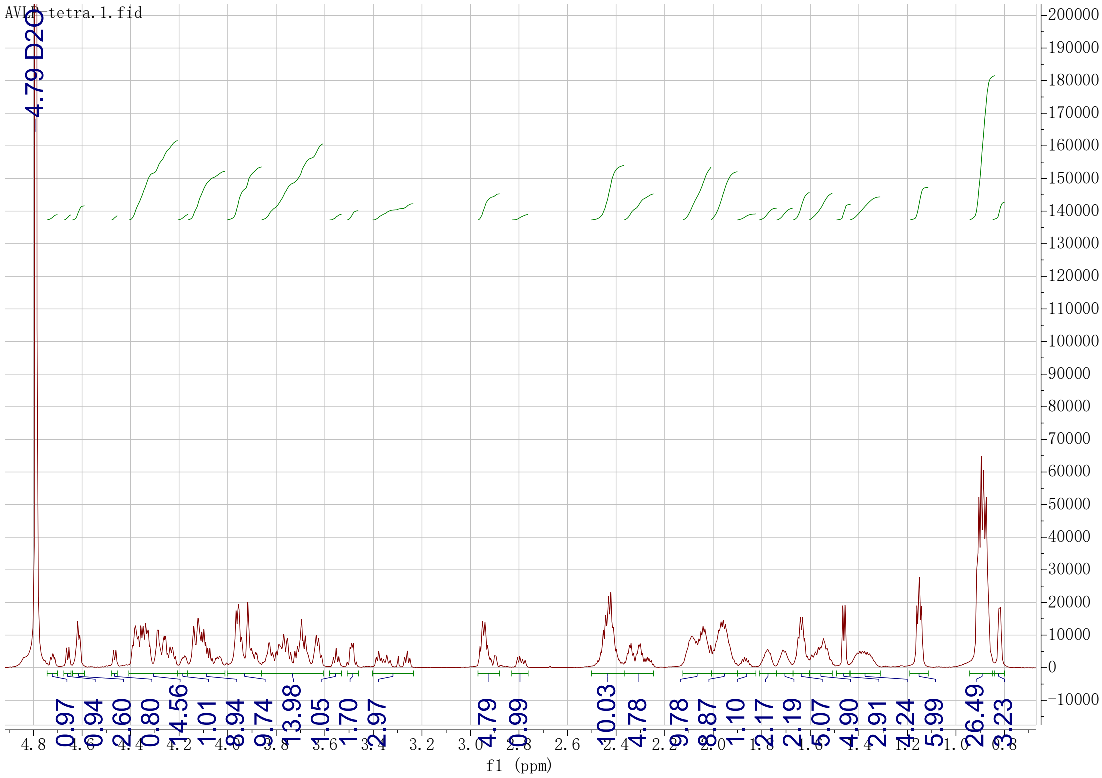

AVIII-tetra.1.fid
4.79 D2O
f1 (ppm)

^{13}C NMR (201 MHz, D_2O)

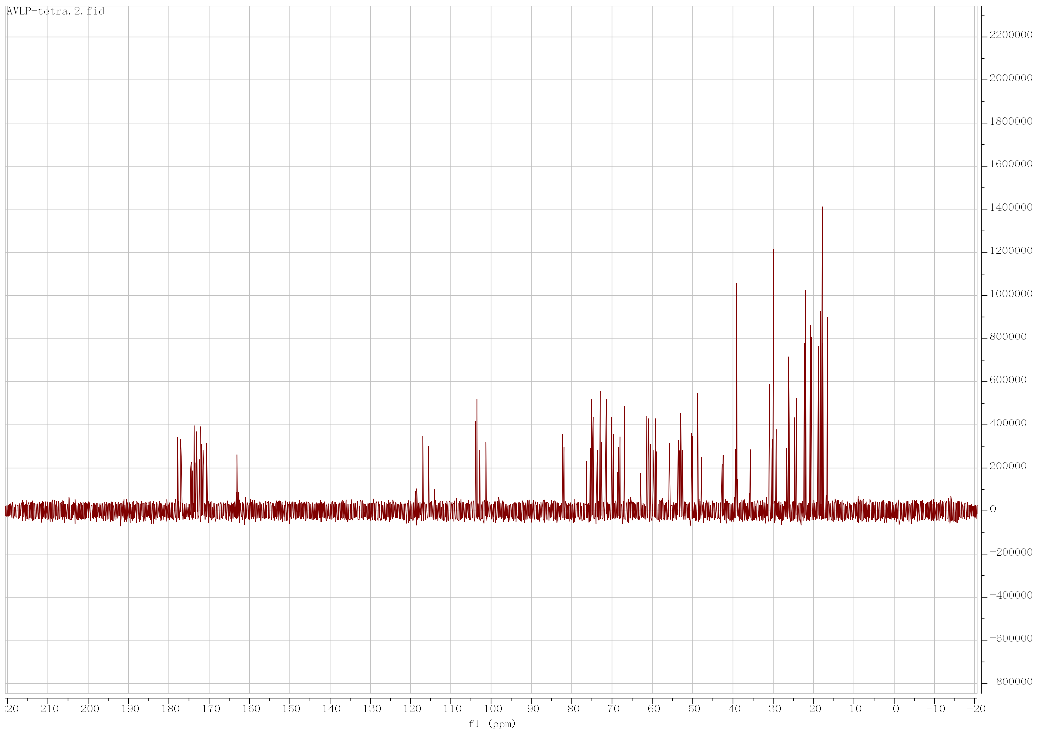

COSY (800 MHz, D_2O)

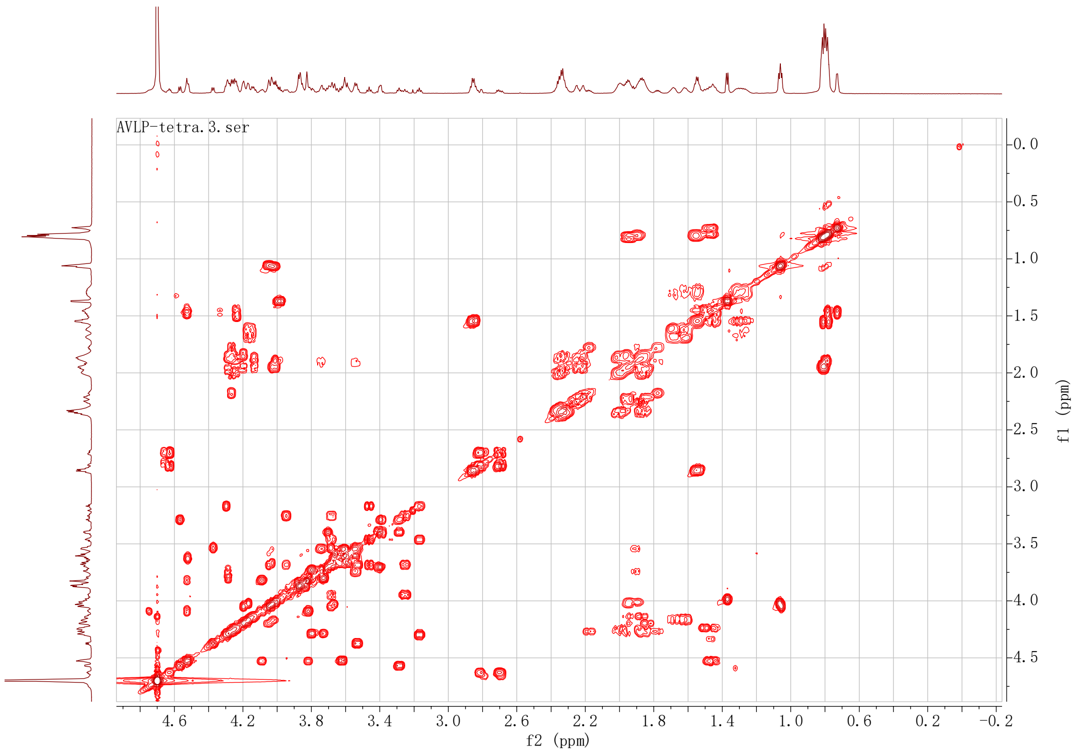

HSQC (800 MHz, D_2O)

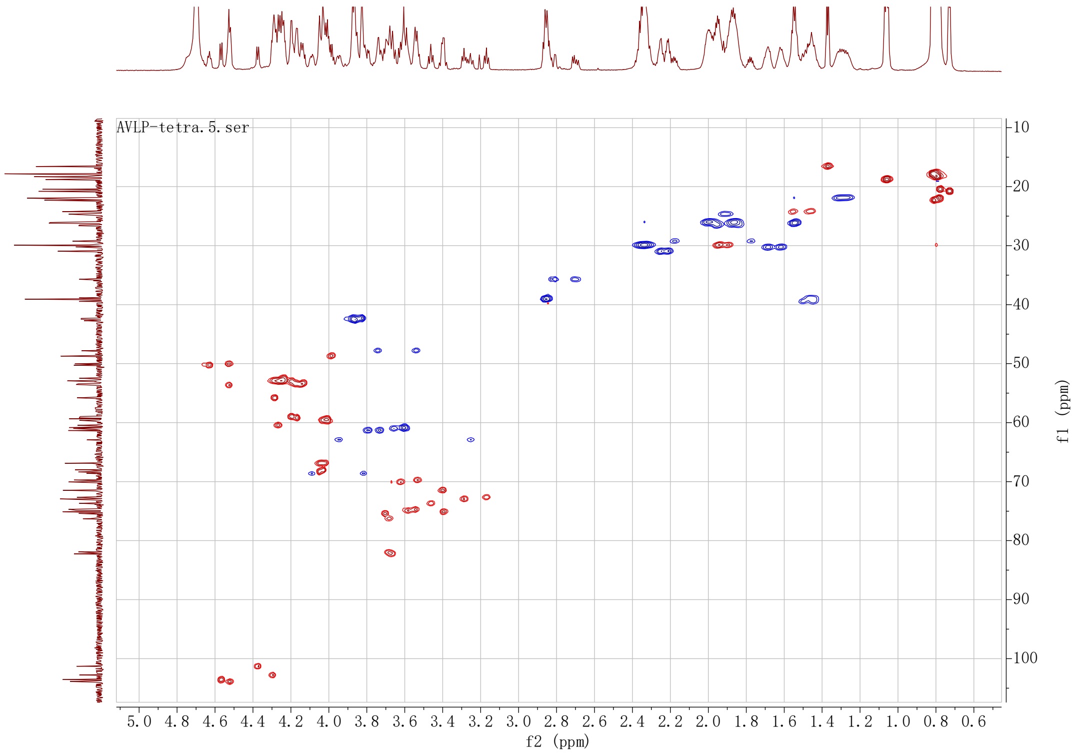

Coupled HSQC (800 MHz, D_2O)

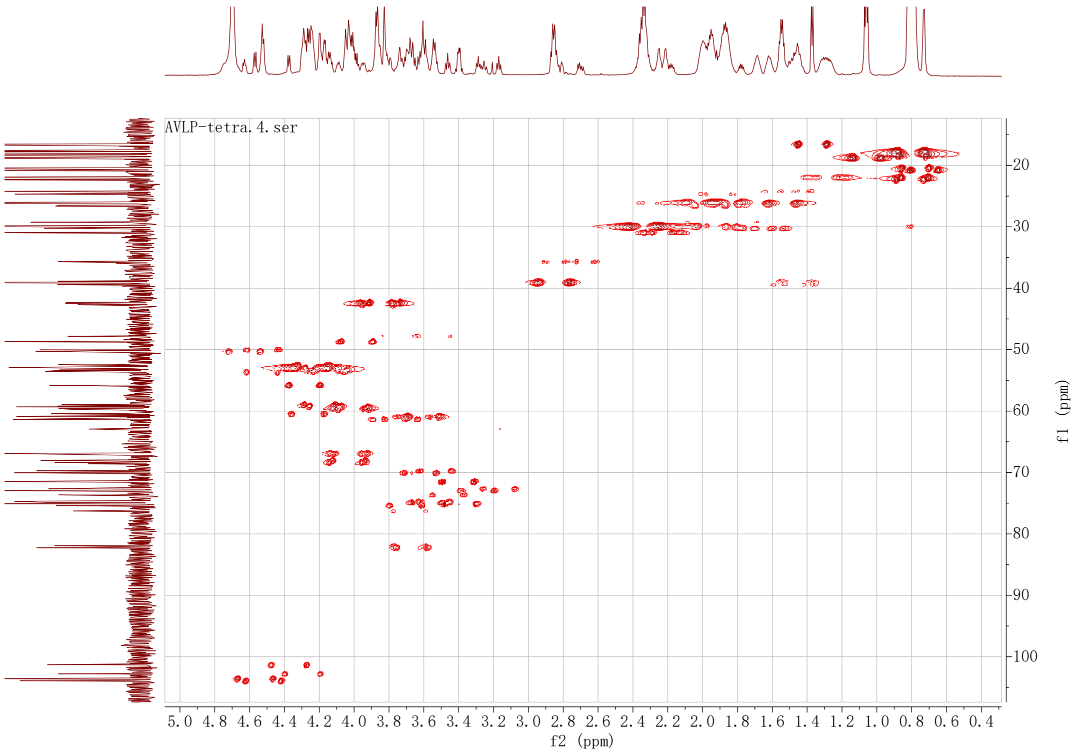

HMBC (800 MHz, D_2O)

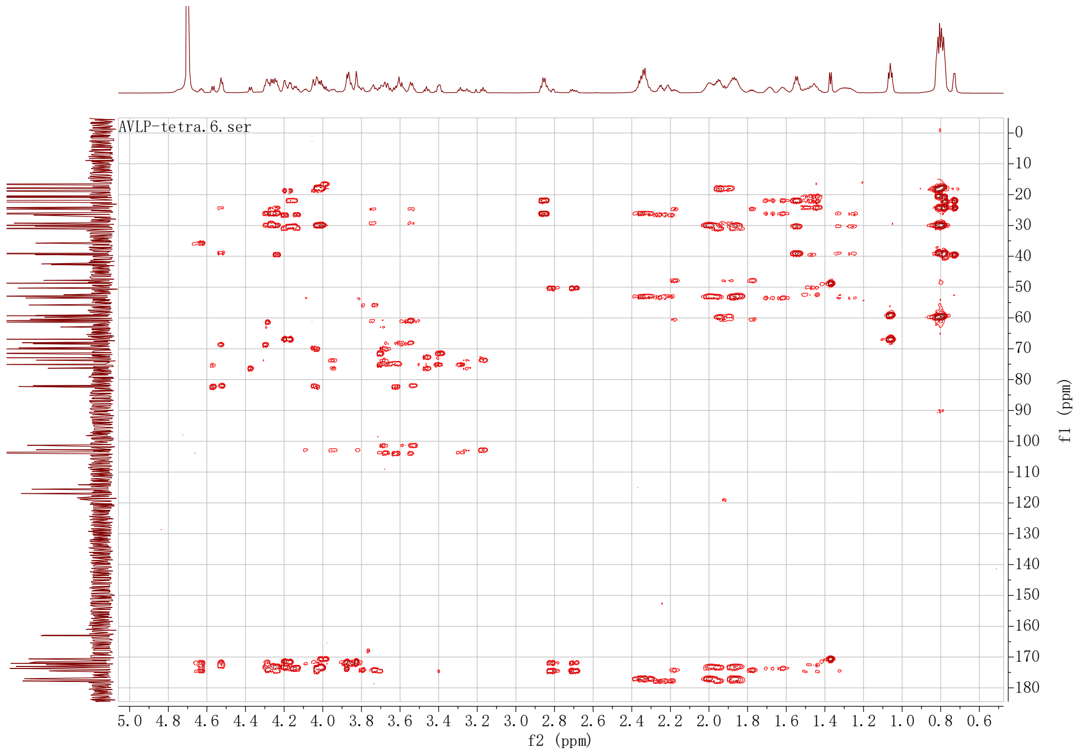

HPLC

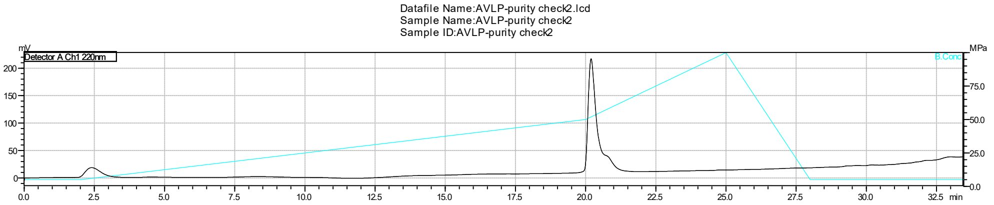

MS

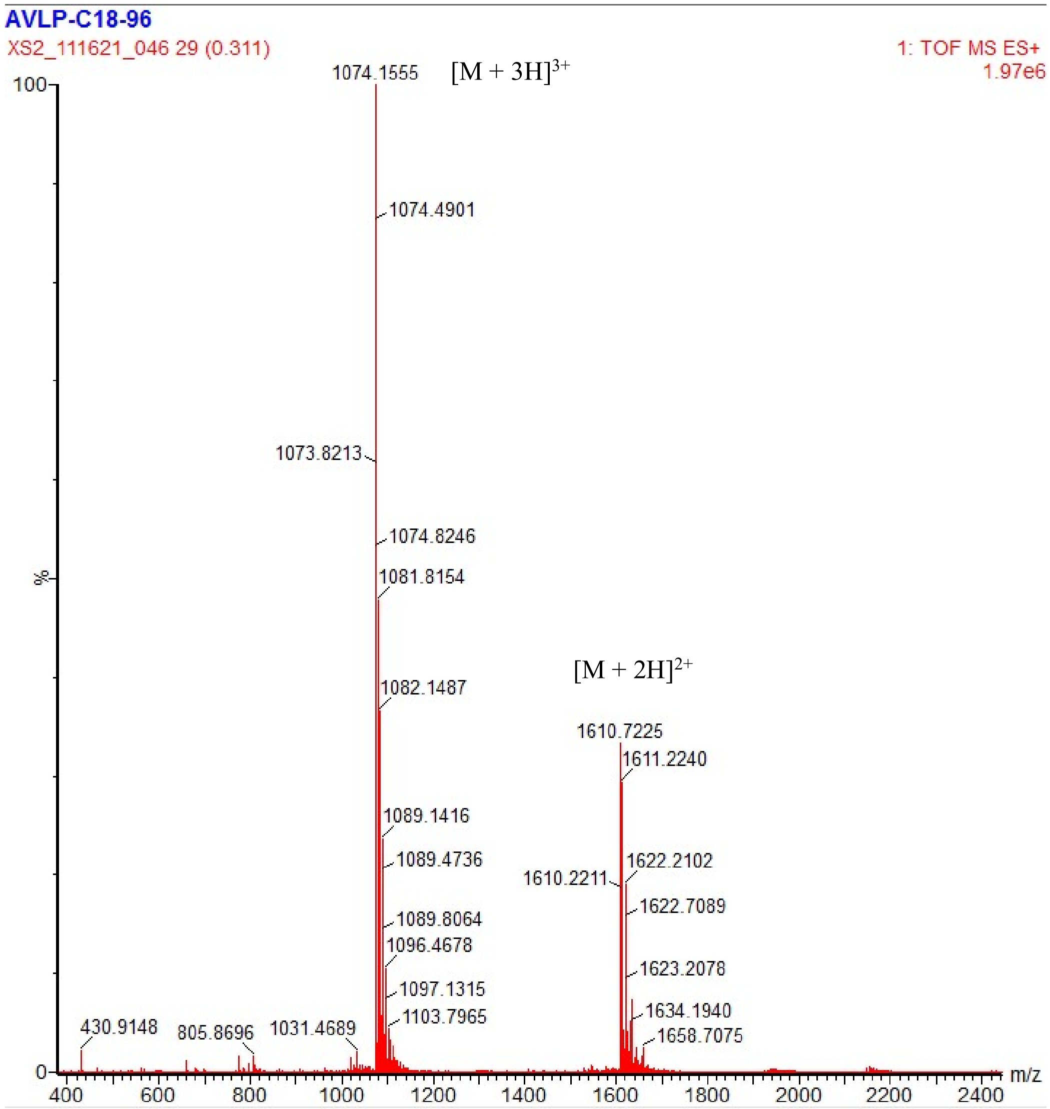

AVLP-C18-96
XS2_111621_046 29 (0.311)
1: TOF MS ES+
1.97e6
[M + 3H]$^{3+}$
[M + 2H]$^{2+}$
1074.1555
1074.4901
1073.8213
1074.8246
1081.8154
1082.1487
1089.1416
1089.4736
1089.8064
1096.4678
1097.1315
1103.7965
430.9148
805.8696
1031.4689
1610.7225
1611.2240
1610.2211
1622.2102
1622.7089
1623.2078
1634.1940
1658.7075
100
%
0
m/z
400
600
800
1000
1200
1400
1600
1800
2000
2200
2400

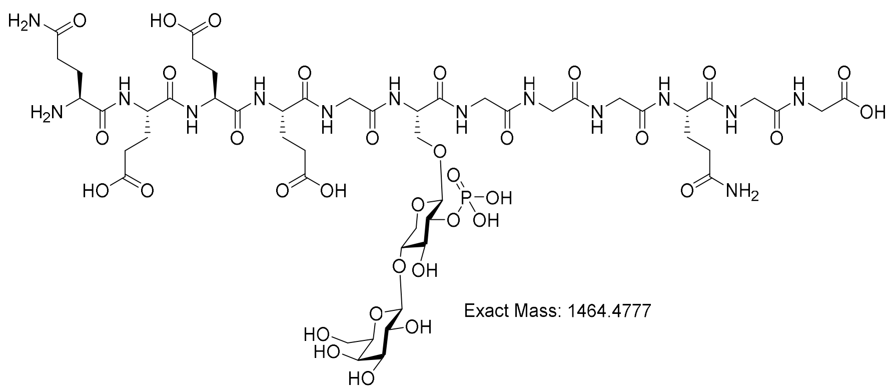

^{1}H NMR (800 MHz, D_2O)

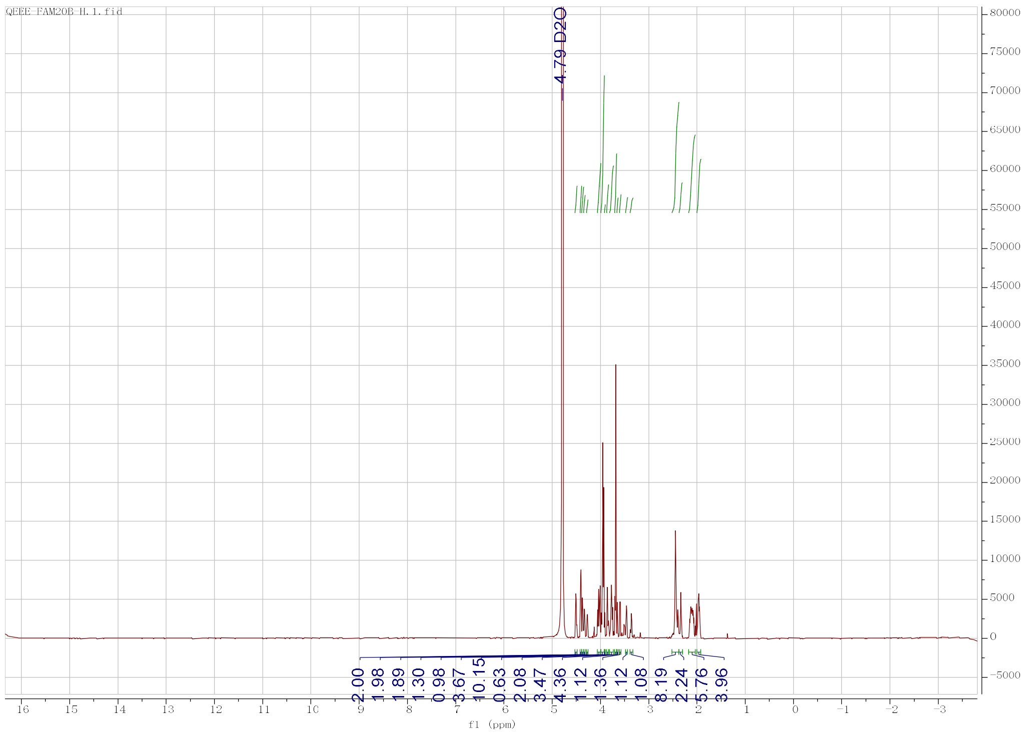

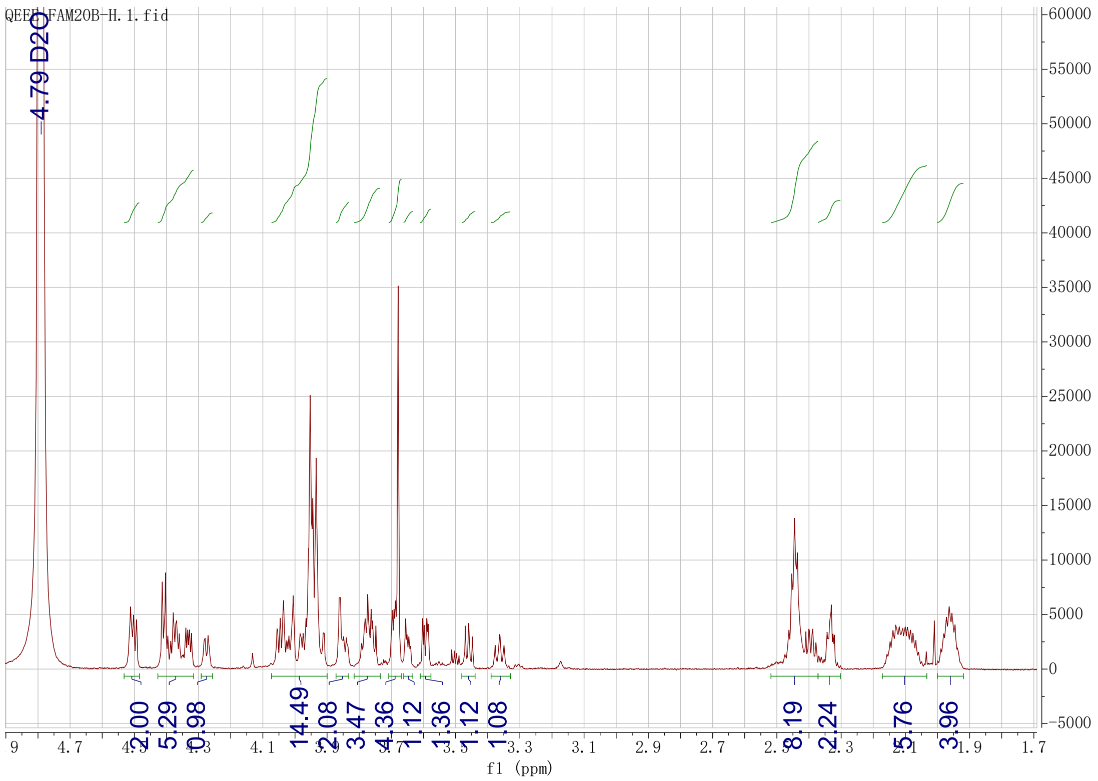
QEEE-FAM20B-H.1.fid
4.79 D2O
2.00
5.29
0.98
14.49
2.08
3.47
4.36
1.12
1.36
1.12
1.08
8.19
2.24
5.76
3.96
f1 (ppm)

^{13}C NMR (201 MHz, D_2O)

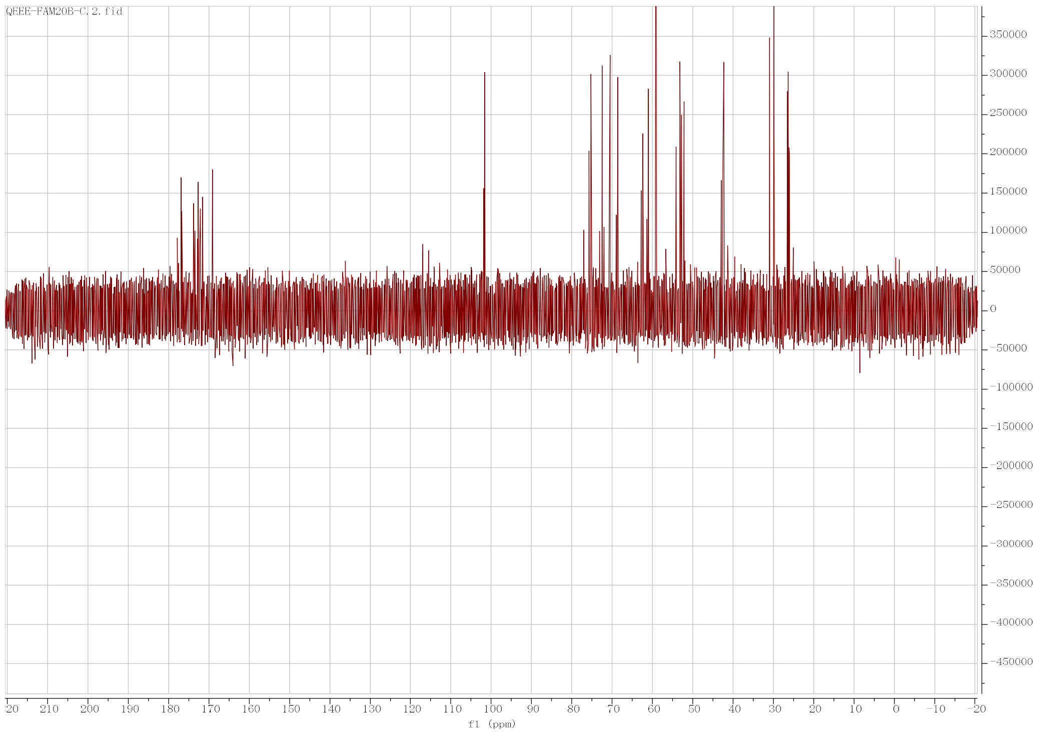

COSY (800 MHz, D_2O)

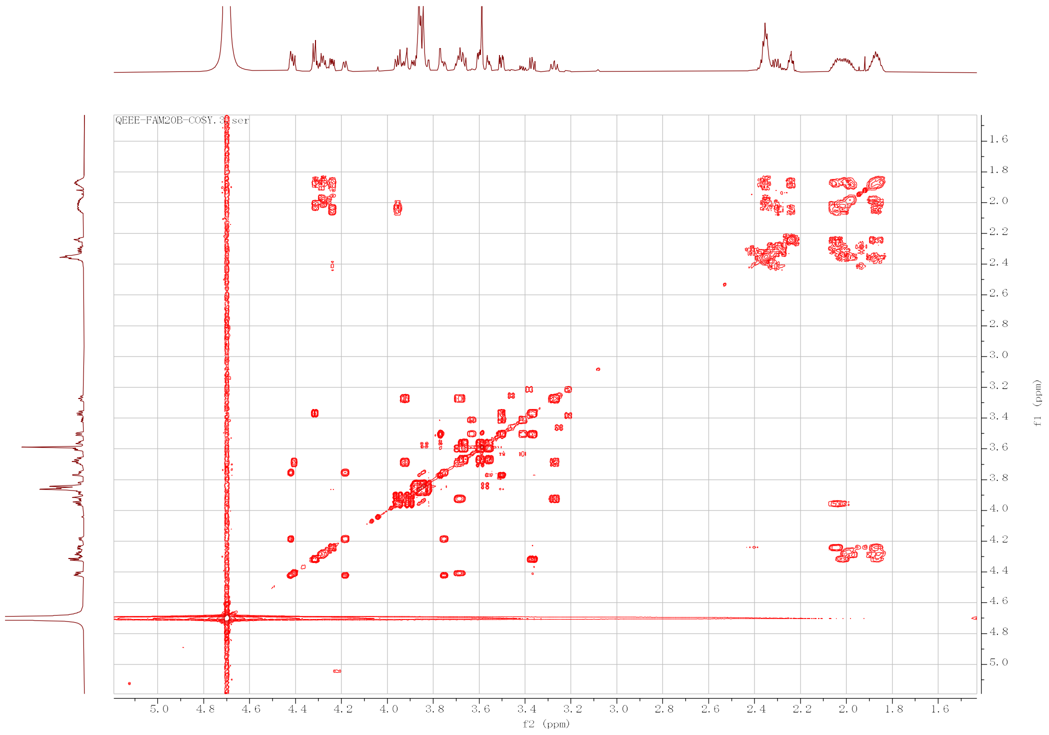

HSQC (800 MHz, D_2O)

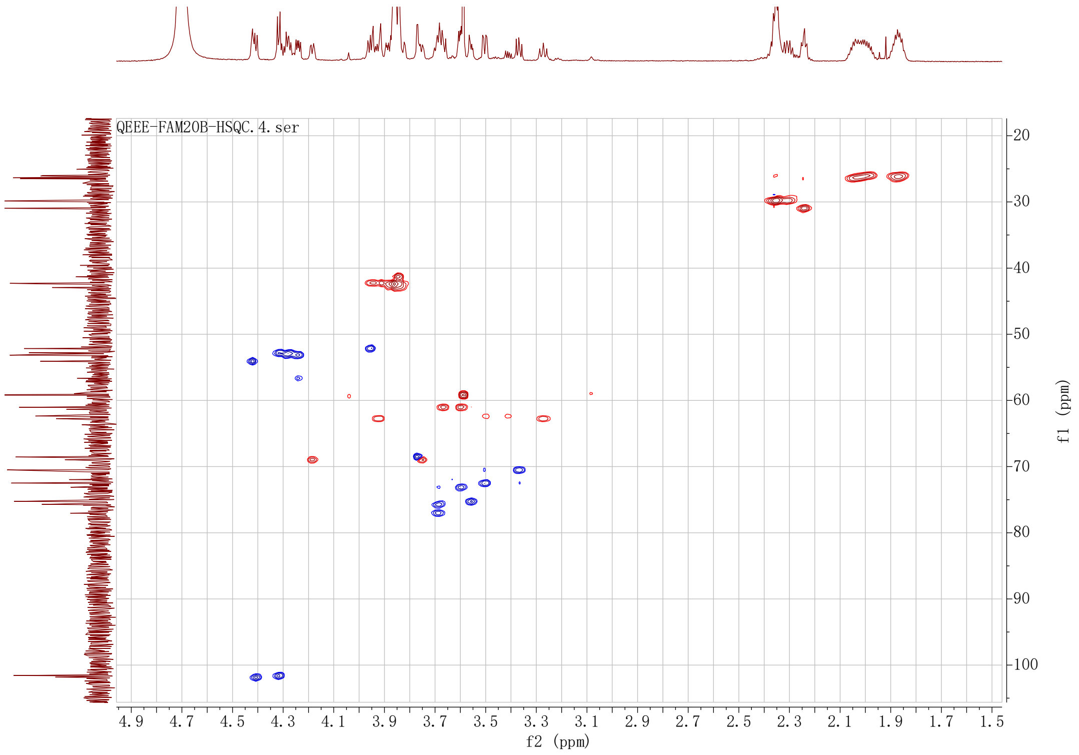

Coupled HSQC (800 MHz, D_2O)

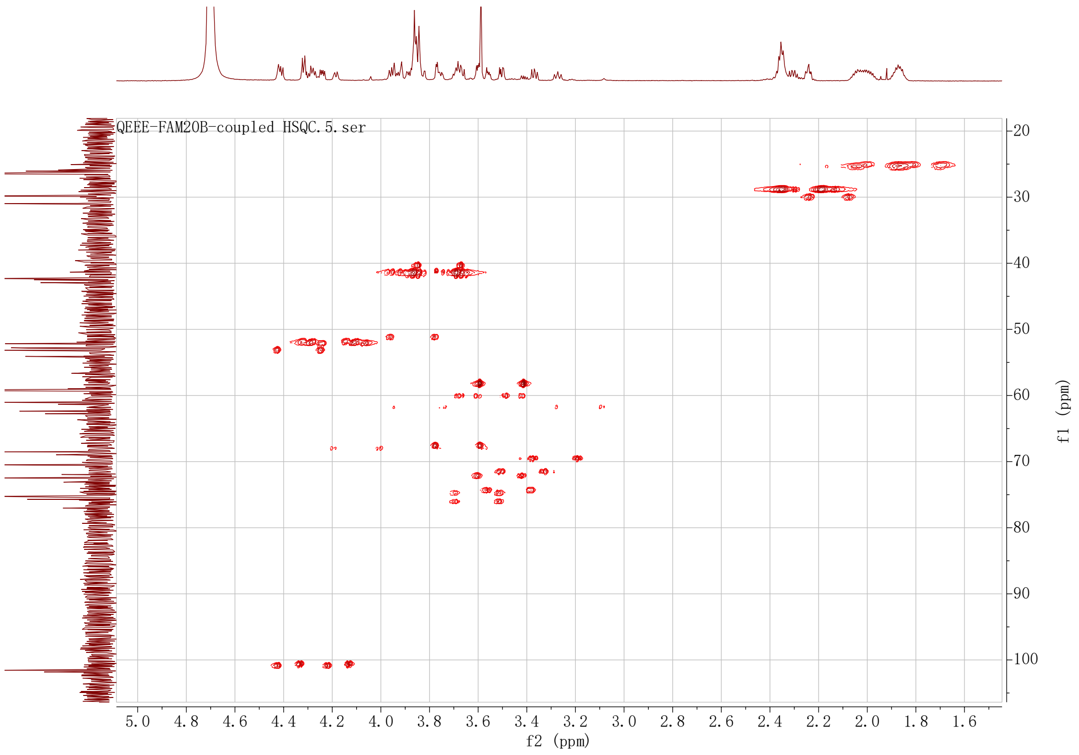

HMBC (800 MHz, D_2O)

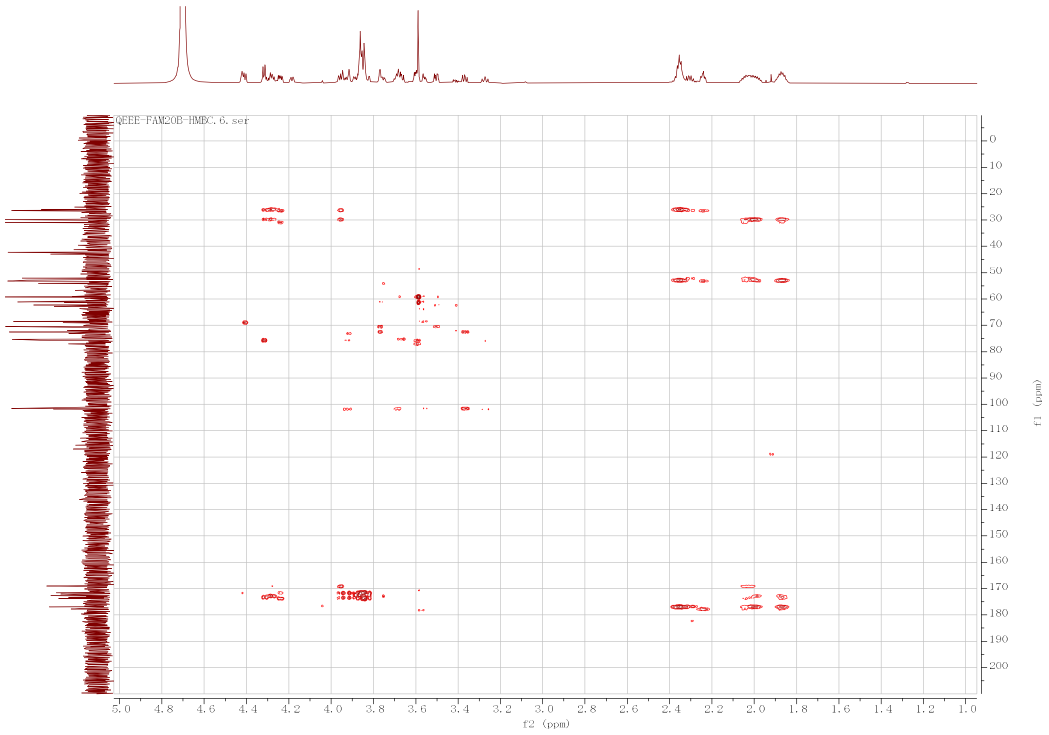

HPLC

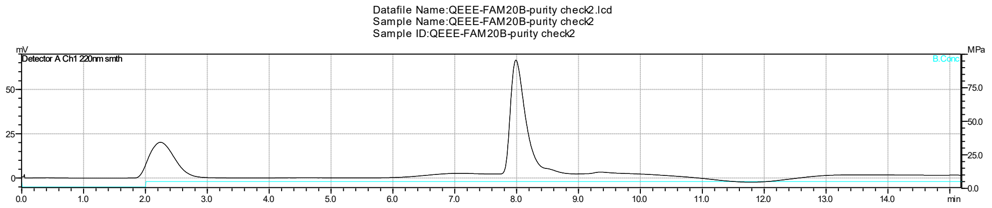

MS

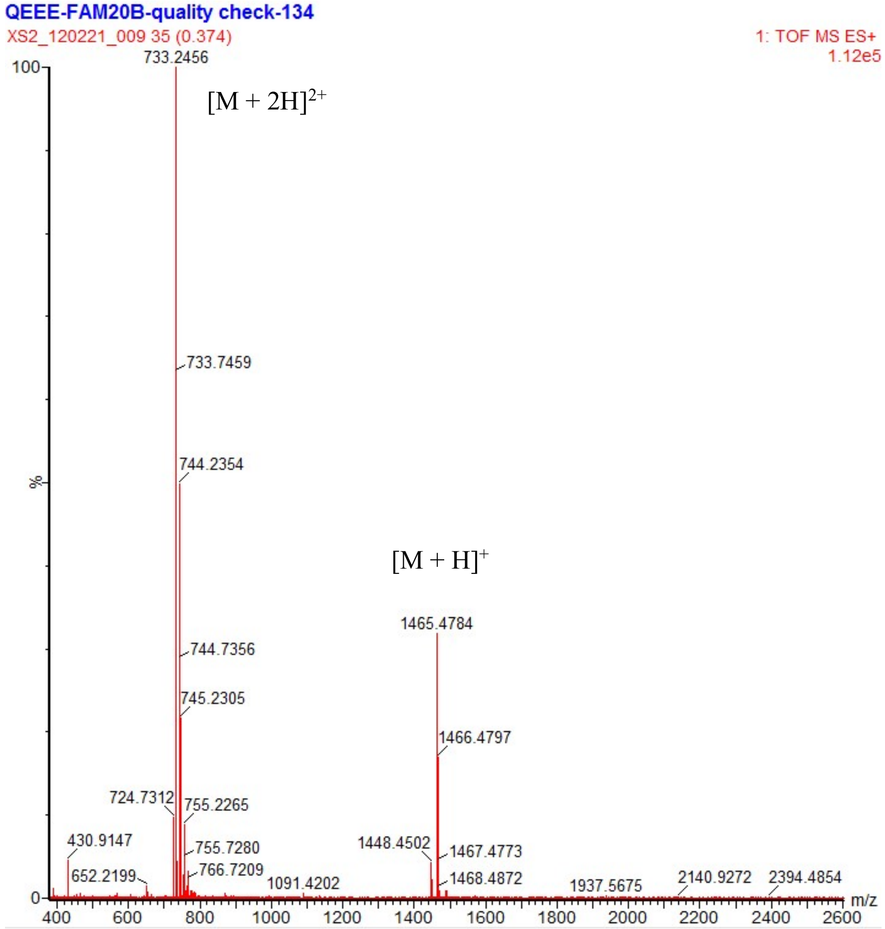
QEEE-FAM20B-quality check-134
XS2_120221_009 35 (0.374)
1: TOF MS ES+
1.12e5
733.2456
[M + 2H]2+
733.7459
744.2354
[M + H]+
1465.4784
744.7356
745.2305
1466.4797
724.7312
755.2265
430.9147
755.7280
1448.4502
1467.4773
766.7209
652.2199
1091.4202
1468.4872
1937.5675
2140.9272
2394.4854
%
m/z

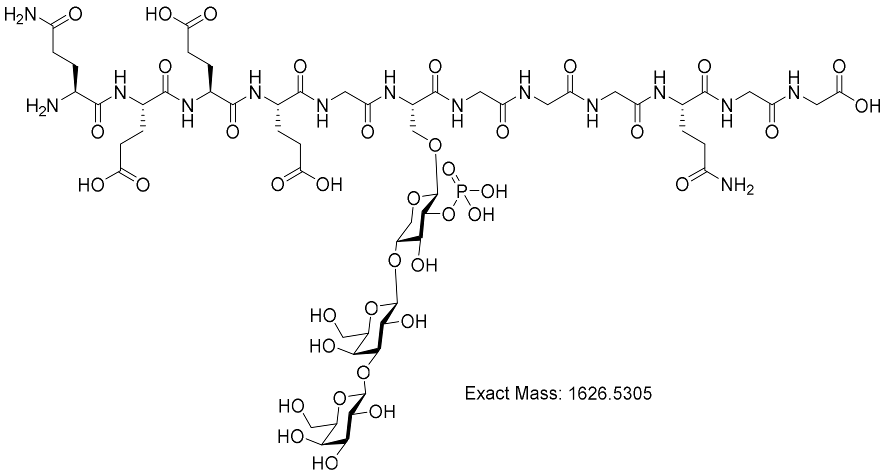

Exact Mass: 1626.5305

^{1}H NMR (800 MHz, D_2O)

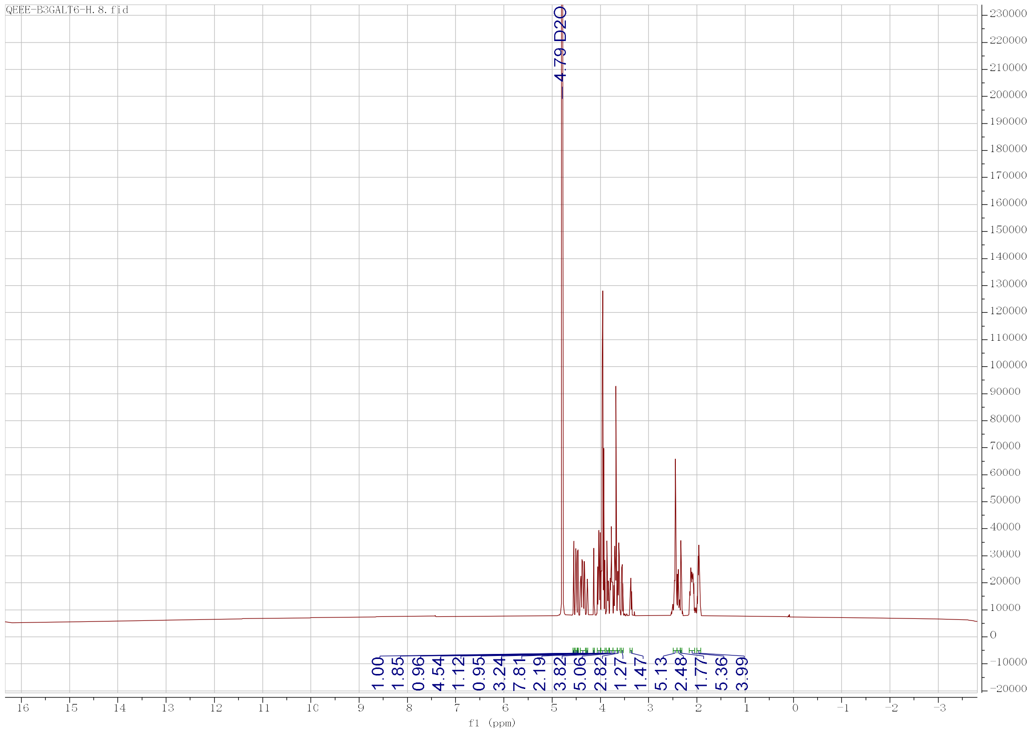

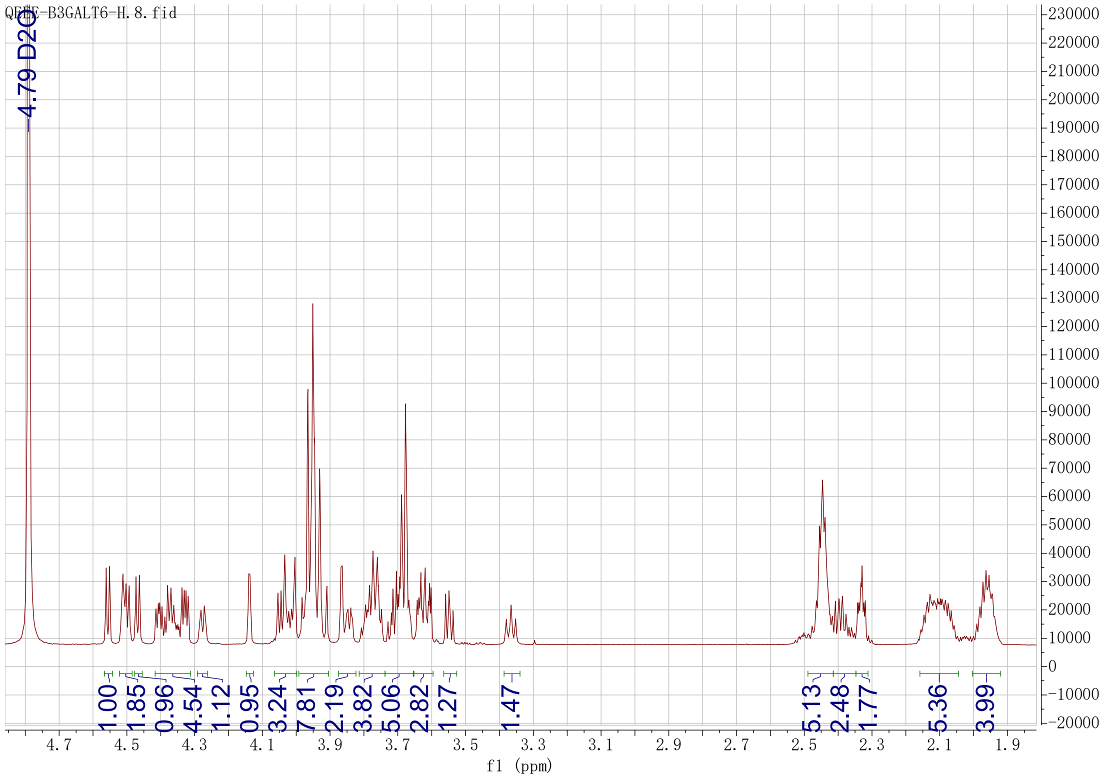
QE?E-B3GALT6-H.8.fid
4.79 D2O
1.00
1.85
0.96
4.54
1.12
0.95
3.24
7.81
2.19
3.82
5.06
2.82
1.27
1.47
5.13
2.48
1.77
5.36
3.99
4.7 4.5 4.3 4.1 3.9 3.7 3.5 3.3 3.1 2.9 2.7 2.5 2.3 2.1 1.9
f1 (ppm)
230000 220000 210000 200000 190000 180000 170000 160000 150000 140000 130000 120000 110000 100000 90000 80000 70000 60000 50000 40000 30000 20000 10000 0 -10000 -20000

^{13}C NMR (201 MHz, D_2O)

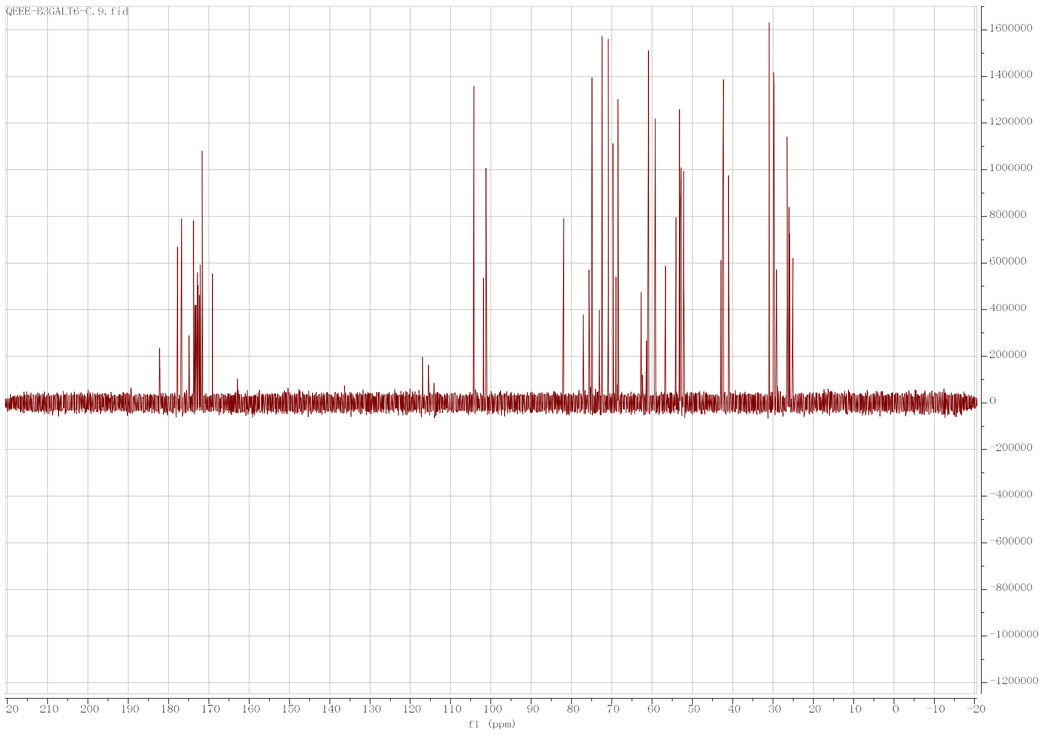

COSY (800 MHz, D_2O)

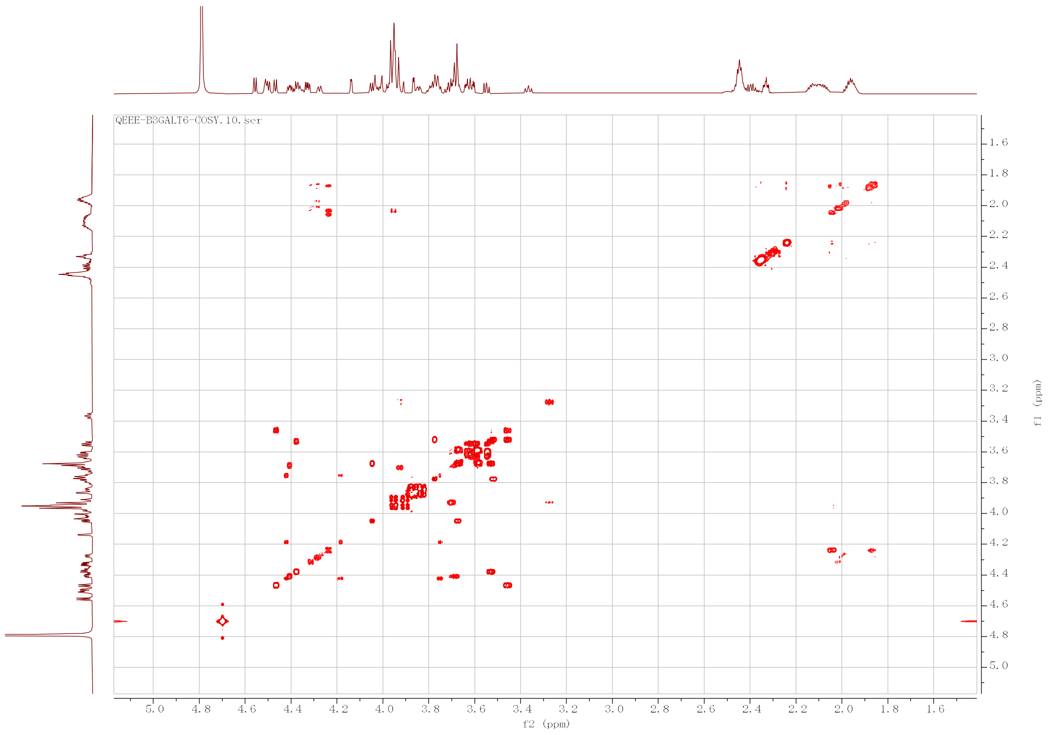

HSQC (800 MHz, D_2O)

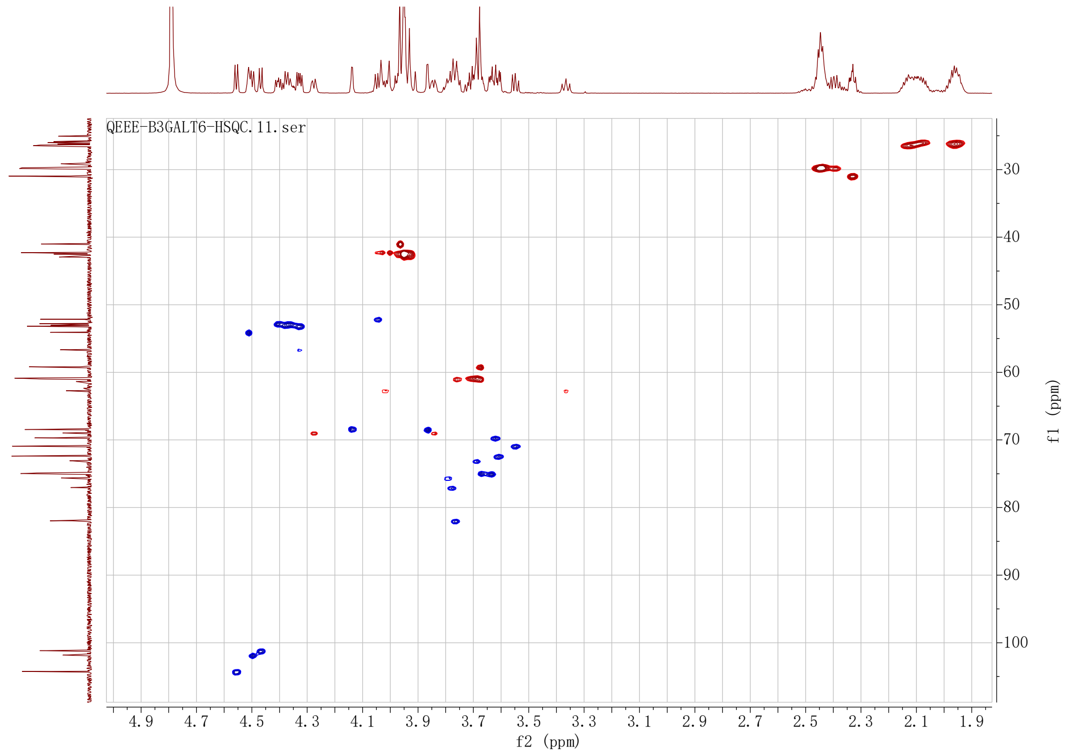

Coupled HSQC (800 MHz, D_2O)

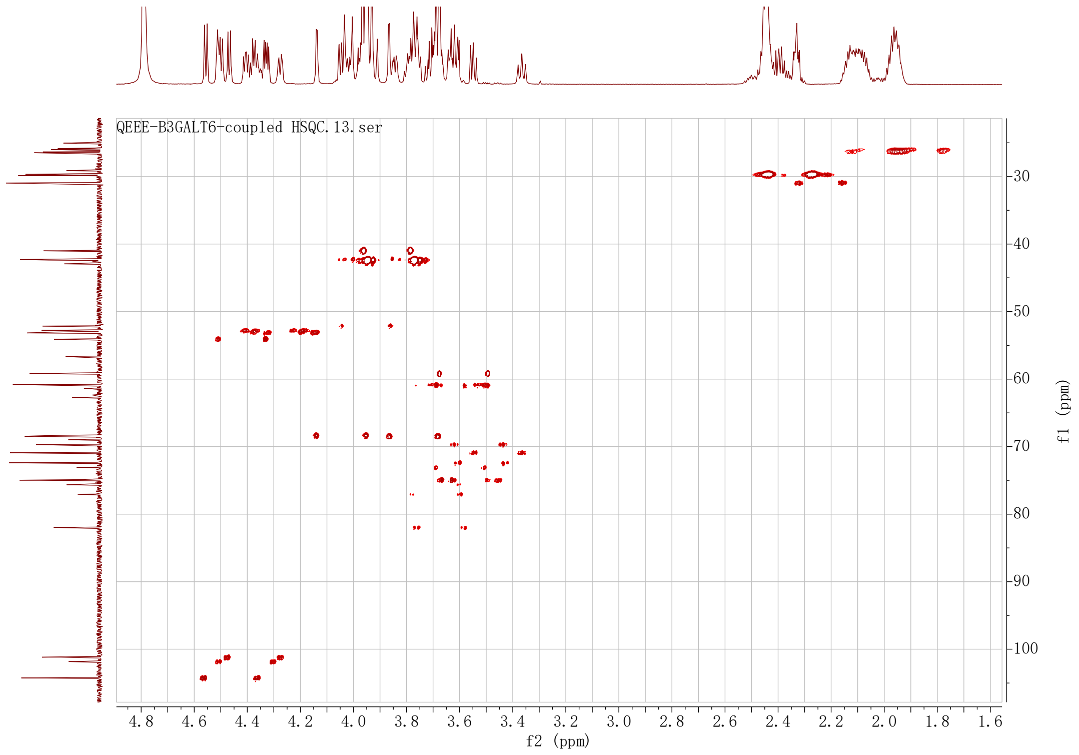

HMBC (800 MHz, D_2O)

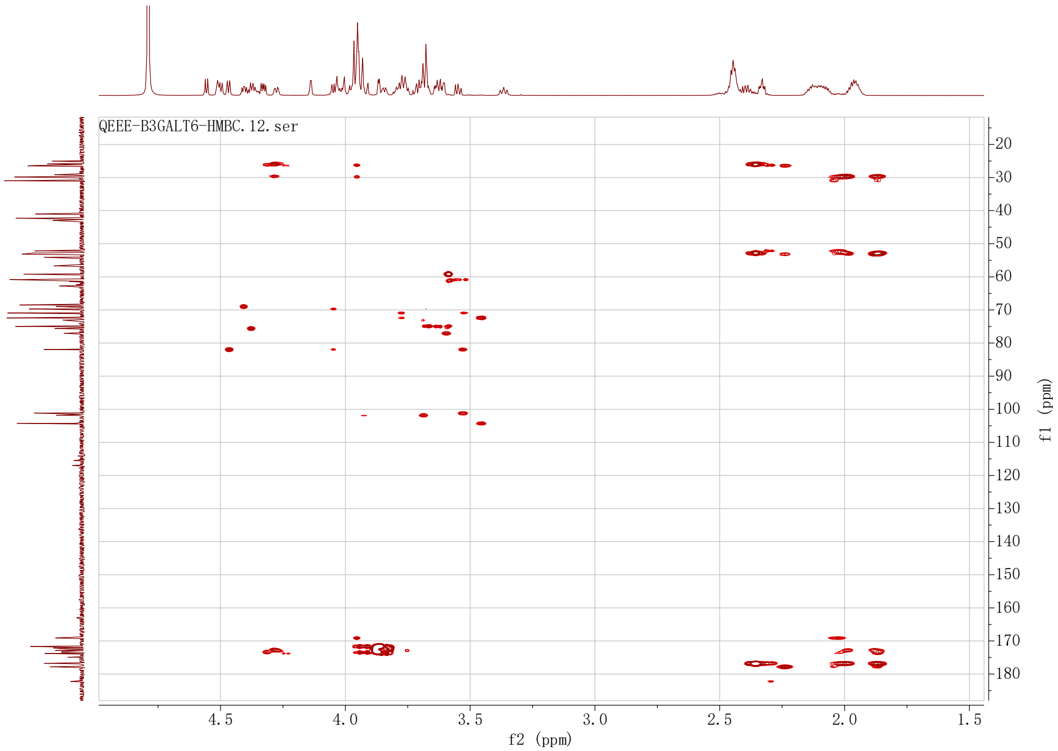

MS

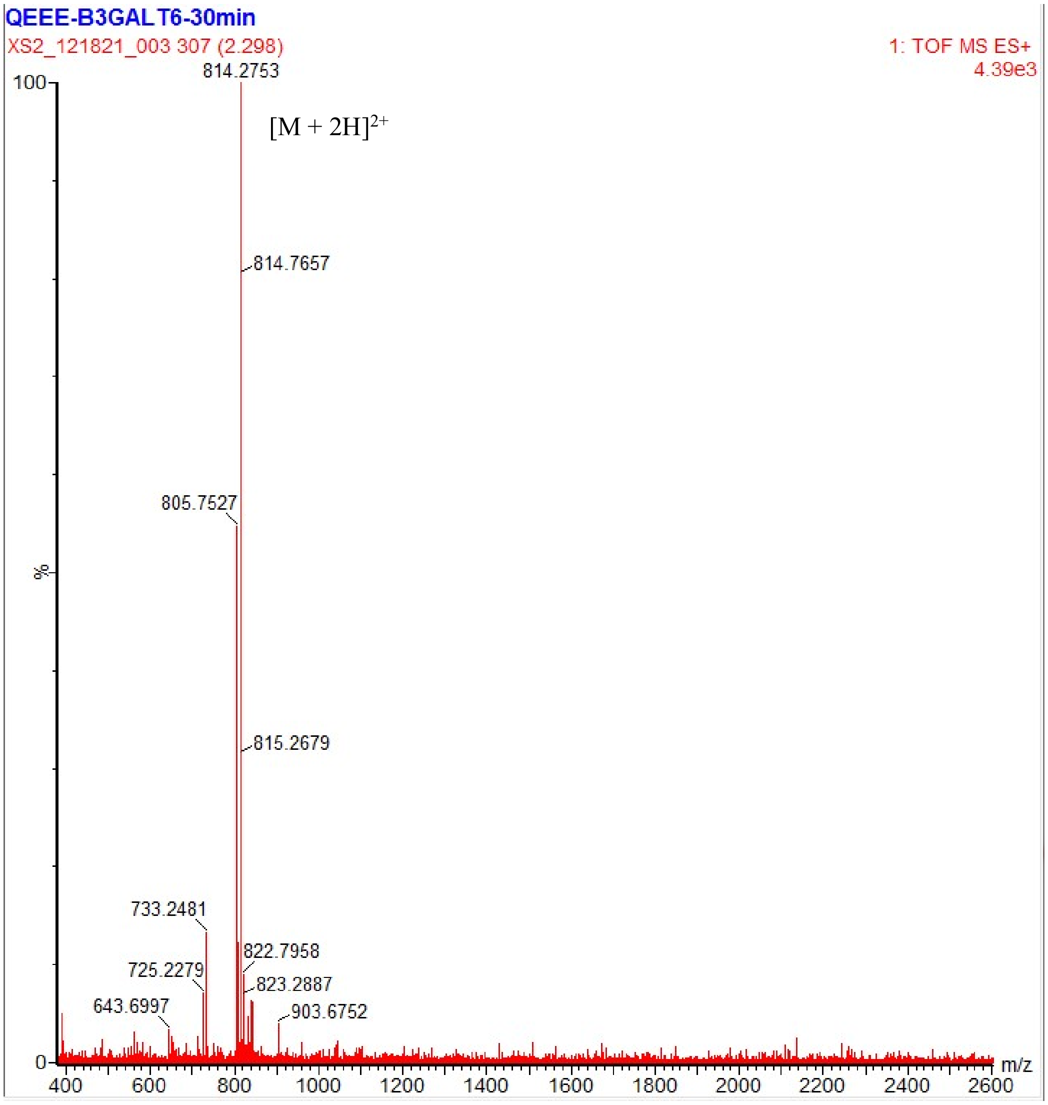
QEEE-B3GALT6-30min
XS2_121821_003 307 (2.298)
1: TOF MS ES+
4.39e3
814.2753
[M + 2H]2+
814.7657
805.7527
815.2679
733.2481
822.7958
725.2279
823.2887
643.6997
903.6752
100
%
0
400
600
800
1000
1200
1400
1600
1800
2000
2200
2400
2600
m/z

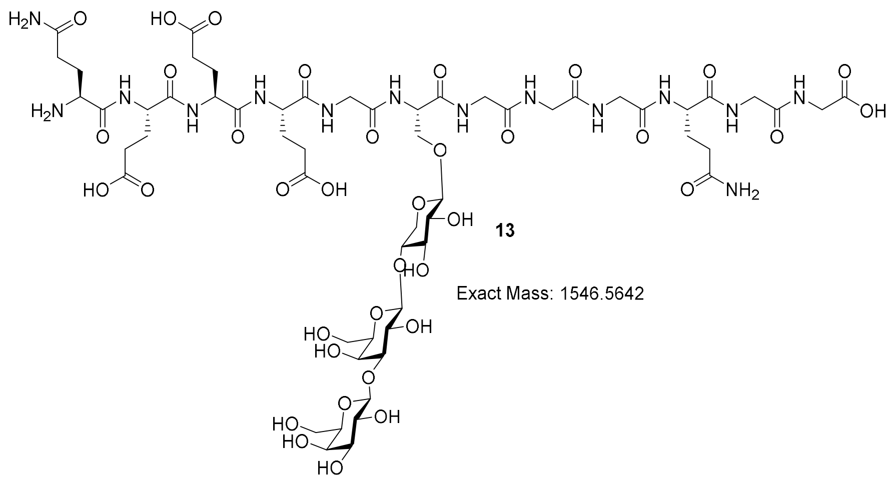

13

Exact Mass: 1546.5642

^{1}H NMR (800 MHz, D_2O)

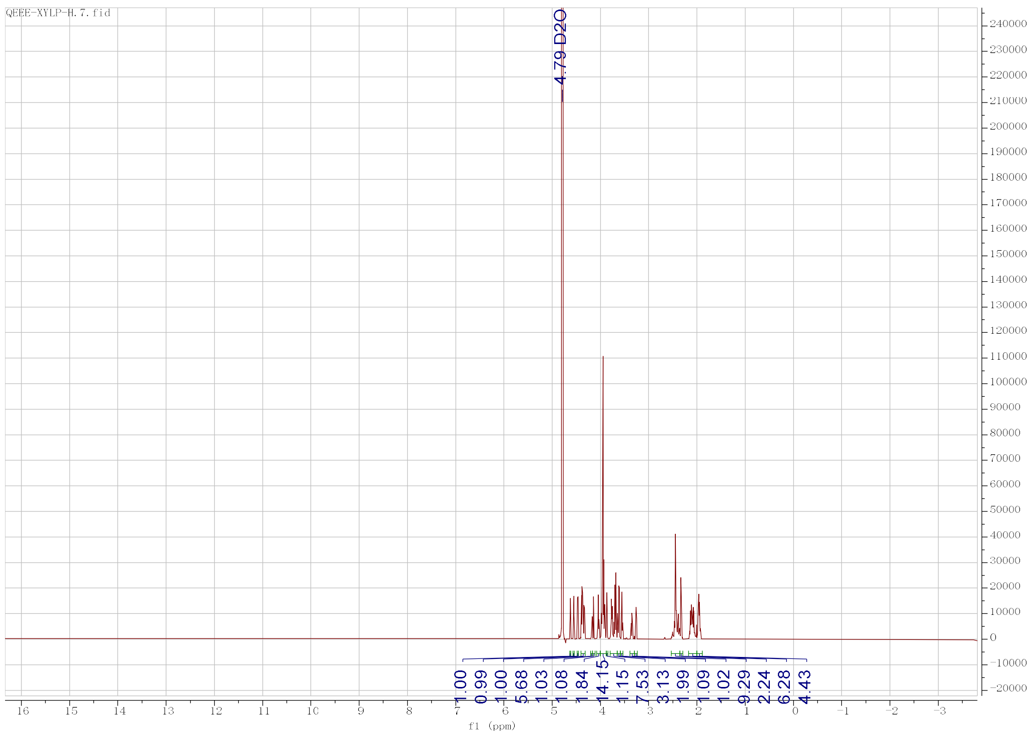

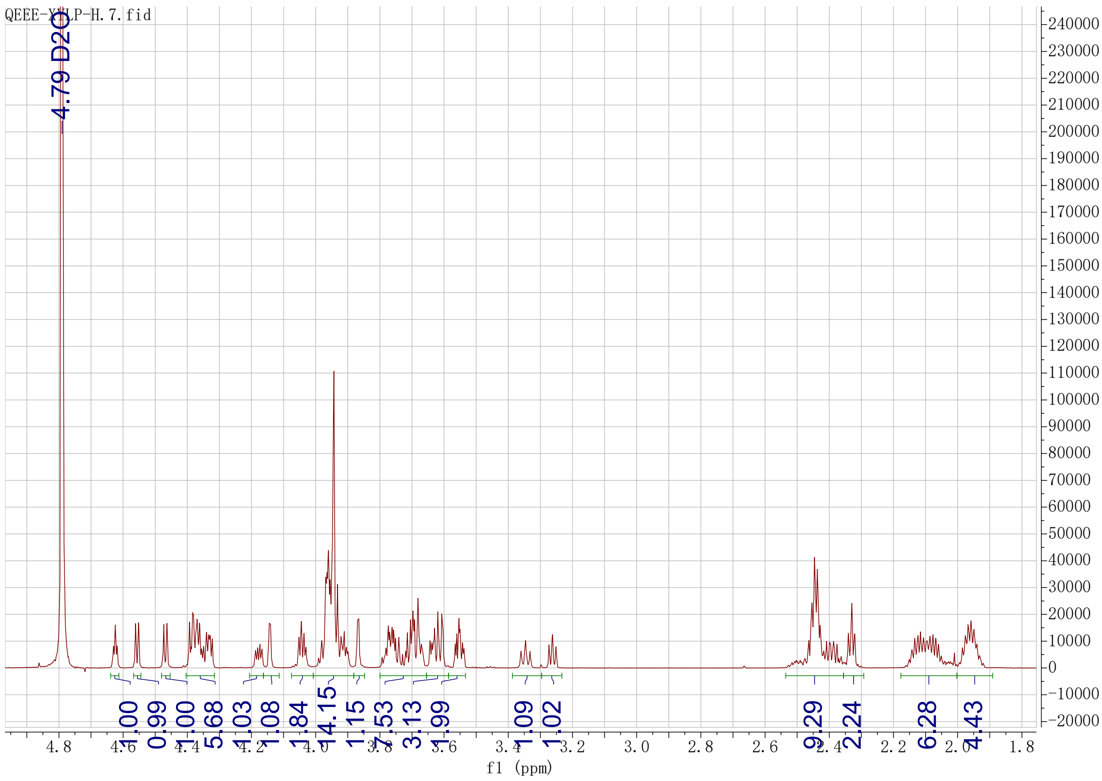
QEEE-XHLP-H.7.fid
4.79 D2O
f1 (ppm)

^{13}C NMR (201 MHz, D_2O)

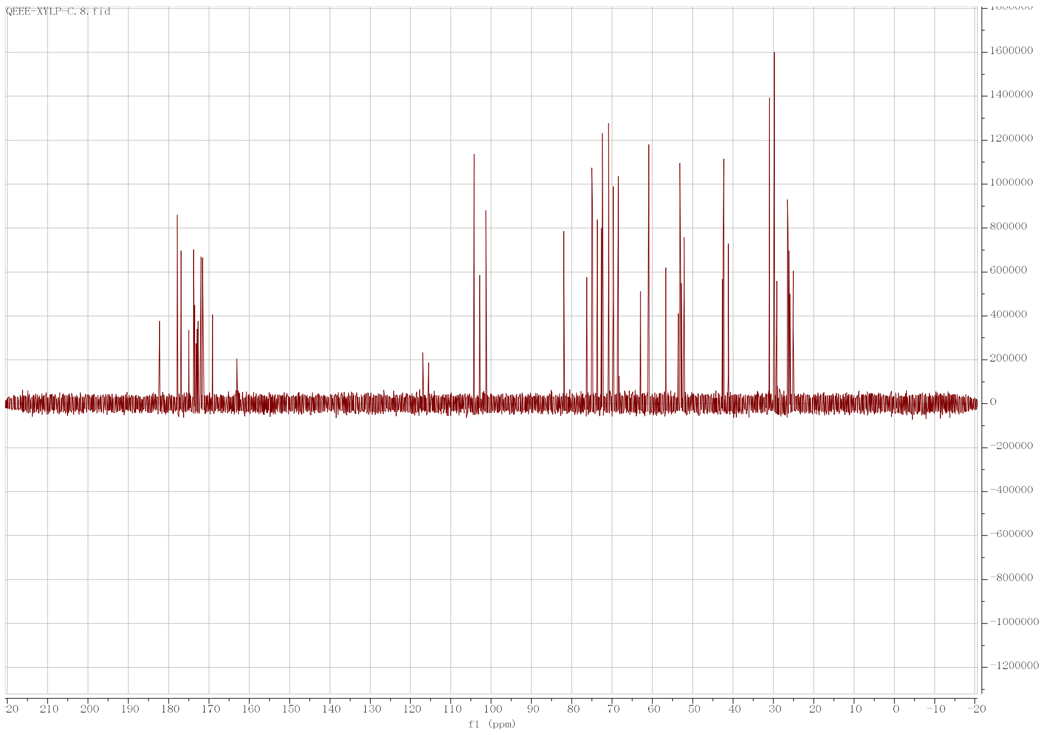

COSY (800 MHz, D_2O)

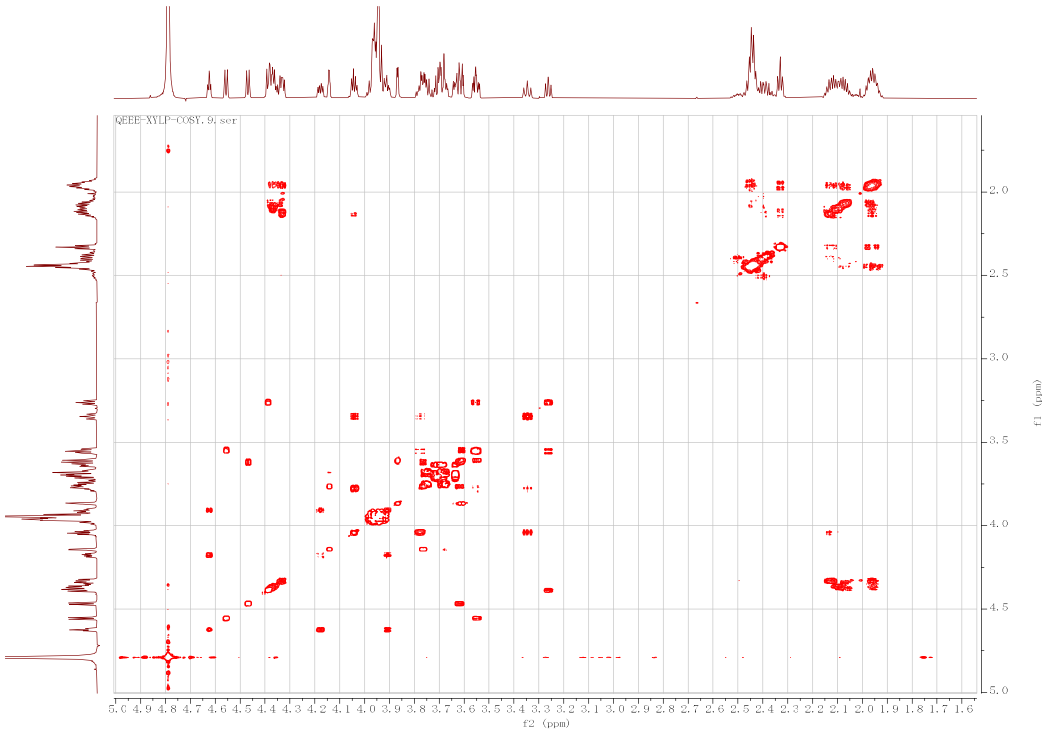

HSQC (800 MHz, D_2O)

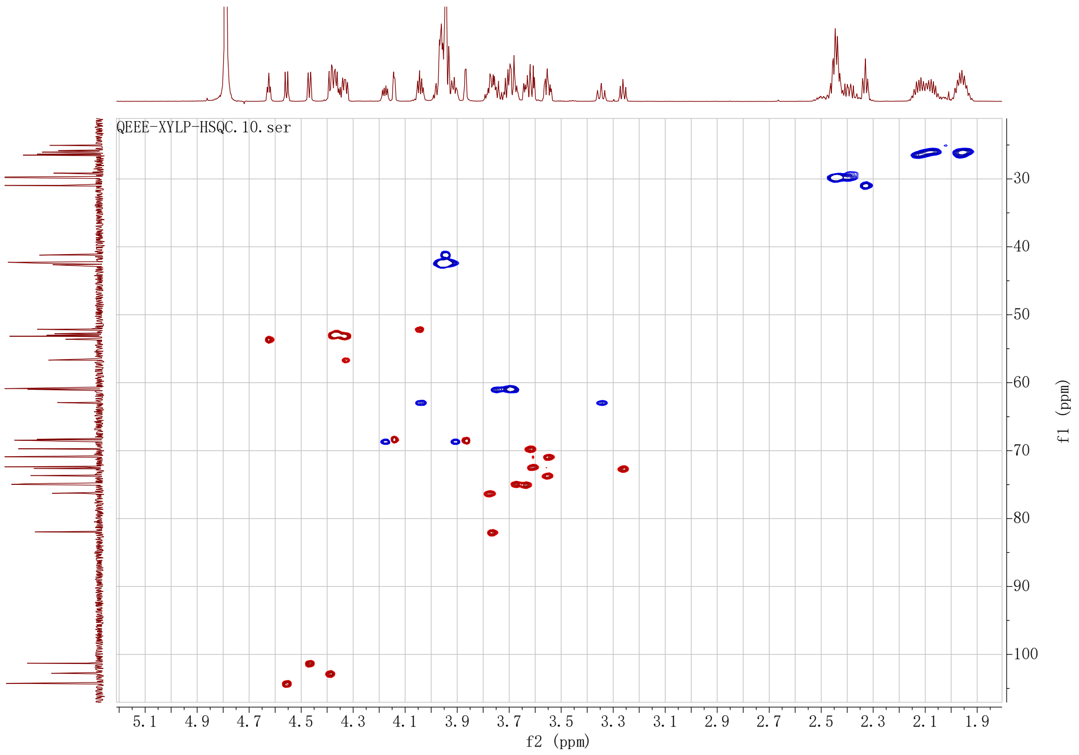

Coupled HSQC (800 MHz, D_2O)

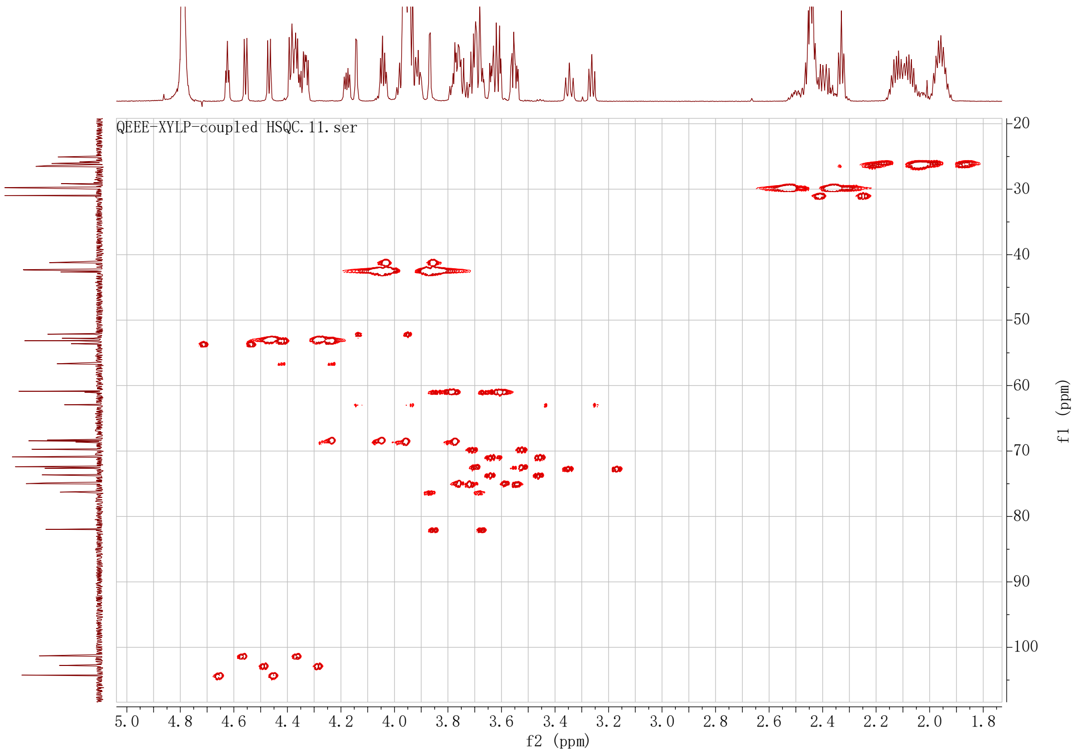

HMBC (800 MHz, D_2O)

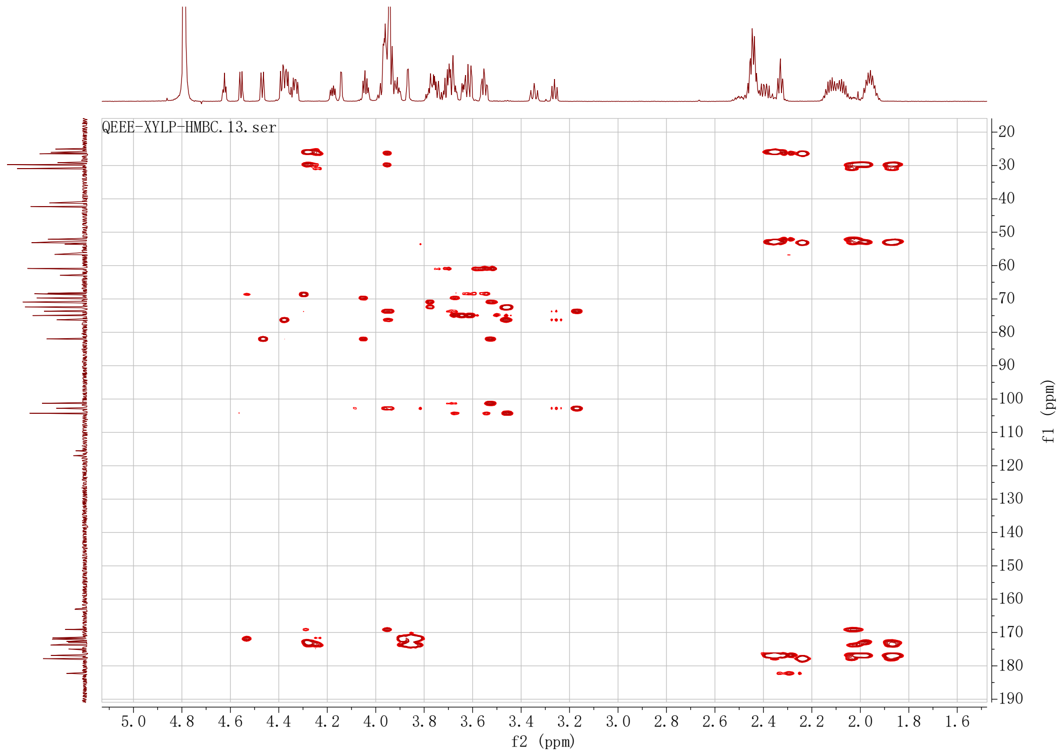

HPLC

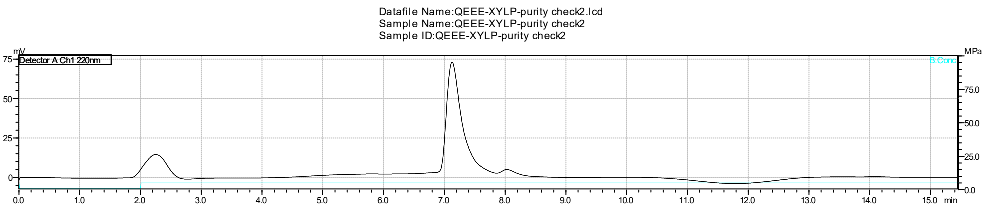

MS

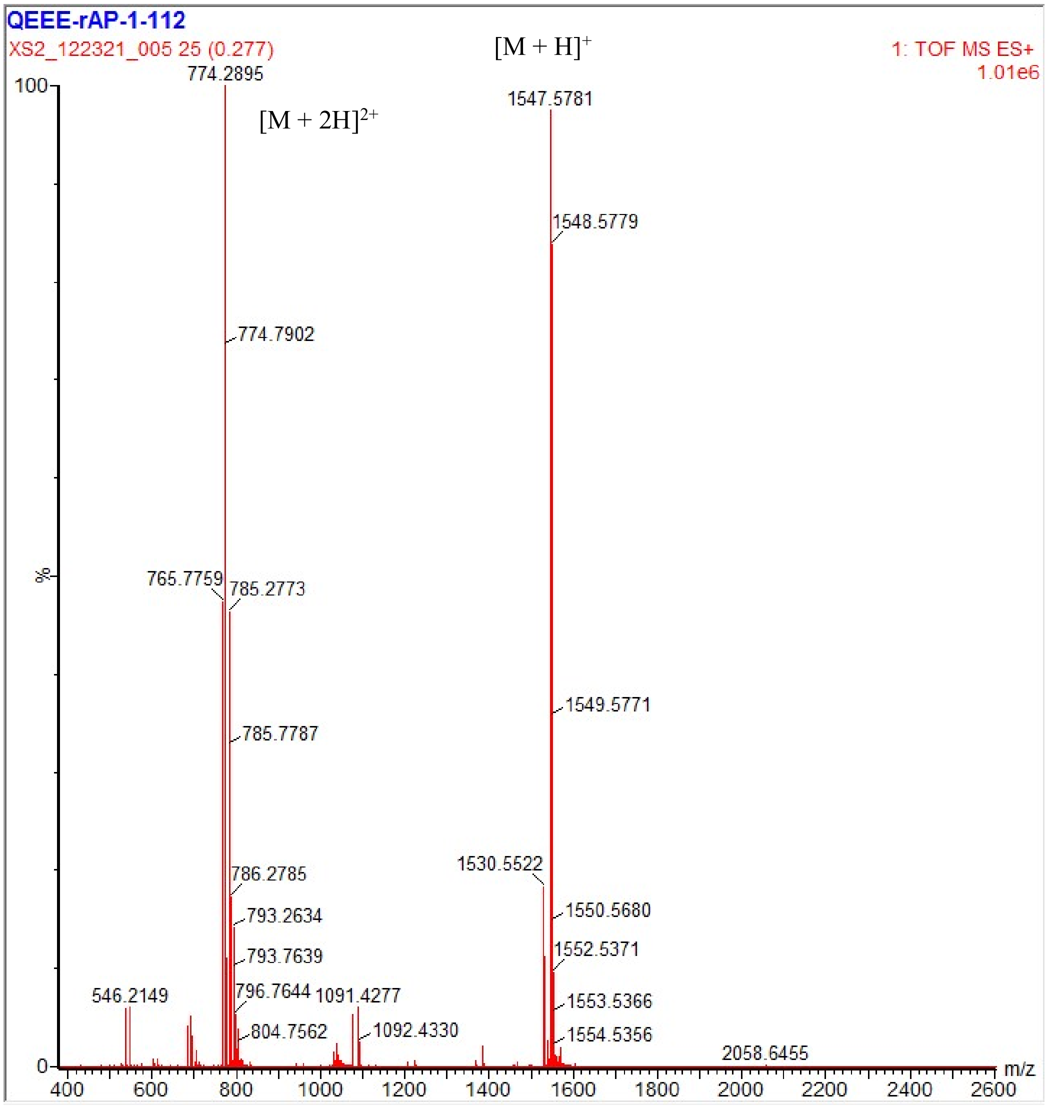
QEEE-rAP-1-112
XS2_122321_005 25 (0.277)
1: TOF MS ES+
1.01e6
[M + H]+
[M + 2H]2+
774.2895
1547.5781
1548.5779
774.7902
765.7759
785.2773
1549.5771
785.7787
786.2785
1530.5522
1550.5680
793.2634
1552.5371
793.7639
546.2149
796.7644
1091.4277
1553.5366
804.7562
1092.4330
1554.5356
2058.6455
100
%
0
400
600
800
1000
1200
1400
1600
1800
2000
2200
2400
2600
m/z

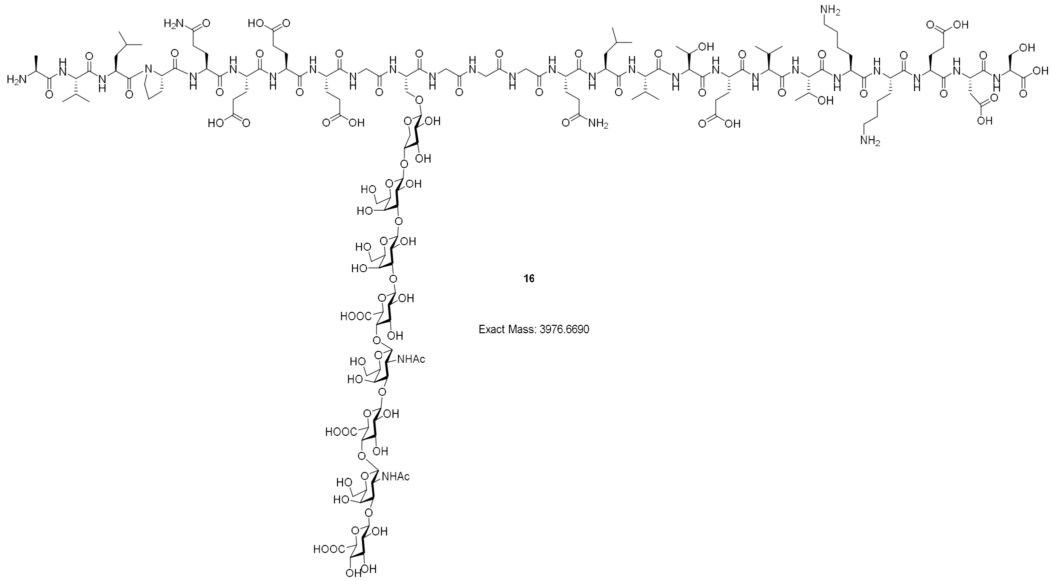

16

Exact Mass: 3976.6690

^{1}H NMR (600 MHz, D_2O)

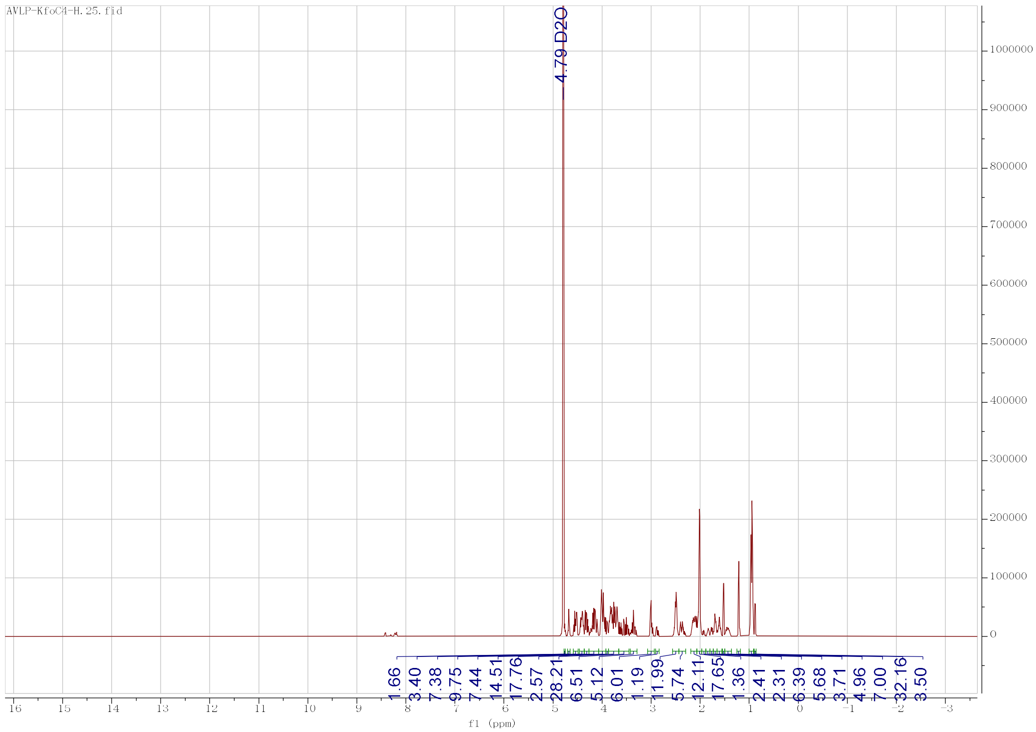

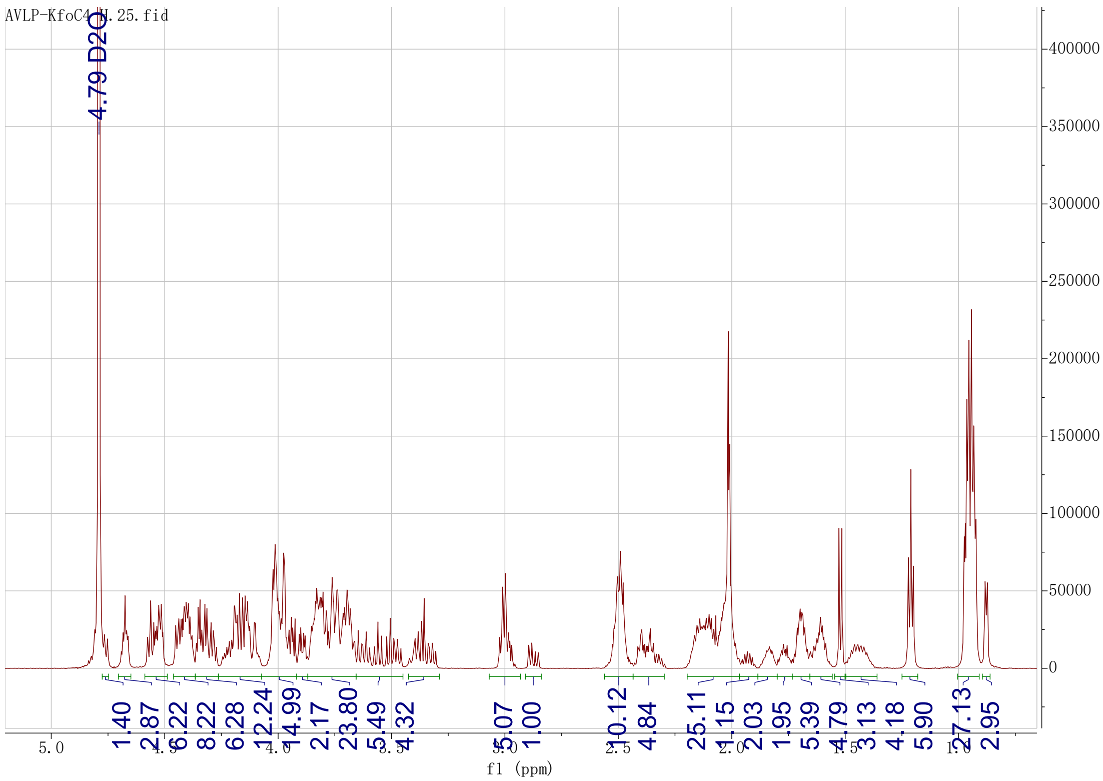

^{13}C NMR (151 MHz, D_2O)

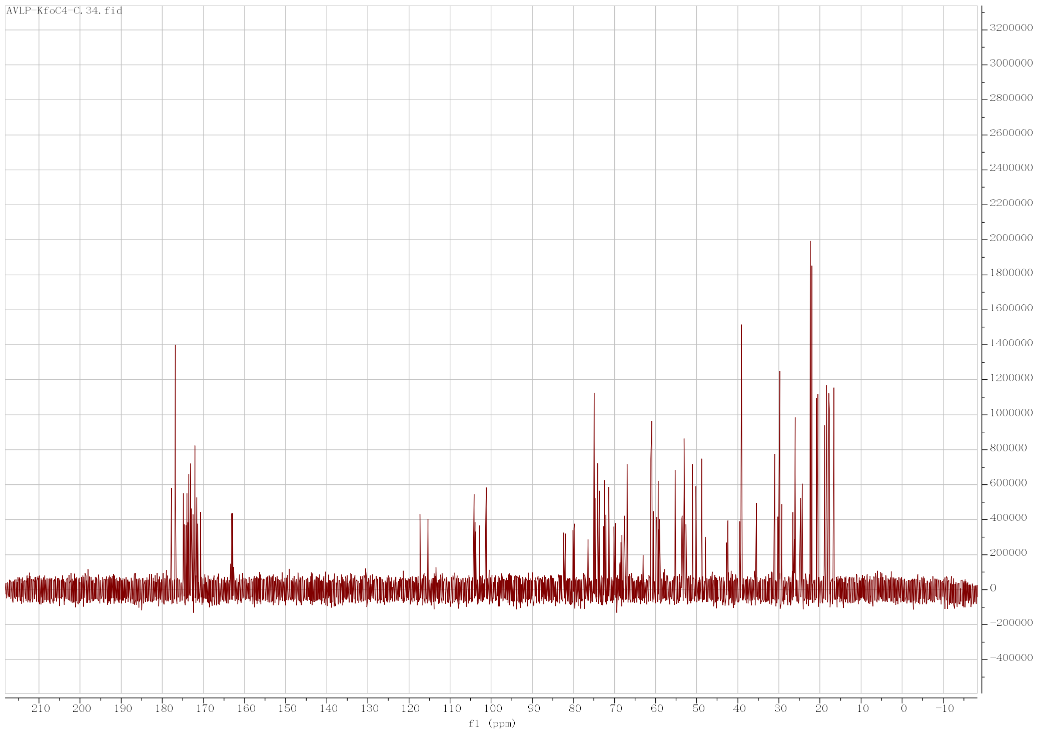

COSY (600 MHz, D_2O)

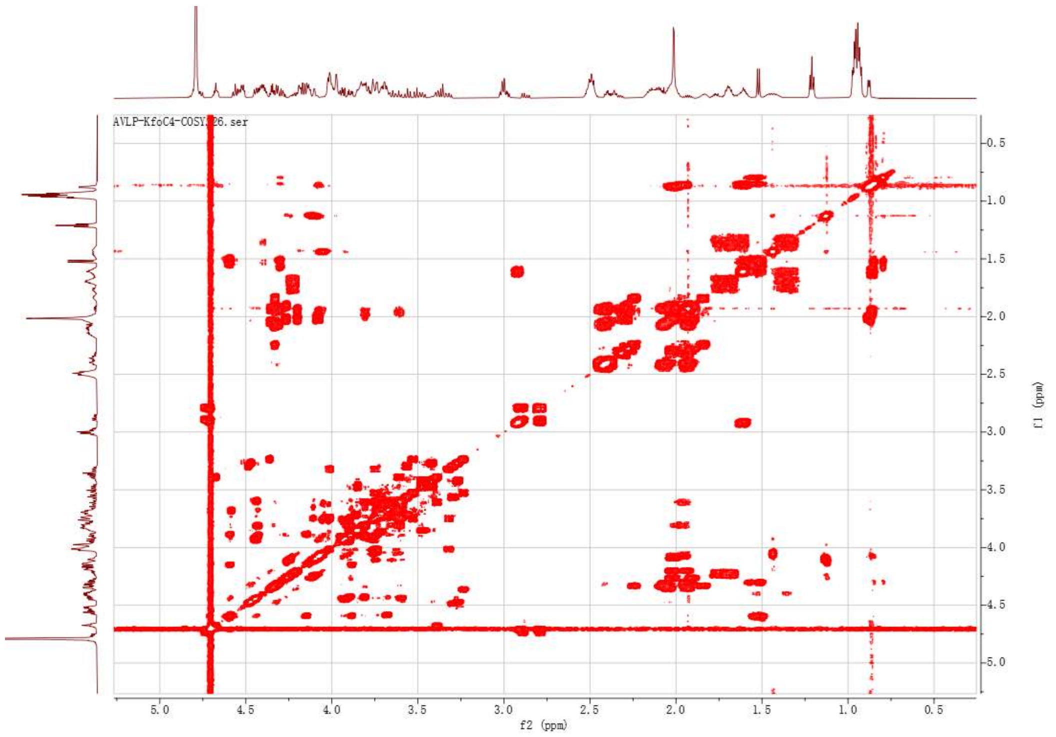

HSQC (600 MHz, D_2O)

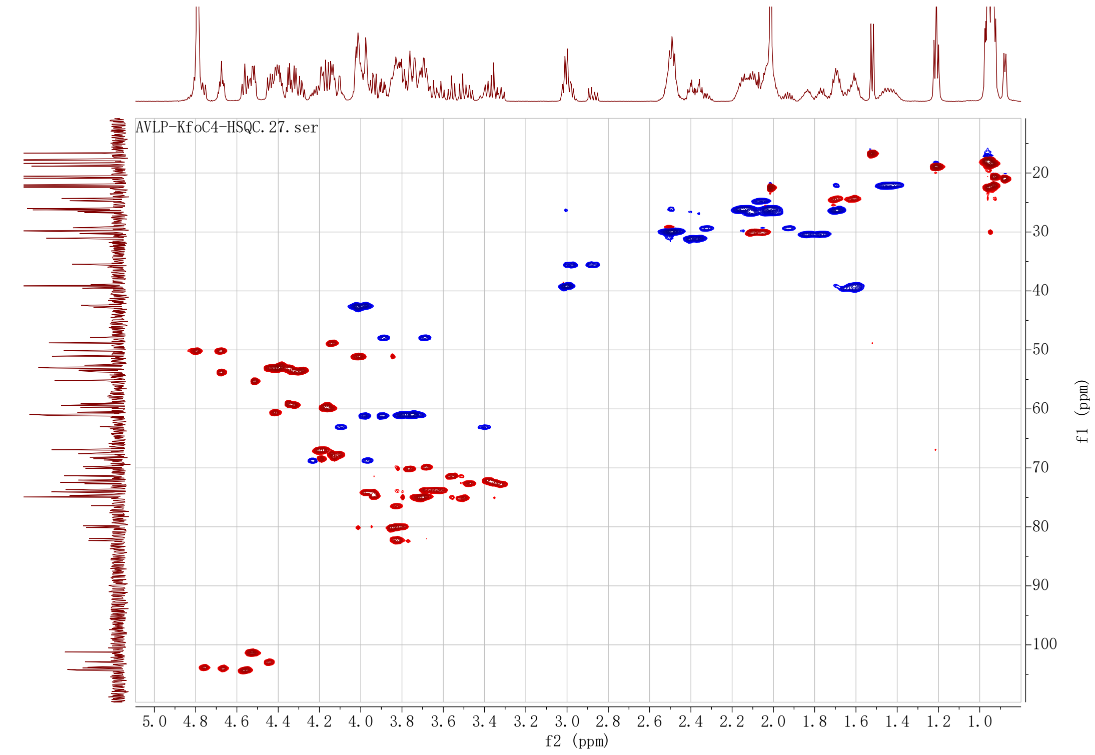

Coupled HSQC (600 MHz, D_2O)

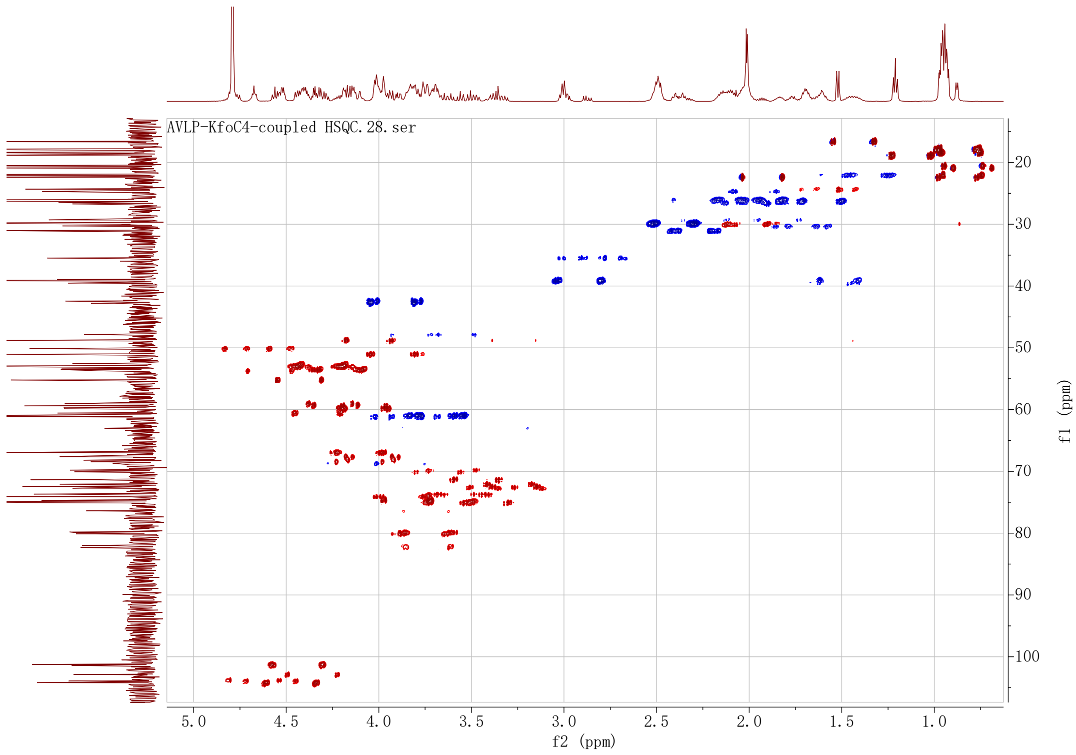

HMBC (600 MHz, D_2O)

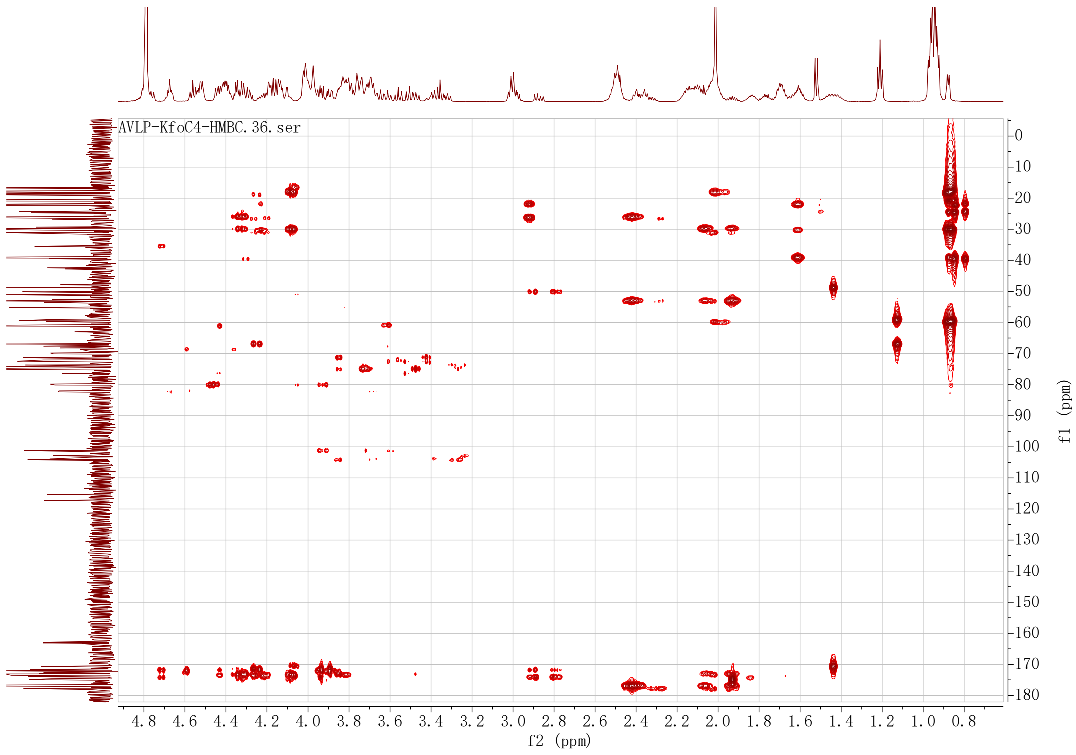

HPLC

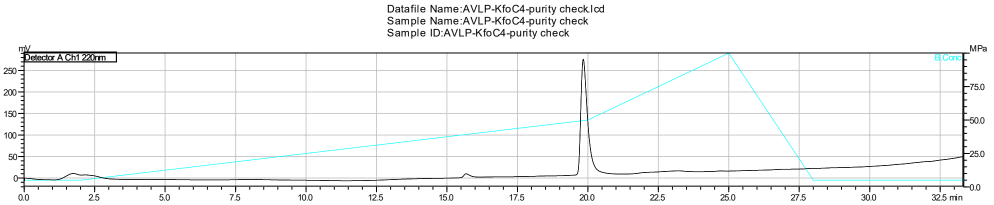

Datafile Name:AVLP-KfoC4-purity check.lcd
Sample Name:AVLP-KfoC4-purity check
Sample ID:AVLP-KfoC4-purity check
mV
MPa
Detector A Ch1 220nm
B.Conc
min

MS

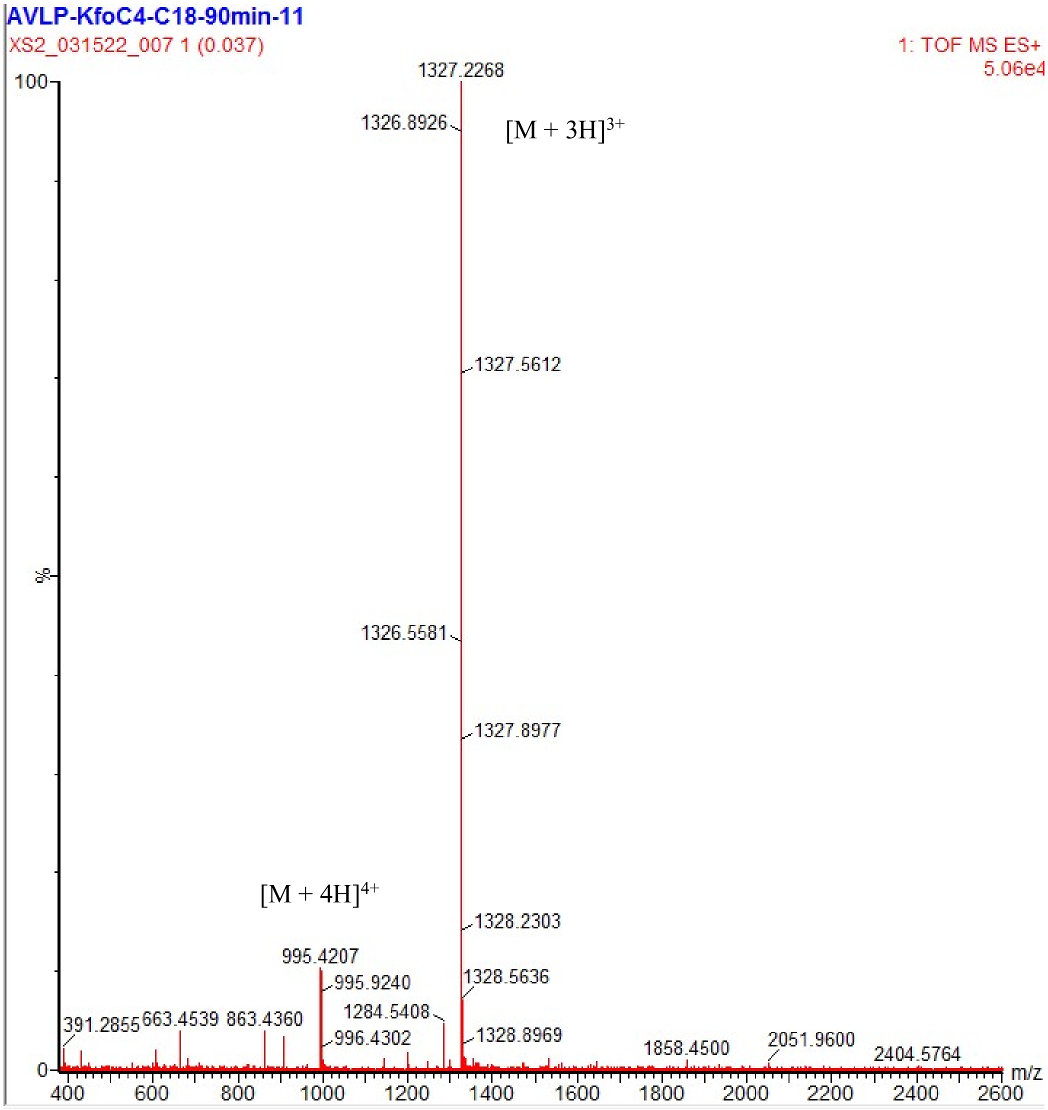

AVLP-KfoC4-C18-90min-11
XS2_031522_007 1 (0.037)
1: TOF MS ES+
5.06e4
1327.2268
1326.8926
[M + 3H]$^{3+}$
1327.5612
1326.5581
1327.8977
[M + 4H]$^{4+}$
1328.2303
995.4207
995.9240
1328.5636
1284.5408
391.2855
663.4539
863.4360
996.4302
1328.8969
1858.4500
2051.9600
2404.5764
%
m/z

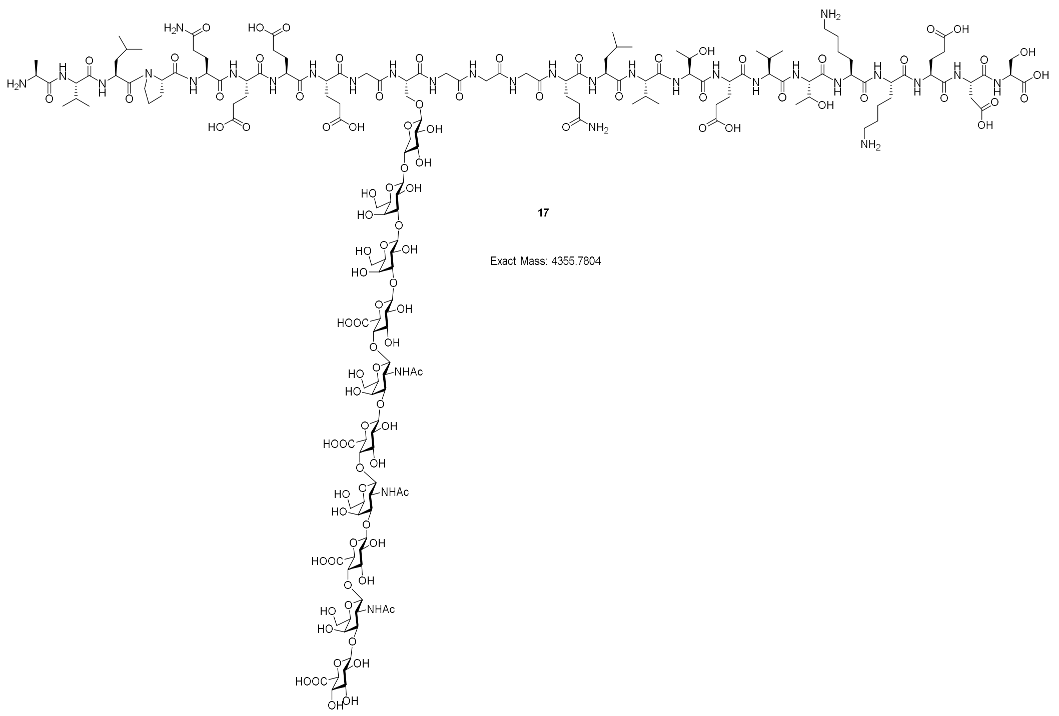

^{1}H NMR (800 MHz, D_2O)

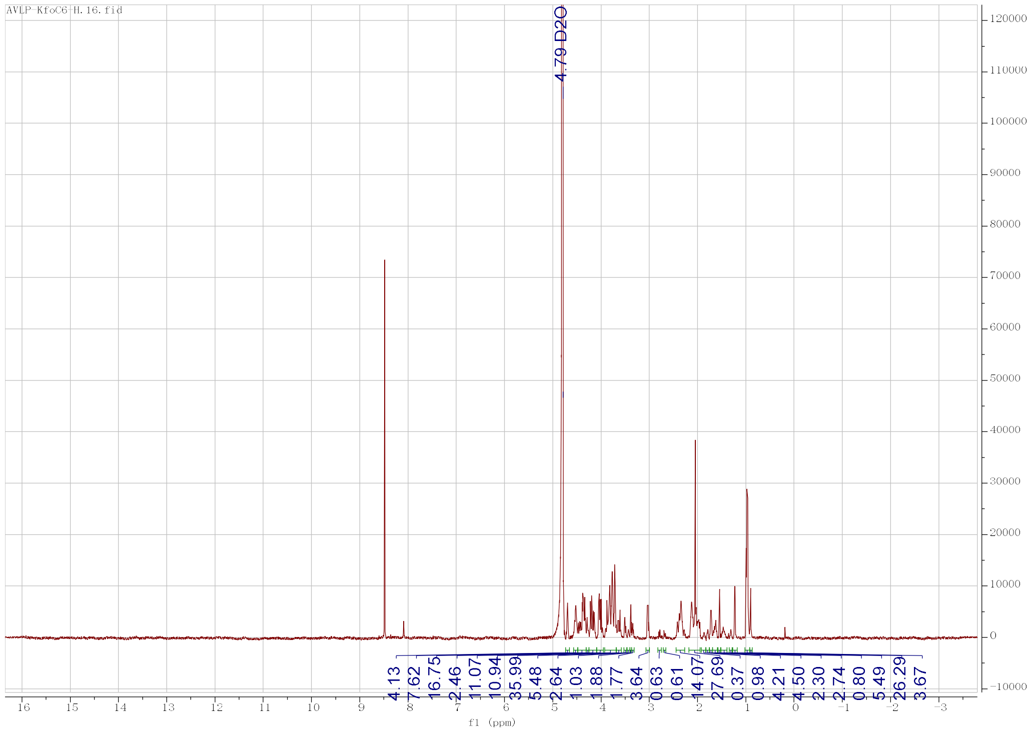

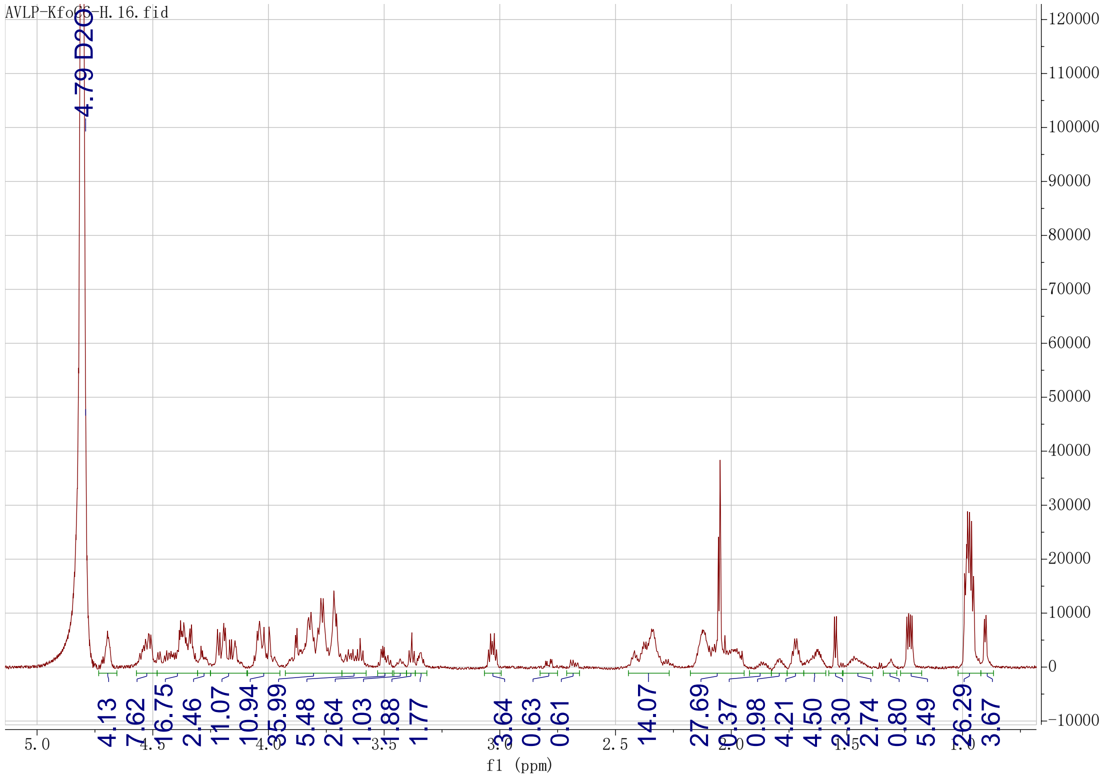
AVLP-Kfo[illegible]-H.16.fid
4.79 D2O
f1 (ppm)
4.13
7.62
16.75
2.46
11.07
10.94
35.99
5.48
2.64
1.03
1.88
1.77
3.64
0.63
0.61
14.07
27.69
0.37
0.98
4.21
4.50
2.30
2.74
0.80
5.49
26.29
3.67

COSY (800 MHz, D_2O)

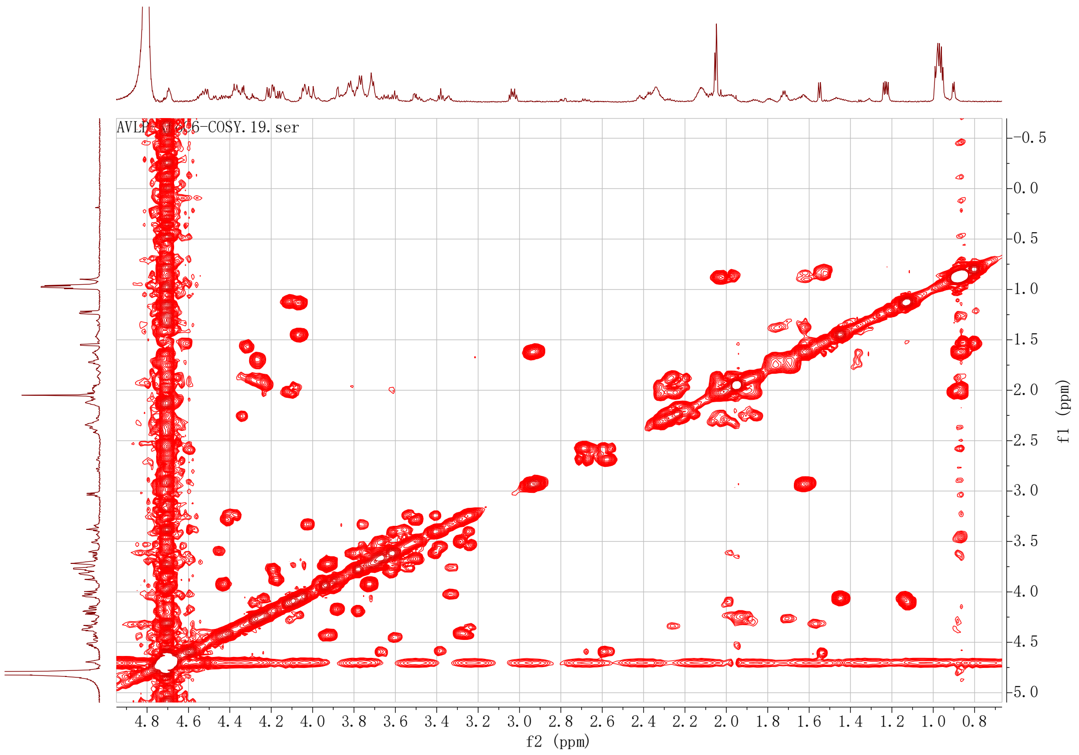

HSQC (800 MHz, D_2O)

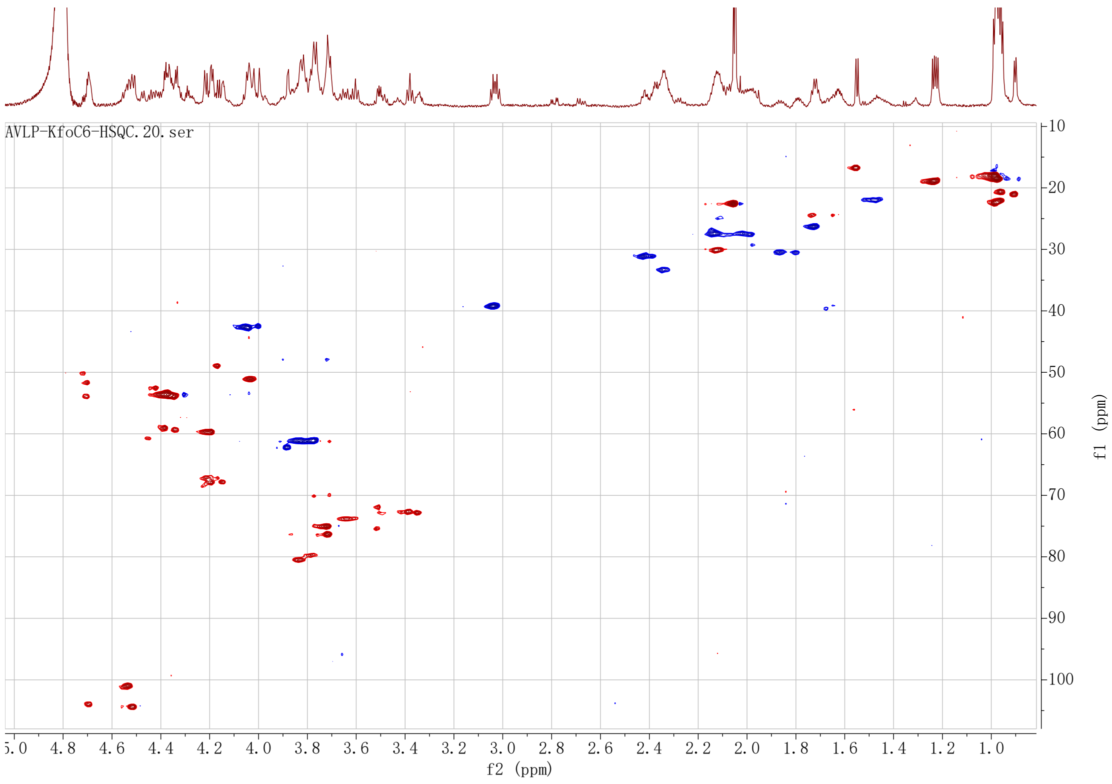

Coupled HSQC (800 MHz, D_2O)

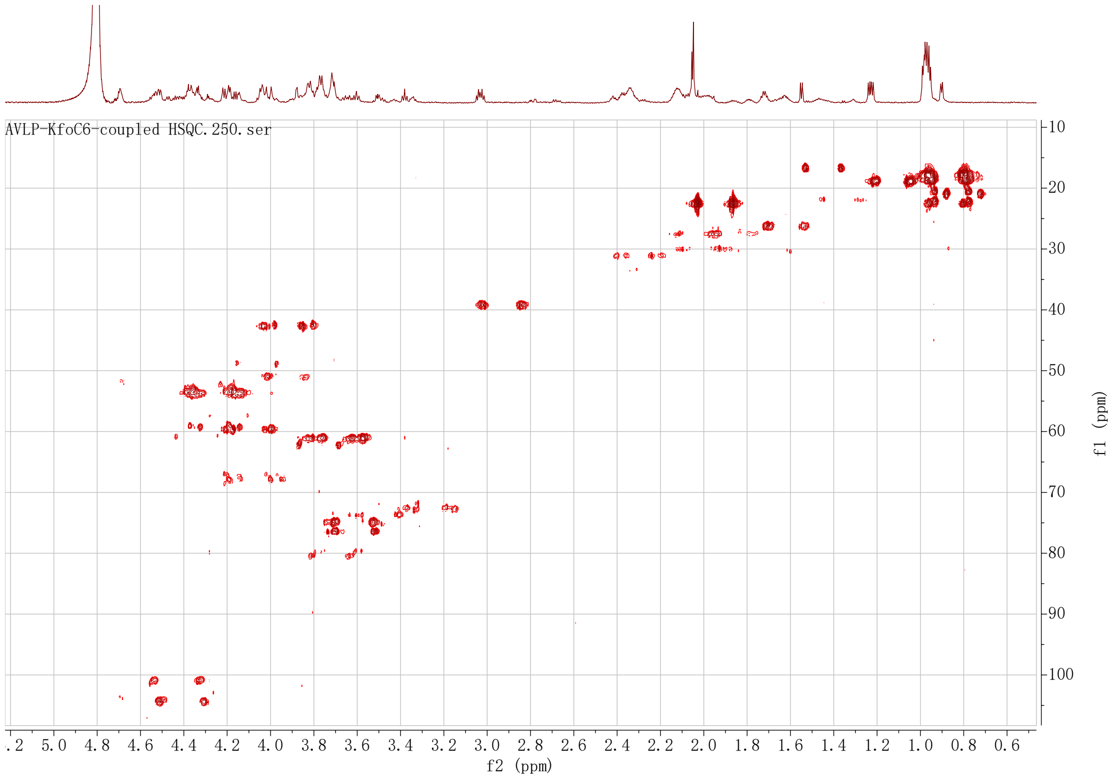

HPLC

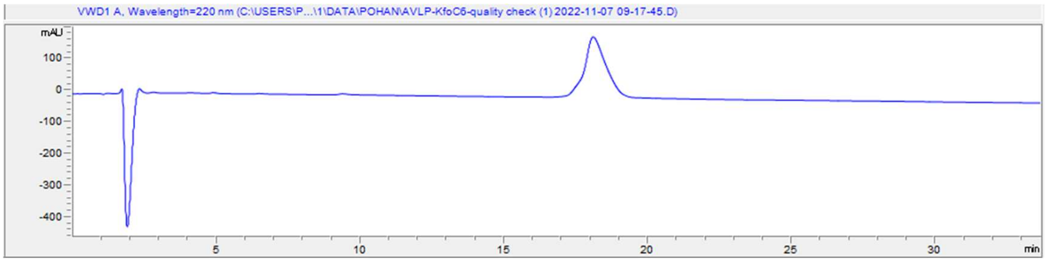

MS

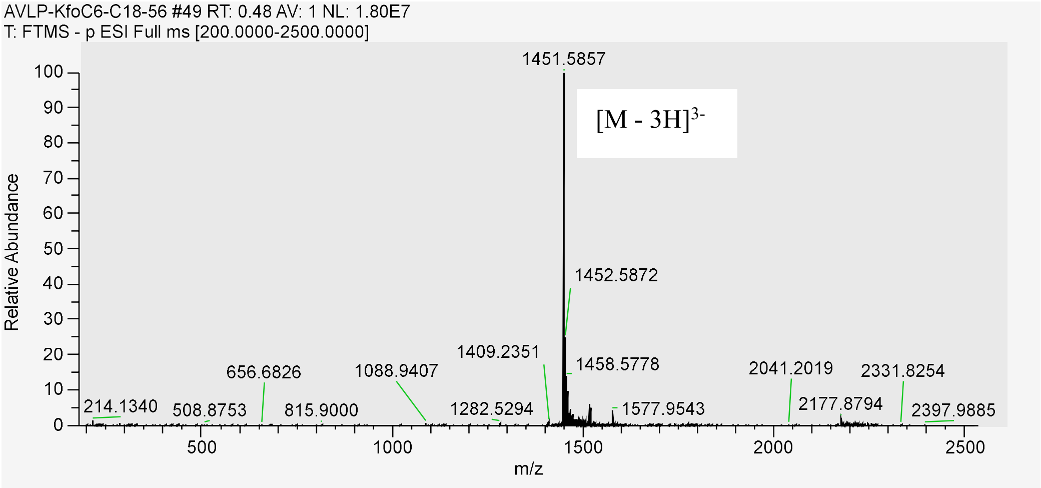
AVLP-KfoC6-C18-56 #49 RT: 0.48 AV: 1 NL: 1.80E7
T: FTMS - p ESI Full ms [200.0000-2500.0000]
[M - 3H]3-
1451.5857
1452.5872
1458.5778
1409.2351
1088.9407
656.6826
214.1340
508.8753
815.9000
1282.5294
1577.9543
2041.2019
2177.8794
2331.8254
2397.9885
Relative Abundance
m/z
0
10
20
30
40
50
60
70
80
90
100
500
1000
1500
2000
2500

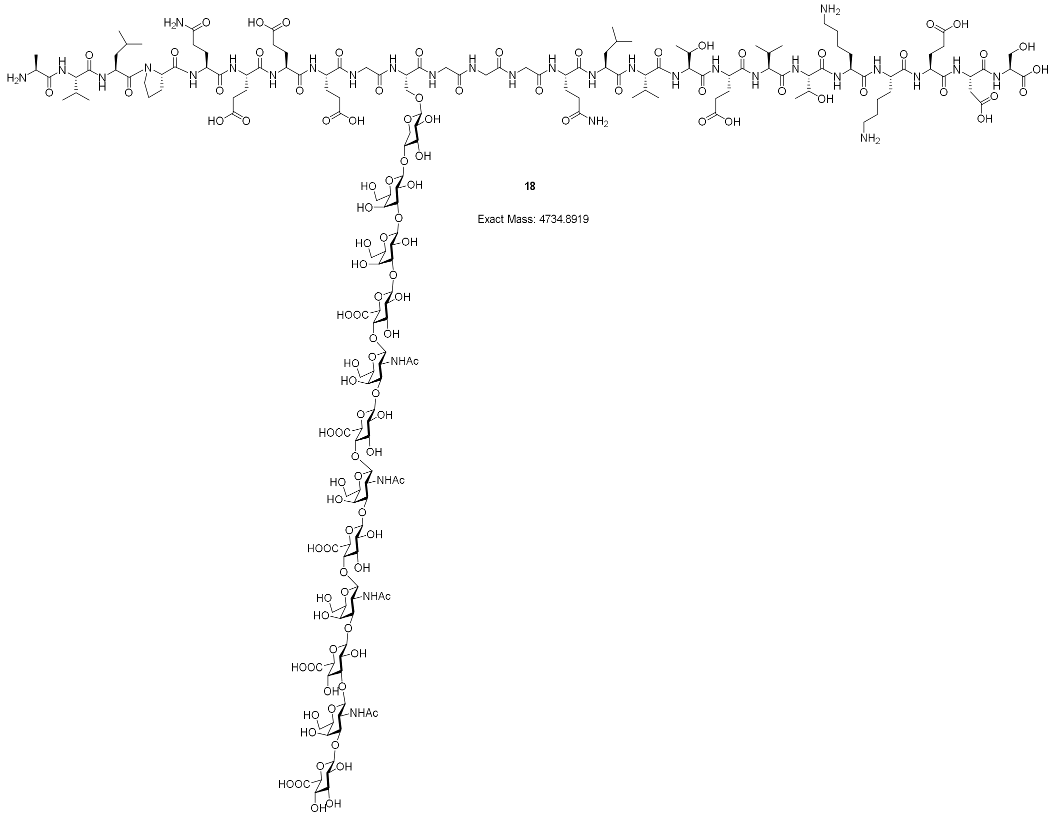

18

Exact Mass: 4734.8919

^1H NMR (800 MHz, D_2O)

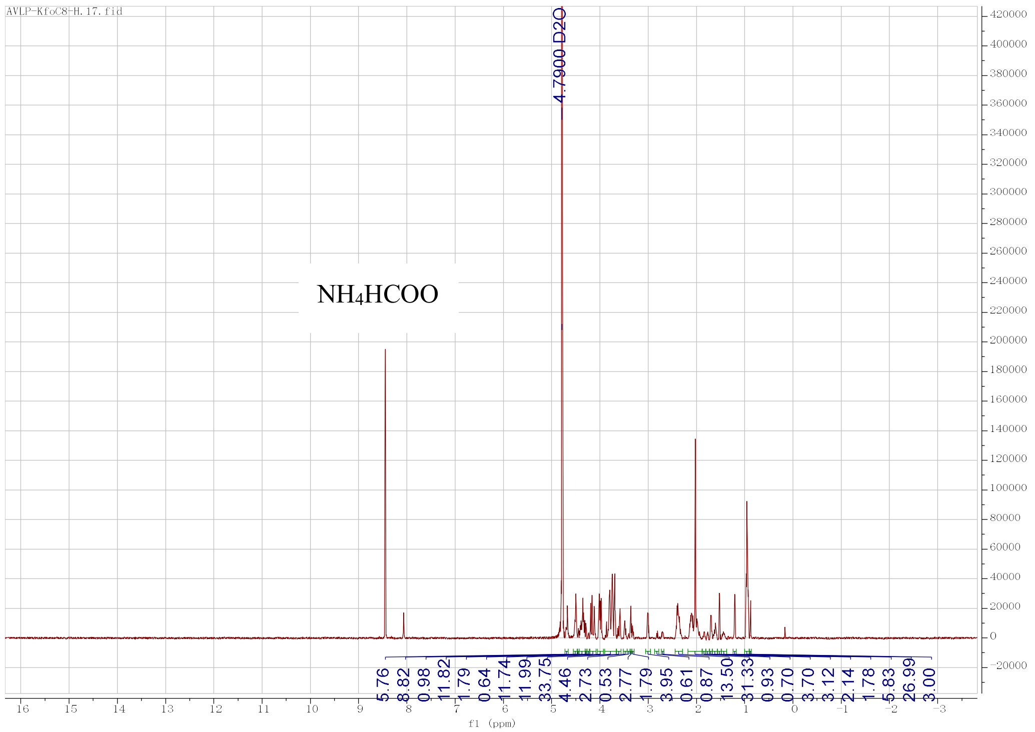

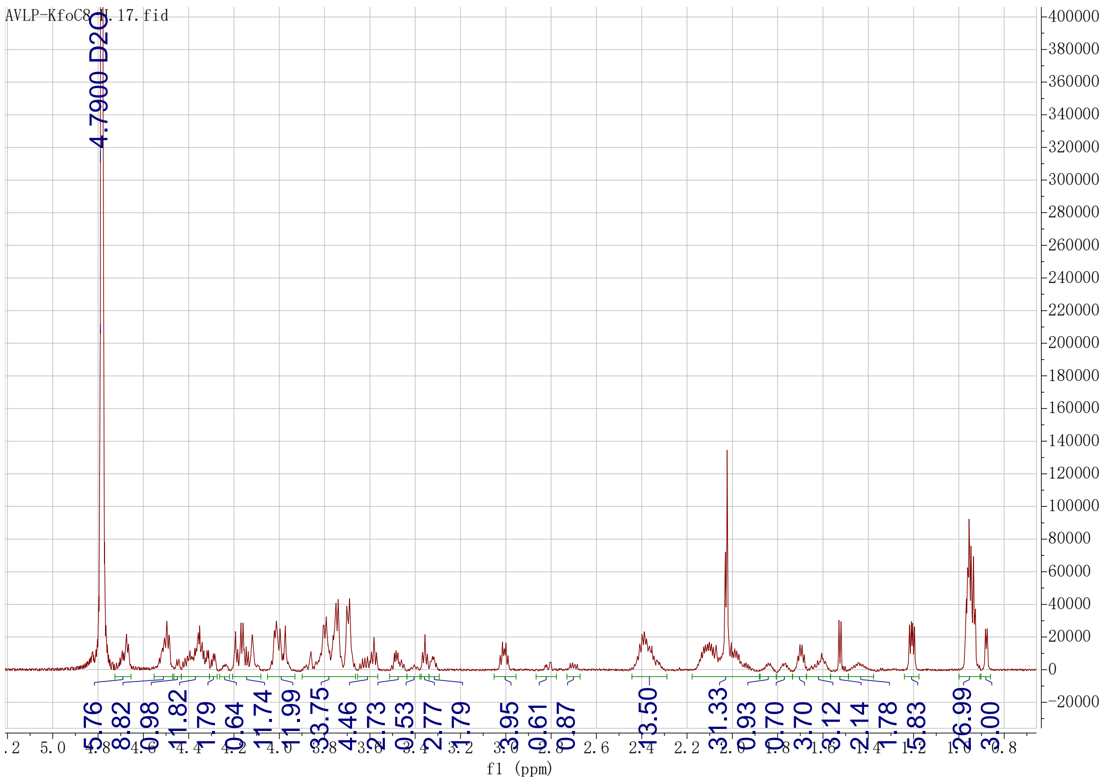

^{13}C NMR (201 MHz, D_2O)

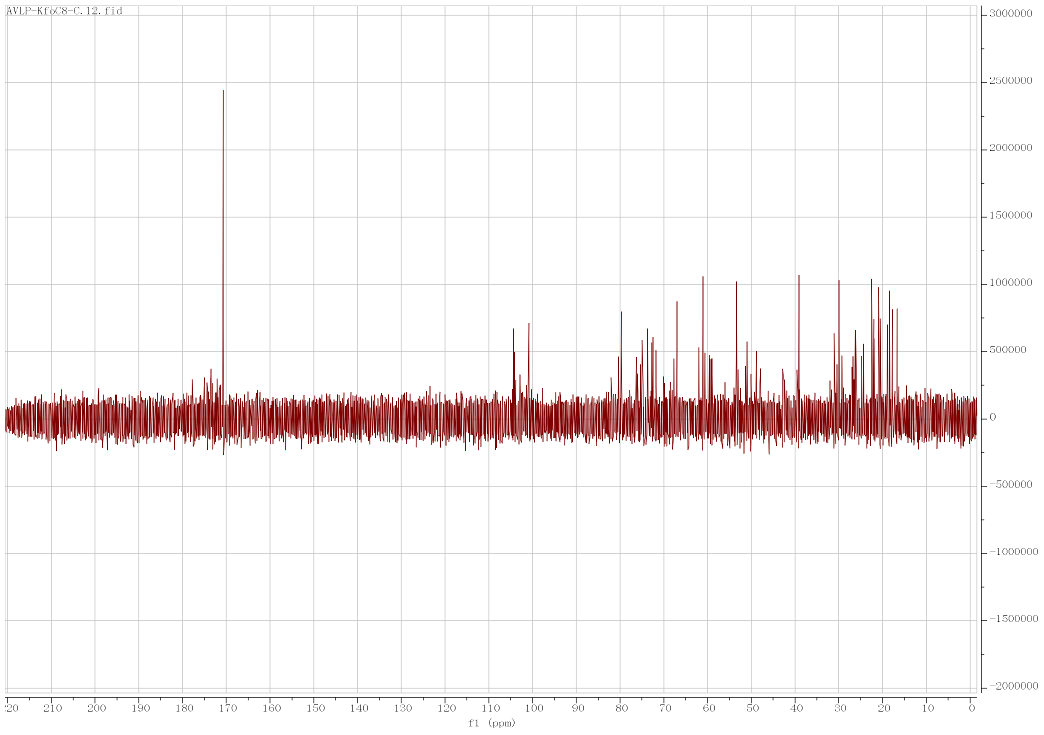

COSY (800 MHz, D_2O)

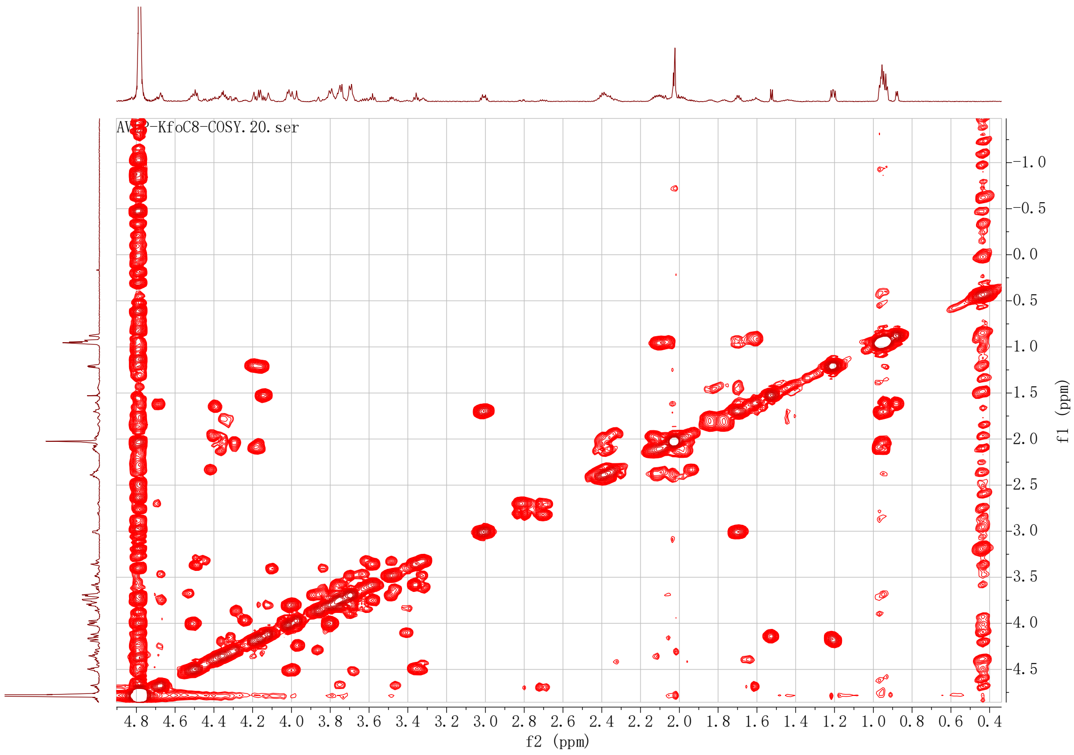

HSQC (800 MHz, D_2O)

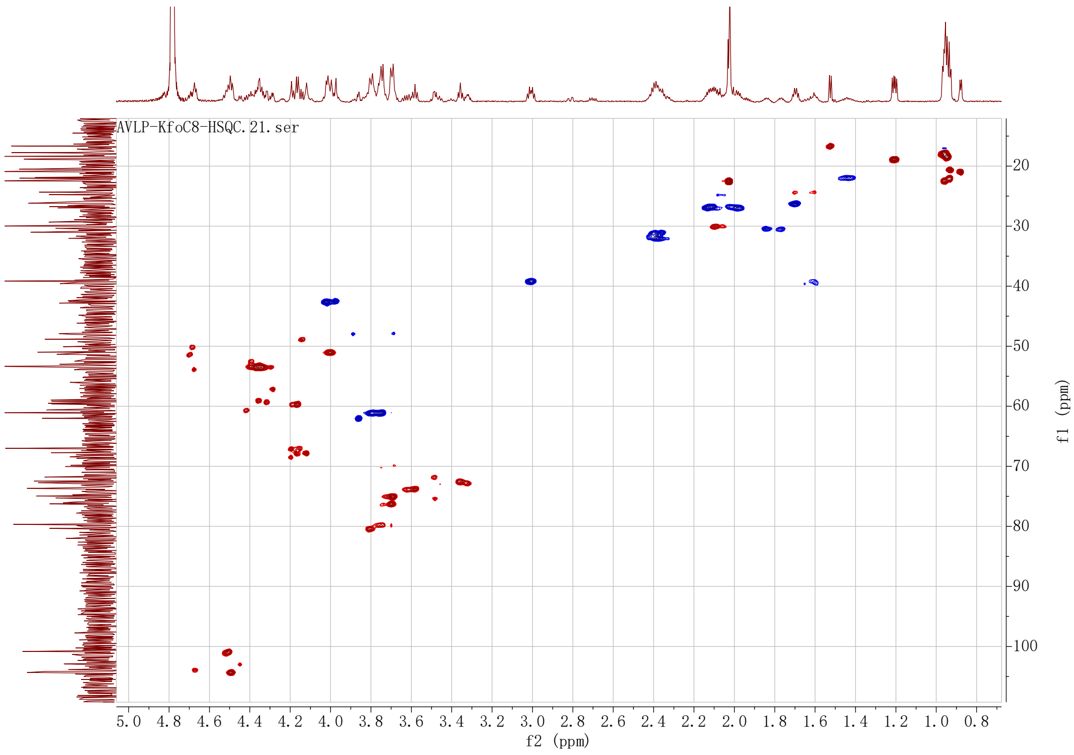

Coupled HSQC (800 MHz, D_2O)

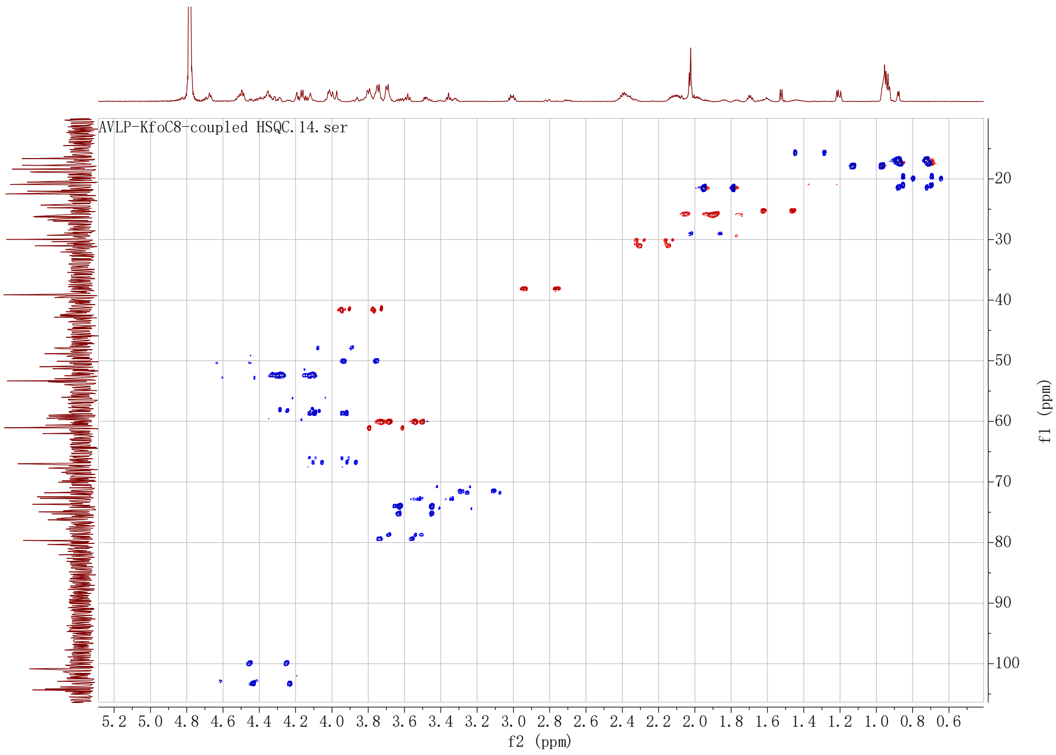

HMBC (800 MHz, D_2O)

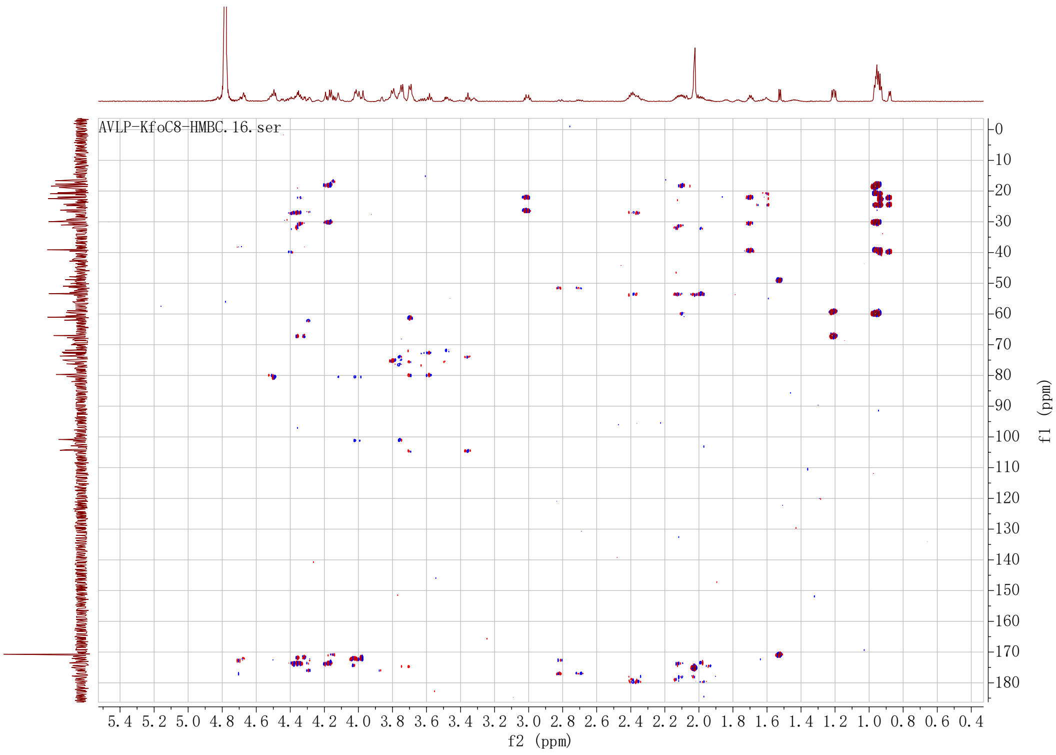

HPLC

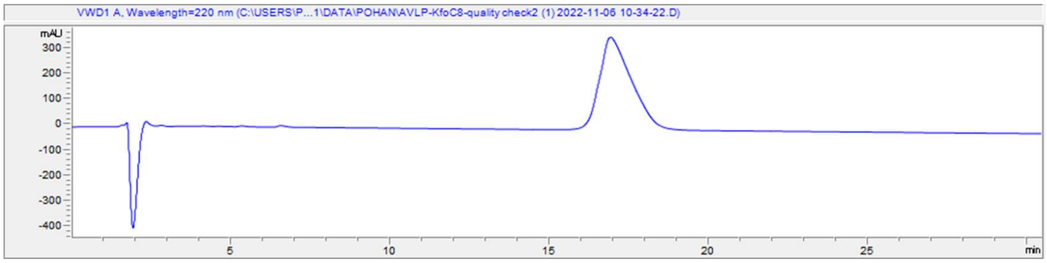
VWD1 A, Wavelength=220 nm (C:\USERS\P...1\DATA\POHAN\AVLP-KfoC8-quality check2 (1) 2022-11-06 10-34-22.D)
mAU
300
200
100
0
-100
-200
-300
-400
5
10
15
20
25
min

MS

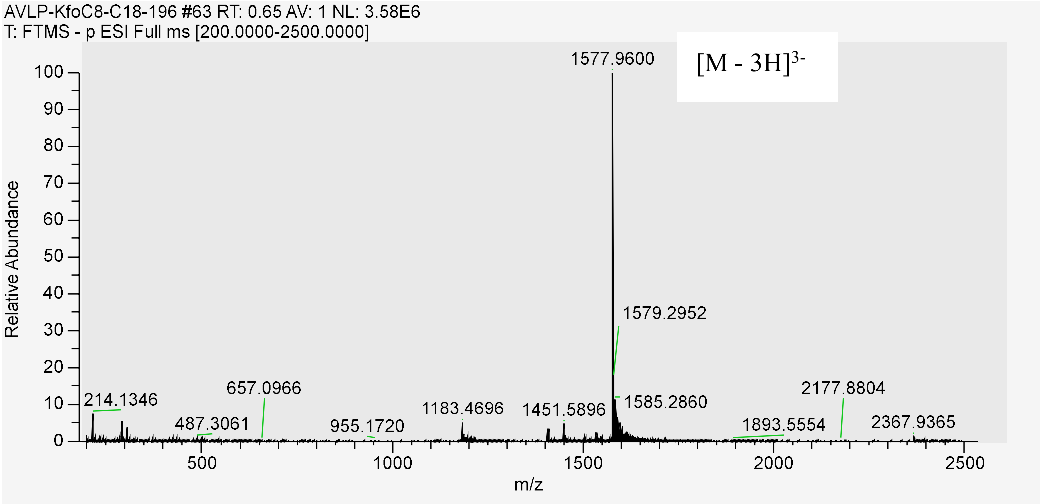
AVLP-KfoC8-C18-196 #63 RT: 0.65 AV: 1 NL: 3.58E6
T: FTMS - p ESI Full ms [200.0000-2500.0000]
[M - 3H]3-
1577.9600
1579.2952
1585.2860
214.1346
657.0966
487.3061
955.1720
1183.4696
1451.5896
2177.8804
1893.5554
2367.9365
Relative Abundance
m/z

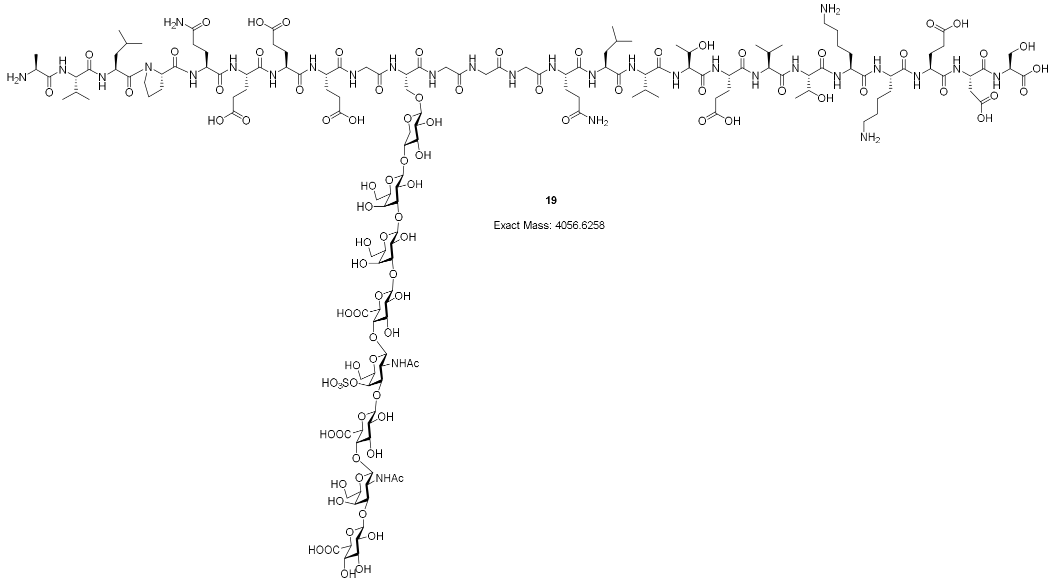

19

Exact Mass: 4056.6258

^{1}H NMR (800 MHz, D_2O)

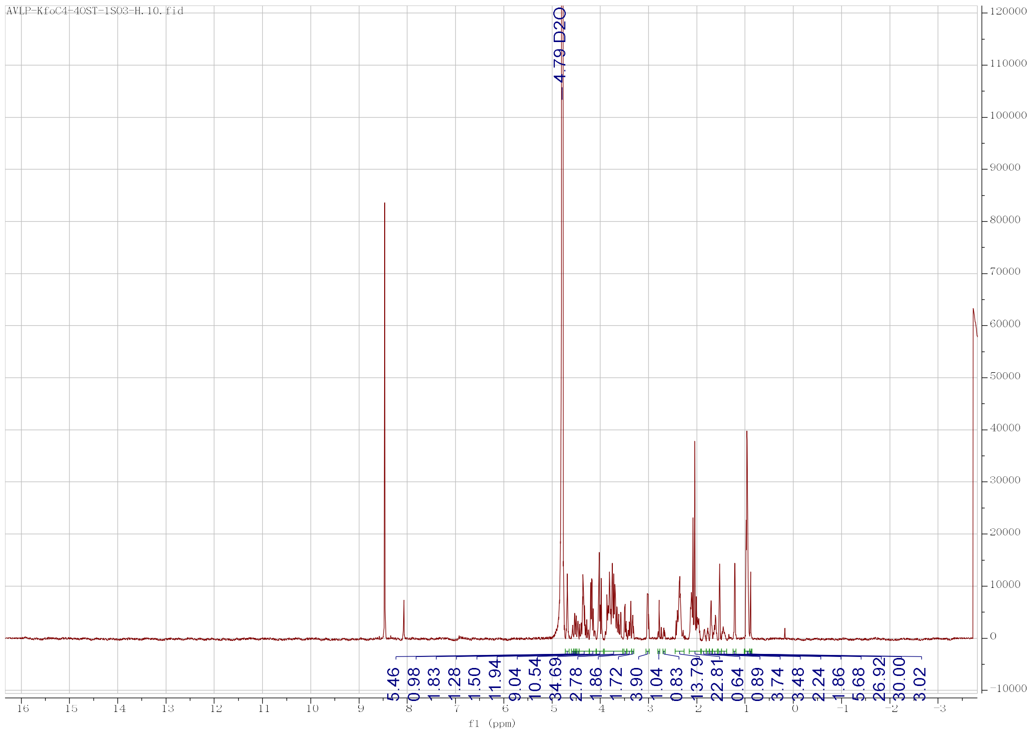

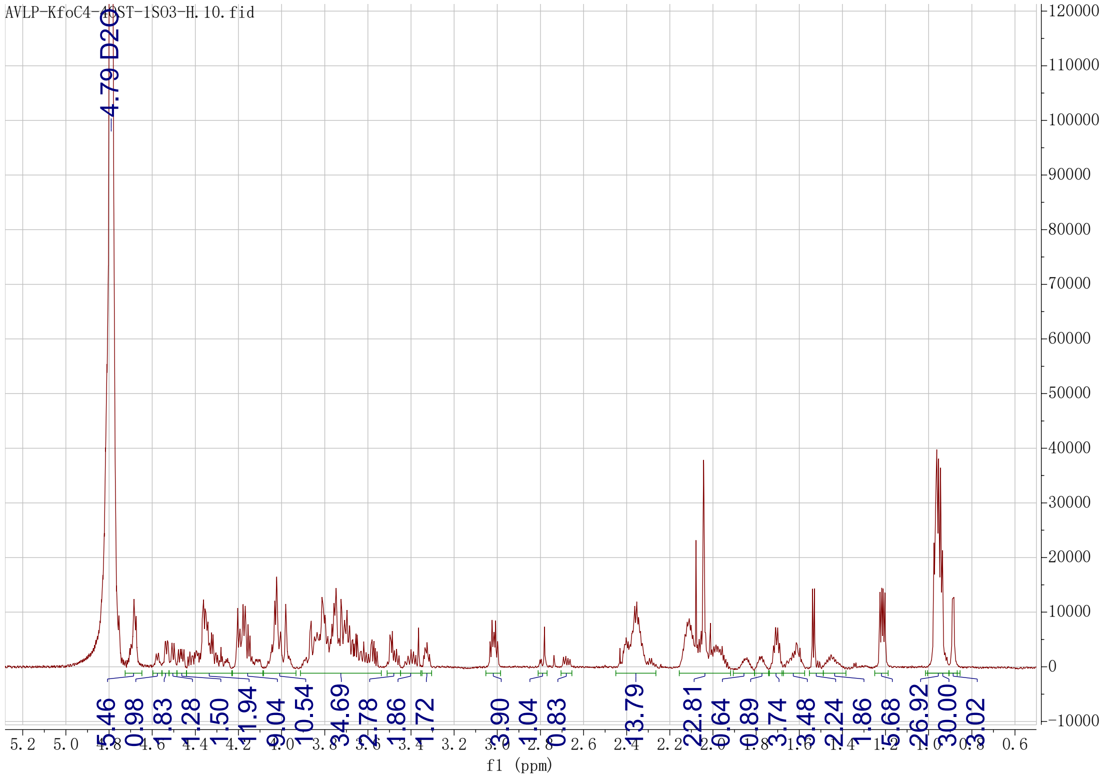
AVLP-KfoC4-[illegible]ST-1SO3-H.10.fid
4.79 D2O
5.46
0.98
1.83
1.28
1.50
11.94
9.04
10.54
34.69
2.78
1.86
1.72
3.90
1.04
0.83
13.79
22.81
0.64
0.89
3.74
3.48
2.24
1.86
15.68
26.92
30.00
3.02
f1 (ppm)

^{13}C NMR (201 MHz, D_2O)

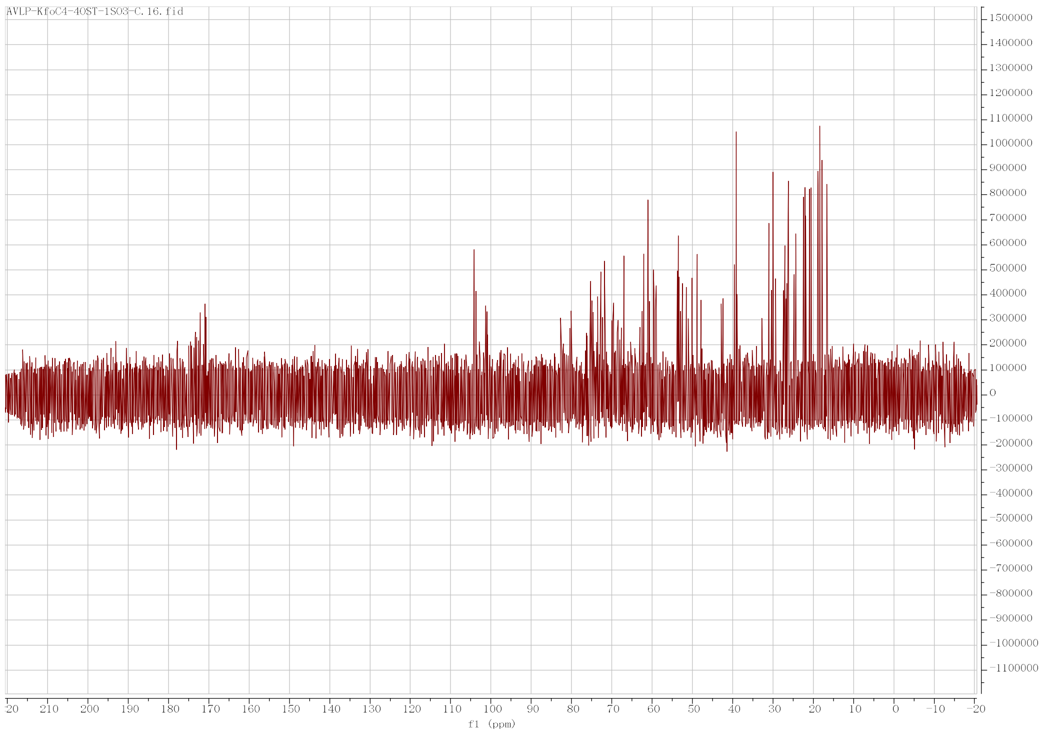

COSY (800 MHz, D_2O)

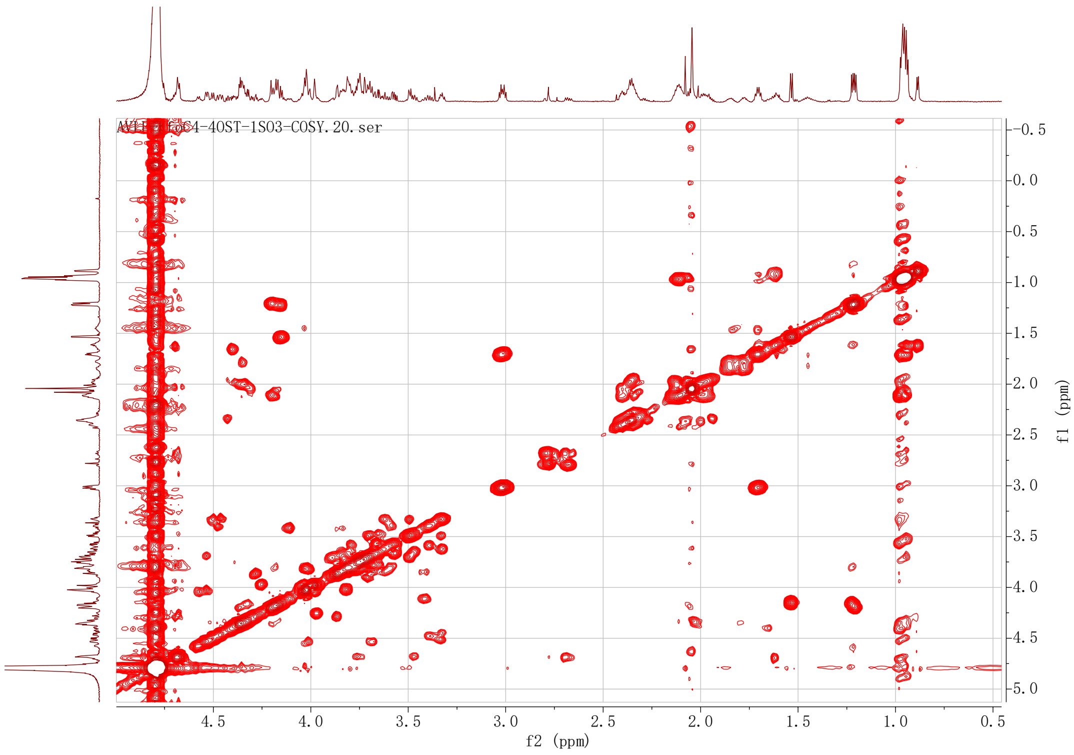

HSQC (800 MHz, D_2O)

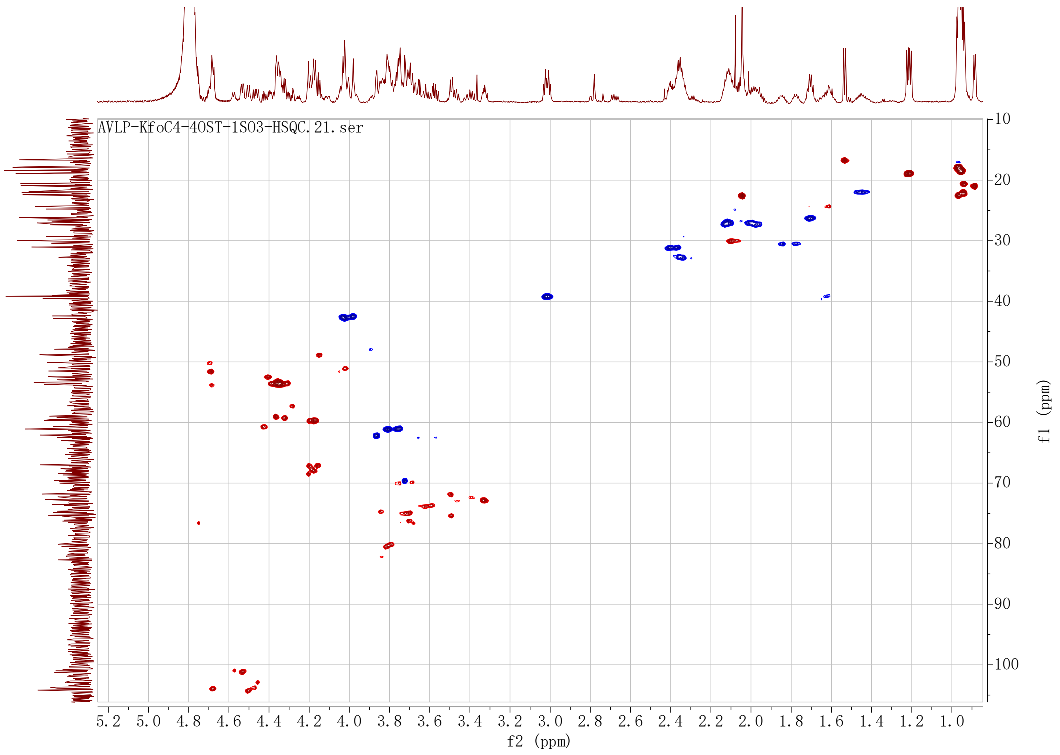

Coupled HSQC (800 MHz, D_2O)

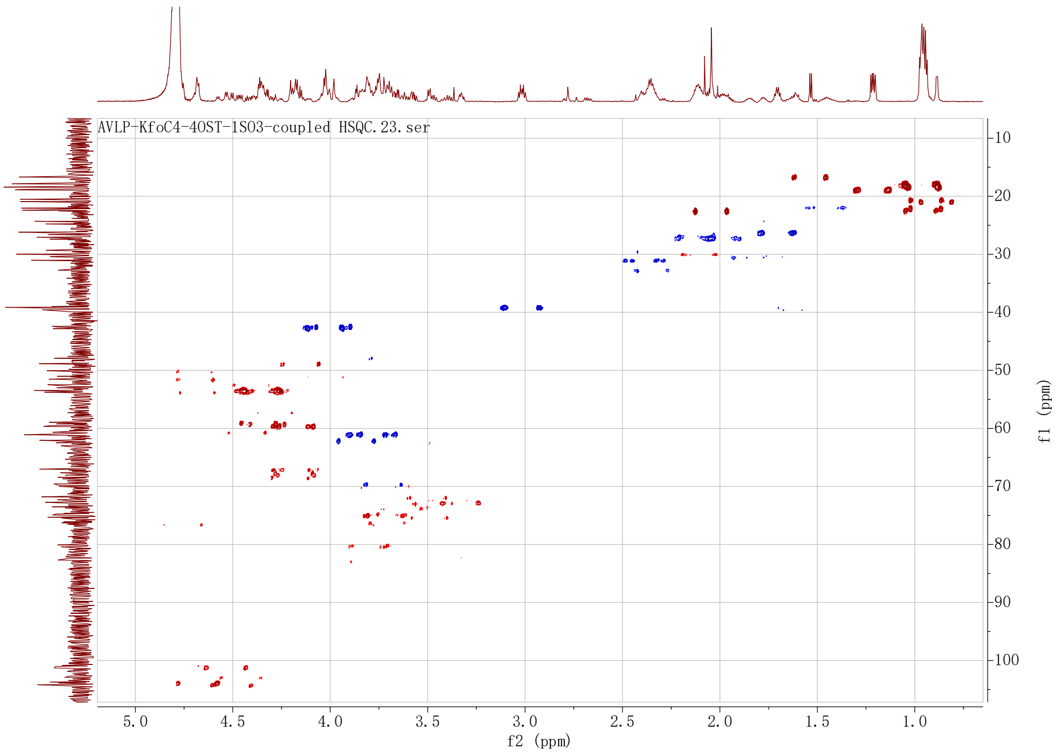

HMBC (800 MHz, D_2O)

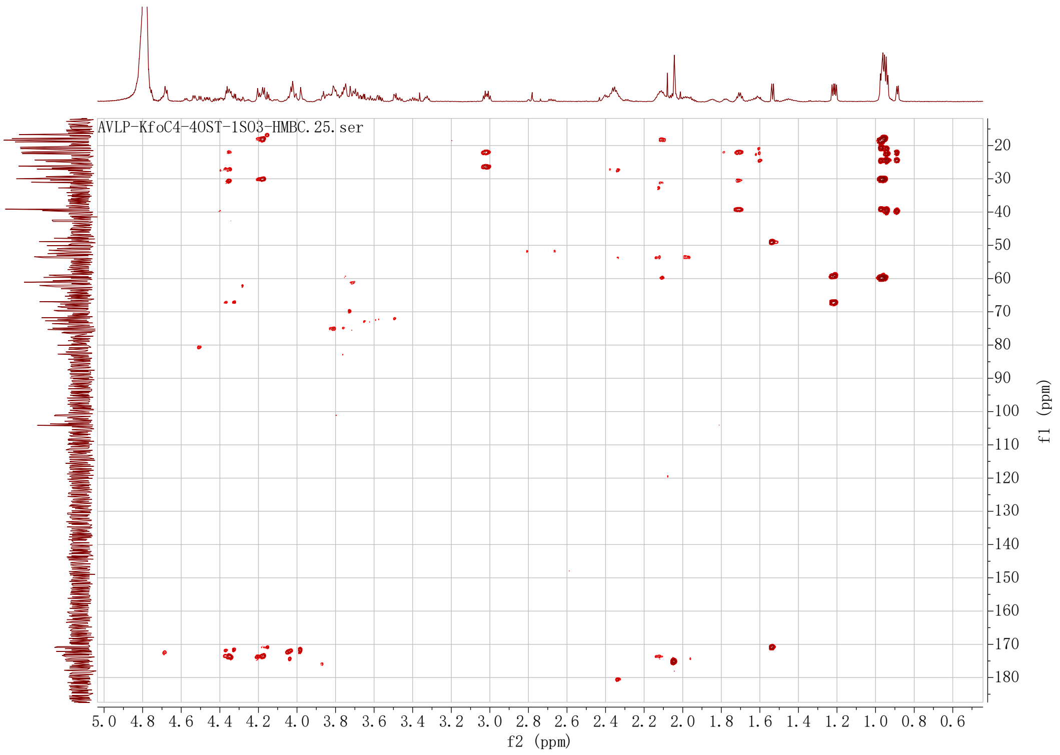

HPLC

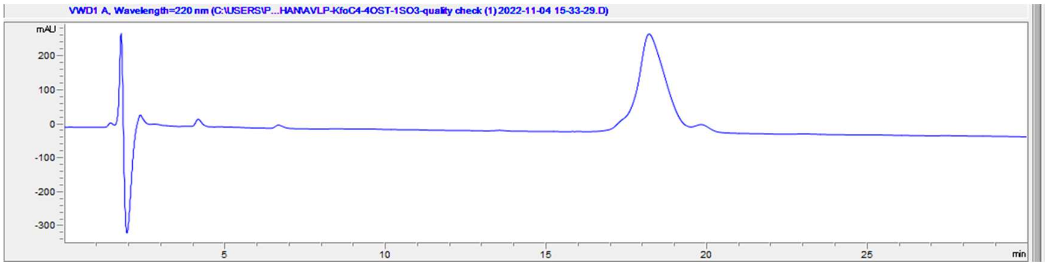

MS

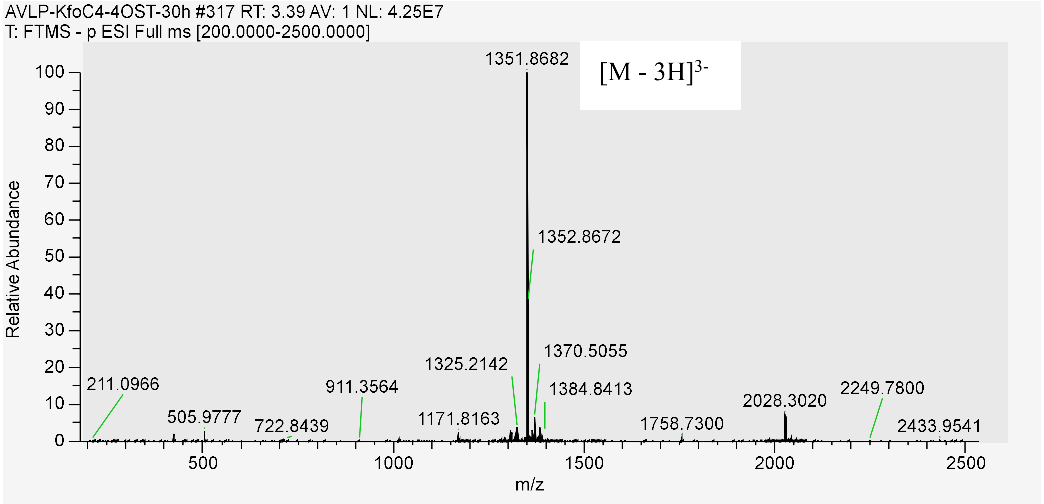

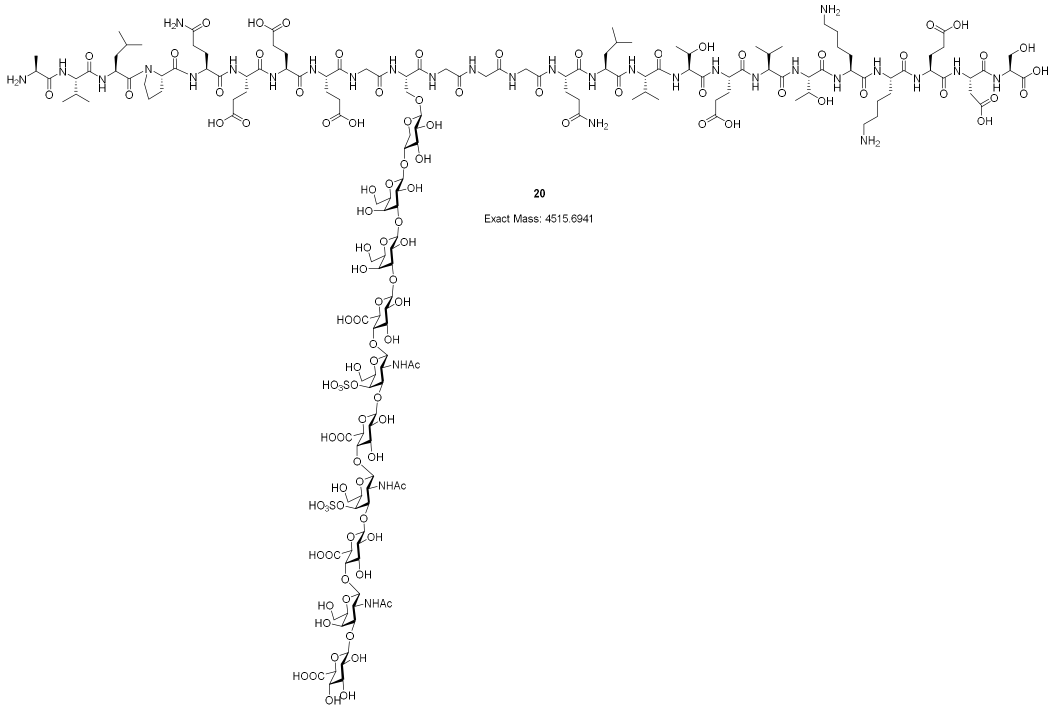

20

Exact Mass: 4515.6941

^{1}H NMR (800 MHz, D_2O)

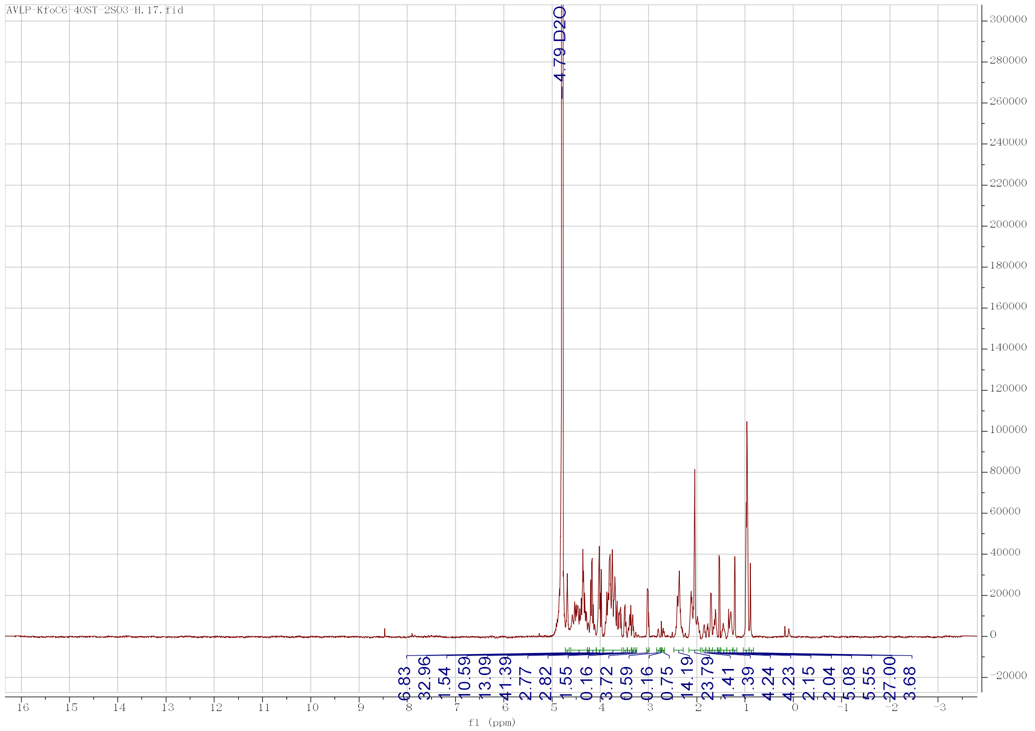

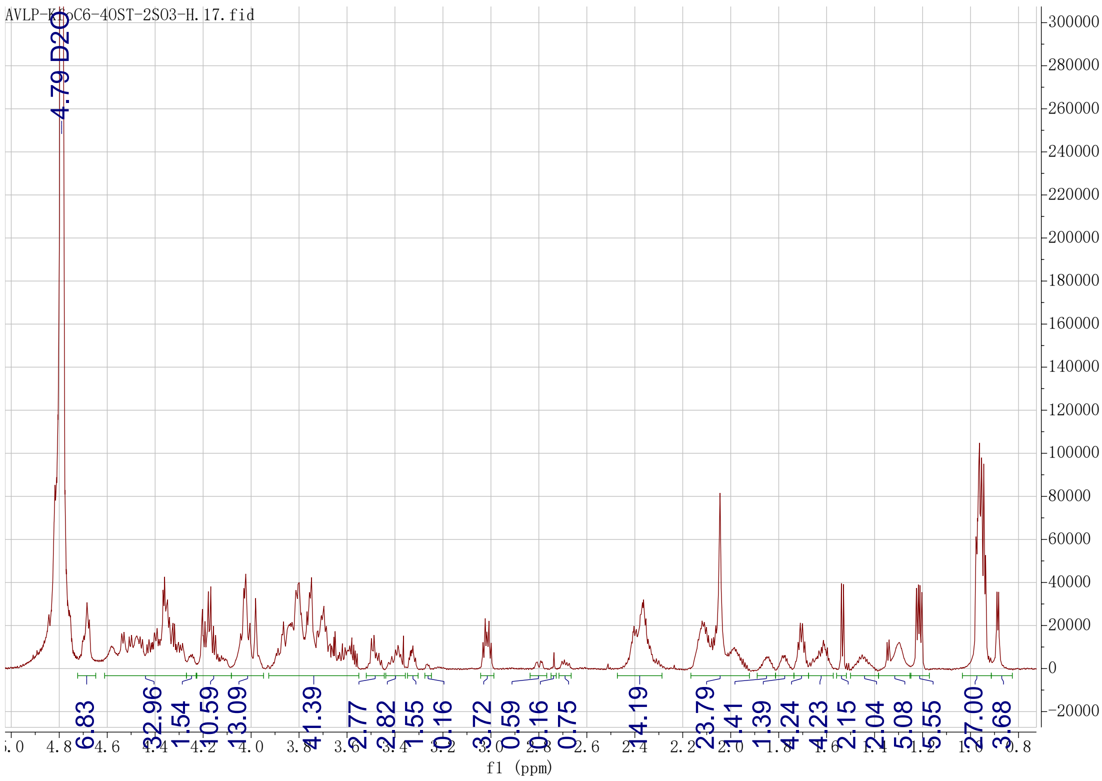
4.79 D2O
6.83
32.96
1.54
10.59
13.09
41.39
2.77
2.82
1.55
0.16
3.72
0.59
0.16
0.75
14.19
23.79
1.41
1.39
4.24
4.23
2.15
2.04
5.08
5.55
27.00
3.68
f1 (ppm)

COSY (800 MHz, D_2O)

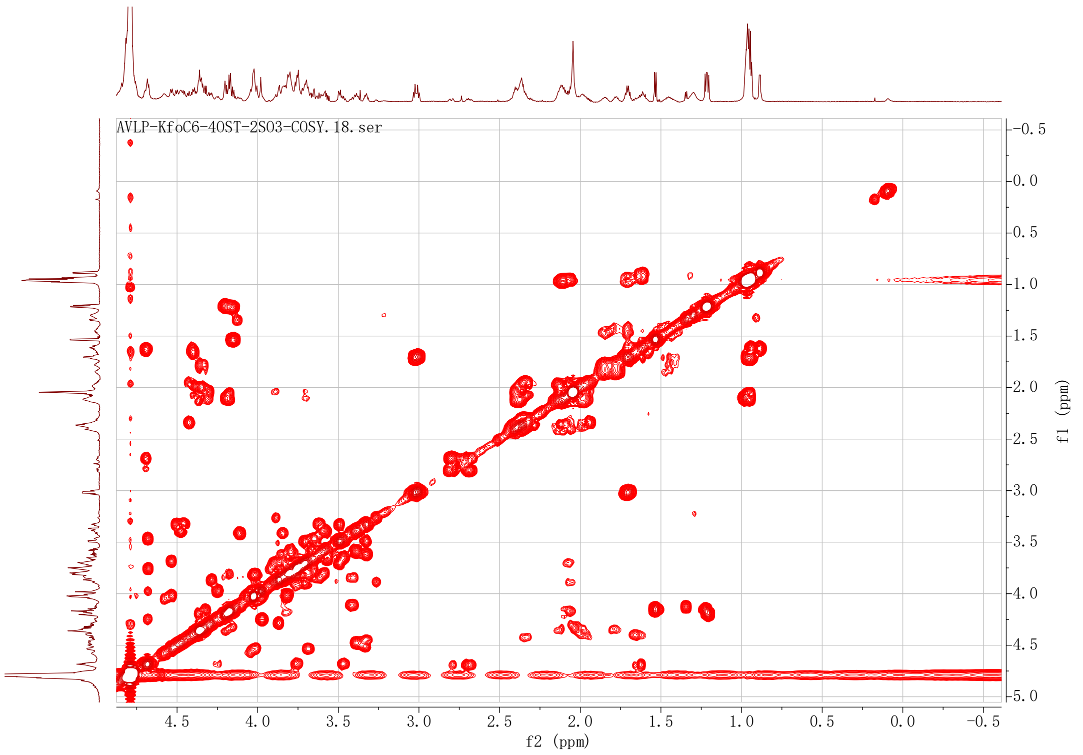

HSQC (800 MHz, D_2O)

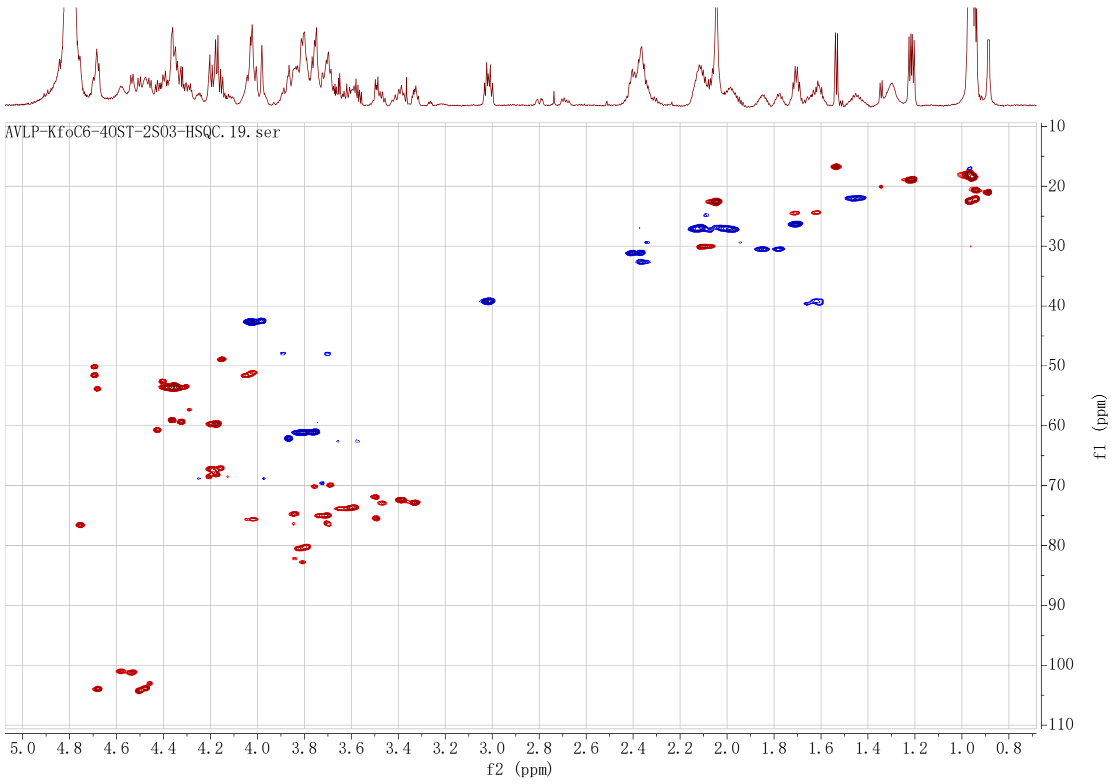

Coupled HSQC (800 MHz, D_2O)

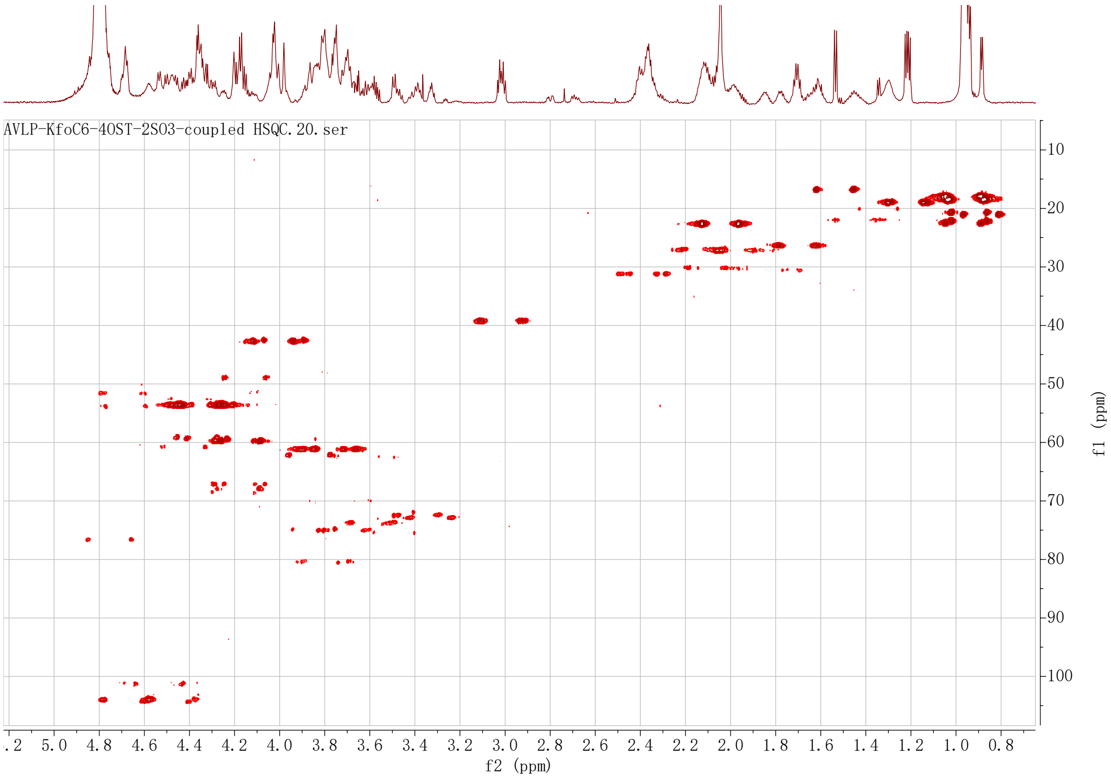

HPLC

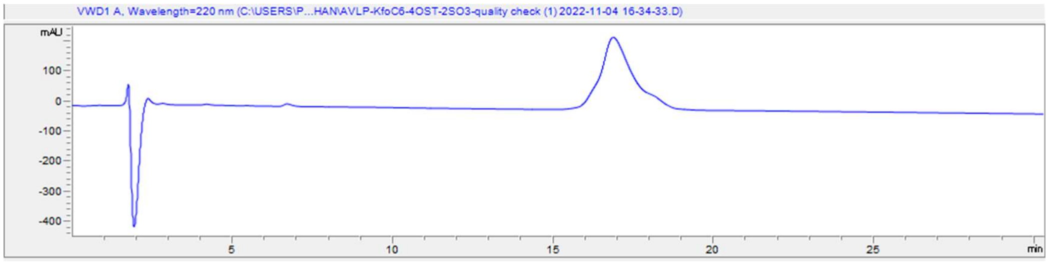

MS

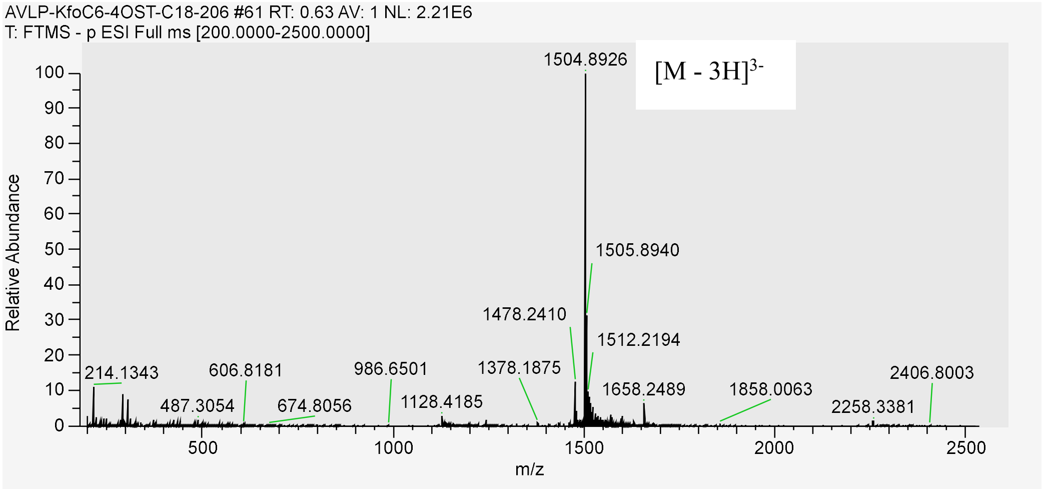

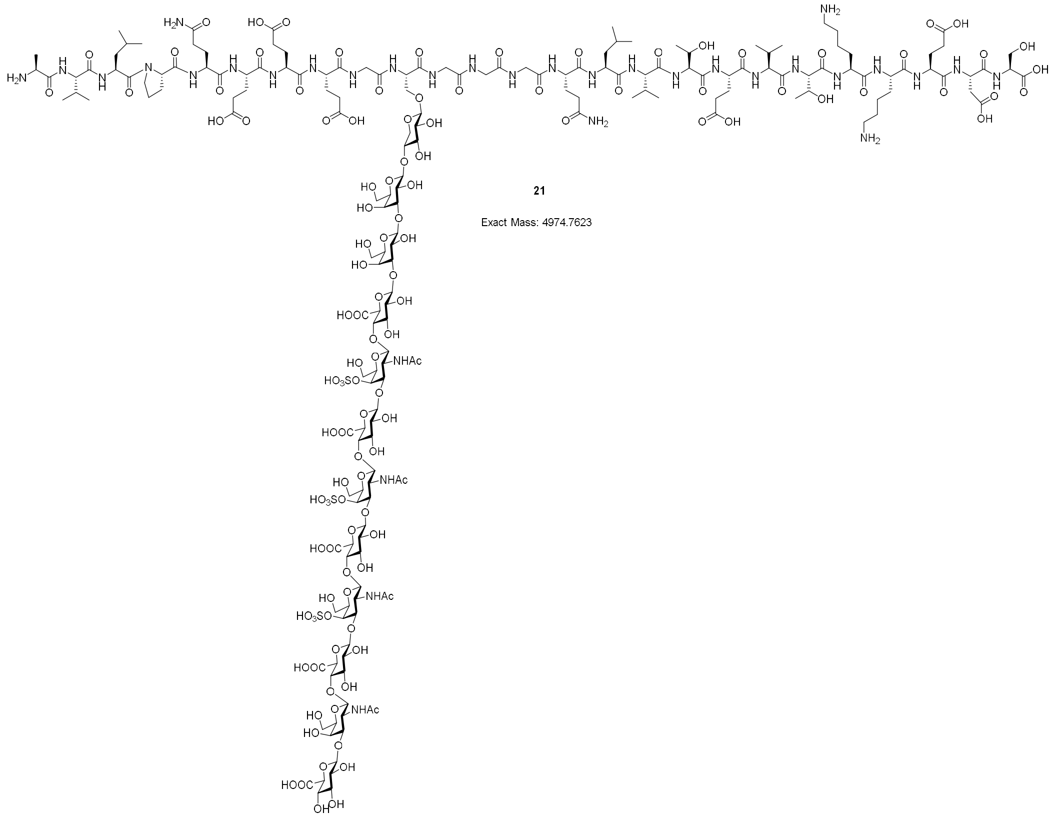

21

^{1}H NMR (800 MHz, D_2O)

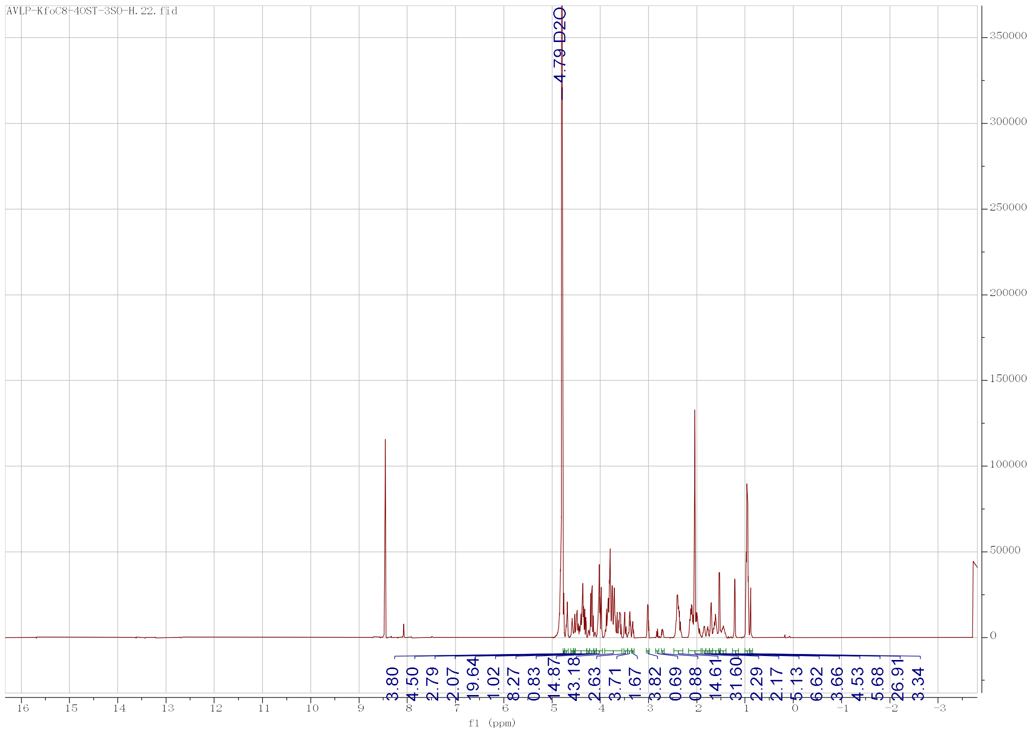

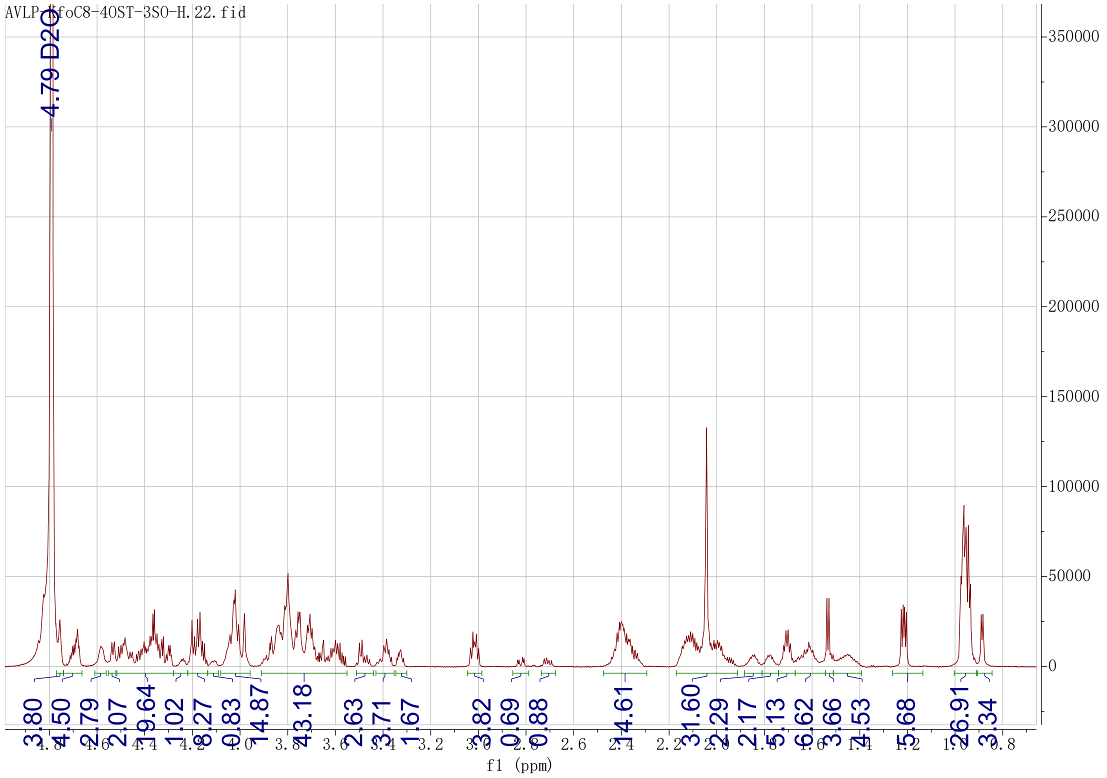
AVLP-IfoC8-4OST-3SO-H.22.fid
4.79 D2O
f1 (ppm)

^{13}C NMR (201 MHz, D_2O)

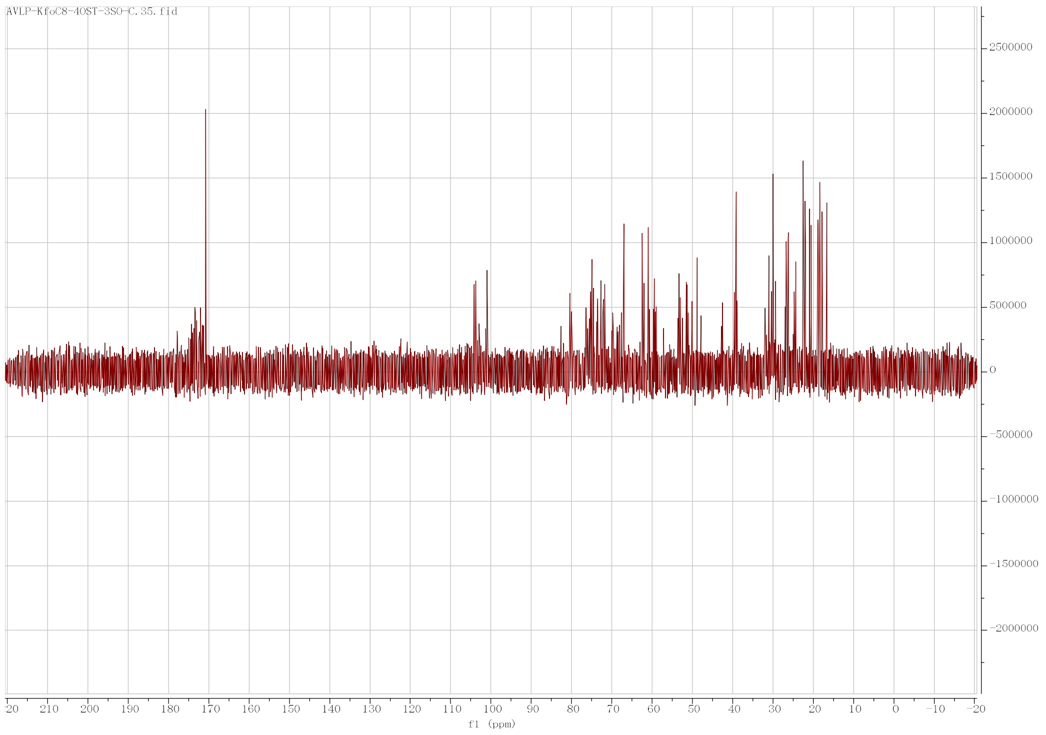

COSY (800 MHz, D_2O)

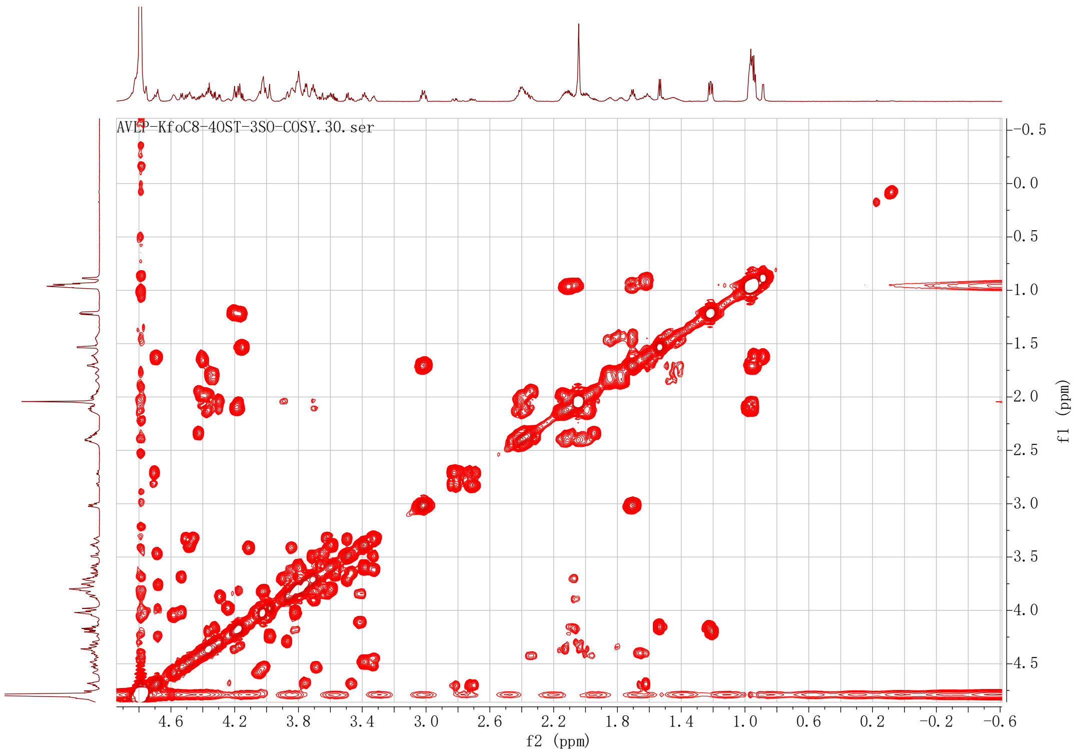

HSQC (800 MHz, D_2O)

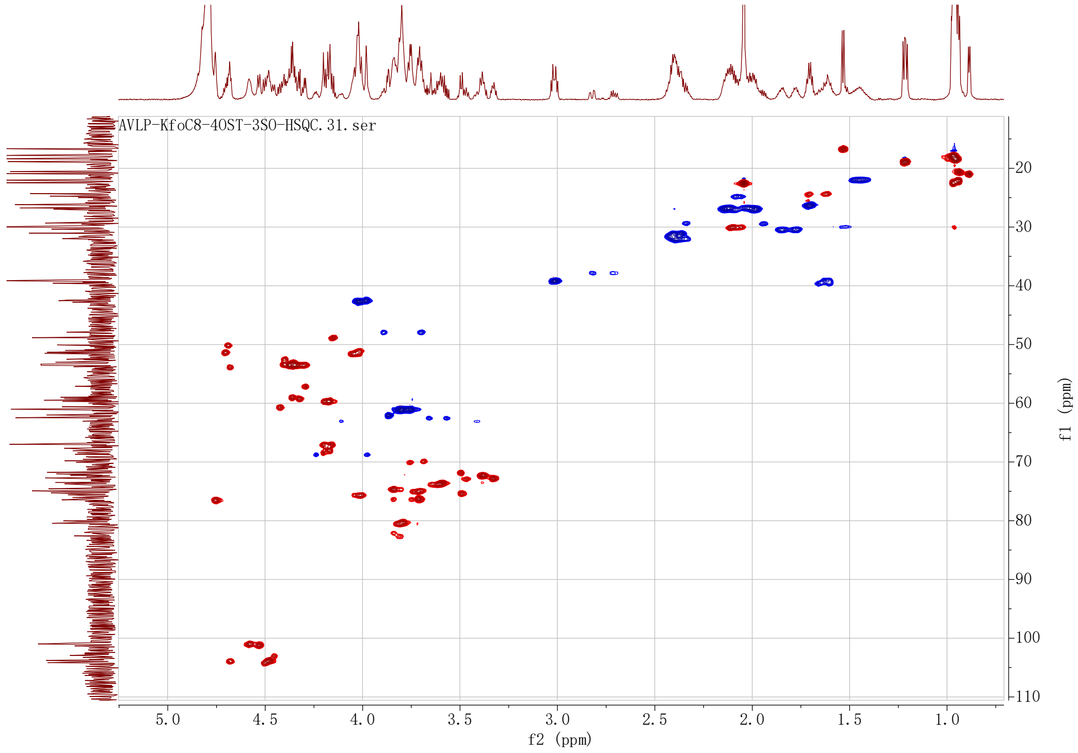

Coupled HSQC (800 MHz, D_2O)

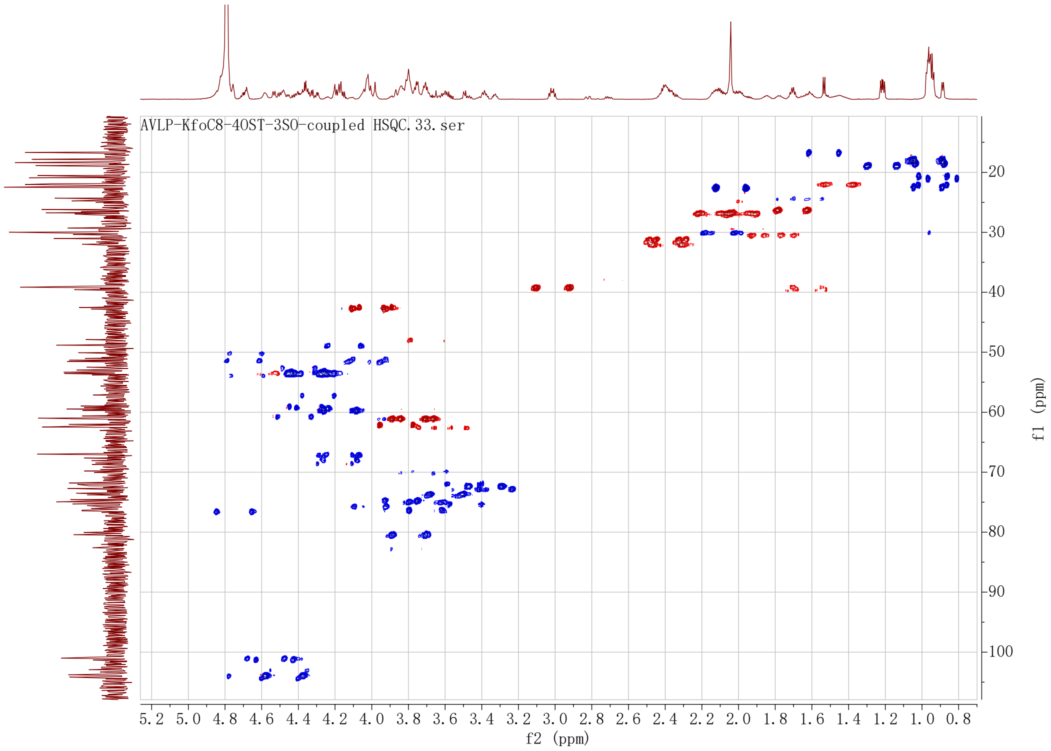

HMBC (800 MHz, D_2O)

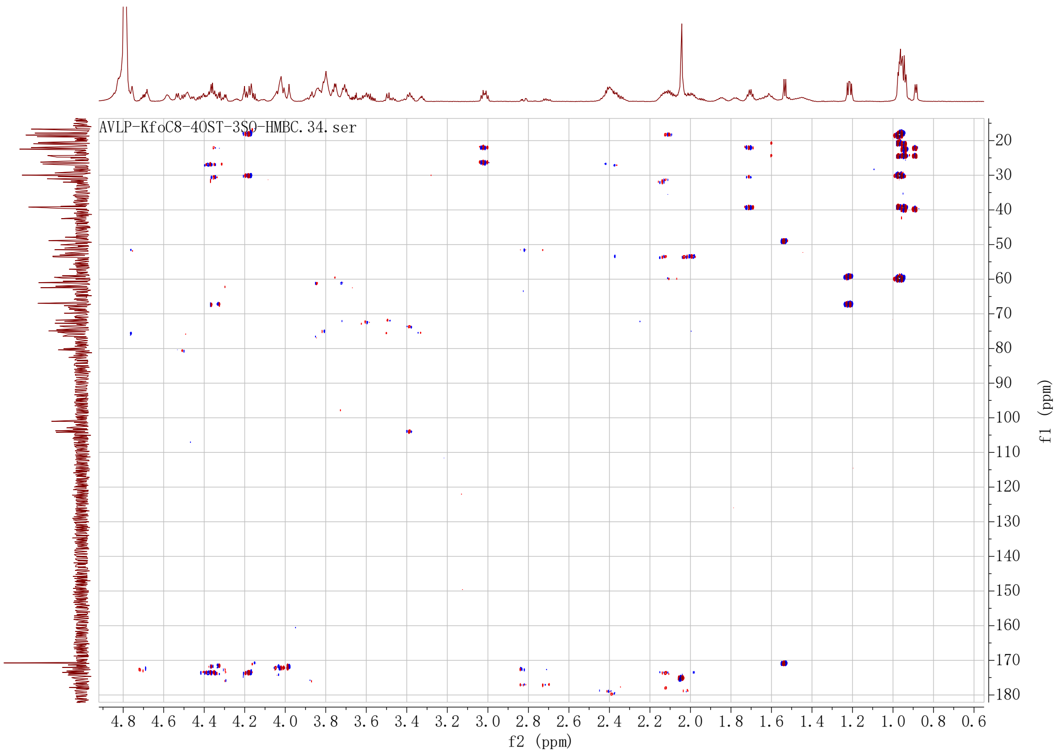

HPLC

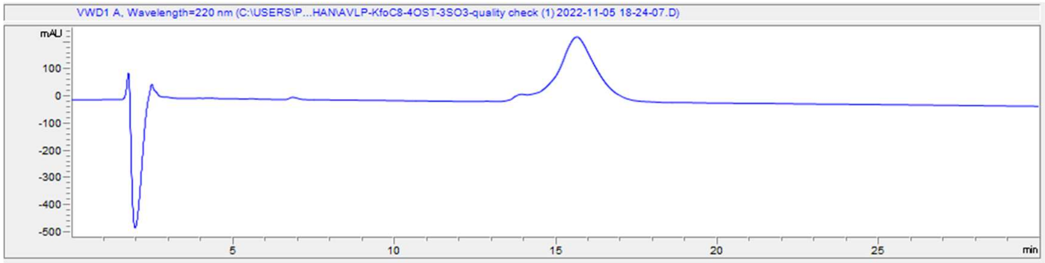

MS

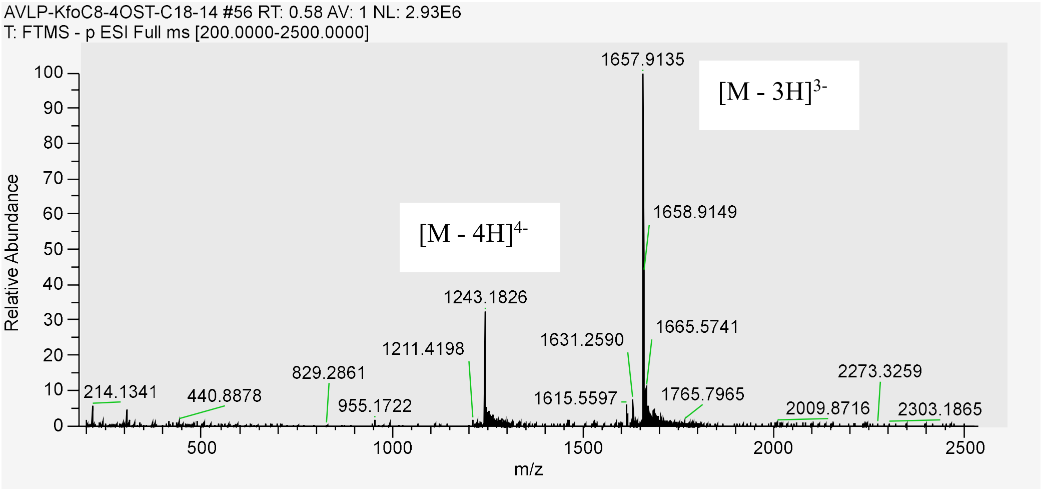

Supplement: Supporting info [file NIHMS2049806-supplement-Supporting_info.pdf]
